# Charting the landscape of graphical displays for meta-analysis and systematic reviews: a comprehensive review, taxonomy, and feature analysis

**DOI:** 10.1186/s12874-020-0911-9

**Published:** 2020-02-07

**Authors:** Michael Kossmeier, Ulrich S. Tran, Martin Voracek

**Affiliations:** Department of Basic Psychological Research and Research Methods, School of Psychology, University of Vienna, Liebiggasse 5, A-1010 Vienna, Austria

**Keywords:** Meta-analysis, Systematic reviews, Research synthesis, Network meta-analysis, Data visualization, Graphical display, Funnel plot, Forest plot, L’Abbé plot, Galbraith plot

## Abstract

**Background:**

Data-visualization methods are essential to explore and communicate meta-analytic data and results. With a large number of novel graphs proposed quite recently, a comprehensive, up-to-date overview of available graphing options for meta-analysis is unavailable.

**Methods:**

We applied a multi-tiered search strategy to find the meta-analytic graphs proposed and introduced so far. We checked more than 150 retrievable textbooks on research synthesis methodology cover to cover, six different software programs regularly used for meta-analysis, and the entire content of two leading journals on research synthesis. In addition, we conducted Google Scholar and Google image searches and cited-reference searches of prior reviews of the topic. Retrieved graphs were categorized into a taxonomy encompassing 11 main classes, evaluated according to 24 graph-functionality features, and individually presented and described with explanatory vignettes.

**Results:**

We ascertained more than 200 different graphs and graph variants used to visualize meta-analytic data. One half of these have accrued within the past 10 years alone. The most prevalent classes were graphs for network meta-analysis (45 displays), graphs showing combined effect(s) only (26), funnel plot-like displays (24), displays showing more than one outcome per study (19), robustness, outlier and influence diagnostics (15), study selection and *p*-value based displays (15), and forest plot-like displays (14). The majority of graphs (130, 62.5%) possessed a unique combination of graph features.

**Conclusions:**

The rich and diverse set of available meta-analytic graphs offers a variety of options to display many different aspects of meta-analyses. This comprehensive overview of available graphs allows researchers to make better-informed decisions on which graphs suit their needs and therefore facilitates using the meta-analytic tool kit of graphs to its full potential. It also constitutes a roadmap for a goal-driven development of further graphical displays for research synthesis.

## Background

Data visualization is essential for the exploration of any empirical data and for the communication of statistical results in science in general [1–3]. Graphical displays allow to present complex statistical information in a comprehensive way. They are especially suited for the illustration of data comparisons, patterns, trends, and relationships [4].

Meta-analysis is the statistical approach for quantitatively combining and synthesizing the results of two or more empirical studies with identical or comparable research questions [5, 6]. Its principal aim is to critically assess and to summarize the available data answering to a specific research hypothesis. Meta-analysis is highly relevant across all fields of empirical science, which invariably depend on the accumulation of empirical evidence over time, in order to support or to reject hypotheses and theories.

Meta-analytic data and results represent complex data structures. Their interpretation relies on the evaluation and integration of a multitude of statistical information, for example, whole collections of effect sizes, their respective confidence intervals, meta-analytic study weights, the influence of single studies on the summary effect, or associations of effect sizes with study characteristics. For these combined reasons, meta-analysis may be considered a prime candidate domain for the application of data-visualization methods. Visualization may facilitate insight into, and allow drawing firmer conclusions from, complex meta-analytic data.

**Fig. 1:**
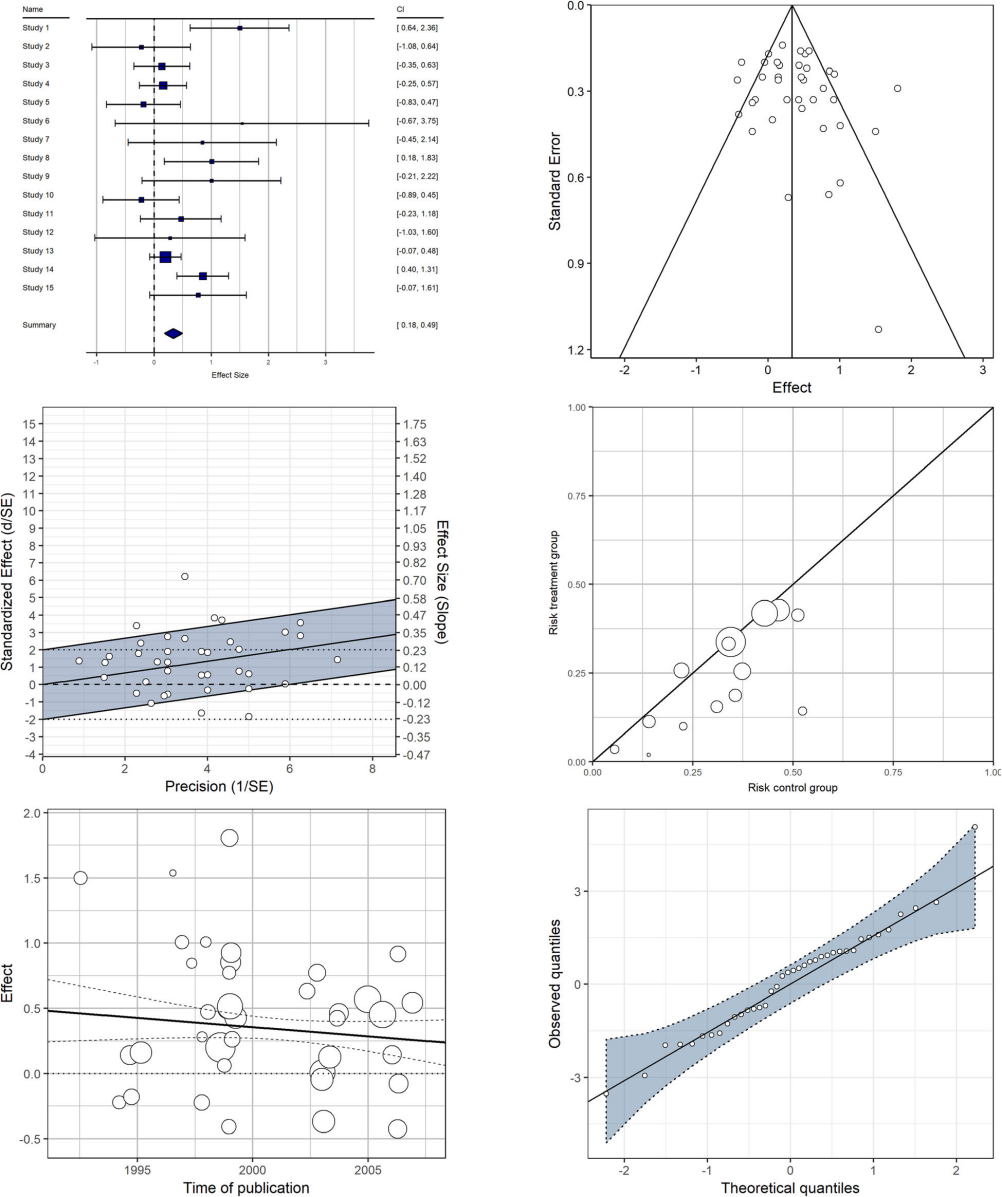
Examples of the graphical display types most frequently covered in textbooks on meta-analysis methodology. Forest plot (top left), funnel plot (top right), Galbraith/radial plot (middle left), L’Abbé plot (middle right), bivariate scatter plot with meta-regression line (bottom left), normal Q-Q plot (bottom right)

As a matter of fact, a considerable number of graphical displays is available, which have been designed and introduced with the purpose of visualizing key topics of interest in meta-analysis. These include the estimation of summary effects and their statistical uncertainty, outlier and sensitivity analysis, the exploration of between-study effect heterogeneity, and the assessment of publication bias and related forms of evidence distortion. Some examples of widely known, and most frequently used, options for displaying meta-analytic data are shown in Fig. 1.

Several reviews of general graphing options available for meta-analysis have been published over the years, partly as book chapters [7–9], partly as journal articles [10–12]. In addition, two reviews about specific graphical displays for network meta-analysis are available [13, 14]. The currently most comprehensive of these general reviews covers about 50 data graphical display variants, with a focus on just four well-known meta-analytic displays, namely, the forest plot, the funnel plot, the L’Abbé plot, and the Galbraith plot [11].

**Fig. 2:**
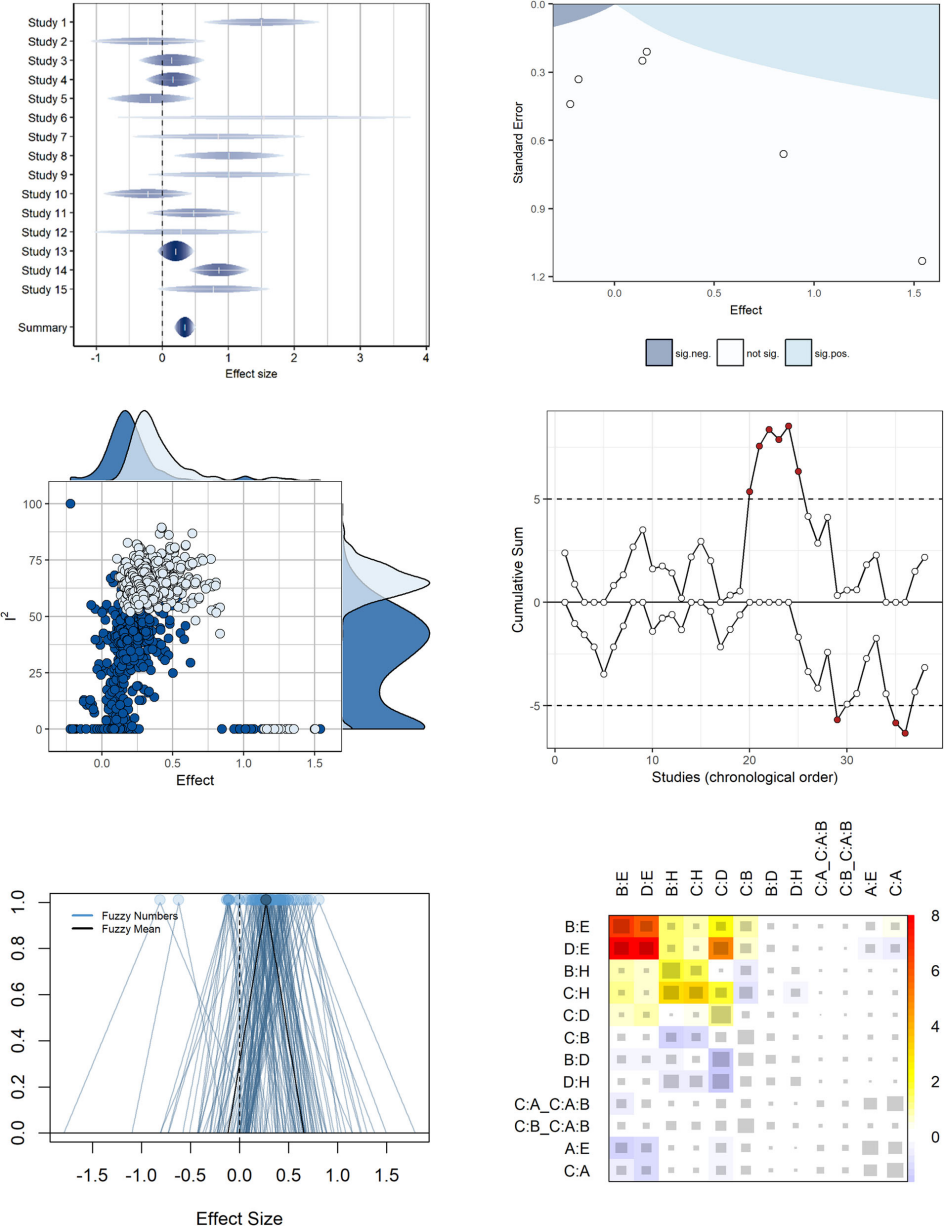
Selected examples of novel (recently proposed) graphical displays for meta-analytic data. Rainforest plot (top left), additional evidence funnel plot (top right), GOSH plot (middle left), CUMSUM chart (middle right), fuzzy number plot (bottom left), netheat plot (bottom right)

Data visualization for meta-analysis, as part of meta-analytic methodology, is subject to ongoing research and rapid development. Consequently, a multitude of novel data-visualization methods have been proposed during the past few years, in the fashion of a “graphics explosion” in the field of research synthesis. We estimate that the number of distinct graphical displays, including variants of these, certainly has more than tripled since the year 2005, and likely has almost doubled since the year 2010. Figure 2 shows several examples of such just recently proposed displays for meta-analysis. Hence, a comprehensive, up-to-date, general overview, accounting for these most recent developments, is overdue. In addition, previous reviews of graphical displays for meta-analysis were not conducted in a systematic manner, but rather were foremost based on expert opinion and mere awareness of available meta-analytic graphical displays.

Here, we aim to provide an up-to-date, and systematically gathered, compilation of available graphical displays, and to categorize and to describe this large and diverse body of data-visualization methods specific to meta-analysis and systematic reviews. Thereby, the present review serves two purposes: first, it represents a comprehensive and structured overview for researchers on available visualization methods and their utility to visualize different aspects of meta-analytic data. Second, it reveals possible gaps in the current landscape of data-visualization methods and therefore, in a way of a roadmap, may supply impulses for future developments of visualization methods in the context of research synthesis approaches.

## Methods

### Evidence search strategy: graphical displays

For the inclusion of a graphical display in our collection, three criteria had to be fulfilled. First, the graphical display depicts statistical information from empirical meta-analytic data. Second, the graph is not merely an aesthetic variant of a display already included in the corpus of graphical displays (i.e., differences that do not alter the statistical information conveyed). Third, the graph had to be used in the past in a published reference to depict meta-analytic data. For assembling the collection of graphical displays for meta-analytic data, we utilized a systematic, multi-tiered evidence search strategy, as described in the following.

First, we checked all retrievable monographs on meta-analysis methodology (as detailed in section 2.2) cover to cover. Second, we checked Google Scholar (end of May 2018) for relevant scientific publications, using the search string *allintitle: “visualization* “*OR “display* “*OR “graph* “*OR “plot* “*“meta-analysis* “*OR “meta analyses“*. All retrieved 134 results were screened whether they contained graphical displays eligible for inclusion in our corpus. Third, we conducted a Google Image search (end of May 2018) with the same search string as above and checked all retrieved results. Fourth, we investigated all plotting options for meta-analytic data in three widely used specialized meta-analysis software programs: CMA (Comprehensive Meta-Analysis; version 3) [15], Revman (version 5.3) [16], and Mix 2.0 [17]. Relatedly, three widely used multi-purpose statistical software programs were checked: Stata [18], NCSS (version 12) [19], and R [20]. These latter searches included all 102 R packages dedicated to meta-analysis, as listed in the *CRAN (Comprehensive R Archive Network) Task View: Meta-Analysis* [21]. Fifth, we conducted cited-reference searches (end of April 2018) in Google Scholar for the two most comprehensive and most cited review articles on graphical displays for meta-analysis [10, 11] and checked all resulting citing references. Sixth and finally, two authors (MK, MV) hand-searched all articles in all issues of the journal *Research Synthesis Methods* (from 2010 onwards) and screened all abstracts of the journal *BMC Medical Research Methodology* (from 2001 onwards) containing the search string (meta-analysis OR meta-analyses) AND (display OR plot OR graph* OR visual*) up to May 2018. As of this writing, *Research Synthesis Methods* is the only journal exclusively dedicated to the methodology of meta-analysis and systematic reviews, and *BMC Medical Research Methodology* has a long tradition in publishing methodological approaches to meta-analysis and systematic reviews in the health sciences. Both journals regularly publish papers on new data-visualization methods in this context (e.g., [22–26]).

For each graphical display included in our corpus, we ascertained the year in which the graph was first introduced in print or, alternatively, was used in the context of meta-analysis, along with the corresponding published source reference (if applicable and retrievable).

### Evidence search strategy: monographs on meta-analysis

We exclusively considered monographs mainly concerned with meta-analytic methods. We therefore excluded textbooks containing merely single chapters on meta-analysis (such as broad-ranging textbooks on quantitative research methods), as well as books not primarily concerned with meta-analytic methods, but rather describing the results of meta-analytic applications (with the exception of the earliest ones of this type, which typically comprise method development and application conjointly; e.g., [27]). If there was more than one version of a book over the years, we additionally considered any later (revised or expanded) editions. Importantly, we also considered non-English sources, with the only language-based restriction (i.e., non-eligibility) being that the monograph was in a language written in a non-Latin alphabet (Arabic, Chinese, Hebrew, Japanese, Korean, or Russian). Of meta-analytic software manuals, we included early and influential (mostly, commercial) ones, but did not add the manuals and documentations of the now more than 100 R packages related to meta-analysis (see above) to the list of monographs. Finally, we also did not consider journal special issues on meta-analytic methodology. Superficially, these might be deemed as rather similar to edited textbooks. A main difference is, however, that edited books generally are planned ahead (in terms of their focus, scope, coverage, and contributors) to a greater extent, as well as more centrally, than usually is the case for topical issues of journals. One might therefore expect less overlap and redundancy across chapters of edited books than across individual articles of a journal special issue.

The starting point for the literature search for monographs on meta-analysis was an existing corpus of such textbooks held in possession of one author (MV). This corpus was complemented and updated by the following two search strategies (up to May 2018): first, by systematically searching Amazon.com, using the search string *allinanchor:“Meta analysis” OR “Meta analyses” site:**amazon.com* in Google; second, by searching WorldCat, the largest online meta-catalogue of library catalogues from all over the globe, for books with the word meta-analysis appearing in the title (in ten languages: English, Danish, Dutch, French, German, Italian, Norwegian, Portuguese, Spanish, and Swedish).

These multi-tiered search strategies combined resulted in a corpus of 153 textbooks, published between 1980 and 2018, and totalling about 38,000 book pages. The earliest book in the corpus was the pioneering meta-analytic monograph on the efficiency of psychotherapeutic treatment [27], for which Glass and colleagues had developed core methods of meta-analysis, published in the same book. The most recent book in the corpus was a textbook on network meta-analysis published in March 2018 [28]. All books in the final corpus were checked cover to cover for content regarding graphical representations in meta-analysis and systematic reviews, and the relevant information was independently extracted by two authors (MK, MV). This mainly included which graphs were displayed, which graphs were explicitly discussed, and which suggestions were provided regarding their use.

The complete bibliography of textbooks on meta-analytic methodology can be found in Additional File 1. It is, to our knowledge, the most comprehensive one of its kind. For this reason, apart from documenting a main information source for our review work, it constitutes a basic, and readily usable, bibliographic resource of its own, for future scholarly investigations (e.g., research on the history of meta-analysis, or the evolution and propagation of its methodology during the past four decades).

### Taxonomy of data-visualization methods in research synthesis (meta-analysis and systematic reviews)

For the sake of clarity, we grouped all displays according to a derived classification system, or taxonomy (Table 1). This classification system was developed by using a bottom-up strategy; that is, the graphical displays were classified into categories which, in the end, contained similar plots. The categories were derived, and graphical displays assigned to categories, by all three authors in an iterative, consensual process. There is a plethora of possible ways to construct such classification systems, and the one proposed here is one of many imaginable ones. In fact, some plots might arguably be assigned to more than one category (e.g., a cumulative meta-analysis plot showing the summary effect and its confidence interval for additional studies added over time is a “cumulative meta-analysis and time trends” plot, and at the same time is a “forest plot-like” display). However, for the sake of a clear and structured presentation of the multitude of available graphical displays, a categorization appeared practical.

**Table 1:** A taxonomy of graphical displays for meta-analysis

Category	Key properties of displays in this category
01 - Forest plot-like	Display of study effects, their confidence intervals, and a summary effect or study-group summary effects.
02 - Funnel plot-like	Bivariate display of study effect size (or functions thereof) and study precision (or functions thereof).
03 - Continuous effect moderators	Display of the association of effect sizes and continuous covariates for the explanation of between-study heterogeneity.
04 - Robustness, outlier, and influence diagnostics	Illustrates the sensitivity of meta-analytic estimates, or the influence of single studies/outliers.
05 - Cumulative meta-analysis and time trends	Depicts the cumulative development of a meta-analytic estimate over time.
06 - Effect-size distribution	Depicts study effect-size distributions, but no meta-analytic summary statistics.
07 - Study or subgroup characteristics	Plot of study (or study-group) features other than effect size, standard error, or meta-analytic estimates.
08 - More than one effect size per study (multivariate)	Depicts more than one effect size per study.
09 - Combined effect(s) only	Displays meta-analytic summary effect(s), but not study-level effects.
10 - Study selection and *p*-value based	Displays primarily based on the *p* values of study results; usually for publication bias assessment.
11 - Network meta-analysis	Displays specifically proposed to visualize results of a network meta-analysis.

Within each category, we present different variants of the same display together. Variants of the same display were defined as conveying the same information, but, in addition, graphically showing some further, or alternative, statistical information. Aesthetic differences alone were not counted as distinct variants. Moreover, to avoid redundancies, we did not consider variants of variants. For example, the rainforest plot is a recently proposed variant of the forest plot [23] and, as such, was added to the graph collection. However, variants of the rainforest plot (e.g., a subgroup rainforest plot) were not added to the collection, because the rainforest plot itself is already a variant, and a subgroup forest plot (as a variant of the forest plot) was already included.

On the lowest level of the (two-level or three-level) taxonomy, graphical displays are presented in chronological order, using the publication year of the reference in which they were first proposed.

### Description (feature analysis) of meta-analytic visualization methods

The full set of meta-analytic displays was described according to a variety of different functionality dimensions by two authors (MK, MV). For this purpose, we iteratively and consensually derived and used 24 functionality features (Table 2). Each graph feature in this functionality space was rated as either present, partly present, or not present (coded on an ordinal scale: 2, 1, 0) for a specific plot or plot variant (in all cases, “not present” was equivalent to “not applicable”). In inconclusive cases, the plot or plot variant shown in Additional File 2 formed the basis for the description. After completion of the initial rating process, cases of rater disagreement were jointly resolved in discussion.

**Table 2:** The 24 graph-functionality features used to describe the 208 retrieved graphical displays for meta-analysis

Functionality features of meta-analytic plots	
01 - Displays summary outcome point estimate	
02 - Displays summary outcome interval estimate	
03 - Displays heterogeneity summary estimates (e.g., *I*^2^, *Q*; also, inconsistency in network meta-analysis)	
04 - Displays individual study effect-size point estimates	
05 - Displays individual study effect interval estimate	
06 - Displays individual study meta-analytic weight/precision/*N* (including contribution of comparisons in network meta-analysis)	
07 - Displays individual study names or identifiers	
08 - Displays more than one outcome per study	
09 - Displays individual study significance dichotomously (i.e., significant vs. not)	
10 - Displays individual study significance continuously (i.e., allows to assess how close a study *p* value was to statistical significance thresholds)	
11 - Informs about the likelihood, or posterior distribution, of meta-analytic parameter values	
12 - Suitable to display association of effect sizes with categorical study features	
13 - Suitable to display association of effect sizes with continuous study features	
14 - Suitable to display individual study or study-group features (additionally or exclusively)	
15 - Suitable and informative for small-sized meta-analyses (10 studies or less)	
16 - Suitable and informative for medium-sized meta-analyses (say, about 50 studies)	
17 - Suitable and informative for large-sized meta-analyses (say, hundreds of studies)	
18 - Suitable to assess small-study effects/publication bias and other forms of biases	
19 - Suitable to assess the temporal development of meta-analytic estimates	
20 - Suitable to assess an excess of between-study (or study-group) effect heterogeneity (also, inconsistencies in network meta-analysis)	
21 - Suitable to assess assumptions about the distribution of estimates (e.g., normality of effects)	
22 - Suitable to assess the robustness of summary effect(s)	
23 - Suitable to assess the robustness of heterogeneity statistics (e.g., *I*^2^, τ^2^, *Q*)	
24 - Suitable to identify influential studies (i.e., outliers, leverage points)	

## Results

The compilation of graphical displays for meta-analytic or systematic-review data totalled 208 plots. These 208 plots can be further divided into 114 (54.8%) distinct stem displays vs. 94 (45.2%) variants of these. Table 3 lists these graphical displays for meta-analytic data in their entirety, including their categorization (Section 3.2), source reference (if applicable and retrievable), and the year of introduction. Graph vignettes, with complete presentations and short descriptions for all 208 graphical displays, can be found in Additional File 2.

**Table 3:** Annotated taxonomy of 208 retrieved graphical displays for meta-analysis

ID	Name	Year	Source reference
1.1	Confidence interval plot, caterpillar plot	1978	[23]
1.2	Forest plot	1982	n.a.
1.2.1	Subgroup forest plot	n.a.	n.a.
1.2.2	Summary forest plot	n.a.	n.a.
1.2.3	Shrinkage plot, Bayesian forest plot	n.a.	n.a.
1.2.4	Raindrop plot	2003	[29]
1.2.5	Limits of equivalence forest plot	2007	[30]
1.2.6	Confidence distribution plot	2010	[31]
1.2.7	Rainforest plot	2015	[22]
1.2.8	Thick forest plot	2015	[23]
1.2.9	Contour-enhanced forest plot	2017	[32]
1.3	Odd man out plot	1988	[33]
1.4	Fuzzy number plot	2016	[34]
1.4.1	Fuzzy number plot with subgroups	2016	[34]
2.1	Funnel plot	1984	[35]
2.1.1	Subgroup funnel plot	1984	[35]
2.1.2	Regression test funnel plot	1997	[36]
2.1.3	Funnel plot with weighted mean, median and mode	1998	[37]
2.1.4	Trim-and-fill funnel plot	2000	[38]
2.1.5	Significance contour-enhanced funnel plot	2008	[39]
2.1.6	Additional evidence contours funnel plot: Summary effect significance	2012	[40]
2.1.7	Additional evidence contours funnel plot: Heterogeneity	2012	[40]
2.1.8	Additional evidence contours funnel plot: Summary CI width	2012	[41]
2.1.9	Additional evidence contours funnel plot: Summary CI lower/upper bound	2012	[41]
2.1.10	Additional evidence contours funnel plot: Limits of equivalence	2012	[41]
2.1.11	Additional evidence contours funnel plot: Summary effect	2015	[42]
2.1.12	Funnel plot with imputed non-statistically-significant unreported effects	2015	[43]
2.1.13	Meta-analyser funnel plot	2016	[44]
2.1.14	Funnel plot with summary diamond	n.a.	n.a.
2.1.15	Funnel plot with bias-corrected effect sizes	n.a.	n.a.
2.2	Galbraith plot (a.k.a. radial plot)	1988	[45]
2.2.1	Subgroup Galbraith plot	1988	[45]
2.2.2	Galbraith plot with Egger regression	1997	[36]
2.2.3	3D Galbraith plot	n.a.	n.a.
2.3	Gravity plot	2005	[46]
2.4	Doi plot	2016	[47]
2.5	Begg and Mazmudar test display	n.a.	n.a.
2.5.1	Begg and Mazmudar test display with subgroups	n.a.	n.a.
3.1	Scatterplot of effect size with continuous moderator	1977	[6]
3.1.1	Binned scatterplot	n.a.	n.a.
3.2	Meta-regression plot	1977	[6]
3.2.1	Meta-regression weight bubble plot	n.a.	n.a.
3.2.2	Meta-regression plot with subgroups	n.a.	n.a.
3.2.3	Meta-regression plot with confidence or prediction bands	n.a.	n.a.
3.2.4	Meta-regression plot including effect-size confidence intervals	1984	[35]
3.2.5	Surface plot	1998	[48]
3.2.6	Trim-and-fill meta-regression plot	2012	[49]
3.2.7	Meta-analytic regression/classification tree	2014	[50]
3.2.8	Meta-analytic partial dependence plot	2017	[51]
3.3	Time-to-event data: Meta-STEPP	2016	[52]
4.1	Tau square sensitivity plot	1993	[53]
4.1.1	Tau square sensitivity plot with posterior distribution	n.a.	n.a.
4.2	Leave-one-out sensitivity lineplot	1998	[37]
4.2.1	Leave-one-out sensitivity forest plot	2000	[54]
4.3	Baujat plot	2002	[55]
4.4	Number of additional participants required to obtain significance plot	2003	[56]
4.5	Influence plot	2010	[57]
4.6	Study influence and outlier diagnostic lineplots	2010	[57]
4.7	Metaplot	2010	[58]
4.8	GOSH plot	2012	[59]
4.9	Outlier probability plot	2014	[60]
4.10	Forward plot	2016	[61]
4.11	Impact of unmeasured confounding sensitivity plot	2017	[62]
4.12	Tau square estimator sensitivity plot	n.a.	n.a.
4.13	Cross-validated residual plot	n.a.	n.a.
5.1	Effect-size time-series plot	1984	[35]
5.1.1	Quality control chart: X bar chart	2010	[63]
5.1.2	Quality control chart: CUMSUM chart	2010	[63]
5.2	Cumulative meta-analysis plot	1992	[64]
5.2.1	Subgroup cumulative meta-analysis	n.a.	n.a.
5.2.2	Cumulative meta-analysis with monitoring boundaries	1997	[65]
5.2.3	Cumulative summary effect ratio plot	1999	[66]
5.2.4	Cumulative heterogeneity plot	2004	[67]
5.2.5	Cumulative Bayes factor plot	2016	[68]
5.2.6	Predicted Bayes factor for an additional study plot	2017	[69]
5.3	Plot of cumulative results	2015	[70]
5.4	Comparison of meta-analyses: Initial vs. subsequent evidence scatterplot	n.a.	n.a.
6.1	Histogram	n.a.	n.a.
6.1.1	Histogram, weighted	n.a.	n.a.
6.1.2	Histogram, subgroups	n.a.	n.a.
6.2	Boxplot	n.a.	n.a.
6.2.1	Boxplot, weighted	n.a.	n.a.
6.3	Stem-and-leaf display	n.a.	n.a.
6.3.1	Stem-and-leaf display, subgroups	n.a.	n.a.
6.4	Dot plot	n.a.	n.a.
6.5	Density plot	n.a.	n.a.
6.5.1	Density plot, weighted	n.a.	n.a.
6.5.2	Density plot, subgroups	n.a.	n.a.
6.6	Collection of study-effect likelihoods	1993	[71]
6.7	Normal quantile-quantile (Q-Q) plot of effect sizes	1998	[72]
7.1	Bar chart	n.a.	n.a.
7.2	Pie chart	n.a.	n.a.
7.3	Line/dot chart with continuous covariate	n.a.	n.a.
7.4	Risk of bias plot	2008	[73]
7.4.1	Risk of bias summary plot	2008	[73]
7.5	Harvest plot	2008	[24]
7.6	PRISMA flow chart	2009	[74]
7.7	Comparison of meta-analyses: Veritas plot	2009	[75]
7.8	Error matrix display	2010	[76]
7.8.1	3D error matrix plot	2010	[76]
7.9	Effect-direction plot	2013	[77]
7.10	Evidence-map bubble plot	2016	[78]
7.11	Dendrogram of meta-cluster analysis	2017	[79]
8.1	Dichotomous outcomes: L’Abbé plot	1987	[80]
8.1.1	Dichotomous outcomes: Subgroup L’Abbé plot	1987	[80]
8.1.2	Dichotomous outcomes: L’Abbé plot with summary effect contours	1997	[81]
8.1.3	Dichotomous outcomes: L’Abbé weight bubble plot	n.a.	n.a.
8.1.4	Dichotomous outcomes: Baseline graph	n.a.	n.a.
8.2	Time-to-event data: Study survival curves	1989	[82]
8.3	Bivariate meta-analysis plot	1993	[71]
8.3.1	Bivariate meta-analysis plot with confidence ellipse	1993	[71]
8.3.2	Cross-hairs scatterplot	2016	[83]
8.3.3	Cross-hairs scatterplot with subgroups	2016	[83]
8.4	Dichotomous outcomes: ROC plot	1993	[84]
8.4.1	Dichotomous outcomes: ROC plot with summary ROC curve	1993	[84]
8.4.2	Dichotomous outcomes: Cross-hairs plot	2010	[85]
8.4.3	Dichotomous outcomes: ROC plot with subgroups	n.a.	n.a.
8.5	Dichotomous outcomes: Treatment benefit vs. control plot per 100 patients	2001	[86]
8.6	Dichotomous outcomes: Olliaro display	2010	[87]
8.6.1	Dichotomous outcomes: Subgroup Olliaro display	2010	[87]
8.7	Dichotomous outcomes: Threshold plot	2016	[88]
8.8	Meta-analytic Bland-Altman plot	n.a.	n.a.
9.1	Glass distributional overlap display	1976	[5]
9.2	Time-to-event data: Summary survival curve	1989	[82]
9.2.1	Time-to-event data: Subgroup survival curves	1989	[82]
9.3	Summary path diagram (SEM)	1991	[89]
9.4	Genetic data: Summary *q* value plot	2002	[90]
9.5	Genetic data: Summary Q-Q plot	2003	[91]
9.6	Hattie barometer display	2008	[92]
9.7	Comparison of meta-analyses: FEM vs. REM summary estimates	2011	[93]
9.8	Comparison of meta-analyses: Heterogeneity	2011	[93]
9.9	Comparison of meta-analyses: Tau square estimates	2011	[93]
9.10	Genetic data: Meta-analytic circos plot	2012	[94]
9.11	Fishbone diagram	2017	[95]
9.12	Evidence flowers	2018	[96]
9.13	Likelihood, prior, or posterior distribution plot	n.a.	n.a.
9.13.1	Bootstrap chart	n.a.	n.a.
9.13.2	Likelihood, confidence, or posterior region plot for two parameters	n.a.	n.a.
9.13.3	Predictive distribution or interval plot	n.a.	n.a.
9.14	Dichotomous outcomes: Fagan nomogram	n.a.	n.a.
9.14.1	Dichotomous outcomes: Probability-modifying plot	n.a.	n.a.
9.15	Genetic data: Meta-analytic Manhattan plot	n.a.	n.a.
9.15.1	Genetic data: Meta-analytic Miami plot	n.a.	n.a.
9.15.2	Genetic data: Meta-analytic regional association plot	n.a.	n.a.
9.16	Genetic data: Meta-analytic volcano plot	n.a.	n.a.
9.17	Genetic data: Position-summary line plot	n.a.	n.a.
9.18	Genetic data: Summary heat map	n.a.	n.a.
9.19	Meta-analytic neuroimaging plot	n.a.	n.a.
10.1	Schweder-Spjøtvoll plot	1982	[97]
10.2	Publication-probability plot (selection model)	1992	[98]
10.3	Sensitivity contour plot (selection model)	2000	[99]
10.4	Test of excess significance alpha sensitivity plot	2007	[100]
10.5	Caliper test display	2008	[101]
10.6	Plot of truncated normal distribution	2008	[102]
10.7	Genetic data: P-M plot	2012	[103]
10.8	*p*-curve	2014	[104]
10.8.1	*p*-curve sensitivity plot	2016	[105]
10.9	*p*-value plot for selection bias (selection model)	2015	[106]
10.10	Treatment effect plot (selection model)	2015	[106]
10.11	Albatross plot	2017	[107]
10.11.1	Albatross subgroup plot	2017	[107]
10.12	Weighted effect-size density (selection model)	n.a.	n.a.
10.13	Maximum-bias forest plot	n.a.	n.a.
11.1	Network graph	2002	[108]
11.1.1	Flow-of-evidence graph	2013	[109]
11.1.2	3D network graph	2017	[110]
11.1.3	Matrix display of treatment comparisons	n.a.	n.a.
11.2	Contrast forest plot	2008	[111]
11.2.1	Summary forest plot matrix	2014	[112]
11.2.2	Summary forest plot table	2014	[112]
11.2.3	Network indirect path decomposition forest plot	2014	[26]
11.2.4	Invariant interval forest plot	2017	[113]
11.3	Checkerboard unit plot	2008	[114]
11.4	Treatment dissimilarity table plot	2008	[115]
11.4.1	MDS inconsistency plot	2008	[115]
11.5	Ranking table plot	2009	[116]
11.5.1	Barplot of ranking probabilities	2011	[13]
11.5.2	Median rank chart	2014	[112]
11.5.3	Ranking and ranking probability for different outcome preferences	2016	[117]
11.5.4	Ranking scatterplot for single outcome	2013	[14]
11.6	Inconsistency plot	2009	[118]
11.7	Rankogram (cumulative or absolute)	2009	[119]
11.7.1	Probability to be within a range of the best treatment plot	2011	[11]
11.8	Network meta-regression plot	2009	[120]
11.8.1	Contribution network meta-regression plot	2018	[121]
11.8.2	Heat network meta-regression plot	2018	[121]
11.9	Diagnostic network plot (leverage, deviance, residuals)	2009	[120]
11.10	Comparison-adjusted funnel plot	2013	[14]
11.11	Contribution plot	2013	[25]
11.12	Netheat plot	2013	[25]
11.13	Shade plot for contrast weights of treatment comparison	2013	[122]
11.14	Shade plot for *p* values of treatment comparison	2013	[122]
11.15	Minimal parallelism vs. mean path length scatterplot	2013	[109]
11.16	Hsu mean-mean plot	2013	[122]
11.17	Clustered ranking plot for two outcomes	2013	[14]
11.18	Network risk of bias chart	2014	[123]
11.18.1	Network risk of bias direct evidence contribution chart	2014	[123]
11.19	Bland-Altman heterogeneity plot	2015	[106]
11.20	Rank heat plot	2016	[124]
11.21	Hasse diagram	2017	[125]
11.22	Partial orderings for two outcomes plot	2017	[125]
11.22.1	Biplot of partial orderings for more than two outcomes	2017	[126]
11.23	Invariant region plot	2017	[113]
11.24	Bivariate network meta-analysis crosshair plot	2018	[127]
11.25	Covariate distribution plot	2018	[121]
11.26	Covariate contribution scatterplot	2018	[121]
11.27	Covariate contribution heat plot	2018	[121]
11.28	Network meta-analysis survival plot	n.a.	n.a.

Shown for each display are its ID, the year of its introduction (or earliest retrievable source) in the context of meta-analysis, and the corresponding source reference. If no year of introduction, along with the corresponding source reference, could be identified with reasonable degree of confidence, the respective cell entry is n.a. (not available). A full presentation of the total of 208 displays and display variants is provided in Additional File 2. For each graph, the first digit of its 2-digit (or 3-digit) ID number refers to the assigned category in the taxonomy (1: Forest plot-like, 2: Funnel plot-like, 3: Continuous effect moderators, 4: Robustness, outlier, and influence diagnostics, 5: Cumulative meta-analysis and time trends, 6: Effect-size distribution, 7: Study or subgroup characteristics, 8: More than one outcome per study (multivariate), 9: Combined effect(s) only, 10: Study selection and *p*-value based, 11: Network meta-analysis). The second and third digits are serial numbers, which (for the second digit) are assigned chronologically within each main category, and (for the third digit) within each stem display are assigned for variants of the respective display

In the following sections, the compilation of the data-visualization methods available for research synthesis approach is ordered, grouped, and described. In Section 3.1, the historical development of these data-visualization methods and their coverage in meta-analytic textbooks over time is illustrated. In Section 3.2, an overview of the graph collection is provided, using the derived categorization system (taxonomy). In Section 3.3, the entirety of graphs is described with respect to a suite of different functionality features (graph-feature analysis).

### Historical development of data visualization in meta-analysis

**Fig. 3:**
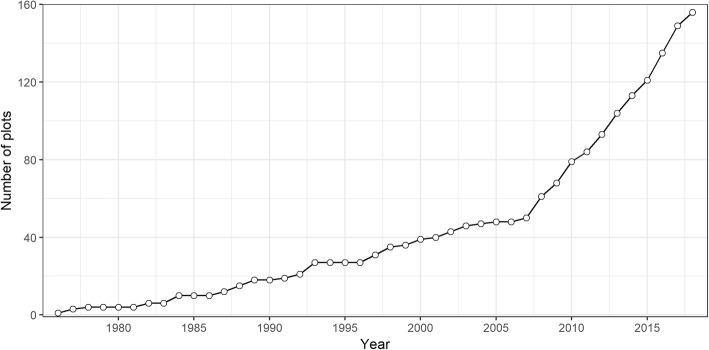
Evolution of graphical displays for meta-analytic data over time. For each year, the cumulative number of graphical displays available for meta-analytic data is shown. From the total of 208 ascertained plots, the 156 plots with retrievable year of introduction (see Table 3) are included

Data visualization in meta-analysis is a topic of ongoing development, and many graphs have been proposed rather recently (Fig. 3). For 156 of all 208 display a year of introduction could be identified with a reasonable degree of confidence (Table 3) and these form the basis for the following historical descriptions of graph development. The number of available meta-analytic graphs has grown exponentially in the past. On average, the number of graphs (*N*) has grown roughly by 9% annually (*N* = e^1.17 + 0.094*(Year – 1975)^). However, as is evident from Fig. 3, approximately starting in 2007, the number of newly proposed graphical displays has steeply increased. Up to 2006, the graph compilation has grown close to linear, with on average 1.5 novel displays introduced each year. Since 2007, this figure now is 6 times larger, with an average of 9 new displays per year.

**Fig. 4:**
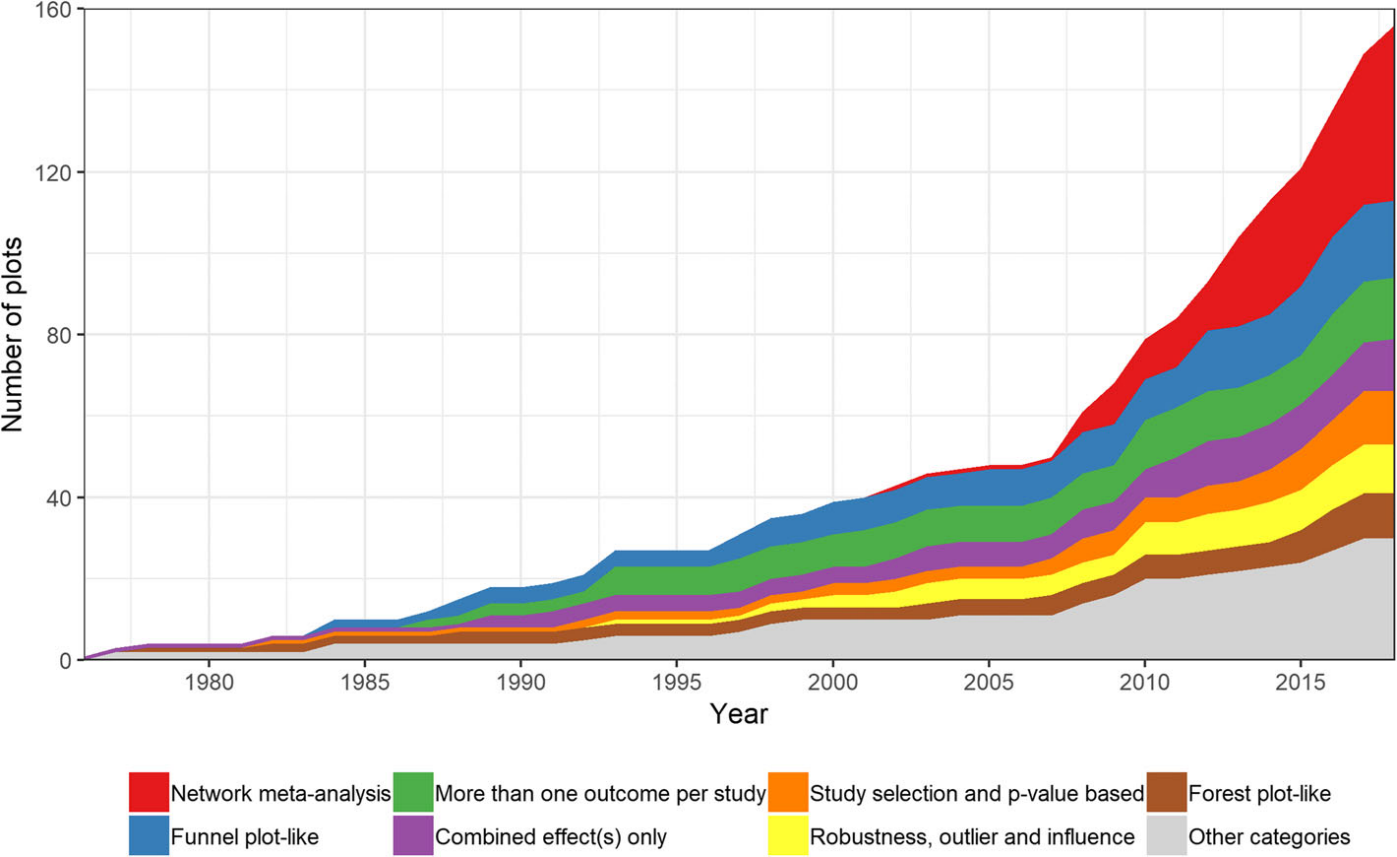
Evolution of graphical displays for meta-analytic data over time, differentiated by graph category. For each year, the cumulative number of available graphical displays for meta-analytic data is shown. The composition of available graphs is shown by colored areas, according to the specific category within the taxonomy of graphs. Of the total of 208 ascertained plots, only those 156 plots with retrievable year of introduction (see Table 3) are shown. The four categories containing the fewest graphs are merged to “other categories”

Looking at the growth of the graph compilation by different graph categories, it is apparent that one – but not exclusively – driving factor for the graphics explosion in meta-analysis in the last decade was the quite large number of novel graphical displays particularly developed for the framework of network meta-analysis (Fig. 4).

However, despite this large number of newly proposed graphs in recent years, most of the graphs actually used in published meta-analyses [22] date back to the very beginnings of meta-analysis in the 1970s and 1980s (e.g., the forest plot was introduced not later than 1982, funnel plots in 1984, the L’Abbé plot in 1987, and the radial plot in 1988).

**Fig. 5:**
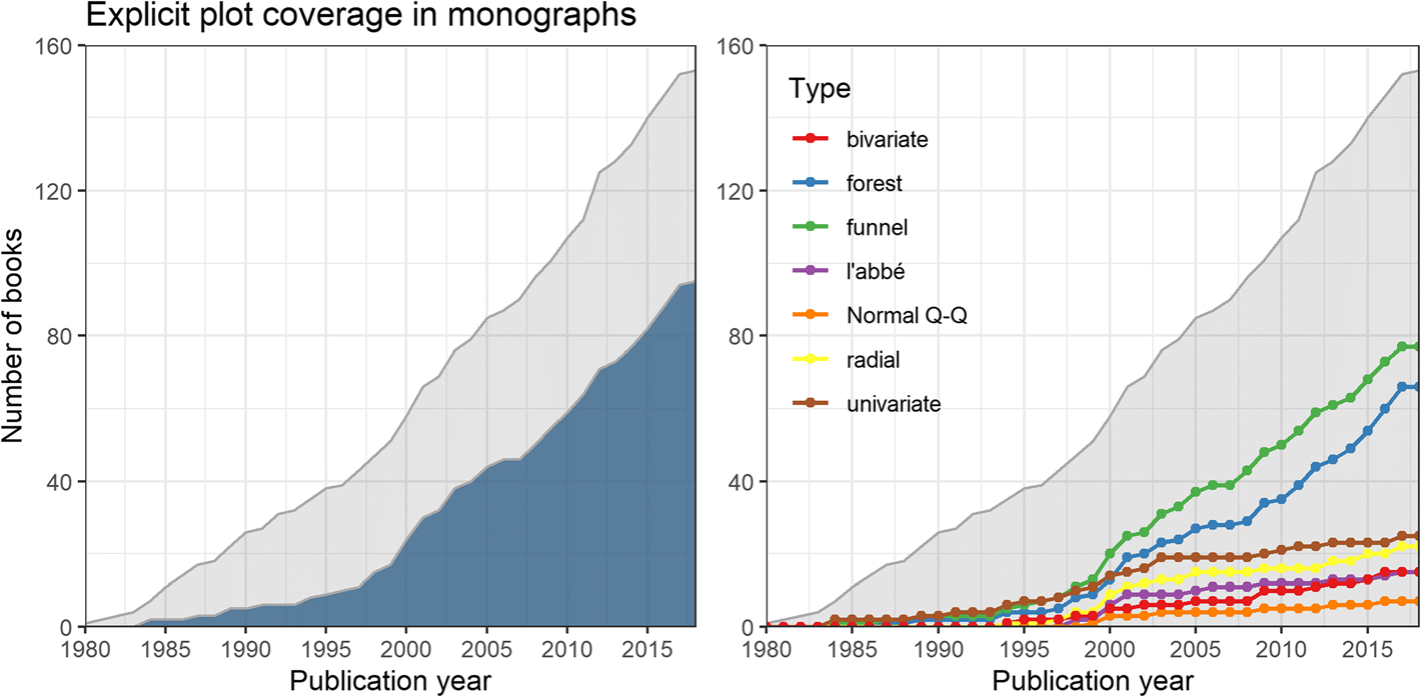
Coverage of graphical displays in textbooks on meta-analytic methodology over time. Cumulative number of textbooks on meta-analytic methodology explicitly covering at least one graphical display over time (left), or, for the seven most prevalent display types, individually (right). The gray shaded area indicates the total cumulative number of textbooks available at a certain time point

To assess the popularity of graphs and data visualization in meta-analysis in a novel way, we looked at their implicit and explicit coverage in all textbooks on meta-analytic methodology. A graph was deemed as explicitly covered, if there was a dedicated presentation and explanation of the graph in the book, whereas for implicit coverage it would be sufficient when the graph was used to show meta-analytic data without any graph-specific explanations. Of all 153 books, 20 (13.1%) show a meta-analytic graphical display on their cover. Overall, 95 (62.1%) of the books at least cover one graph explicitly (Fig. 5), while 129 (84.3%) cover one or more plots at least implicitly.

By far the most prevalent explicitly covered displays (Fig. 5) are the funnel plot and its variants (50.3%) and the forest plot and its variants (43.1%), followed by univariate displays illustrating the distribution of effect sizes (16.3%; e.g., boxplots, histograms, or stem-and-leaf plots), the Galbraith plot (a.k.a. radial plot) and its variants (14.4%), the L’Abbé plot (9.8%), bivariate scatter plots or meta-regression plots showing the association of effect sizes with a continuous covariate (9.8%), and the (normal) quantile-quantile plot (4.6%).

Explicit coverage has not been constant over time (Fig. 5). While explicit graph coverage in textbooks was rare in the first years of meta-analysis (up to the mid-1990s), coverage quickly increased to beyond 50% of all books available up to a specific year since the early 2000s. Descriptively, as indicated by their explicit coverage, the popularity of funnel and forest plots rose in the second half of the 1990s. Coverage then quickly increased from 15.8 and 10.5% (of all books available up to the year 1995) to 43.5 and 31.8% (of all books available in 2005), and to 48.6 and 38.6% (in 2015) for the funnel and forest plot, respectively. Therefore, the number of books covering these two iconic displays relatively grew at a much faster rate than the book corpus itself, illustrating their dissemination and propagation. The proportion of books explicitly covering any of the other most prevalent displays stayed rather constant or even declined; that is, the number of books covering these other plots relatively grew at a similar (or even slower) rate than the book corpus itself.

Compared to explicit coverage, by also considering implicit coverage, the prevalence of forest plots increased greatly from 43.1 to 62.7%, whereas the prevalence of funnel plots essentially stayed the same (50.3% vs. 52.3%). This indicates that funnel plots were hardly displayed in textbooks without being explained and covered explicitly at the same time, whereas this seemed not to be the case for forest plots. Implicit coverage was clearly more common than explicit coverage for bivariate displays of effect sizes and covariates (i.e., scatter plots: 26.1%) and univariate displays of effect-size distributions (e.g., histograms: 21.6%), which is less surprising, when considering their more general, not genuinely meta-analytic, nature.

### A taxonomy of available meta-analytic graphs

To arrive at a structured and ordered presentation of the graph compilation, each graph was categorized into one of 11 distinct graph categories (see Methods section). The most prevalent categories were *network meta-analysis* (45 displays), *combined effect(s) only* (26), and *funnel plot-like* (24), followed by *more than one outcome per study* (19), *robustness, outlier, and influence diagnostics* (15), *study selection and p-value based* (15), *forest plot-like* (14), *effect-size distribution* (13), *study or subgroup characteristics* (13), *continuous effect moderators* (12), and *cumulative meta-analysis and time trends* (12). An overview and summary of the graph compilation using these graph categories is given in the following. For presentations and brief descriptions of all the 208 graphical displays retrieved, see Additional File 2.

#### Forest plot-like graphical displays

The forest plot is probably the most iconic of genuine displays for meta-analytic data. Key characteristics are the depiction of summary and study-level effects, as well as interval estimates and a clear labelling of each study. Showing study effect sizes and their confidence intervals in a confidence interval plot (a.k.a. caterpillar plot) dates back at least to 1978 [128], while the first actual forest plot additionally depicting a meta-analytic summary estimate was first used not later than 1982 (for a historical overview, see [129]). Classic variations of the forest plot are the subgroup forest plot and the summary forest plot, showing and comparing additional or exclusive summary estimates of groups of studies. For Bayesian meta-analysis, a forest plot variant depicting posterior distributions or posterior intervals (a.k.a. shrinkage plots) for each study has regularly been used. An early, nowadays seldom-used, forest plot-like graph is the odd man out plot, visualizing effect-size areas for which at least a specified number of study confidence intervals overlap [33]. Forest plots with vertical lines indicating user-specified limits of equivalence have been used [30], which allow drawing conclusions regarding non-inferiority and equivalence testing on the study and summary-effect level [130]. Examples of recently proposed variants of the forest plot are the rainforest plot and the thick forest plot, which allocate more visual emphasis on those study effects which have been estimated with higher precision [23]. A novel, rather atypical, forest plot-like display is the fuzzy number plot, which shows study and summary effects and respective interval estimates using fuzzy numbers and which has specifically been proposed for large-scale meta-analyses with numerous studies, for which traditional forest plots are less suited [34].

#### Funnel plot-like graphical displays

Apart from the forest plot, the funnel plot is probably the most widely known genuine meta-analytic plot. Funnel plot-like displays can be seen as specialized scatter plots showing effect sizes (or functions thereof) on one axis and the studies’ standard error (or functions thereof) on the other axis. Typical plots in this category are the eponymous funnel plot [35] and the Galbraith plot (a.k.a. radial plot), essentially conveying the same information [45].

Remarkably, the funnel plot is the display in the graph compilation with the most distinct variants (15). Initially proposed for the assessment of potential publication bias, indicated through small-study effects, early variants include visual depictions of statistical methods concerned with publication bias, e.g., by showing studies imputed by the trim-and-fill algorithm [38], or fitted lines of regression tests (e.g., Egger regression test [36]). Specifically, in the last decade a large number of variants in the form of different contour-enhanced funnel plots have been introduced. The significance contour-enhanced funnel plot [39] allows incorporating information about the nominal (statistical) significance of studies into funnel plot assessment. Additional evidence contours [40] show the robustness of the nominal significance (or lack thereof) of the meta-analytic summary effect and the robustness of the magnitude of the estimated between-study heterogeneity with respect to a hypothetical additionally observed study. Further variants show the effect of a hypothetical additional study on the width, or upper and lower bounds, of the summary effect’s confidence interval [41], or on the magnitude of the summary effect [42].

#### Graphical displays for continuous effect moderators

One key aspect of meta-analysis is to explore the role of study covariates (or moderators) on the meta-analytic summary effect. Not surprisingly, scatter plots of study effect sizes and meta-regression plots were one of the first plots used in published meta-analyses [6]. Modern meta-regression plots include differently-sized symbols with respect to study precision or the meta-analytic study weight, and a fitted line and confidence bands for the estimated meta-analytic summary effect. Novel developments within this category came along with methodological advancements. A generalization of the trim-and-fill algorithm to meta-regression has been proposed, along with visualization of the corresponding corrected line of fit [49]. Machine-learning methods have recently been applied to meta-analysis and have led to the visualization of (meta-)regression trees [50] and illustrations of functional associations of single predictors with outcomes in meta-analytic random forests, using partial dependence plots [51].

#### Graphical displays for robustness, outlier, and influence diagnostics

The assessment of the sensitivity of meta-analytic results is another important field of application of meta-analytic graphs. One of the first genuine diagnostic plots has been the τ^2^ sensitivity plot [53], showing the trajectory of the meta-analytic summary effect for increasing values of τ^2^ (i.e., from the fixed-effect model, implying τ^2^ = 0, to a next to unweighted-average model for very large τ^2^ values). Graphs showing the meta-analytic summary effect for single studies being left out have been proposed as line charts [37] and, more commonly, as leave-one-out sensitivity forest plots [54]. The Baujat plot is a genuine meta-analytic plot to detect outliers and influence points by plotting the change of the summary effect for systematically leaving out one study at a time against the contribution of this study to the between-study heterogeneity statistic *Q* [55]. Widely known diagnostic plots for detecting outliers, leverage, and influence points in regression analysis have been proposed in the context of meta-analysis and meta-regression models in particular [57]. These displays include, among others, scatter and line plots of studentized deleted residuals, Cook’s distance values, and hat values.

The GOSH (Graphical Display of Study Heterogeneity) plot [59, 131] shows the results of combinatorial meta-analyses; i.e., meta-analyses of either all 2^k^ – 1 possible subsets of *k* studies, or a random sample of these. For each combination, the resulting meta-analytic summary effect and the *I*^2^ value are shown in a scatter plot, and study subsets including a certain study can be highlighted, thus revealing their influence on the summary effect or the estimated between-study heterogeneity. Forward plots accompany newly proposed methods to detect outlier studies, which monitor the effect on meta-analytic estimates by iteratively adding individual studies to increasingly heterogeneous sets of studies [61].

#### Graphical displays for cumulative meta-analysis and time trends

Questions regarding the development of evidence over time are typical for research synthesis. Time series of published effect sizes were displayed not later than in the mid-1980s [35]. Quality control charts, namely, x bar charts and CUMSUM (cumulative sum) charts, were proposed to identify changes in temporal trends and unusual observations in effect-size time-series data [63]. Cumulative meta-analysis plots show the development of the meta-analytic summary effect point and interval estimate over time in a classic forest plot-like display [64]. Sequential monitoring boundaries have been used and displayed in cumulative meta-analysis plots to assess whether additional evidence is needed [65]. While graphs showing the development of the meta-analytic summary effect have been used predominantly, variants showing meta-analytic heterogeneity statistics over time have been proposed as well [67]. In addition, the trajectory of evidence over time has been shown, using cumulative Bayes factors [68].

#### Graphical displays for effect-size distribution

Standard statistical graphs have primarily been used for the visualization of observed univariate effect-size distributions. These include histograms, boxplots, dot plots, stem-and-leaf displays, and kernel density plots. Weighted variants exist for histograms, boxplots, and density plots, to readily incorporate different precision and therefore different meta-analytic weights of studies. The (normal) quantile-quantile plot has been proposed as a suitable display to check statistical assumptions in the context of meta-analysis, including normality and homogeneity of effects and absence of publication bias [72].

#### Graphical displays for study or subgroup characteristics

Study characteristics other than effect sizes or precision have been displayed using standard statistical graphs. For continuous characteristics, the same plots have been used as to show effect-size distributions (see above), and, to visualize categorical study characteristics, bar or pie charts have been repeatedly used. Genuine meta-analytic plots within this category are the Cochrane risk of bias plot and the risk of bias summary plot [73], visualizing the overall and study-level risk of bias on several dimensions. The PRISMA (Preferred Reporting Items for Systematic Reviews and Meta-Analyses) flow chart [74] informs about literature search and study inclusion and exclusion details in the course of systematic reviews or meta-analyses. The veritas plot is a tool to compare several studies or study subgroups with respect to five different dimensions of relevance arranged in a pentagon (such as between-study heterogeneity, publication bias, evidence and quality gradings) [75]. Specialized displays to visualize the qualitative evidence and characteristics of a potentially diverse set of studies are the harvest plot [24], the error matrix display [76], the effect-direction plot [77], and the evidence-map bubble display [78].

#### Graphical displays for more than one outcome per study (multivariate)

Displays for more than one outcome per study were predominantly developed for visualizing two potentially dependent outcomes per study. Bivariate meta-analyses of two outcomes per study have been visualized with bivariate scatter plots no later than in the early 1990s, including a meta-analytic summary effect and confidence ellipses on the study or summary level [71]. A novel variant of these multivariate displays additionally shows the study-level confidence intervals in both outcomes simultaneously in a so-called multivariate cross-hairs plot [83].

Several multivariate displays were proposed for the visualization of meta-analyses of dichotomous outcomes. The L’Abbé plot is a genuine and classic meta-analytic plot, showing for each study the risk for an event in the treatment and control group in a scatter plot [80]. Variants with superimposed effect contours allow depicting study-level results and the meta-analytic summary effect either as risk ratio, odds ratio, or risk difference [81].

ROC (Receiver Operating Characteristic) plots and their variants are used to simultaneously display the specificity and sensitivity and the ROC curve on the study or the summary level [84]. Cross-hairs plots were proposed as an enhancement, showing the study-level confidence intervals for sensitivity and specificity [85]. For studies reporting sensitivity and specificity values for more than one threshold, recently proposed methods include visualizations of the estimated meta-analytic summary and study-level sensitivities and specificities for different diagnostic thresholds [88].

The Olliaro display was proposed to visualize absolute, as well as relative, effects of a treatment at the same time, showing the absolute failure rate of a treatment on one axis and the difference of failure rates with comparator treatments on the other axis [87].

#### Graphical displays for combined effect(s) only

As a rather heterogeneous category, displays exclusively showing meta-analytic summary or subgroup effects visualize quite different aspects of meta-analyses. The perhaps first genuine meta-analytic display visualized a single meta-analytic summary effect size by two overlapping normal distributions in 1976 [5]. Similarly, Hattie visualized the magnitude of single summary effects with a barometer-type infographic [92]. Fishbone diagrams [95] and evidence flowers [96] have recently been proposed as infographics to enable an overview of several summary findings concurrently (e.g., for different endpoints of interest).

Other typical graphs in this category show distribution-like displays of meta-analytic key parameters. Likelihood functions of meta-analytic parameters, prior, posterior, and posterior predictive distributions have been used to summarize Bayesian meta-analytic results. Likelihood functions or posterior densities for two parameters at the same time (predominantly, the summary effect and heterogeneity estimates) have been visualized, using two-dimensional contour plots or three-dimensional surface plots.

Summary survival curves have been displayed in meta-analyses of time-to-event data [82], whereas the summary results of meta-analyses of path and structural equation models have been visualized via path diagrams [89] not later than in the early 1990s.

Finally, there are several graphs for the depiction of meta-analyses of genetic data, displaying a large number of summary effects for different gene loci at the same time. Adopted displays from visualizing the results of primary studies include the meta-analytic Manhattan and Miami plots, the regional association plot, the volcano plot, and (summary) heat maps of gene expressions. A display genuinely proposed for meta-analysis of genetic data is the circos plot which shows meta-analytically derived summary estimates of down-regulated or up-regulated gene expressions for certain diseases in a circular display [94].

#### Graphical displays for study selection and *p* values

The majority of displays based on the *p* value of studies are related to methods for publication-bias assessment. A contour-line plot has been used to illustrate the sensitivity of the summary result to the parameters used in a selection model [99]. The test of excess significance [100] has been supplemented by a sensitivity display, showing the trajectory of the test result for different significance thresholds α. Formann used plots of truncated normal distributions to visualize the likely region of unpublished effects due to publication bias [102]. The caliper test display shows the distribution of *p* values associated with test statistics and highlights an abundance of just-significant results in a specific histogram [101]. Similarly, the *p*-curve display shows peculiarities of distributions of *p* values in the significance region and allows assessing the likely presence of *p*-hacking and the evidential value of a set of studies with a specific line plot [104]. The P-M display was proposed for genetic data, showing the *p* values of studies on one axis and the posterior probability that the effects exist in each study on the other axis [103].

A few further displays exist which focus on the presentation of study *p* values. One early account is the Schweder-Spjøtvoll display introduced in 1982, essentially showing the empirical distribution function of observed *p* values of a set of studies [97]. A recently proposed display based on *p* values is the albatross plot, showing the *p* values and sample sizes of studies in a scatter plot-like display. In addition, effect-size contours are overlain, showing for a specific effect size the resulting *p* values for all possible sample sizes, thereby allowing to assess the probable magnitude of the underlying effect, as well as an excess of between-study heterogeneity [107].

#### Graphical displays for network meta-analysis

Graphs specifically proposed for network or mixed-treatment comparison meta-analysis constitute the most recent, and already largest, category in the graph compilation. Basically, within this category four main types of network graphs can be distinguished.

First, there are graphs, showing which treatments are directly compared in the network. Examples for this type of graphs are network graphs, with vertices visualizing treatments and edges visualizing the number observed comparisons [108], and the flow-of-evidence graph, showing in a network graph for a certain treatment comparison which direct and indirect paths contribute to the network estimate [109]. Three-dimensional network plots, showing comparison-specific covariate values on a third axis within a network graph have recently been proposed [110].

Second, for the presentation of the results from a network meta-analysis, forest plots [111, 112] and funnel plots [14] have been adapted and enhanced for depicting network results on the treatment-contrast level.

Third, several displays exist for the visualization of estimated treatment rankings. Rankograms show for each treatment the estimated (absolute or cumulative) probability for each treatment ranking [119]. For two outcomes, a bivariate ranking scatter plot shows their ranking metrics simultaneously for each treatment [14]. Also, rank heat plots were proposed to compare treatment rankings on more than one outcome in a circular heat display [124]. Hasse diagrams were introduced to visualize rankings of treatments in a network graph with respect to more than one outcome, using partial ordering of treatments [125].

Fourth, there are a number of graphs which primarily visualize inconsistencies between directly and indirectly estimated treatment comparisons (analogously to effect heterogeneity in direct-evidence, univariate meta-analysis), as well as the contribution of direct and indirect treatment comparisons to the network estimates (analogously to study weights in direct-evidence, univariate meta-analysis). The network indirect path decomposition forest plot shows the contribution of indirect evidence and the resulting summary effects, considering only direct evidence, as compared to indirect evidence [26]. The netheat plot visualizes the contribution of different direct and indirect treatment comparisons, as well as inconsistencies related to specific comparisons in a matrix display [25]. Recently, several displays for network meta-regression were proposed, visualizing the contribution of single studies and ranges of covariate values to the network meta-regression estimates [121].

### Description of meta-analytic graphical displays by their functionality (feature analysis)

**Fig. 6:**
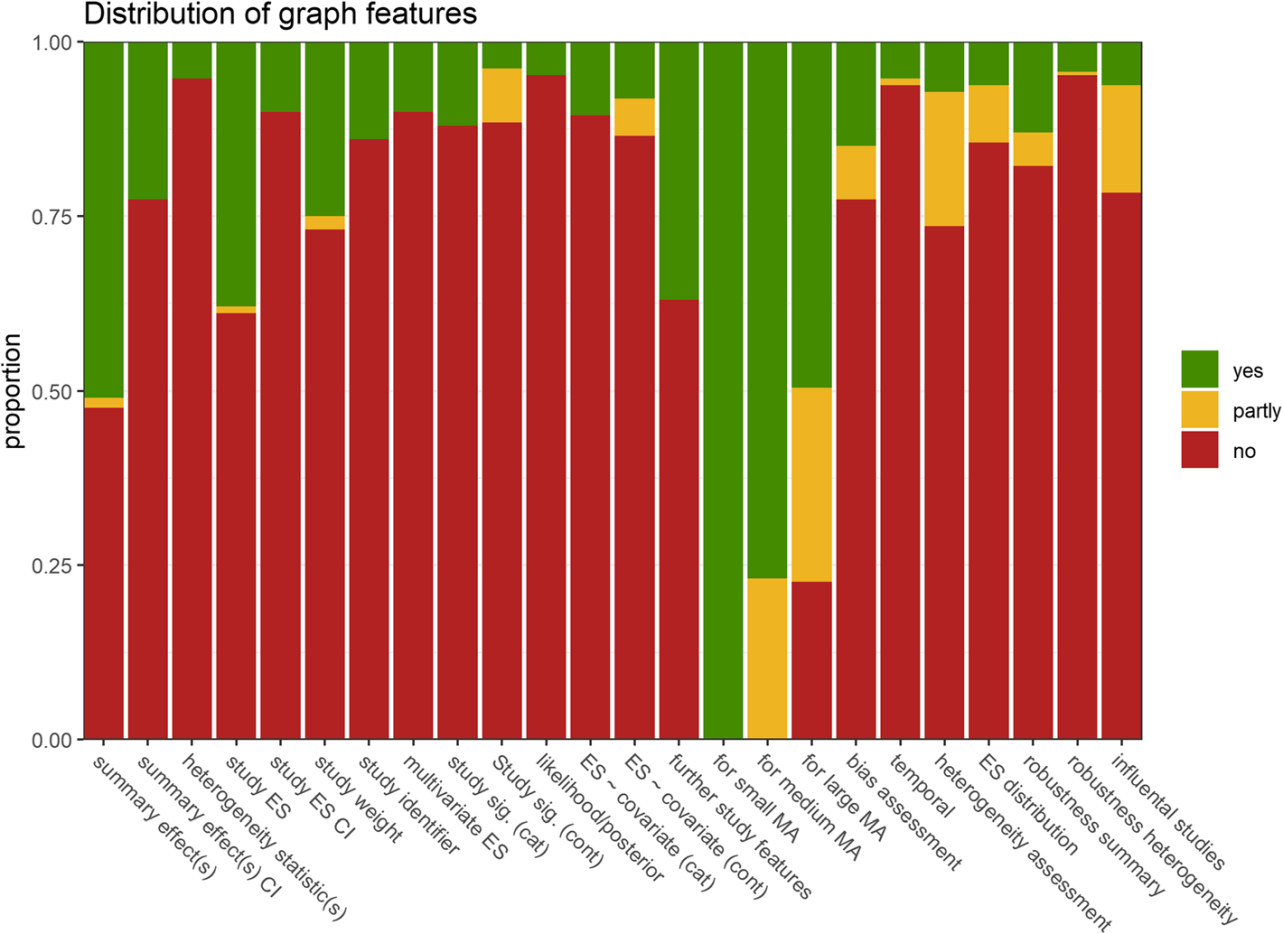
Proportion of meta-analytic graphical displays with a certain functionality feature present

In the following, the inventory of data-visualization methods in meta-analysis is described with respect to the 24 graph-functionality features (Table 2; Fig. 6). Feature assessments for the entire collection of graphs can be found in Additional File 3.

Whereas all graphical displays are suitable to display small-sized meta-analyses (say, 10 studies), 76.9 and 49.5% of graphical displays remain fully suited for medium-sized (say, 50 studies) and large-sized meta-analyses (say hundreds of studies), respectively. The most common further (fully present) functionality features were depiction of summary effect(s) (51.0% of all displays), depiction of individual study effect sizes (38.0%), depiction of further study features (37.0%), and depiction of study weight/sample size/standard error (25.0%).

Features that allow assessing the trustworthiness, sensitivity, and robustness of meta-analytic results were less common: 14.9% of all displays are suitable to assess publication bias and other forms of biases (7.7% partly), 13.0% are suitable to assess the robustness of the summary effect (4.8% partly), 4.3% the robustness of heterogeneity summary effects (0.5% partly), 6.2% are suitable to assess distributional assumptions of effect sizes (8.2% partly), and 6.2% are fully suited to identify influential studies (15.4% are partly suited).

Despite the prevalence of displays which depict study and summary effects, those which also show confidence intervals of effect sizes (10.1%) and confidence intervals of summary effects (22.6%) were less frequent. The likelihood or posterior distribution of meta-analytic parameter estimates was conveyed by 4.8% of all graphs. In addition, while nearly 40% of graphs showed study effect sizes, only 13.9% allowed identifying studies with study identifiers; 10.6% allowed for a categorical classification of study-level significance (i.e., significant vs. not), and 3.8% (7.7% partly) for a continuous classification. Of all displays, 10.1% show more than one effect size per study.

Remarkably, despite heterogeneity being one of the key topics of meta-analysis, only 5.3% of displays visualize summary heterogeneity statistics, and 7.2% displays were suited to assess between-study heterogeneity (19.2% of displays were partly suited). Taken together, this suggests that surprisingly few specialized plots for heterogeneity assessment are available. For the explanation of between-study heterogeneity, 22.1% of all displays allow examining the association of study effect sizes with categorical (10.6%) and continuous (8.2, 5.3% partly) study covariates, while 5.3% depict time trends in meta-analytic estimates (1% partly).

On average, graphs had 5.4 functionality features fully present (*Mdn* = 5, *SD* = 1.7, Min = 2, Max = 11) and 6.6 at least partly present (*Mdn* = 6, *SD* = 2.6, Min = 3, Max = 15). The graphical displays with the most features fully present, and therefore potentially conveying the most information, were a Galbraith plot variant, which additionally showed subgroup information (11 features, 15 at least partly), the subgroup forest plot (10 features, 14 at least partly), and the rainforest plot, a novel forest plot variant (10 features, 14 at least partly).

Of all 208 plots or plot variants in the compilation, 130 (62.5%) possessed a unique combination of graph features. When only fully present features were considered and compared to features partly present or not present combined, still 116 graphs (55.8%) showed a combination of features that no other graph in the compilation possessed. Arguably, this further attests to the heterogeneous, non-redundant, and specialized nature of the landscape of graphs available for meta-analysis.

**Fig. 7:**
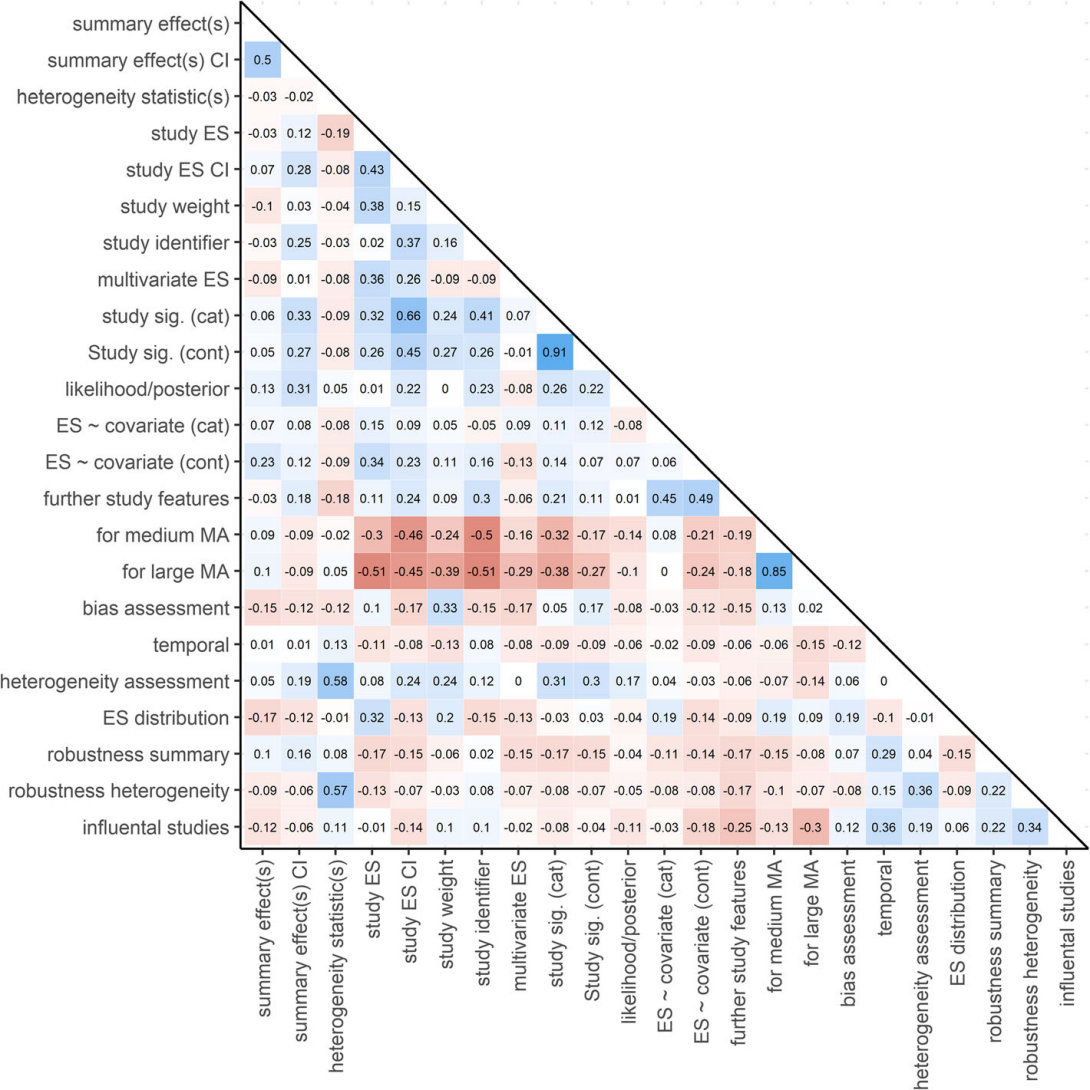
Correlations of the functionality features (coded: 2 = yes, 1 = partly, 0 = no) of meta-analytic graphical displays

Of particular interest is that the presence or absence of functionality features in a specific graph is not random (Fig. 7). Exploring features that often or seldom occur together in the same graph might help identifying potential gaps in the current graph inventory for meta-analysis and may serve as a roadmap for future development of graphical displays for research synthesis.

There is a strong negative association of a graph showing, on the one hand, summary outcome interval estimates, individual study-level effects, study-effect interval estimates, study weights, or study identifiers, and, on the other hand, being suitable for larger or medium-sized meta-analyses. Although naturally hard to combine, displays for medium-sized to large-sized meta-analyses, which still allow identifying each study and its effects, apparently are rare and thus a fruitful avenue for future graph development.

Graphs suitable for the assessment of publication bias or other forms of bias tend to show neither a meta-analytic summary effect nor effect-size confidence intervals, and seldom are suited for showing more than one effect size per study. In addition, displays showing more than one effect size per study (multivariate meta-analysis), influential or outlier studies, and displays suitable for the assessment of distributional assumptions of effect sizes, tend to show no meta-analytic summary outcomes. Moreover, showing some kind of meta-analytic summary estimate (summary effect estimate, heterogeneity summary statistics) is negatively related to displaying any additional study features. The most prevalent combinations of graph features are as expected: graphs showing a summary effect tend to show a confidence interval (or some other form of interval estimator) as well; graphs suitable for medium-sized meta-analyses are often suited for large-sized meta-analysis, too (e.g., by showing only summary, not study-level, estimates); and graphs often allow to depict nominal statistical significance on the study level categorically, as well as continuously at the same time.

## Discussion

We collected, structured, classified, and described the landscape of meta-analytic graphs in unprecedented scope and detail. The introduction of new graphical displays for research synthesis (meta-analysis and systematics reviews) has grown at a remarkable pace: all in all, we collected 208 distinct graphs and graph variations. The availability of such a large number of statistical graphs for meta-analysis may well come as a surprise for many. Previously available general reviews on graphs in meta-analysis covered at most one quarter the size of the present compilation. One driving factor of the graphics explosion in the field of meta-analysis in the mid-2000s has been the continuing development of new displays for network meta-analysis. New plotting options have been added recently, however, for practically any other type of meta-analysis as well. Meta-analytic graphs and their variants possess a rich and diverse set of graph features. Thus, the present graph compilation contains a large number of diverse and specialized displays for numerous aspects of meta-analysis.

However, despite the availability and potential of graphical displays for exploring and communicating meta-analytic results, their usage in published meta-analyses was, and still is, rather limited. In an early review, Light, Singer, and Willet reported that for 74 meta-analyses published in *Psychological Bulletin* between 1985 and 1991, only 19% included graphical displays [7]. This proportion increased to 52% among 60 meta-analyses published in the same journal from 2000 to 2005 [9]. In both these studies, the majority of graphical displays observed were univariate depictions of effect-size distributions (e.g., histograms). Schild and Voracek systematically reviewed graph use in meta-analyses published in top journals in medicine, psychology, and business research over 30 years (1981 to 2011) [22]. Of the total of 993 meta-analyses inspected, only 50% contained any graphical display to communicate their results. The single dominant display was the forest plot; hardly any other graphs were used.

Also, graphical displays are barely covered in existing published guidelines. In APA-issued MARS (Meta-Analysis Reporting Standards) [132], graphical displays are not mentioned at all. In PRIMSA, solely the optional use of forest plots for visualizing individual study results is recommended [74]. Relatedly, given the evidence for a graphics explosion in the domain of meta-analysis since the mid-2000s, it is perhaps ironic to observe that, while the first two editions (1994 and 2009) of a major textbook resource of research synthesis methodology each had included a dedicated chapter on visual displays for meta-analysis [7, 9], the most recent edition thereof (2019) has none such [133].

We observed consistent results when examining graph use in meta-analysis by looking at both implicit and explicit graph coverage in textbooks. In the available textbooks on meta-analytic methodology (Additional File 1), the forest plot and the funnel plot once more were the most often covered displays, and often the only ones.

Hence, despite the diverse and large number of available graphical displays, it seems that only very few of these are regularly applied in scientific practice. Existing reporting guidelines clearly fail to explicitly encourage their use. The existing repertoire of visualization methods is thus likely not used to its full potential in exploring and presenting meta-analytic results.

As to why many graphical displays are not used on a common basis by meta-analysts, we highlight three possible reasons: first, many of the available graphical displays and their uses might be widely unknown. Second, researchers who publish meta-analyses, as well as editors and reviewers, might not see the additional benefits in using graphical displays towards the goal of communicating meta-analytic results optimally. Third, user-friendly software for creating graphical displays might not be readily available. We hope that the comprehensive survey of currently available graphical displays at hand might successfully counter the first two of these inhibitory reasons.

Reviews on software availability for graphing meta-analytic data have been conducted elsewhere ([22, 134]) and are beyond the intended scope of our account. In short, most of the widely used classic meta-analytic software packages primarily allow to create traditional meta-analytic displays, namely, forest plots (CMA [15], Revman [16], Mix 2.0 [17]), funnel plots (CMA [15], Revman [16], Mix 2.0 [17]), radial plots (Mix 2.0 [17]), L’Abbé plots (Mix 2.0 [17]), and meta-regression plots (CMA [15], Mix 2.0 [17]). Many of the more recently proposed and potentially less known graphs can only be created using syntax-based statistical software and software packages (e.g., R [20] or Stata [18]). User-friendly statistical software solutions for a large number of the graphs and graph variants described here currently are unavailable.

The primary aim of our account is to give an overview of available graph options for meta-analysis. However, because of the large number of graphs found, it was not feasible to discuss each and every display in more detail other than in the form of a vignette (Additional File 2). For a more elaborated and focused discussion, as well as for suggestions on the use of the most widely known displays for univariate meta-analysis (namely, the forest, funnel, L’Abbé, and Galbraith plots), we recommend to refer to [11]. Likewise, for a focused treatment of a number of graphical displays for network meta-analysis, we refer to [13].

Although much thought and iterative effort was put into the derivation of a useful taxonomy, our choice is only one of many imaginable ones, and thus the membership of a plot to a certain category in this taxonomy should not be overstated. For the description of plots, we used a bottom-up derived list of graph features evaluated by two expert raters (Additional File 3). These ratings should be taken as a crude guide as to which plot in principle conveys which statistical information. The ratings are not intended to compete with, or replace, original empirical research on the visual perception of specific statistical information from different meta-analytic graphs (e.g., [10]; for forest plot variants: [23]).

Data visualization in meta-analysis is a field of long tradition and swiftly ongoing development. Typical feature spaces of currently available graphs still show gaps and thus leave ample room for novel visualization methods. Two examples for such gaps identified here are, firstly, graphs allowing to depict more than two effect sizes per study (or, more generally, per level in multilevel meta-analysis), and secondly, suitable displays for medium-sized to large-sized meta-analyses, which nevertheless allow to depict study-level effects and study identifiers. Therefore, despite the large number of already available graphs, in all likelihood the trend of new developments will continue in the foreseeable future, in tandem with advancements in meta-analytic methodology.

There arguably are a number of potentially useful, but currently underused, or at least underreported, graphs. One area of such underreported graphs are most likely diagnostic graphs, which assess the robustness and sensitivity of meta-analytic results to study inclusions and common methodological decisions (e.g., fixed-effect vs. random-effects model). Given the possibility of providing additional supplemental files online, there remain few, if any, reasons on the side of article authors for not providing more such diagnostic plots, in order to beneficially increase the transparency of their meta-analytic reporting [135].

## Conclusion

The present overview took stock of a total of 208 retrievable distinct graphical displays, which so far have been proposed and used for exploring and communicating meta-analytic results. We hope this resource will contribute to utilizing the available tool kit of data-visualization methods in meta-analysis to its full potential and enable researchers to make better-informed decisions on which graphs to consider for presenting their meta-analytic data. Likewise, the current overview may well constitute a roadmap for goal-driven development of further graphical displays for research synthesis.

## Supplementary information

**Additional file 1.** Chronological bibliography of textbooks, monographs, and software manuals on meta-analysis and systematic reviews.

**Additional file 2.** Graph vignettes for the entire collection of 208 graphical displays for meta-analysis and systematic reviews.

**Additional file 3.** Feature assessments for the entire collection of 208 graphical displays for meta-analysis and systematic reviews. 

## Data Availability

All data generated and analysed during this study either are included in this article and its supplementary information files and/or are available at the Open Science Framework repository, https://osf.io/gkpe5/.

## Abbreviations

CMA: Comprehensive Meta-Analysis
CRAN: Comprehensive R Archive Network
CUMSUM: Cumulative Sum
GOSH: Graphical Display of Study Heterogeneity
MARS: Meta-Analysis Reporting Standards
PRISMA: Preferred Reporting Items for Systematic Reviews and Meta-Analyses
ROC: Receiver Operating Characteristic
