# Supplementary material for: Charting the landscape of graphical displays for meta-analysis and systematic reviews: a comprehensive review, taxonomy, and feature analysis
Source: BMC Med Res Methodol. 2020 Feb 7;20:26. doi: 10.1186/s12874-020-0911-9 (PMC7006175; doi:10.1186/s12874-020-0911-9)
Supplement: Supplementary file 1 — Additional file 1. Chronological bibliography of textbooks, monographs, and software manuals on meta-analysis and systematic reviews. [file 12874_2020_911_MOESM1_ESM.doc]

**Additional File 1 – A chronological bibliography of textbooks, monographs, and software manuals**

**on research synthesis methodology (meta-analysis and systematic reviews)**

This bibliography of research synthesis methodology textbooks, starting with the very first (applied) meta-analytic monograph (Glass et al., 1980) and spanning almost four decades, is, to our knowledge, the most comprehensive one of its kind (see main text, Section 2.2., for the evidence search strategies employed for compiling it). It is more up-to-date (up to late 2018), and has more entries, than similar book lists currently accessible online (see, for instance, <http://www.um.es/metaanalysis/texts.php>). For this reason, apart from documenting a main information source for our investigation, this list of monographs constitutes a bibliographic resource of its own, for future use; in particular, for research on the scholarly history of meta-analysis and the development and dissemination of meta-analytic methodology over time.

The entire text corpus of the 153 monographs listed here comprises about 38000 book pages. A considerable number of monographs (29 out of 153, or 19%) is in languages other than English (7 books each in German and Spanish, 5 in Italian, 4 in Swedish, 3 in French, 2 in Dutch, and 1 in Danish).

With the single exception of Aguilar (2001), a 67-pages, Spanish-language, textbook from Chile, which proved inaccessible via both interlibrary loan services and online book markets, we were able to acquire all of these and inspected all of them cover to cover in the course of our investigation (see main text for procedural details).

One side benefit of our literature searches and continued attempts to acquire all of these textbooks was that the state of facts concerning a few alleged, or mystifying, publications could be clarified and some bibliographic errors resolved. To begin with, at the URL provided above, there is a reference to a book with the title “Clinical meta-analysis“, edited by George Davey Smith (2001, New York, Wiley). Entries to this book are also found in various online databases (e.g., at Amazon.com and WorldCat.org); however, mystifyingly, there neither are copies for selling, nor library holdings. The solution to this is that the book was announced (and thus already had left traces online), but in the end did not materialize and was not published (personal communication G. Davey Smith to M. Voracek, Dec 17, 2010). A similar case appears to be the Spanish-language title “Meta-analysis en medicine“ (2013). It can be found at Amazon.es, but is available nowhere. Finally, a 2nd edition of Wolf (1986) was announced for 2013, according to Amazon.com. This title has also not been published up to now.

1980

1. Smith, M. L., Glass, G. V., & Miller, T. I. (1980). *The benefits of psychotherapy*. Baltimore: Johns Hopkins University Press.

1981

1. Glass, G. V., McGraw, B., & Smith, M. L. (1981). *Meta-analysis in social research.* Beverly Hills, CA: Sage.

1982

1. Hunter, J. E., Schmidt, F. L., & Jackson, G. B. (1982). *Meta-analysis: Cumulating research findings across studies.* Beverly Hills, CA: Sage.

1983

1. Treinies, G., & Fricke, R. (1983). *Deskriptive Methoden der Metaanalyse zur Integration experimenteller Studien dargestellt an einem Beispiel aus der Instruktionspsychologie* [in German]*.* Hannover: Arbeitsstelle für Unterrichtsforschung (Bericht Nr. 5).

1984

1. Cooper, H. (1984). *The integrative research review: A systematic approach.* Beverly Hills, CA: Sage.
2. Light, R. J., & Pillemer, D. B. (1984). *Summing up: The science of reviewing research.* Cambridge, MA: Harvard University Press.
3. Rosenthal, R. (1984). *Meta-analytic procedures for social research*. Beverly Hills, CA: Sage.

1985

1. Berglund, G. W. (1985). *Meta-analys: Om syntes av kvantativa forskningsresultat* [in Swedish]. *Uppsala:* Pedagogiska Institutionen, Uppsala Universitet.
2. Hedges, L. V., & Olkin, I. (1985). *Statistical methods for meta-analysis.* San Diego, CA: Academic Press.
3. Mullen, B., & Rosenthal, R. (1985). *BASIC meta-analysis: Procedures and programs.* Hillsdale, NJ: Erlbaum.
4. Fricke, R., & Treinies, G. (1985). *Einführung in die Metaanalyse* [in German]*.* Bern: Huber.

1986

1. Farley, J. U., & Lehmann, D. R. (1986). *Meta-analysis in marketing: Generalization of response models.* Lexington, MA: Lexington Books.
2. Hyde, J. S., & Linn, M. C. (1986). *The psychology of gender: Advances through meta-analysis.* Baltimore, MD: Johns Hopkins University Press.
3. Wolf, F. M. (1986). *Meta-analysis: Quantitative methods for research synthesis.* Beverly Hills, CA: Sage.

1987

1. Gómez, J. (1987). *Meta-análisis* [in Spanish]. Barcelona: PPU.
2. Jenicek, M. (1987). *Méta-analyse en médecine: Évaluation et synthèse de l’information clinique et épidémiologique* [in French]. St.Hyacinthe (Québec)/Paris: EDISEM/Maloine Éditeurs.
3. Rosenthal, R. (1987). *Judgment studies: Design, analysis, and meta-analysis.* Cambridge, MA: Cambridge University Press.

1988

1. Smith, M. C. (1988). *Meta-analysis of nursing intervention research.* Birmingham, AL: Author.

1989

1. Cooper, H. M. (1989). *Integrating research: A guide for literature reviews* (2nd ed.). Newbury Park, CA: Sage.
2. Hedges, L. V., Shymansky, J. A., & Woodworth, G. (1989). *A practical guide to modern methods of meta-analysis.* Washington, DC: National Science Teachers Association.
3. Johnson, B. T. (1989). *DSTAT: Software for the meta-analytic review of research literatures.* Hillsdale, NJ: Erlbaum.
4. Mullen, B. (1989). *Advanced BASIC meta-analysis.* Hillsdale, NJ: Erlbaum.

1990

1. Drinkmann, A. (1990). *Methodenkritische Untersuchungen zur Metaanalyse* [in German]*.* Weinheim: Deutscher Studien-Verlag.
2. Hunter, J. L., & Schmidt, F. L. (1990). *Methods of meta-analysis: Correcting error and bias in research findings.* Newbury Park, CA: Sage.
3. Saris, W. E., & van Meurs, A. (Eds.). (1990). *Evaluation of measurement instruments by meta-analysis of multitrait multimethod studies*. Amsterdam: Royal Netherlands Academy of Arts and Sciences.
4. Wachter, K. W., & Straf, M. L. (Eds.) (1990). *The future of meta-analysis.* New York: Russell Sage.

1991

1. Rosenthal, R. (1991). *Meta-analytic procedures for social research* (rev. ed.). Newbury Park, CA: Sage.

1992

1. Cook, T. D., Cooper, H., Cordray, D. S., Hartmann, H., Hedges, L. V., Light, R. J., Louis, T. A., & Mosteller, F. (1992). *Meta-analysis for explanation: A casebook.* New York: Russell Sage.
2. Eddy, D. M., Hasselblad, V., & Shachter, R. (1992). *Meta-analysis by the confidence profile method: The statistical synthesis of evidence.* Boston: Academic Press.
3. National Research Council – Panel on Statistical Issues and Opportunities for Research in the Combination of Information [Draper, G., Gaver, D. P., Goel, P. K., Greenhouse, J. B., Hedges, L. V., Morris, C. N., Tucker, J., & Waternaux, C.] (1992). *Combining information: Statistical issues and opportunities for research.* Washington, DC: National Academy Press.
4. Plath, I. (1992). *Unterstanding meta-analyses: A consumer’s guide to aims, problems, evaluation and developments.* Baden-Baden: Nomos Verlagsgesellschaft.

1993

1. Johnson, B. T. (1993). *DSTAT 1.10: Software for the meta-analytic review of research literatures: Upgrade documentation.* Hillsdale, NJ: Erlbaum.

1994

1. Cooper, H., & Hedges, L. V. (Eds.) (1994). *The handbook of research synthesis*. New York: Russell Sage.
2. Farin, E. (1994). *Forschungsperspektive und Methodik der Metaanalyse* [in German]. Forschungsberichte des Psychologischen Instituts der Albert-Ludwigs-Universität Freiburg i. B. Nr. 113.
3. Petitti, D. B. (1994). *Meta-analysis, decision analysis, and cost-effectiveness analysis: Methods for quantitative synthesis in medicine.* New York: Oxford University Press.

1995

1. Chalmers, I., & Altman, D. G. (Eds.). (1995). *Systematic reviews.* London: BMJ Publishing Group.
2. Di Nuovo, S. (1995). *La meta-analisi: Fondamenti teorici e applicazioni nella ricerca psicologica* [in Italian]*.* Rome, Italy: Edizioni Borla.
3. van Houwelingen, J. C. (Ed.). (1995). *Meta-analyse* [in Dutch]. Leiden: Boerhaave Commissie voor Postacademisch Onderwijs in de Geneeskunde, Rijksuniversiteit Leiden.

1996

1. de Hollander, A. E. M., Preller, E. A., Heisterkamp, S. H., & Jansen, J. (1996). *Meta-analyse van observationeel onderzoek: Mogelijkheden en beperkingen bij toepassingen ten behoeve van het kwantificeren van gezondheidsrisico’s* [in Dutch]. Bilthoven (The Netherlands): Rijksinstituut voor Volksgezondheid en Milieu.

1997

1. Cucherat, M. (1997). *Méta-analyse des essais thérapeutiques* [in French]. Paris: Masson.
2. Hunt, M. (1997). *How science takes stock: The story of meta-analysis.* New York: Russell Sage.
3. Rosenberg, M. S., Adams, D. C., & Gurevitch, J. (1997). *MetaWin: Statistical software for meta-analysis with resampling tests.* Sunderland, MA: Sinauer.
4. van den Bergh, J. C. J. M., Button, K. J., Nijkamp, P., & Pepping, G. C. (1997). *Meta-analysis in environmental economics.* Dordrecht (The Netherlands): Kluwer.

1998

1. Cooper, H. (1998). *Synthesizing research: A guide for literature reviews* (3rd ed.). Thousand Oaks, CA: Sage.
2. Cucherat, M., & Haugh, M. (1998). *EasyMA 98b: Reference manual*. Department of Clinical Pharmacology, University of Lyon, France.
3. Mulrow, C., & Cook, D. (Eds.). (1998). *Systematic reviews: Synthesis of best evidence for health care decisions.* Philadelphia: American College of Physicians.
4. Sutton, A. J., Abrams, K. R., Jones, D. R., Sheldon, T. A., & Song, F. (1998). Systematic reviews of trials and other studies. *Health Technology Assessment, 2(19),* 1-276.

1999

1. Böhning, D. (1999). *Computer-assisted analysis of mixtures and applications: Meta-analysis, disease mapping and others.* Boca Raton, FL: Chapman & Hall/CRC.
2. Shadish, W. R., Robinson, L., & Lu, C. (1999). *ES: A computer program for effect sizes calculation.* St. Paul, MN: Assessment Systems Corporation.
3. Wang, M. C., & Bushman, B. J. (1999). *Integrating results through meta-analytic review using SAS software.* Cary, NC: SAS Institute.

2000

1. Borenstein, M., Hedges, L. V., Higgins, J. P. T., & Rothstein, H. R. (2000). *Comprehensive Meta Analysis version 1 manual.* Englewood, NJ: Biostat.
2. Chevarier, P., Cucherat, M., Freiburger, T., Maupas, J., Visele, N., Bugnard, F., & Bazog, P. (2000). *WeasyMA*. Lyon: ClinInfo.
3. Dobrilla, G., & Capurso, L. (2000). *Meta-analisi in gastroenterologia: Incertezze, apporto conoscitivo, aiuto decisionale* [in Italian]*.* Roma: Il pensiero scientifico.
4. Petitti, D. B. (2000). *Meta-analysis, decision analysis, and cost-effectiveness analysis: Methods for quantitative synthesis in medicine* (2nd ed.). New York: Oxford University Press.
5. Rosenberg, M. S., Adams, D. C., & Gurevitch, J. (2000). *MetaWin: Statistical software for meta-analysis version 2*. Sunderland, MA: Sinauer.
6. Song, F., Eastwood, A. J., Gilbody, S., Duley, L., & Sutton, A. J. (2000). Publication and related biases. *Health Technology Assessment, 4(10),* 1-115.
7. Stangl, D. K., & Berry, D. A. (2000). *Meta-analysis in medicine and health policy.* New York: Dekker.
8. Sutton, A. J., Abrams, K. R., Jones, D. R., Sheldon, T. A., & Song, F. (2000). *Methods for meta-analysis in medical research.* Chichester, West Sussex: Wiley.

2001

1. Aguilar, P. B. (2001). *Plan de análisis cuantitativo, cualitativo y meta-análisis en investigación psicológica* [in Spanish]. Santiago de Chile: Eds. Univ. Diego Portales.
2. Arthur, W., Bennett, W., & Huffcutt, A. I. (2001). *Conducting meta-analysis using SAS.* Mahwah, NJ: Erlbaum.
3. Egger, M., Smith, G. D., & Altman, D. G. (Eds.) (2001). *Systematic reviews in health care: Meta-analysis in context* (2nd ed.). London: BMJ Books.
4. Glasziou, P., Irwig, L., Bain, C., & Colditz, G. (2001). *Systematic reviews in health care: A practical guide.* Cambridge: Cambridge University Press.
5. Khan, K. S., ter Riet, G., Glanville, J., Sowden, A. J., & Kleijnen, J. (2001). *Undertaking systematic reviews of research on effectiveness: CRD’s guidance for those carrying out or commissioning reviews.* University of York: NHS Centre for Reviews and Dissemination.
6. Leandro, G. (2001). *Guida alla meta-analisi: Manuale pratico ed operativo* [in Italian]. Roma: Il pensiero scientifico.
7. Lipsey, M. W., & Wilson, D. B. (2001). *Practical meta-analysis.* Thousand Oaks, CA: Sage.
8. Molina, L. E., & Marino, A. P. (2001). *Introducción al análisis de datos en meta-análisis* [in Spanish]. Madrid: Ediciones Díaz de Santos.
9. Paterson, B. L., Thorne, S. E., Canam, C., & Jillings, C. (2001). *Meta-study of qualitative health research: A practical guide to meta-analysis and meta-synthesis.* Thousand Oaks, CA: Sage.

2002

1. Botella, J., & Gambara, H. (2002). *Qué es el meta-análisis* [in Spanish]. Madrid: Biblioteca Nueva.
2. Rudner, L., Glass, G. V., Evartt, D. L., Emery, P. J. (2002). *A user’s guide to the meta-analysis of research studies*. ERIC Clearinghouse on Assessment and Evaluation, University of Maryland, College Park. [<http://echo.edres.org:8080/meta/metaman.htm>]
3. Whitehead, A. (2002). *Meta-analysis of controlled clinical trials.* Chichester, West Sussex: Wiley.

2003

1. Andersson, G. (2003). *Metaanalys: Metoder, tillämpningar och kontroverser* [in Swedish]. Lund: Studentlitteratur.
2. Forsberg, C., & Wengstrom, Y. (2003). *Att göra systematiska litteraturstudier: Värdering, analys och presentation av omvårdnadsforskning* [in Swedish]. Stockholm: Natur & Kultur.
3. Khan, K. S., Kunz, R., Kleijnen, J., & Antes, G. (2003). *Systematic reviews to support evidence-based medicine: How to review and apply findings of healthcare research.* London: Royal Society of Medicine Press.
4. Murphy, K. R. (Ed.). (2003). *Validity generalization: A critical review.* Mahwah, NJ: Erlbaum.
5. Rustenbach, S. J. (2003). *Metaanalyse: Eine anwendungsorientierte Einführung* [in German]*.* Bern: Huber.
6. Schulze, R., Holling, H., & Böhning, D. (2003). *Meta-analysis: New developments and applications in medical and social sciences.* Cambridge, MA: Hogrefe & Huber.
7. Torgerson, C. (2003). *Systematic reviews.* London: Continuum.

2004

1. Hunter, J. E., & Schmidt, F. L. (2004). *Methods of meta-analysis: Correcting error and bias in research findings* (2nd ed.). Thousand Oaks, CA: Sage.
2. Khan, K., S., Kunz, R., Kleijnen, J., & Antes, G. (2004). *Systematische Übersichten und Meta-Analysen: Ein Handbuch für Ärzte in Klinik und Praxis sowie Experten im Gesundheitswesen* [in German]*.* Berlin: Springer.
3. Schulze, R. (2004). *Meta-analysis: A comparison of approaches.* Cambridge, MA: Hogrefe & Huber.

2005

1. Borenstein, M., Hedges, L. V., Higgins, J. P. T., & Rothstein, H. R. (2005). *Comprehensive Meta Analysis version 2 manual.* Englewood, NJ: Biostat.
2. Glenny, A. M., Altman, D. G., Song, F., Sakarovitch, C., Deeks, J. J., D’Amico, R., Bradburn, M., & Eastwood, A. J. (2005). Indirect comparisons of competing interventions*.* *Health Technology Assessment, 9(26)*, 1-148.
3. Grissom, R. J., & Kim, J. J. (2005). *Effect sizes for research: A broad practical approach.* Mahwah, NJ: Erlbaum.
4. Leandro, G., & Gallus, G. (2005). *Meta-analysis in medical research: The handbook for the understanding and practice of meta-analysis.* Malden, MA: BMJ Books/Blackwell.
5. Roberts, C. J., & Stanley, T. D. (Eds.). (2005). *Meta-regression analysis: Issues of publication bias in economics.* Malden, MA: Blackwell.
6. Rothstein, H. R., Sutton, A. J., & Borenstein, M. (Eds.) (2005). *Publication bias in meta-analysis: Prevention, assessment and adjustments.* Chichester, West Sussex: Wiley.

2006

1. Martín, J. L. R., Tobías, A., & Seoane, T. (Eds.) (2006*). Revisiones sistemáticas en las ciencias de la vida* [in Spanish]. Toledo: FISCAM.
2. Petticrew, M., & Roberts, H. (2006). *Systematic reviews in the social sciences: A practical guide.* Malden, MA: Blackwell.

2007

1. Borenstein, M., Hedges, L. V., & Rothstein, H. R. (2007). *Meta-analysis: Fixed effect vs. random effects.* Englewood, NJ: Biostat.
2. Sandelowski, M., & Barroso, J. (2007). *Handbook for synthesizing qualitative research.* New York: Springer.
3. Webb, C., & Roe, B. H. (Eds.). (2007). *Reviewing research evidence for nursing practice: Systematic reviews.* Oxford: Wiley.

2008

1. Böhning, D., Kuhnert, R., & Rattanasiri, S. (2008). *Meta-analysis of binary data using profile likelihood.* Boca Raton, FL: Chapman & Hall/CRC.
2. Hartung, J., Knapp, G., & Sinha, B. K. (2008). *Statistical meta-analysis with applications.* Hoboken, NJ: Wiley.
3. Higgins, J. P. T., & Green, S. (Eds.). (2008). *Cochrane handbook for systematic reviews of interventions.* Chichester, West Sussex: Wiley.
4. Kulinskaya, E., Morgenthaler, S., & Staudte, R. G. (2008). *Meta analysis: A guide to calibrating and combining statistical evidence.* Chichester, West Sussex: Wiley.
5. Littell, J. H., Corcoran, J., & Pillai, V. (2008). *Systematic reviews and meta-analysis.* New York: Oxford University Press.
6. McQuay, J. J., Kalso, E., & Moore, R. A. (Eds.). (2008). *Systematic reviews in pain research: Methodology refined.* Seattle: IASP Press.

2009

1. Borenstein, M., Hedges, L. V., Higgins, J. P. T., & Rothstein, H. R. (2009). *Introduction to meta-analysis.* Chichester, West Sussex: Wiley.
2. Centre for Reviews and Dissemination (2009). *Systematic Reviews: CRD’s guidance for undertaking reviews in healthcare.* University of York NHS Centre for Reviews & Dissemination.
3. Cooper, H., Hedges, L. V., & Valentine, J. C. (Eds.) (2009). *The handbook of research synthesis and meta-analysis* (2nd ed.). New York: Russell Sage.
4. Kunz, R., Khan, K. S., Kleijnen, J., & Antes, G. (2009). *Systematische Übersichtsarbeiten und Meta-Analysen: Einführung in Instrumente der evidenzbasierten Medizin für Ärzte, klinische Forscher und Experten im Gesundheitswesen* (2., vollst. überarb. Aufl.) [in German]. Bern: Huber.
5. Sterne, J. A. C. (Ed.). (2009). *Meta-analysis in Stata: An updated collection from the Stata Journal.* College Station, TX: Stata Press.

2010

1. Cooper, H. (2010). *Research synthesis and meta-analysis: A step-by-step approach* (4th ed.). Los Angeles: Sage.
2. Ellis, P. D. (2010). *The essential guide to effect sizes: Statistical power, meta-analysis, and the interpretation of research results*. Cambridge: Cambridge University Press.
3. Froehling, H. (2010). *Meta analysis: A critical introduction of its historical origins.* Fort Lauderdale, FL: CreateSpace.
4. Guerra, R., & Goldstein, D. R. (Eds.) (2010). *Meta-analysis and combining information in genetics and genomics.* Boca Raton, FL: CRC Press.
5. Huedo-Medina, T. B., & Johnson, B. T. (2010). *Modelos estadísticos en meta-análisis* [in Spanish]*.* Oleiros, La Coruña (Spain): Netbiblo S. L.
6. Song, F., Parekh, S., Hooper, L., Loke, Y. K., Ryder, J., Sutton, A. J., Hing, D., Kwok, C. S., Pang, C., & Harvey, I. (2010). Dissemination and publication of research findings: An updated review of related biases. *Health Technology Assessment, 14(8),* 1-220.

2011

1. Athanasiou, T. & Darzi, A. (Eds.). (2011). *Evidence synthesis in healthcare: A practical handbook for clinicians*. London: Springer.
2. Card, N. A. (2011). *Applied meta-analysis for social science research.* New York: Guilford.
3. Eden, J., Levit, L. A., Berg, A., & Morton, S. (2011). *Finding what works in health care: Standards for systematic reviews.* Washington, DC: National Academies Press.
4. Jesson, J. K. (2011). *Doing your literature review: Traditional and systematic techniques.* London: Sage.
5. Khan, K., Kunz, R., Kleijnen, J., & Antes, G. (2011). *Systematic reviews to support evidence-based medicine: How to review and apply findings of healthcare research* (2nd ed.)*.* London: Hodder Arnold.

2012

1. Bettany-Saltikov, J. (2012). *How to do a systematic literature review in nursing: A step-by-step guide.* Maidenhead, UK: Open University Press.
2. Booth, A., Papaioannou, D., & Sutton, A. (2012). *Systematic approaches to a successful literature review.* Thousand Oaks, CA: Sage.
3. Bronson, D. E., & Davis, T. S. (2012). *Finding and evaluating evidence: Systematic reviews and evidence-based practice.* New York: Oxford University Press.
4. Cumming, G. (2012). *Understanding the new statistics: Effect sizes, confidence intervals, and meta-analysis.* New York: Routledge.
5. Gough, D., Oliver, S., & Thomas, J. (Eds.) (2012). *An introduction to systematic reviews*. Los Angeles: Sage.
6. Grissom, R. J., & Kim, J. J. (2012). *Effect sizes for research: Univariate and multivariate applications* (2nd ed.). New York: Routledge.
7. Holly, C., Salmond, S. W., & Saimbert, M. K. (Eds.). (2012). *Comprehensive systematic review for advanced nursing practice.* New York: Springer.
8. Makambi, K. (2012). *Alternative methods for meta-analysis: For application in the biomedical sciences.* Saarbrücken: Lap Lambert Academic Publishing.
9. Pigott, T. D. (2012). *Advances in meta-analysis.* New York: Springer.
10. Stanley, T. D., & Doucouliagos, H. (2012). *Meta-regression analysis in economics and business.* London: Routledge.
11. Welton, N. J., Sutton, A. J., Cooper, N. J., Abrams, K. R., & Ades, A. (2012). *Evidence synthesis for decision making in healthcare.* Chichester, UK: Wiley.
12. Tufanaru, C., Huang, W., Tsay, S. F., & Chou, S. S. (2012). Statistics for systematic review authors. Philadelphia: Lippincott Williams & Wilkins.
13. Uttal, W. R. (2012). *Reliability in cognitive neuroscience: A meta-meta-analysis.* Cambridge, MA: MIT Press.

2013

1. Chen, D.-G., & Peace, K. E. (2013). *Applied meta-analysis with R*. Boca Raton, FL: Chapman & Hall/CRC.
2. Koricheva, J., Gurevitch, J., & Mengersen, K. (Eds.). (2013). *Handbook of meta-analysis in ecology and evolution.* Princeton, NJ: Princeton University Press.
3. Ringquist, E. (Ed.). (2013). *Meta-analysis for public management and policy*. San Francisco, CA: Jossey-Bass.

2014

1. Biondi-Zoccai, G. (Ed.). (2014). *Network meta-analysis: Evidence synthesis with mixed treatment comparison*. Hauppauge, NY: Nova Science.
2. Boland, A., Cherry, M. G., & Dickson, R. (Eds.). (2014). *Doing a systematic review: A student’s guide.* Los Angeles (CA): Sage.
3. Nelson, H. D. (2014). *Systematic reviews to answer health care questions.* Philadelphia: Wolters Kluwer Health/Lippincott Williams & Wilkins.
4. Schmidt, F. L., & Hunter, J. E. (2014). *Methods of meta-analysis: Correcting error and bias in research findings* (3rd ed.). Thousand Oaks, CA: Sage.
5. Tramacere, I. (2014). *Meta-analisi per outcomes multipli: Un'estensione delle tecniche di meta-analisi per outcomes multipli* [in Italian]. Saarbrücken: Edizioni Accademiche Italiane.

2015

1. Botella Ausina, J., & Sánchez-Meca, J. (2015). *Meta-análisis en ciencias sociales y de la salud* [in Spanish]. Madrid: Síntesis.
2. Cheung, M. W.-L. (2015). *Meta-analysis: A structural equation modeling approach.* New York: Wiley.
3. Crocetti, E. (2015). *Rassegne sistematiche, sintesi della ricerca e meta-analisi* [in Italian]. North Charleston, SC: CreateSpace.
4. Hesser, H., & Andersson, G. (2015). *Introduktion till metaanalys och systematiska översikter* [in Swedish]. Lund: Studentlitteratur.
5. Jak, S. (2015). *Meta-analytic structural equation modelling*. Cham (Switzerland): Springer.
6. Laroche, P. (Ed.). (2015). *La méta-analyse: Méthodes et applications en sciences sociales* [in French]. Louvain-la-Neuve: De Boeck.
7. Schwarzer, G., Carpenter, J. R., & Rücker, G. (2015). *Meta-analysis with R*. Cham (Switzerland): Springer.

2016

1. Council for International Organizations of Medical Sciences (CIOMS) (Ed.) (2016)*. Evidence synthesis and meta-analysis for drug safety: Report of CIOMS Working Group X*. Geneva: CIOMS.
2. Holly, C., Salmond, S., & Saimbert, M. (Eds.). (2016). *Comprehensive systematic review for advanced practice nursing* (2nd ed.). New York: Springer.
3. Kitchenham, B. A., Budgen, D., & Brereton, P. (2016). *Evidence-based software engineering and systematic reviews.* Boca Raton (FL): Chapman & Hall/CRC.
4. Palmer, T. M., & Sterne, J. A. C. (Eds.). (2016). *Meta-analysis in Stata: An updated collection from the Stata journal* (2nd ed.). College Station, TX: Stata Press.
5. Rosenberg, J., Andresen, K., & Burcharth, J. (2016). *Systematisk review og meta-analyse* [in Danish]. Fort Lauderdale, FL: CreateSpace.

2017

1. Abo-Zaid, G. (2017). *Meta analysis of prognostic factor studies using individual data: A one-step or two-step approach.* Beau Bassin (Mauritius): Scholar’s Press.
2. Cleophas, T. J., & Zwinderman, A. H. (2017). *Modern meta-analysis: Review and update of methodologies.* Cham (Switzerland): Springer.
3. Cooper, H. (2017). *Research synthesis and meta-analysis: A step-by-step approach* (5th ed.). Los Angeles: Sage.
4. Cumming, G., & Calin-Jageman, R. (2017). *Introduction to the new statistics: Estimation, open science, and beyond.* New York: Routledge.
5. Hanji, M. B. (2017). *Meta-analysis in psychiatry research: Fundamental and advanced methods*. Toronto: Apple Academic Press.
6. Paracha, U. Z., Paracha, R. Z., & Paracha, S. Z. (2017). *Basics of meta-analysis with basic steps in R*. Rawalpindi & Islamabad (Pakistan): Amazon eBook.

2018

1. Dias, S., Ades, A. E., Welton, N. J., Jansen, J. P., & Sutton, A. J. (2018). *Network meta-analysis for decision-making.* Chichester, UK: Wiley-Blackwell.

**Addendum**

After study completion, and upon finalizing this paper in December 2019, we took the opportunity to rerun all literature search strategies (as detailed in the main text) for retrieving meta-analytic or systematic review textbooks. This yielded 6 further titles which, in addition, for the sake of up-to-dateness and completeness of this bibliographic resource for researchers and practitioners, are listed here below.

Biondi-Zoccai, G. (Ed.) (2018). *Diagnostic meta-analysis: A useful tool for clinical decision-making.* Cham (Switzerland): Springer.

Borenstein, M. (2019). *Common mistakes in meta-analysis and how to avoid them.* Englewood, NJ: Biostat.

Chun-Pei, C. (2016). *Genome-wide meta-analysis of biomarkers and genetic variations*. Beau Bassin (Mauritius): Golden Light Academic Publishing.

Cooper, H., Hedges, L. V., & Valentine, J. C. (Eds.) (2019). *The handbook of research synthesis and meta-analysis* (3rd ed.). New York: Russell Sage.

Leandro, G. (2018). *Meta analysis: The handbook for learning, understanding and practice of meta analysis in medical research* (2nd ed.). [n.p.]: The Bridge Publishing.

Makowski, D., Piraux, F., & Brun, F. (2019). *From experimental network to meta-analysis: Methods and applications with R for agronomic and environmental sciences.* Dordrecht (The Netherlands): Springer.
