# Supplementary material for: Charting the landscape of graphical displays for meta-analysis and systematic reviews: a comprehensive review, taxonomy, and feature analysis
Source: BMC Med Res Methodol. 2020 Feb 7;20:26. doi: 10.1186/s12874-020-0911-9 (PMC7006175; doi:10.1186/s12874-020-0911-9)
Supplement: Supplementary file 2 — Additional file 2. Graph vignettes for the entire collection of 208 graphical displays for meta-analysis and systematic reviews. [file 12874_2020_911_MOESM2_ESM.docx]

**Additional File 2 – Taxonomy and description of 208 graphical displays for meta-analysis and systematic reviews**

*Prefatory note*.––In this additional file, the entirety of the 208 graphical displays and display variants for meta-analytic data retrieved are shown and described in graph vignettes. If not specifically stated otherwise in these graph vignettes, all graphs were manually created by the authors within the statistical programming language R (R Core Team, 2018) and by drawing on simulated example data for the illustrative purposes intended by this graph collection.

| Confidence interval plot, caterpillar plot [1.1] | Forest plot [1.2] |
| --- | --- |
| 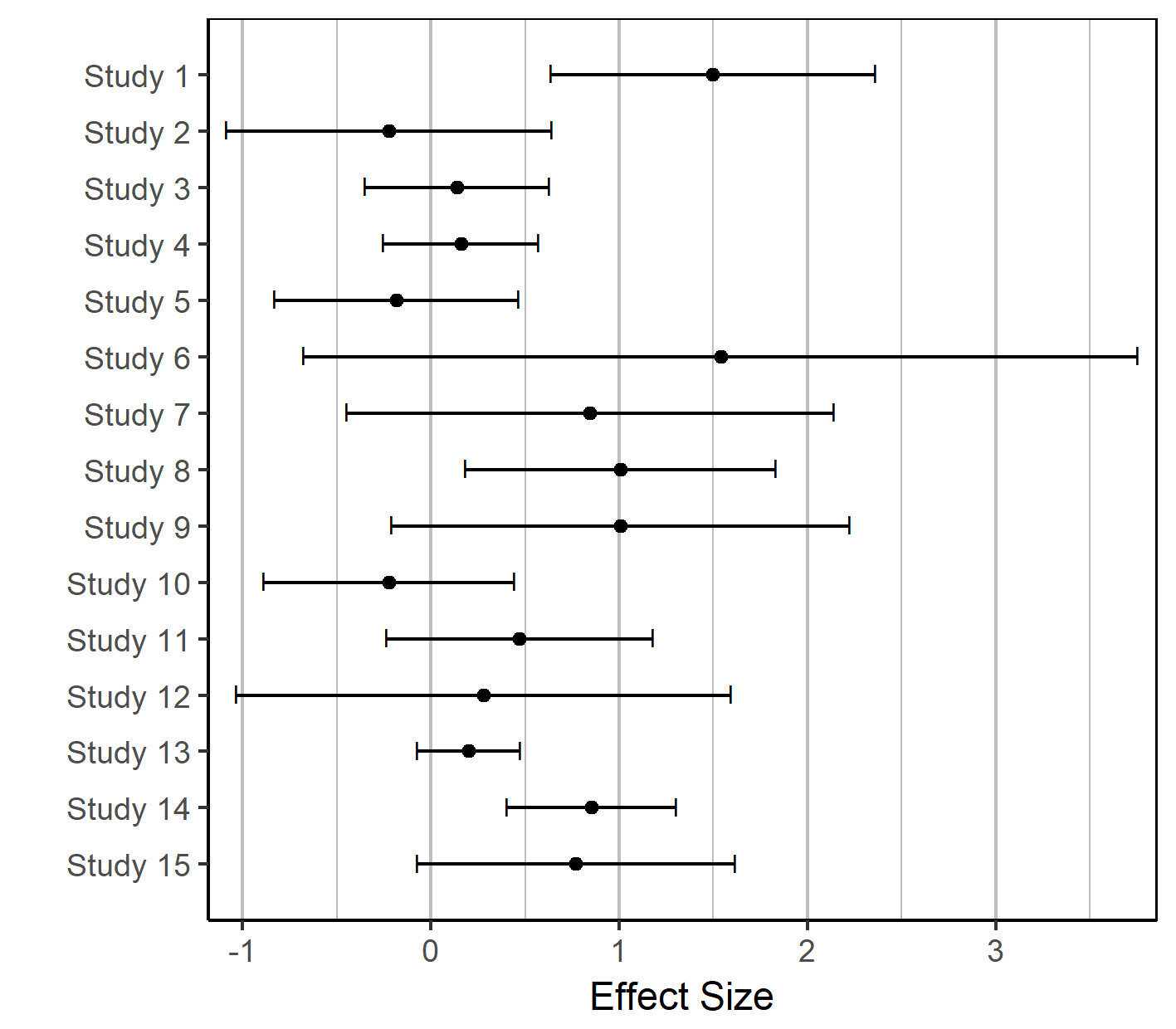 | 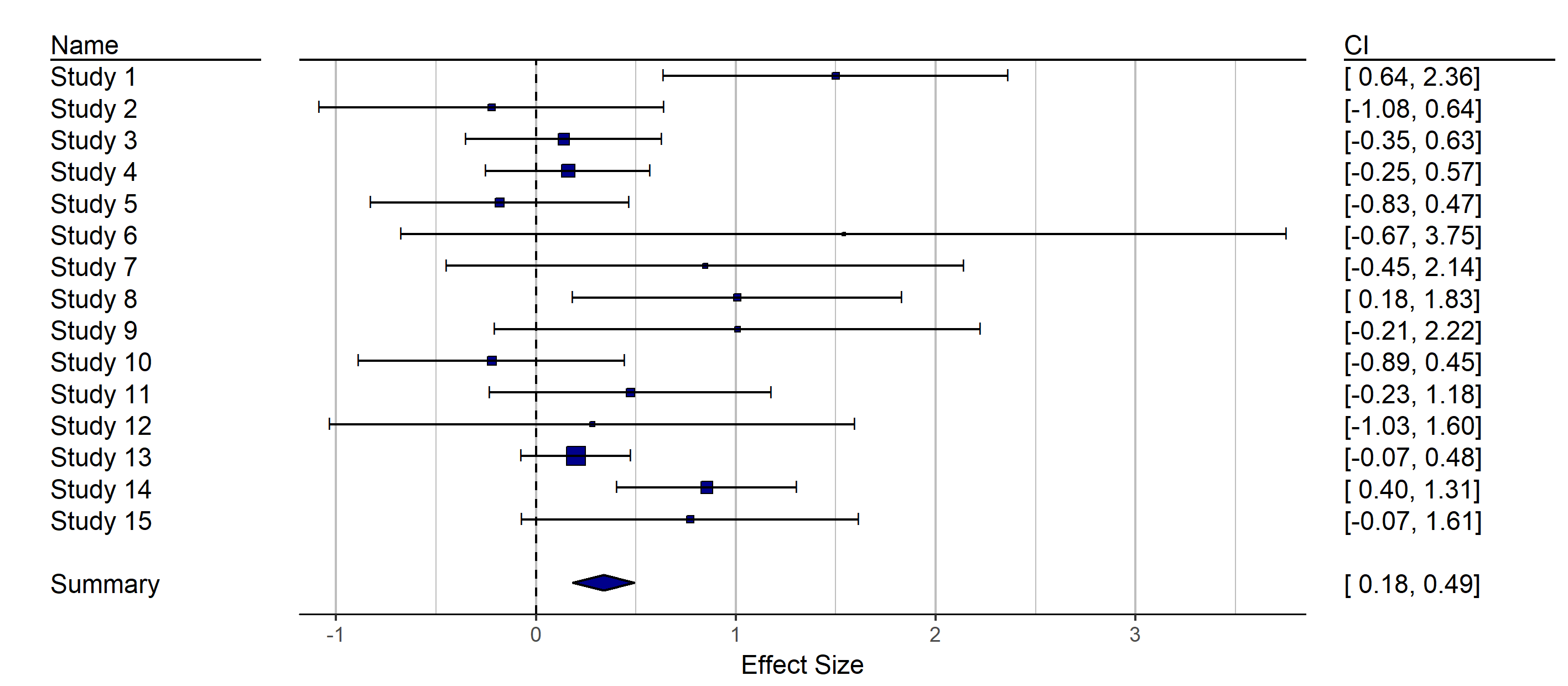 |
| The confidence interval plot is one of the oldest displays used in published meta-analyses. The plot shows study point estimates and their respective confidence intervals, but does not yet visualize any meta-analytic summary data, such as the meta-analytic summary effect or meta-analytic weights. Its use dates back to the very beginning of meta-analysis in the 1970s. One of the earliest applications can be found in Freiman, Chalmers, Smith and Kuebler (1978). The confidence interval plot is also often referred to as caterpillar plot, error bar chart, or ladder plot, and was not genuinely proposed for meta-analysis, but had been used more generally to compare point estimates and their confidence intervals, when estimated from different data sources. Although widely replaced by the forest plot [1.2] and its variants, it still is used to present study results, if they are not combined quantitatively in a meta-analysis. | The forest plot is probably the most iconic genuine display proposed for meta-analysis and nowadays is the most frequently used display for presenting meta-analytic results in published meta-analyses (Schild & Voracek, 2013). In essence, the modern forest plot is a display of each study effect size, the respective confidence interval, meta-analytic weight, as well as the meta-analytic summary effect and its confidence interval. The effect size of each study is conventionally depicted by a quadratic symbol, with area proportional to the meta-analytic weight. The summary effect is traditionally displayed by a diamond, highlighting the distinction between study effects (squares) and the meta-analytic summary effect (diamond). The width of the diamond directly corresponds to the confidence interval of the summary effect, whereas its height is uninformative. Often, a vertical line of no effect is shown. Confidence intervals not crossing that line indicate a significant effect. Forest plots are often annotated with additional textual information to the left or to right and therefore often are regarded as table plots. Tables might contain annotations on the study level (e.g., study names, effect sizes, weights, confidence intervals, sample sizes, moderators), as well as on the summary level (e.g., summary and heterogeneity statistics, and *p* values thereof). The sequence of the studies in the forest plot with respect to an ordinal covariate can give additional insight and often has been recommended, as compared to arbitrary or alphabetical sequences. For ratio measures (e.g., odds ratio, risk ratio), the log scale is widely used on the x axis in the forest plot. This has the advantage that the inverse of the ratio which corresponds to the same practical effect magnitude (say, an odds ratio of 2 and 0.5) is visually equidistant from unity, and the confidence intervals are symmetric. In the context of the random-effects model in addition to, or instead of, confidence intervals, predictive intervals have been shown. The origin of the forest plot remains somewhat unclear, but dates back to at least the 1980s, with the first variant including a summary effect having been published as early as 1982, while the first forest plot showing points proportional to weights was published in 1988 (for a historical account, see Lewis & Clarke, 2001). |

| Subgroup forest plot [1.2.1] | Summary forest plot [1.2.2] | Shrinkage plot, Bayesian forest plot [1.2.3] |
| --- | --- | --- |
| 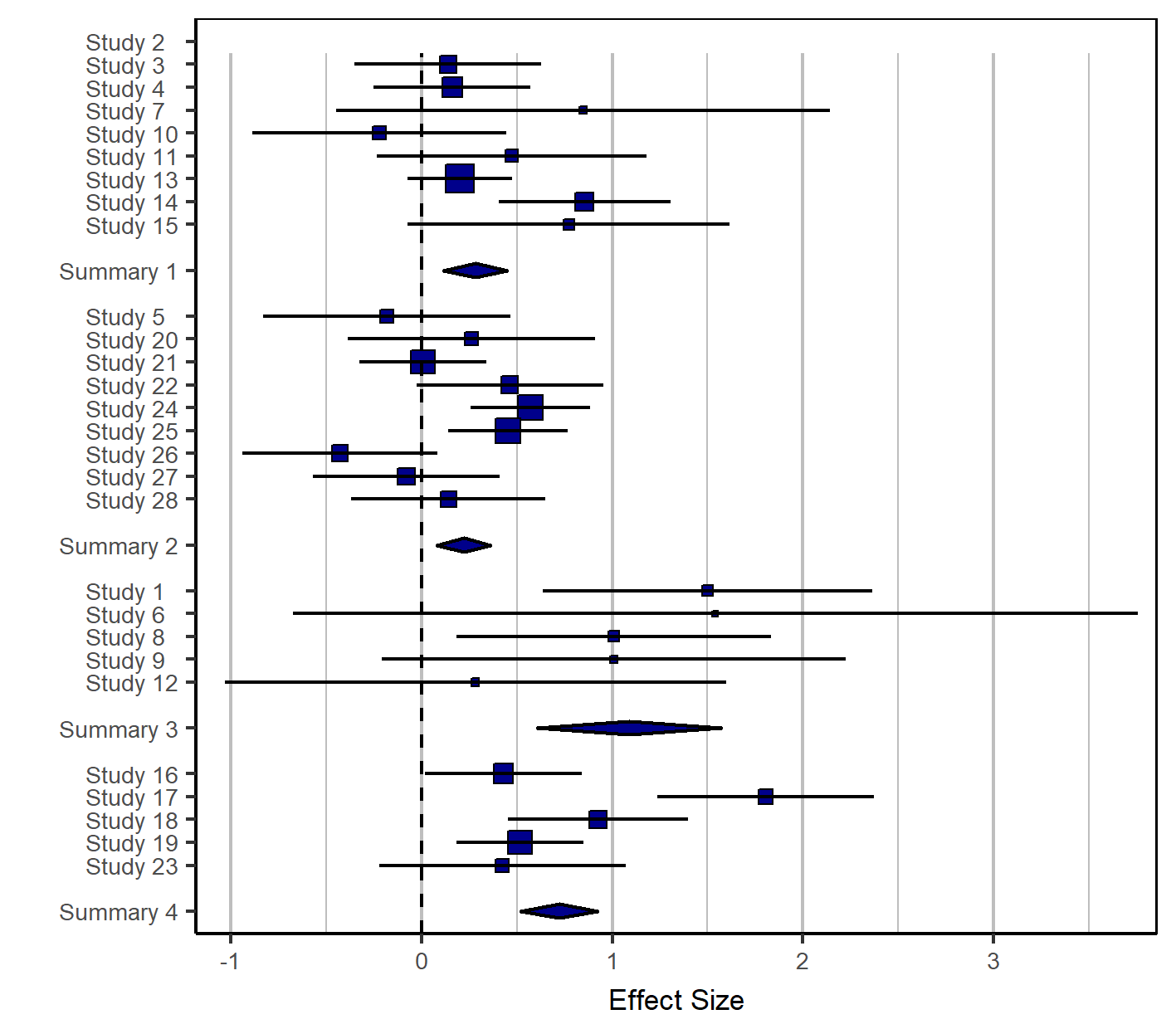 | 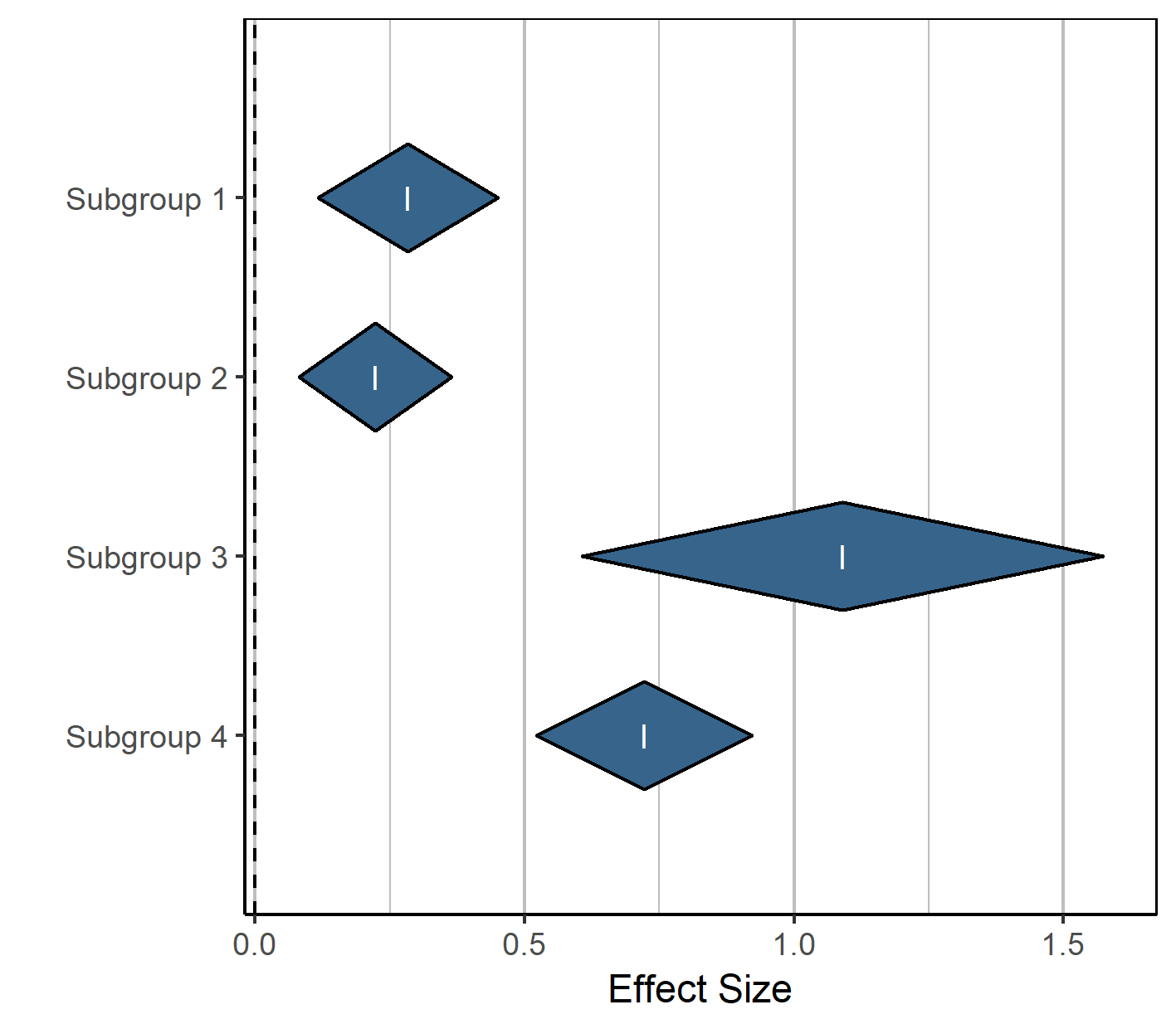 | 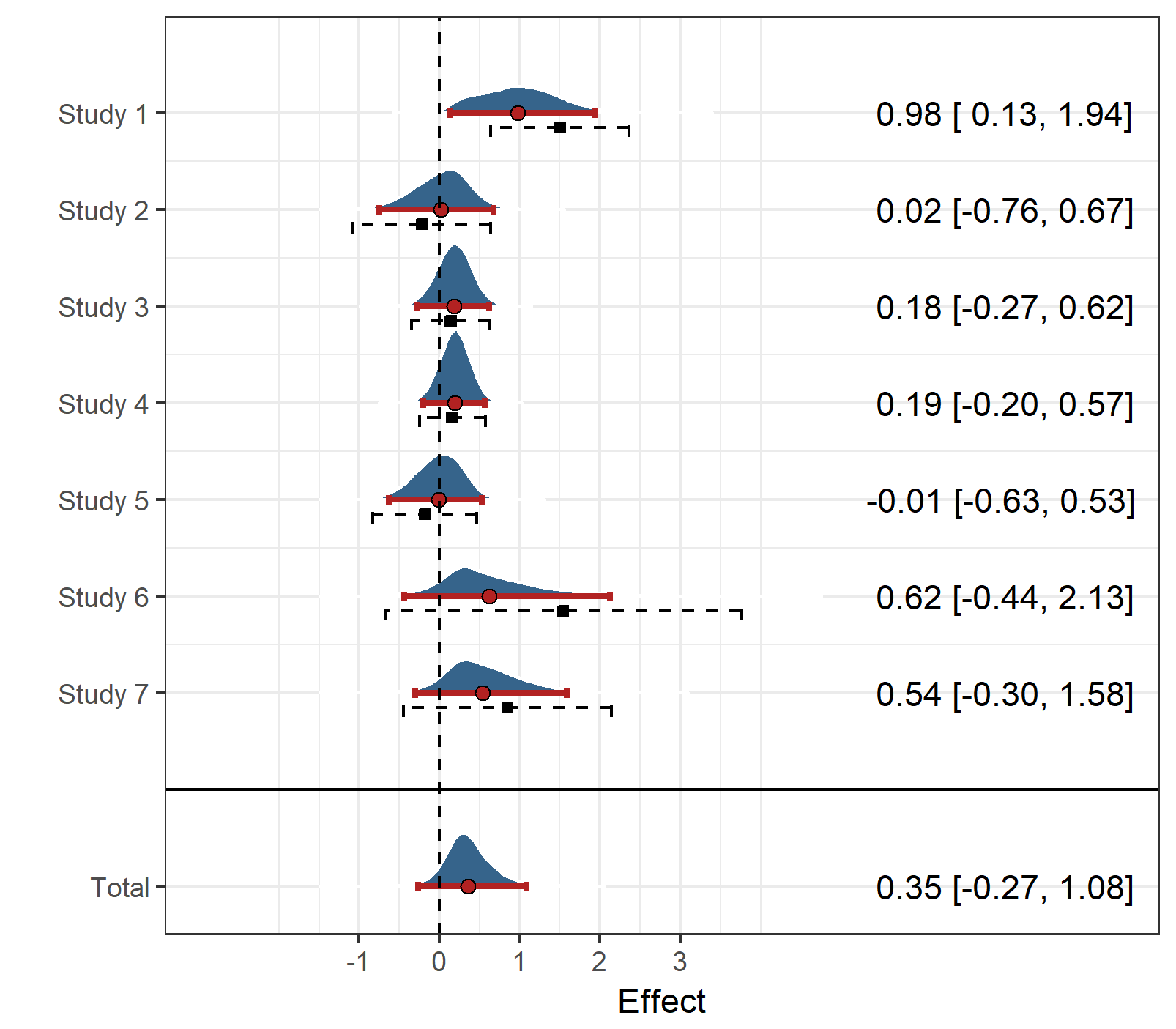 |
| The subgroup forest plot displays meta-analytic results of study subgroups with respect to a categorical moderator. Just like the conventional forest plot [1.2], the subgroup variant has come up as early as the 1980s. For each study subgroup, all study effects (including their confidence intervals) are shown grouped together in the forest plot. In addition, for each subgroup also the respective summary effects are regularly shown. The size of the diamonds representing the subgroup summary effects might be chosen as either constant or – especially if the overall summary effect is shown as well – as proportional to the weight contribution of all studies within a subgroup to the computation of the total summary effect. For an early example application, see Hyde and Linn (1986, p. 198). | Contrary to the subgroup forest plot [1.2.1], this variant has been used to exclusively show the summary effects of each subgroup, whereas no study-level results are shown. This might be beneficial, if the number of studies within subgroups is large, or the focus primarily is on differences between subgroups. To further reflect differences in the amount of information between subgroups, the size of the subgroup symbol has been plotted proportional to the meta-analytic weight of studies within this subgroup contribute to the overall meta-analytic summary effect. For an early example, see Hyde and Linn (1986, p. 198). | Shrinkage plots have been used in the context of meta-analyses based on Bayesian methodology. As in the classic forest plot [1.2], for each study the observed effect and confidence interval is shown (squares and dashed lines in the example plot above). In addition, the mean of the posterior distribution and the corresponding 95% credibility intervals are shown for each study, as well as for the summary effect (in red in the example plot above). These posterior means are generally closer (shrinked) to the prior mean than the observed study effect size, hence the name shrinkage plot. Estimates for the posterior distribution density have been shown as well. For an example, see Normand (1999). |
| Raindrop plot [1.2.4] | Limits of equivalence forest plot [1.2.5] | Confidence distribution plot [1.2.6] |
| 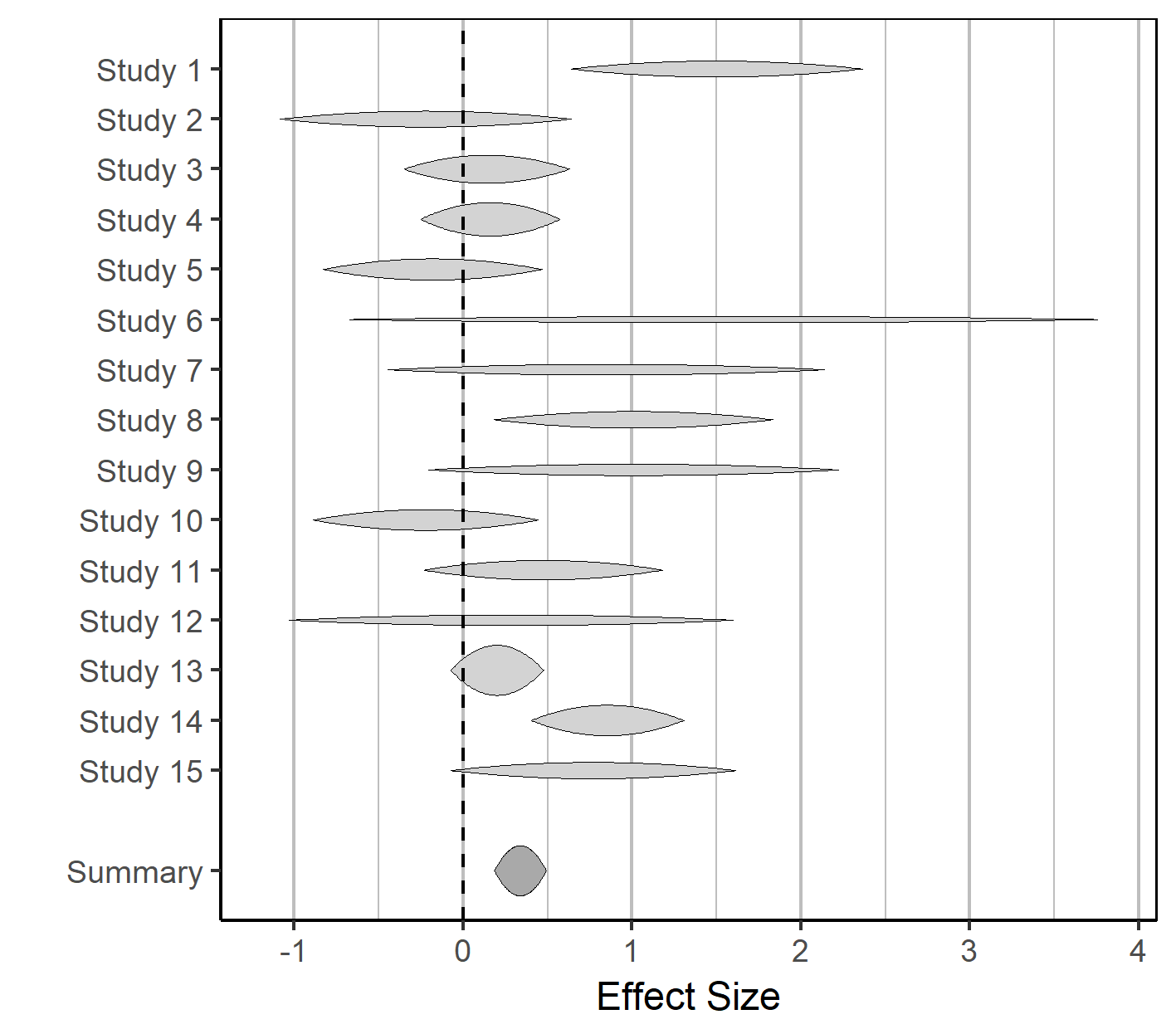 | 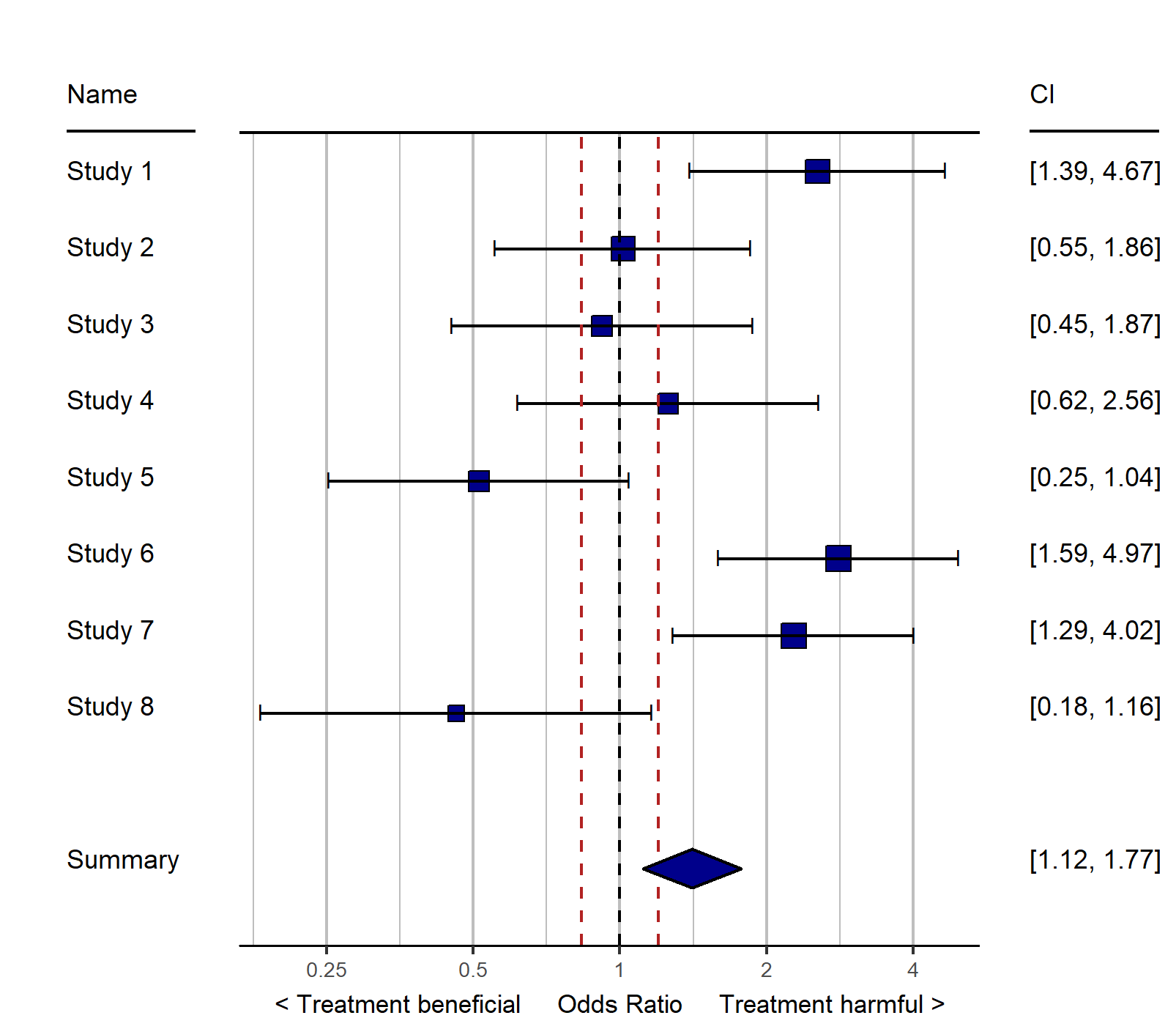 | 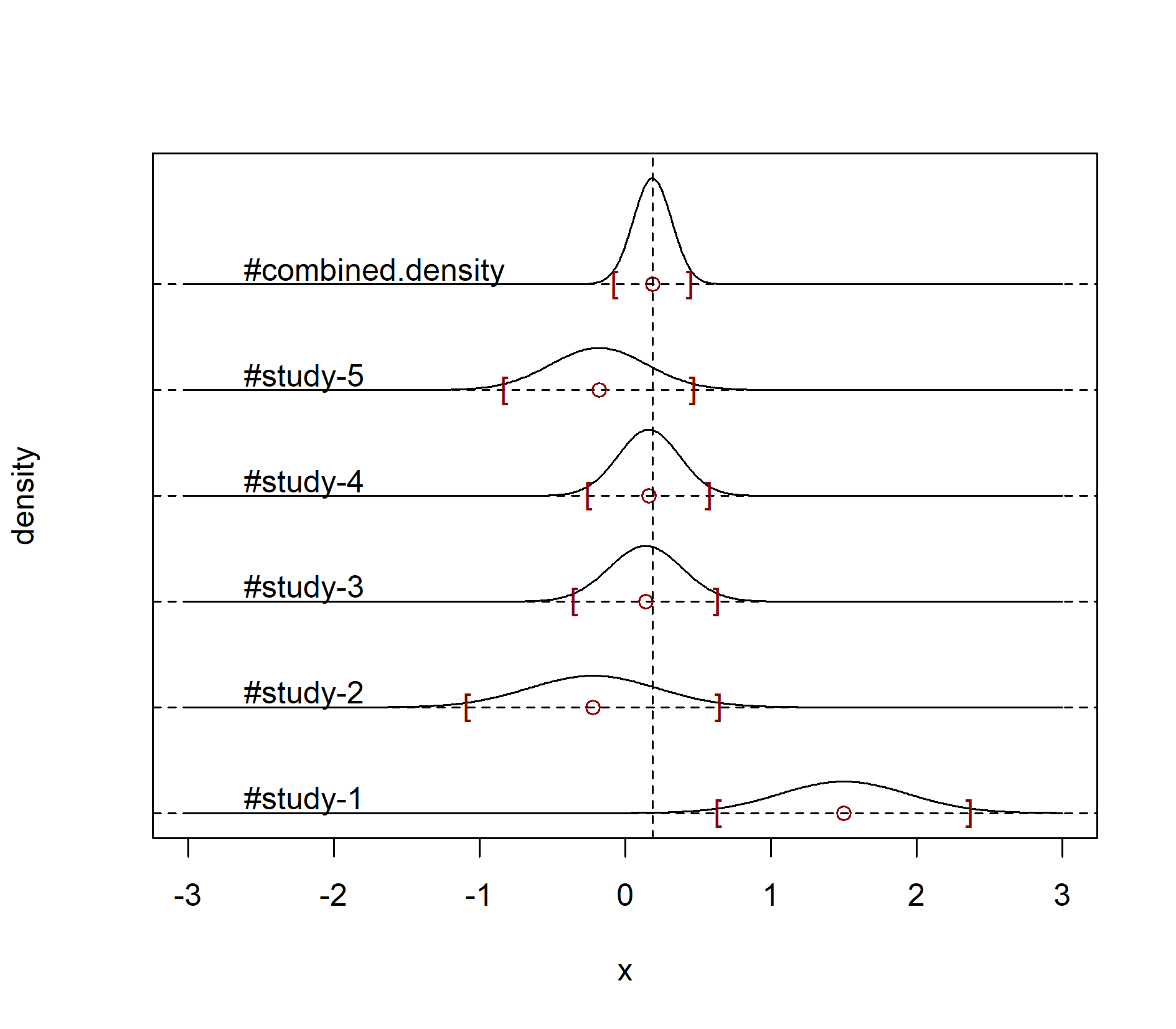 |
| Raindrop plots were not exclusively proposed for meta-analysis, but more generally to display a collection of likelihoods (Barrowman & Myers, 2003). The width of the raindrop corresponds to the (likelihood-based) confidence interval, whereas the height of the raindrop at a certain point is proportional to the log-likelihood for this parameter value. The height of the raindrop therefore informs about the plausibility of true values considering the observed data. By assuming normality of effect sizes – a widely used assumption in meta-analysis – the raindrops are symmetric around the estimated point estimate and the width of the raindrop corresponds to the Wald confidence interval traditionally used in forest plots. However, the shape of the raindrop depends on the assumed likelihood and thus is not necessarily symmetric, if other distributions than normality are assumed (for examples, see Barrowman & Myers, 2003). | Vertical reference lines have been drawn in the forest plot to allow the comparison of point and interval estimates with values of particular interest. Common is the inclusion of a line of no effect (see forest plot [1.2]). Another variant is based on limits of equivalence lines. This display was mainly proposed in the context of clinical research and especially meta-analyses concerning treatment effectiveness. The limits of equivalence are a user-specified threshold, which have to be exceeded, such that the drug or treatment is regarded as relevant (either beneficial or harmful, as compared with a standard therapy). Incorporation of these lines of evidence allows to assess whether observed study results and the meta-analytic summary result show statistically superior (beneficial), inferior (harmful), equivalent, or yet unclear effects, as compared to standard therapy (for details, see Sutton, Cooper, Jones, Lambert, Thompson & Abrams, 2007). They therefore allow the viewer to examine results of a test for equivalence as well as minimal-effect hypothesis testing on the study level and the summary level. | The confidence distribution plot is an enhanced variant of the traditional forest plot [1.2] for visualizing meta-analytic results, utilizing the confidence distribution framework (see Xie & Singh, 2013). For each study and the meta-analytic summary effect, the confidence distribution estimator regarding the true parameter is shown. The confidence distribution can be seen as the frequentist equivalent of the Bayesian posterior distribution (see also [1.2.3]). It shows the traditional confidence interval, but in addition might be interpreted as conveying information about the plausibility of true parameter values. Although rooted in confidence distribution theory, the usually conveyed information resembles raindrop plots [1.2.4], which, in turn, are rooted in likelihood and likelihood confidence interval theory. The above example graph was created using the specialized R package gmeta for meta-analysis, drawing on the confidence distribution framework (Yang, Cheng, Xie & Qian, 2017). |
| Rainforest plot [1.2.7] | Thick forest plot [1.2.8] | Contour-enhanced forest plot [1.2.9] |
| 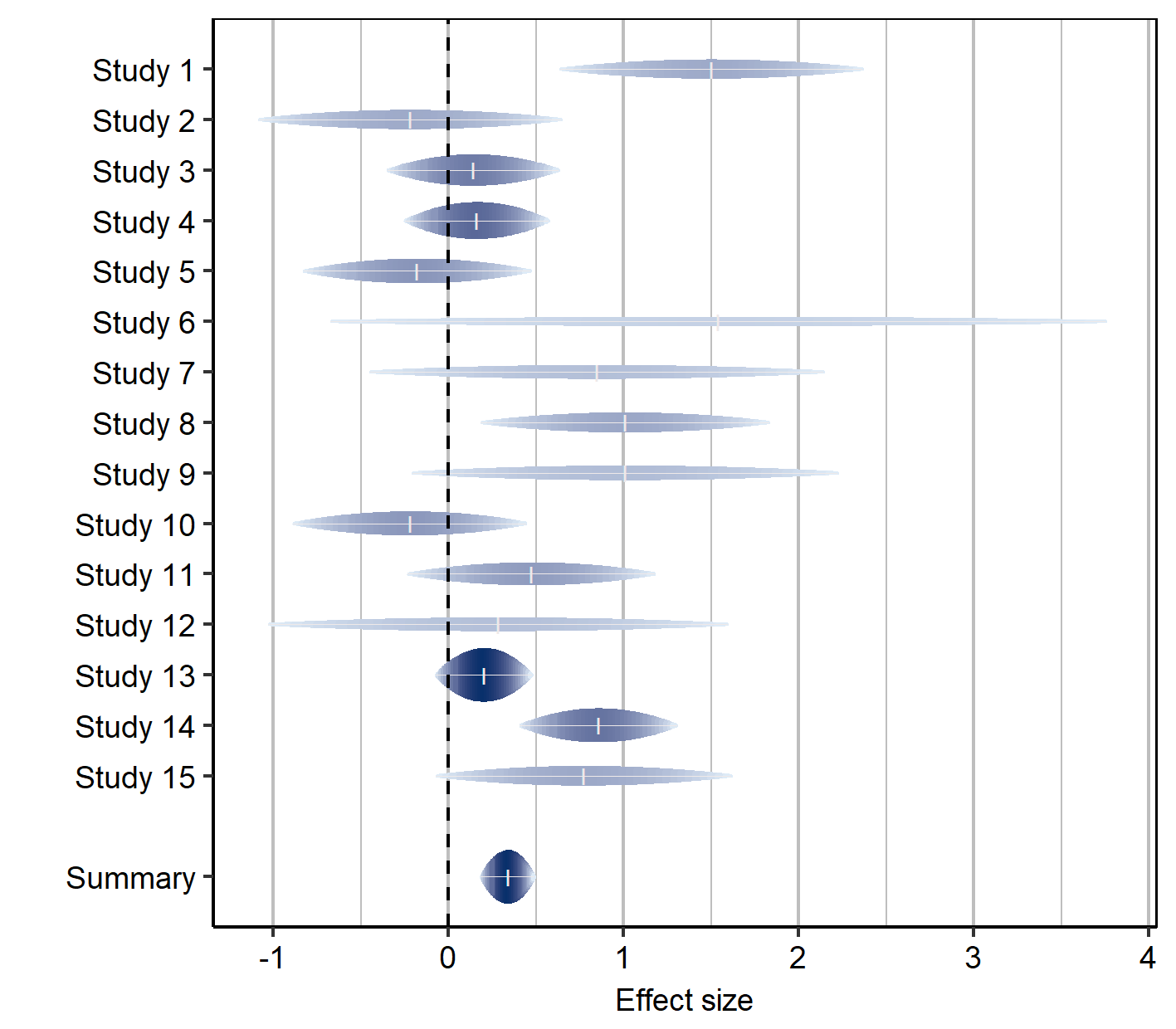 | 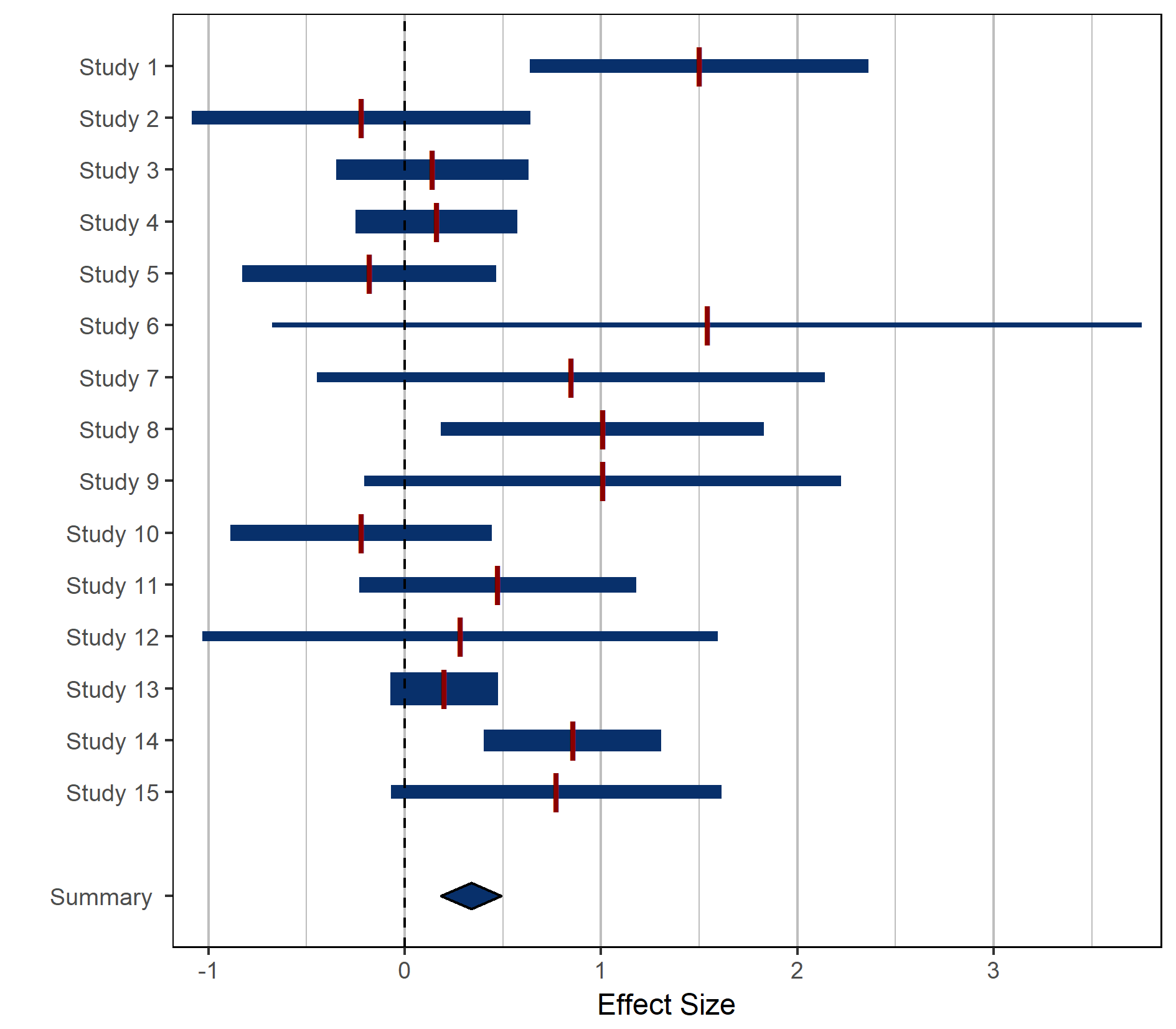 | 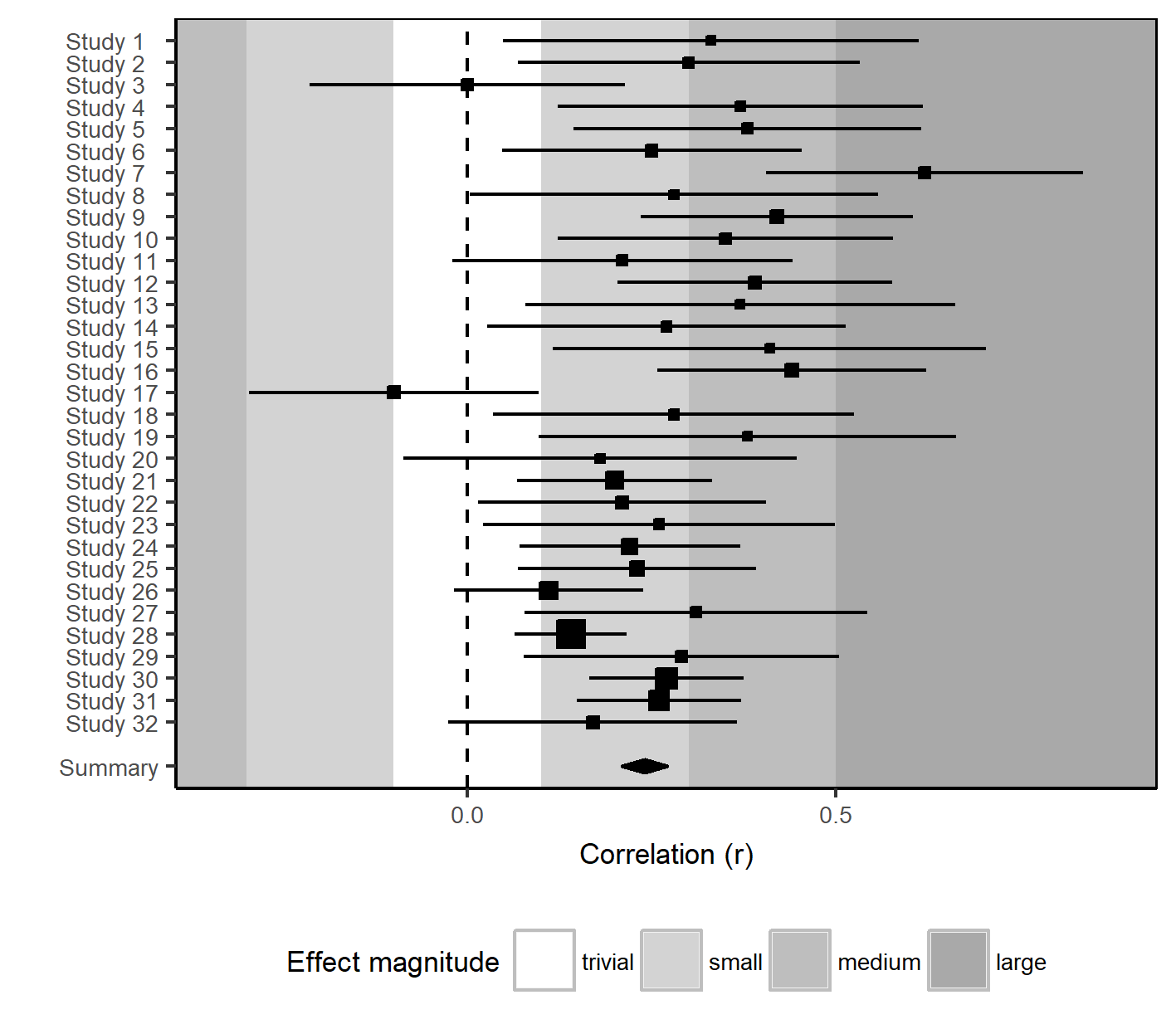 |
| The rainforest plot is an enhanced version of the traditional forest plot [1.2]. The rainforest plot was proposed to address three potential shortcomings of the traditional forest plot (Schild & Voracek, 2015). First, in the traditional forest plot long confidence intervals might draw unwarranted visual attention to studies reporting the least precise estimates. Second, confidence intervals might be incorrectly interpreted such that all points within the interval are equally likely. Third, for studies with large meta-analytic weights and therefore large boxes representing the estimates, the exact point estimate might be hard to discern. The rainforest plot overcomes these shortcomings by using likelihood raindrops (see raindrop plot [1.2.4]) and shading to depict uncertainty around the point estimate. This reduces the visual dominance of imprecise estimates. In addition, the different plausibility of true values around the point estimates are explicitly displayed. Finally, the exact point estimates are depicted with a dedicated tick mark. | The thick forest plot is – like the rainforest plot [1.2.7] – an enhancement of the traditional forest plot [1.2] (Schild & Voracek, 2015). It addresses some shortcomings of the traditional forest plot (see also rainforest plot [1.2.7]), by showing confidence bars with their thickness proportional to the meta-analytic weights of the studies and dedicated tick marks at the point estimates. This draws less visual attention to the least precise estimates (with the longest – but in the case of thick forest plot slimmest – confidence bars), whereas more visual attention to the most precise estimates (with the shortest - but thickest - confidence bars). Dedicated tick marks help to discern the precise point estimates of each study. | A forest plot variant with effect-magnitude contours has been proposed recently (Nakagawa, Noble, Senior, & Lagisz, 2017). Differently shaded areas indicate user-specified categories of effect magnitude. These categories often follow field-specific conventions (in the above example, following Cohen, 1992, absolute correlations below .10, .30, .50 are labeled as trivial, small, and medium, respectively, whereas correlations above .50 are labeled as large). |

| Odd man out plot [1.3] | Fuzzy number plot [1.4] | Fuzzy number plot with subgroups [1.4.1] |
| --- | --- | --- |
| 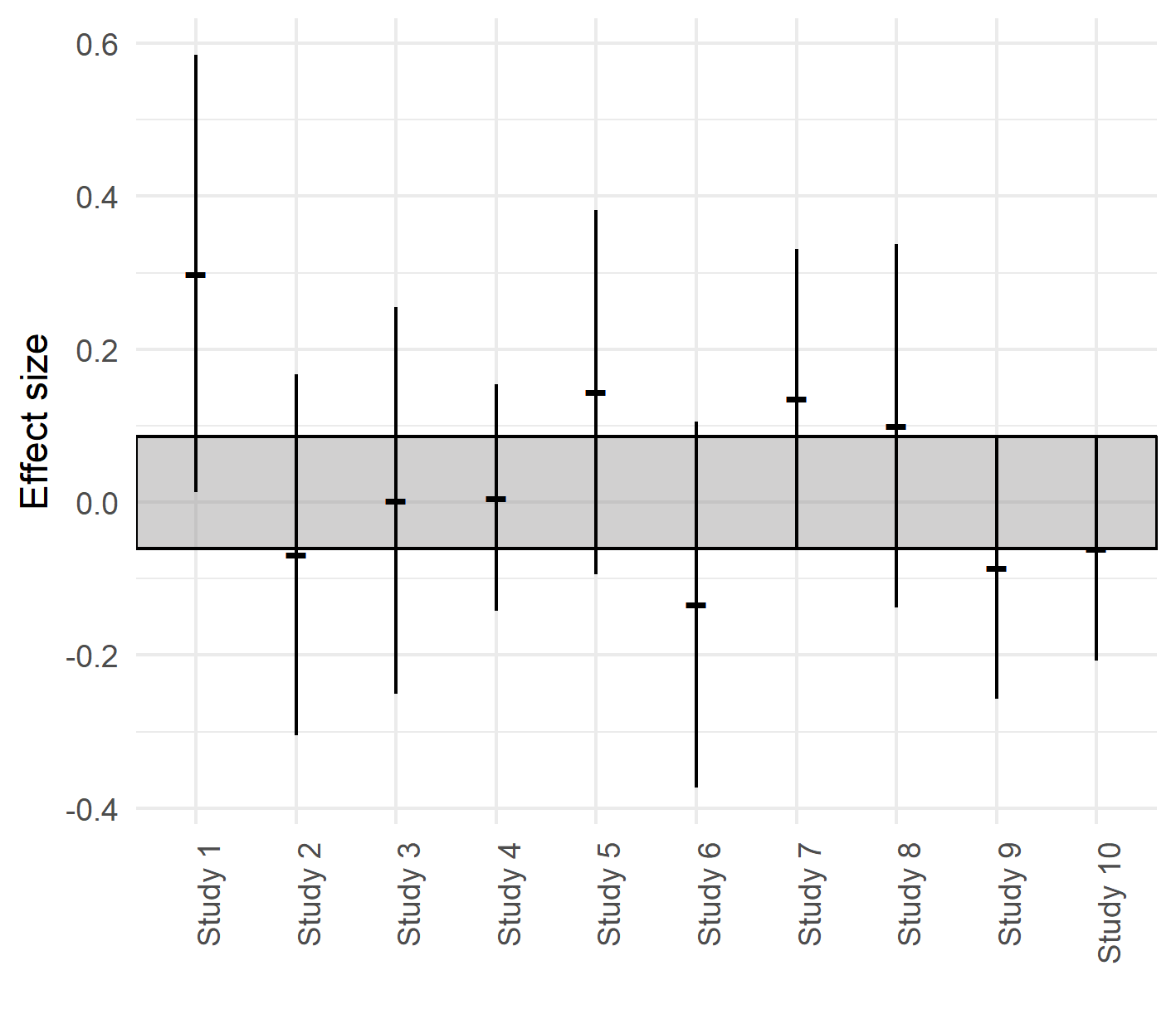 | 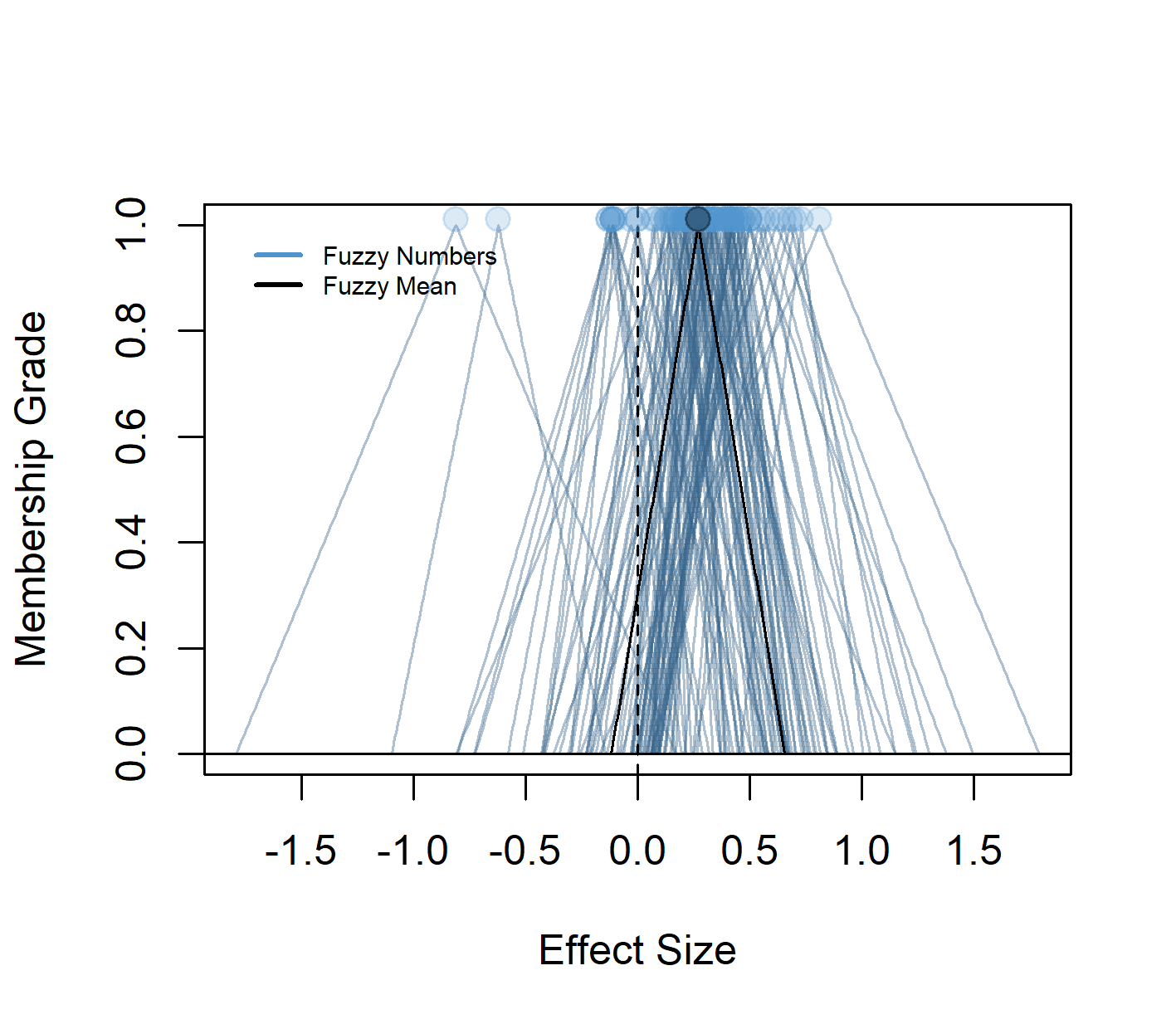 | 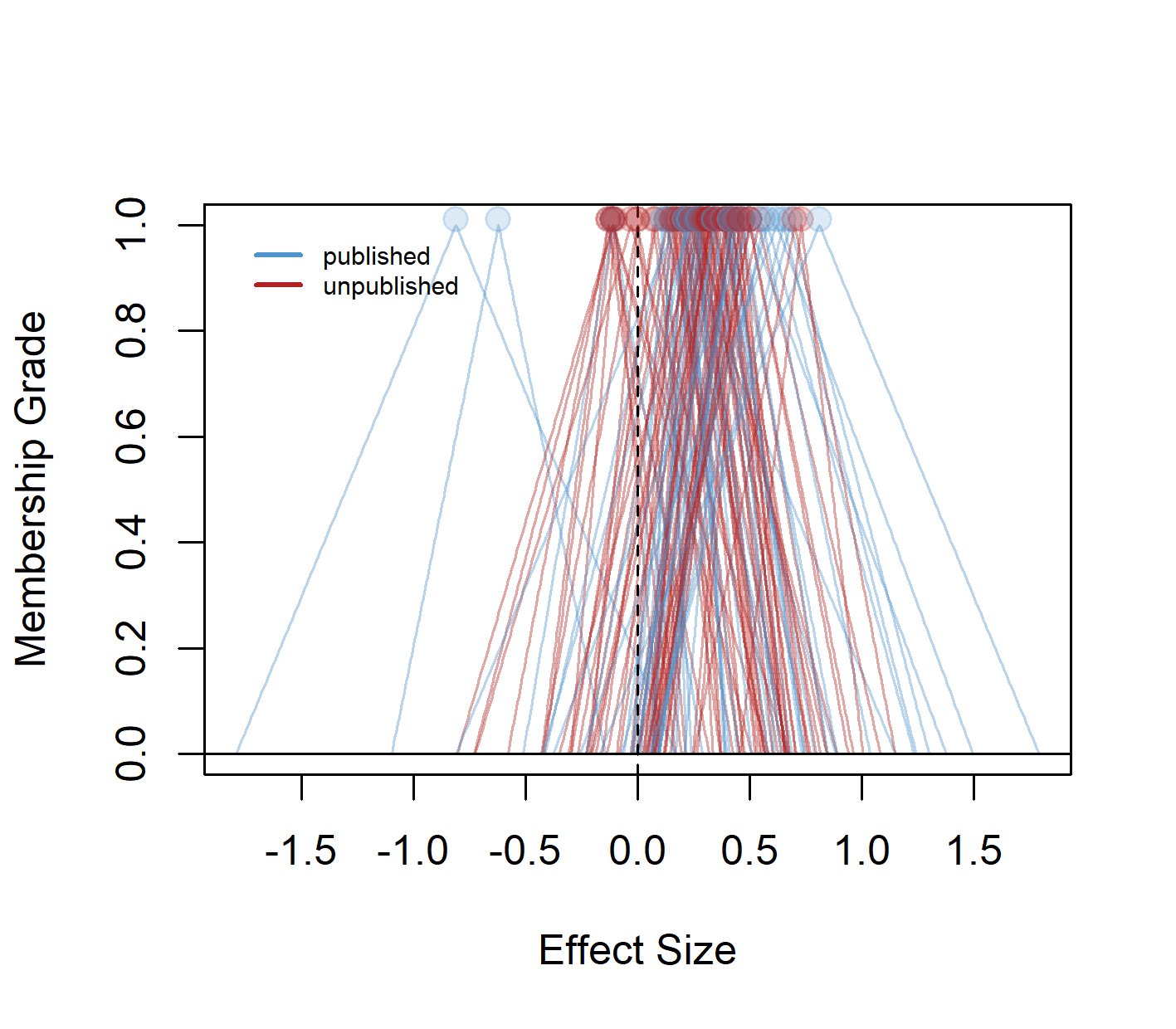 |
| Odd man out is a lesser-known graphical approach to meta-analysis, proposed as early as 1988 by Walker, Martin-Moreno, and Artalejo. For N studies, a summary confidence region is constructed by highlighting effect size areas where at least N-k of all N confidence intervals overlap. The most natural choice for k is 1 (as in the above graph). For a large number of independent confidence intervals for the same parameter, the (a priori) probability that each and every confidence interval contains the true parameter decreases rapidly with growing N. However, the probability that at least N-1 confidence intervals contain the true parameter decreases less rapidly and more closely resembles the study-specific desired confidence level for typical sizes of N in the meta-analytic context. The resulting confidence region can therefore be seen as a rough approximation to a confidence interval for the underlying true effect (with the same confidence level as the study-specific confidence intervals). However, the confidence region in odd man out can consist of two (or more) distinct regions, which might be an indicator for heterogeneous study groups. | The fuzzy number plot displays study effect sizes, and optionally the summary effect, as fuzzy numbers (Thompson, 2016). Displaying an effect size as a (triangular) fuzzy number corresponds to a triangle with the lower side of the triangle defined as the effect size’s confidence interval and the upper apex of the triangle defined as the studies’ effect size (for a more elaborated description, see Thompson, 2016). Each effect size is additionally depicted by a dedicated point at the top of the triangle, and a line of no effect is shown. The fuzzy number plot is therefore essentially constructed with the same information than the forest plot [1.2]. Transparency is used for plotting points and the triangles to visualize areas of overlapping fuzzy numbers. The fuzzy number plot might aid in the assessment of plausible effect size regions (dense regions of overlapping triangles), precision of studies (imprecise studies are depicted by wide triangles), and between-study heterogeneity (outlying triangles or hardly overlapping clusters of triangles). | The fuzzy number plot [1.4] can also be used to display different subgroups of studies. The fuzzy number plot with subgroups utilizes different colors for studies in different subgroups to depict subgroup membership (Thompson, 2016). This allows to explain different clusters of studies or outlying studies in the fuzzy number plot with the help of categorical study features (e.g., experimental vs. observational studies, unpublished vs. published studies). Fuzzy number plots ([1.4], [1.4.1]) were created using and adapting R code from supplemental material of Thompson (2016). |

| Funnel plot [2.1] | | | | | Subgroup funnel plot [2.1.1] | | |  |  |
| --- | --- | --- | --- | --- | --- | --- | --- | --- | --- |
| 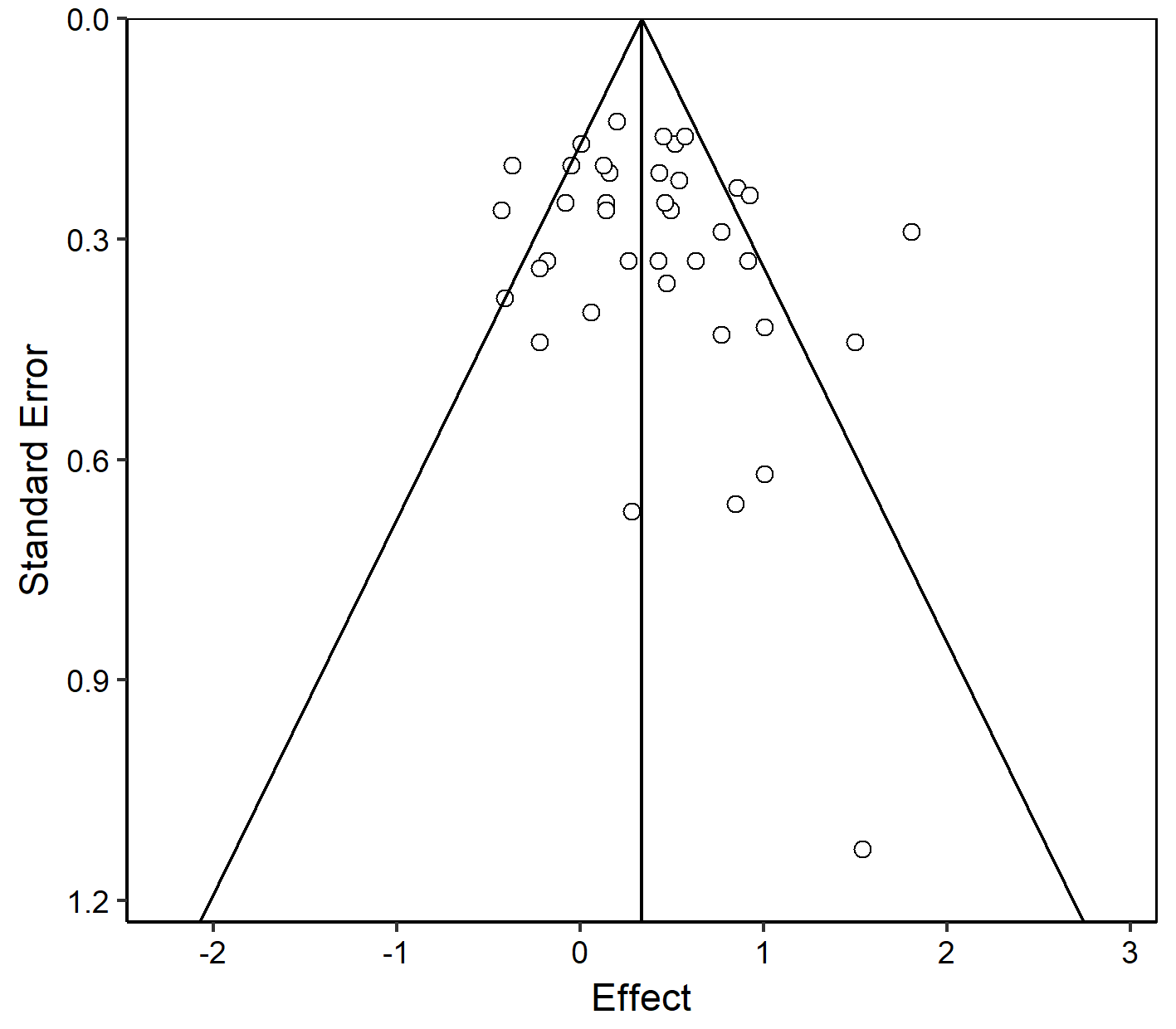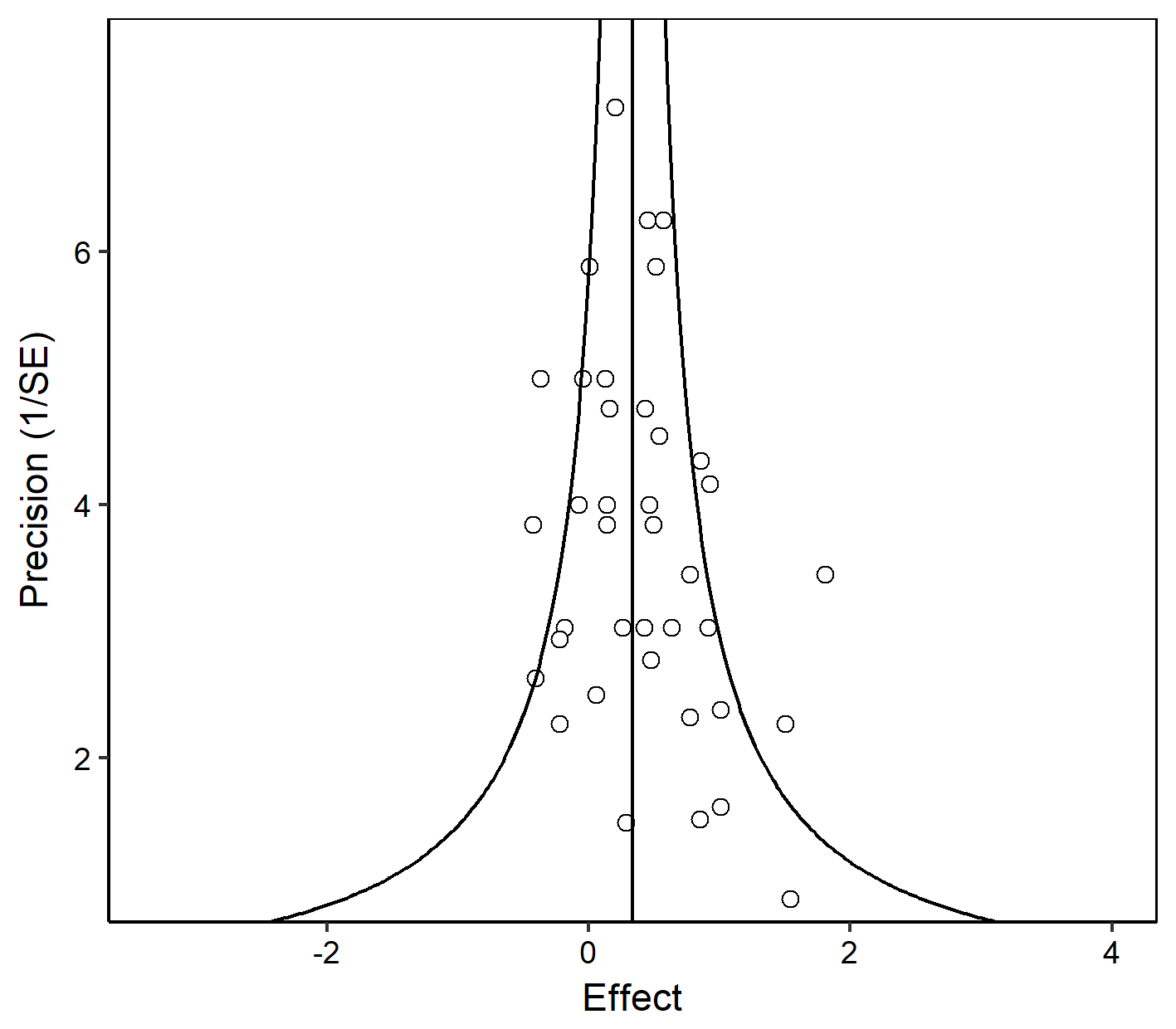 | | | | | 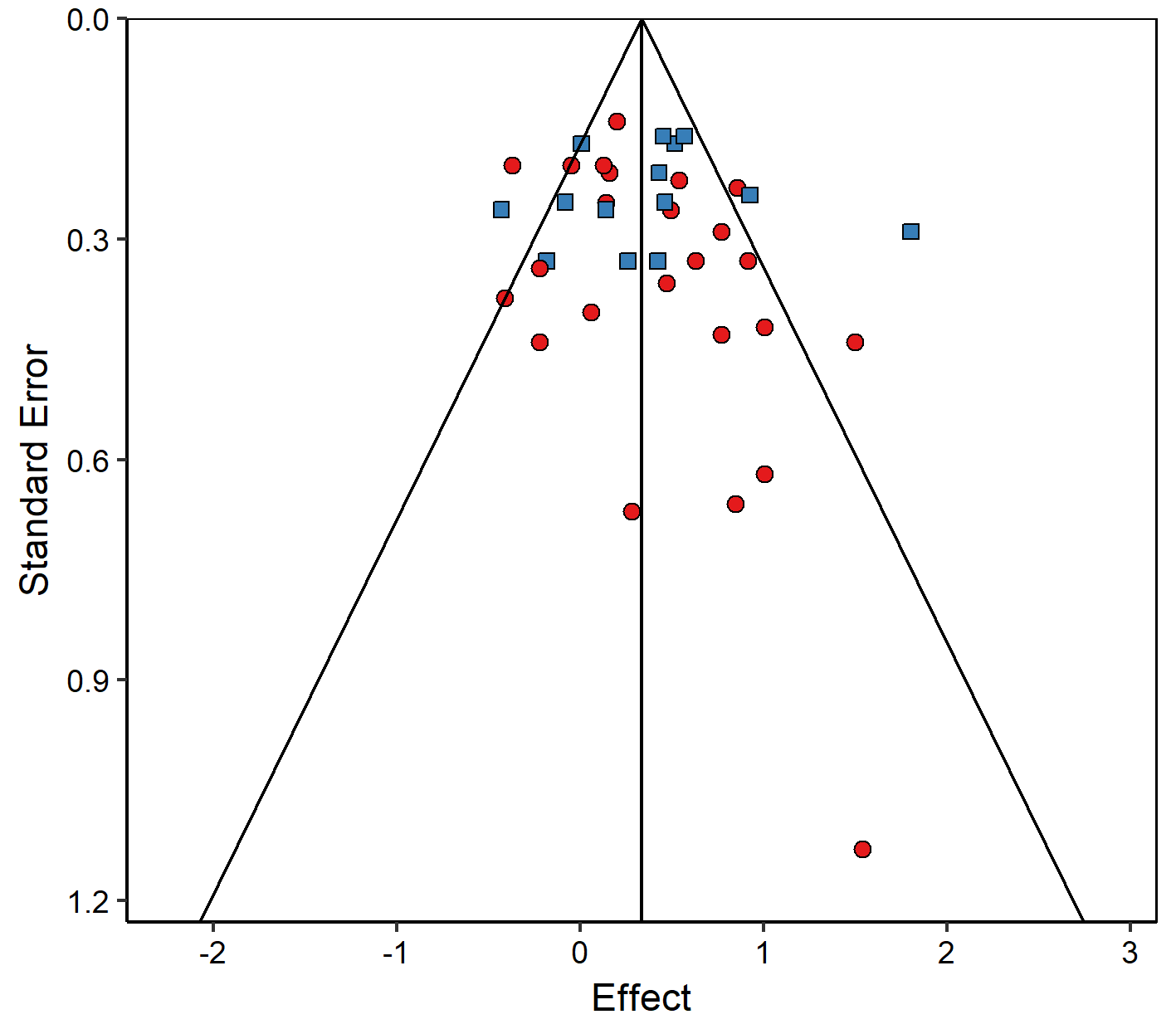 | | |  |  |
| The funnel plot (Light & Pillemer, 1984, pp. 63-73) is the most widely used diagnostic plot for meta-analytic data to investigate publication bias and other biases (Schild & Voracek, 2013). The funnel plot is a scatterplot with the study effect sizes on the abscissa and a measure of variance, such as standard error (left example) or precision (right example), on the ordinate (Sterne & Egger, 2001). Under the meta-analytic fixed-effect and random-effects models, studies should scatter randomly and symmetrically around the meta-analytic summary effect, with a higher effect variation at the bottom of the graph than on the top; hence, leading to the funnel shape. Certain deviations of this expected shape might be suggestive for publication bias. Two underlying assumptions about publication bias are that (a) larger studies (with higher precision) get more likely published than smaller studies (with low precision), and (b) studies with statistically significant findings likewise get more likely published. Therefore, publication bias leads to so-called small-study effects in the funnel plot, such that small studies (with low precision) on average seem to report larger effects than large studies (with high precision). In this scenario, the funnel plot is asymmetric and an association of effect size with standard error (or functions thereof) is apparent. However, publication bias is not the only possible explanation for such funnel-plot asymmetry; other explanations are true effect heterogeneity or chance alone. The meta-analytic summary effect is commonly shown as vertical line with 95% confidence contours around this summary effect. Confidence contours show the expected distribution of studies under the fixed-effect model (i.e., for normally distributed effect sizes in the absence of heterogeneity and publication bias). | | | | | Different plotting symbols or plotting colors have been used to visualize study subgroups within a funnel plot. This probably oldest variation of the classic funnel plot was already used not later than in 1984 (Light & Pillemer, 1984, p. 69). Visualizing subgroups within a funnel plot (instead, for example, within a subgroup forest plot [1.2.1]) allows to assess whether small-study effects might be driven by certain study groups. Although any categorical moderator can be used, especially popular choices have been unpublished vs. published studies, significant vs. non-significant studies, and studies authored by a specific author or group vs. all other studies. The usual hypothesis is that typical small-study effects should only be visible in one subgroup, but not in the other subgroup (or subgroups). | | |  |  |
| Regression test funnel plot [2.1.2] | | Funnel plot with weighted mean, median and mode [2.1.3] | | | | Trim-and-fill funnel plot [2.1.4] | | |  |
| 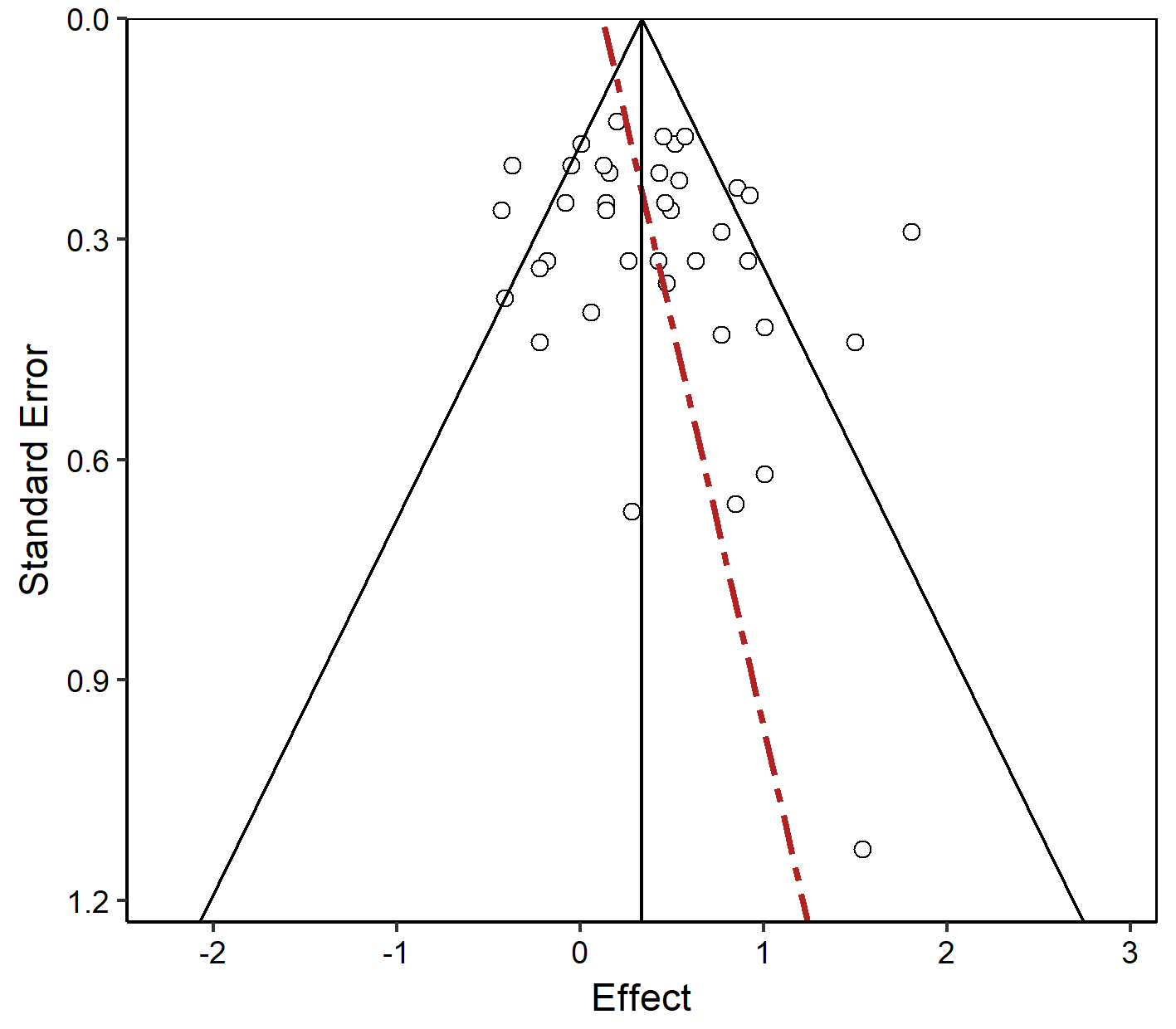 | | 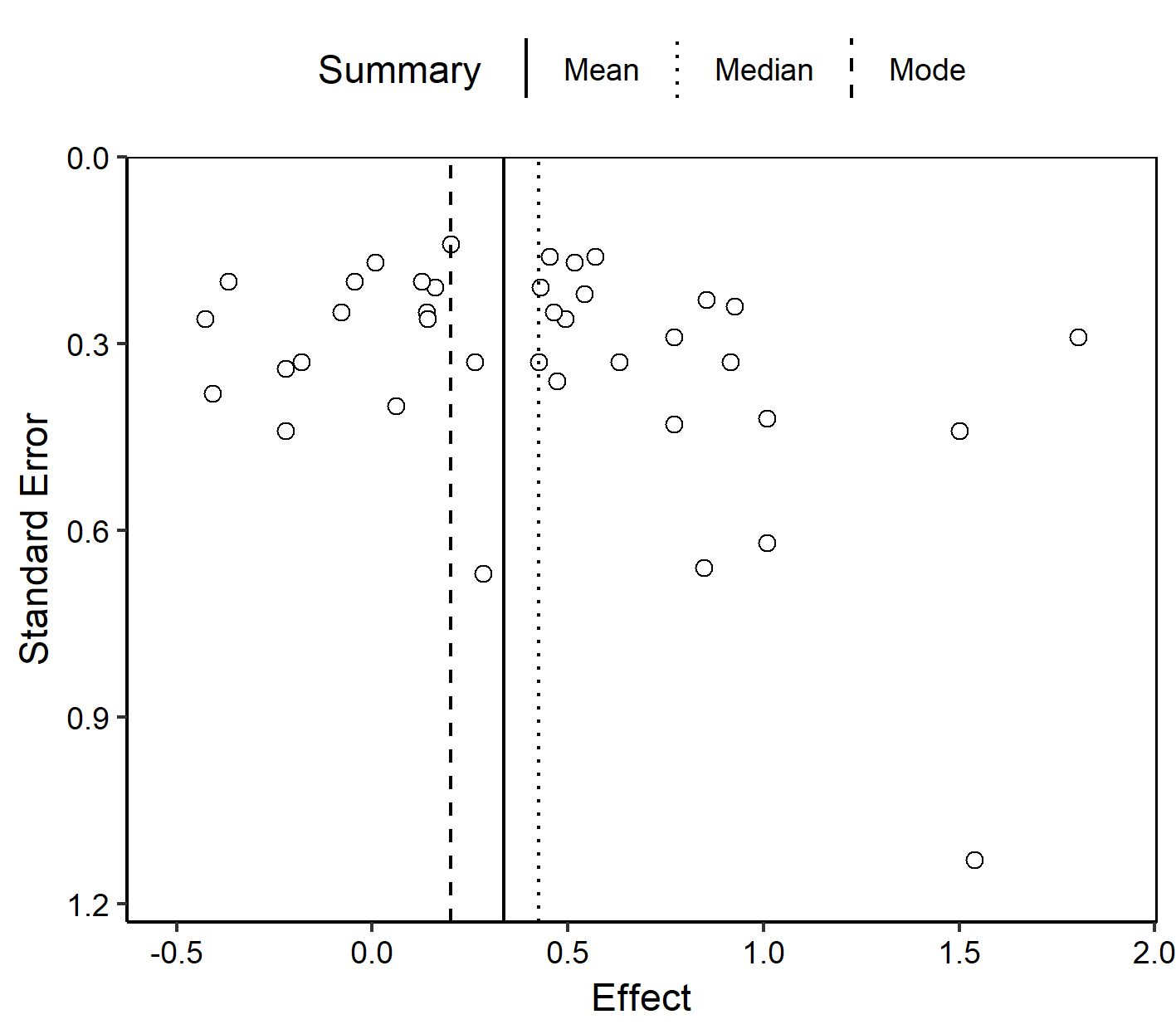 | | | | 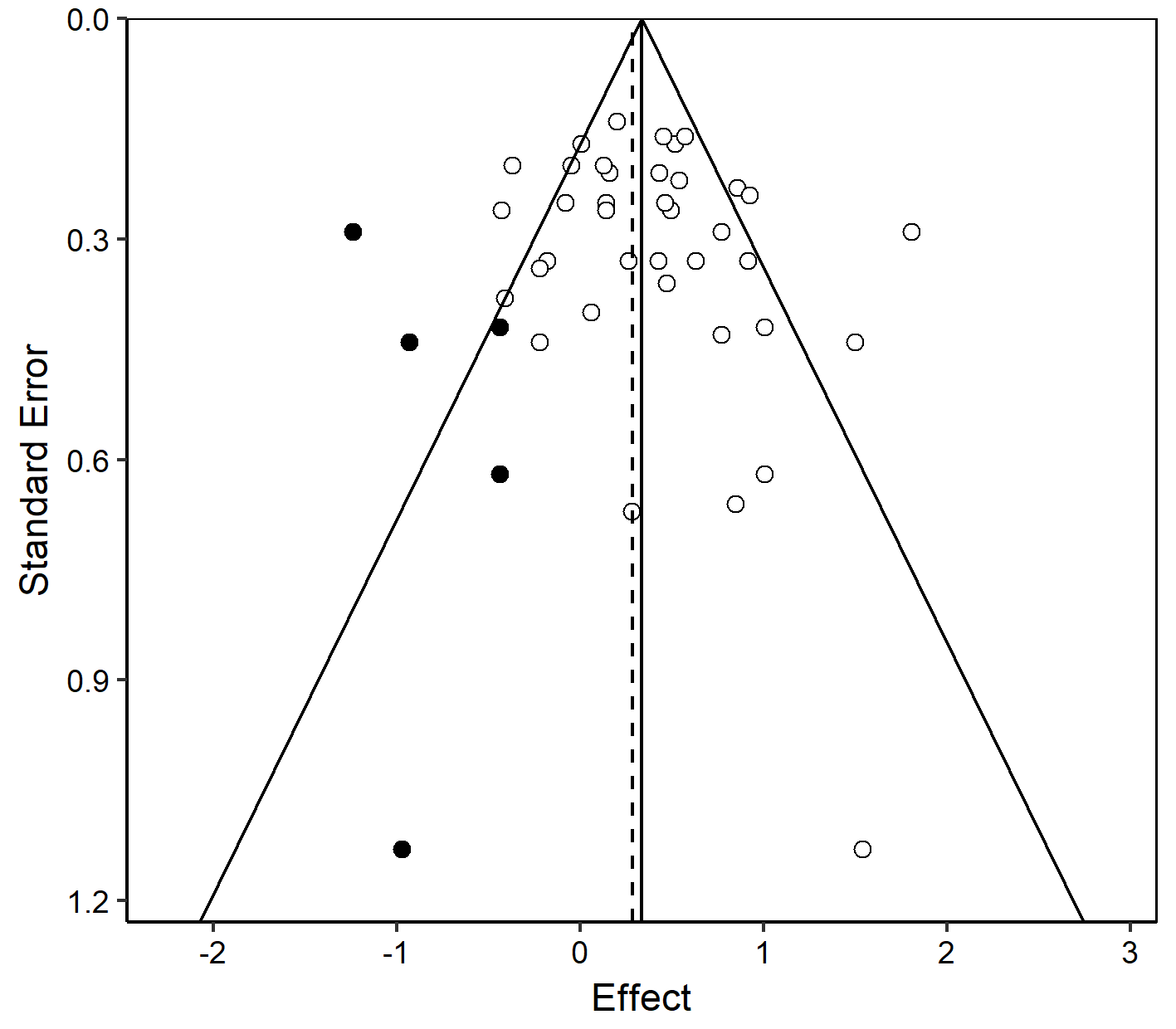 | | |  |
| Lines of fit of regression methods to quantify funnel plot asymmetry can be shown in the funnel plot to aid in the assessment of small-study effects. Popular examples of such methods are Egger’s regression (Egger, Smith, Schneider & Minder, 1997), as in the plot above, or the PEESE curve (Stanley & Doucouliagos, 2014). Egger’s regression test is based on the intercept of the ordinary linear regression line of the standardized effect size (effect size divided by its standard error) on the inverse of the standard error (see also the Galbraith plot [2.2]). A large intercept (in the direction of the hypothesized effect) then is interpreted as indicator for small-study effects. By rearranging the estimated regression equation this can be displayed in the funnel plot with the standard error on the y axis. The slope of the regression line then corresponds to Egger’s regression test and deviations from a vertical line show asymmetry. For a published example application, see Rothstein, Sutton and Borenstein (2005, p. 103). | | To aid the visual assessment of funnel plot asymmetry, showing three measures of centrality of the observed study effects has been proposed (Elvik, 1998): The weighted mean (i.e., the meta-analytic estimate), the weighted median (i.e., the value that divides the sum of meta-analytic weights into two equal parts), and the weighted mode (the value of the study with the largest weight). For symmetric distributions, the three measures of centrality should be equal or rather similar. For asymmetric distributions they can differ substantially. Skewness to the right is indicated by the mean being larger than the median, and the median being larger than the mode. The opposite is true for left-skewed distributions. | | | | The trim-and-fill method (Duval & Tweedie, 2000) estimates and corrects for the number of studies presumably missing due to publication bias. In this variant, imputed studies by the trim-and-fill method (in the above graph shown as black dots), as well as the adjusted summary effect (in the above graph shown as dashed line) are shown. This helps to identify studies driving funnel plot asymmetry. Also, the assumed effect of publication bias on the estimated meta-analytic summary effect can be assessed. The trim-and-fill algorithm basically estimates the number of (extreme) studies responsible for funnel plot asymmetry (see Duval & Tweedie, 2000). It then trims this number of (extreme) studies and computes the adjusted summary effect only considering the remaining studies. Finally, it imputes studies - presumably missing due to publication bias - by mirroring the trimmed (extreme) studies (driving the funnel plot asymmetry) around the adjusted summary effect. | | |  |
| Significance contour-enhanced funnel plot [2.1.5] | | Additional evidence contours funnel plot: Summary effect significance [2.1.6] | | | | Additional evidence contours funnel plot: Heterogeneity [2.1.7] | | |  |
| 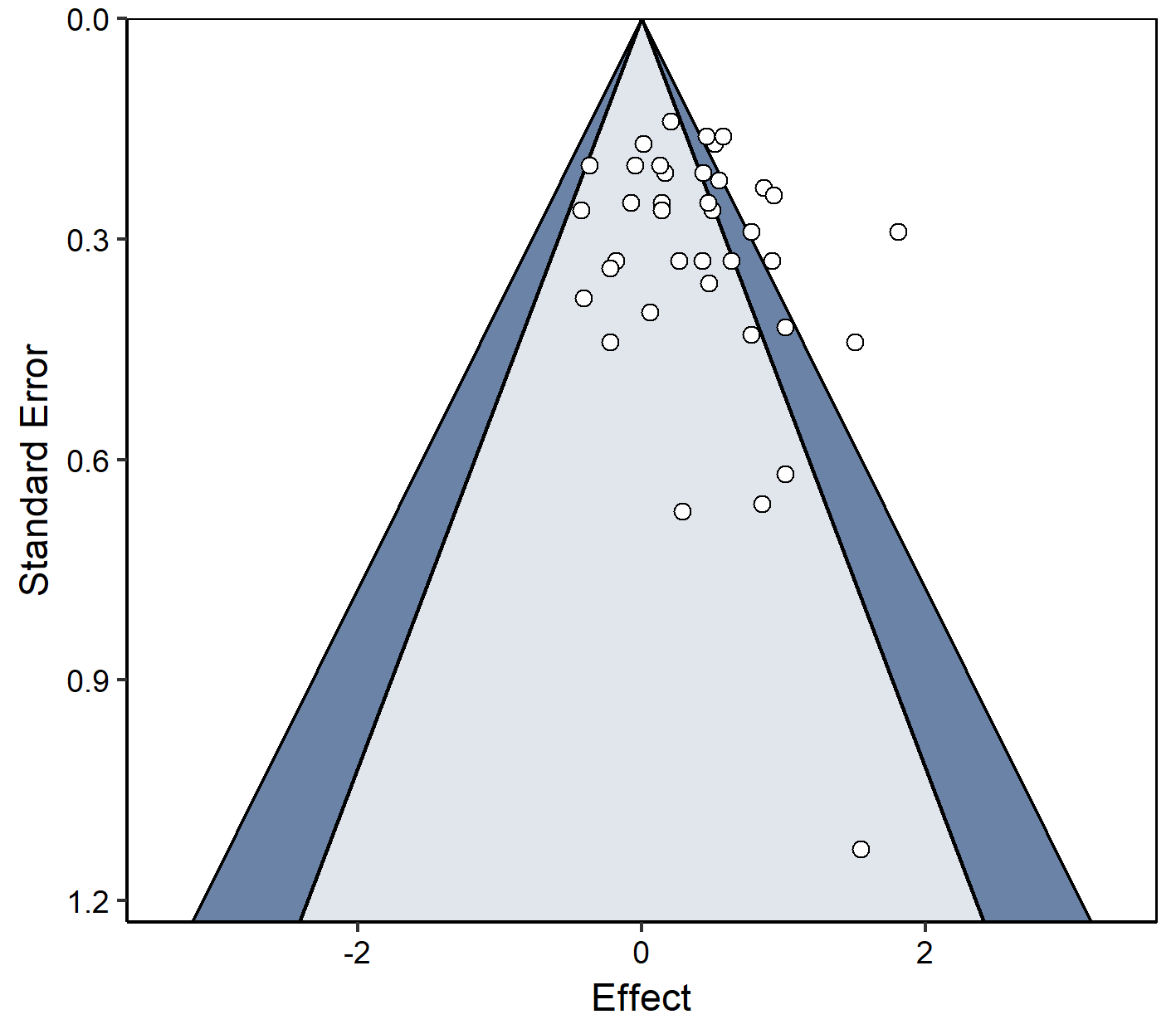 | | 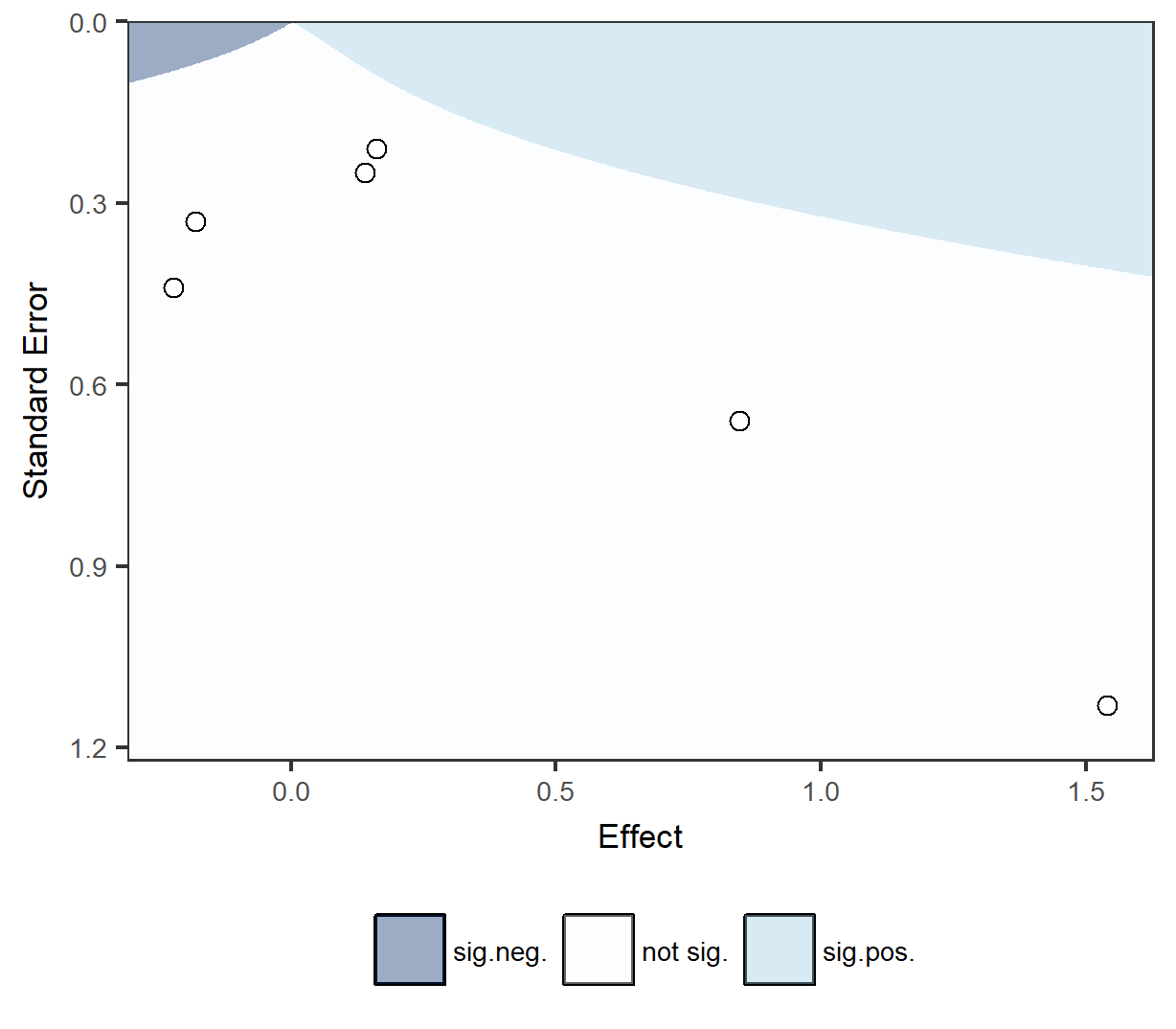 | | | | 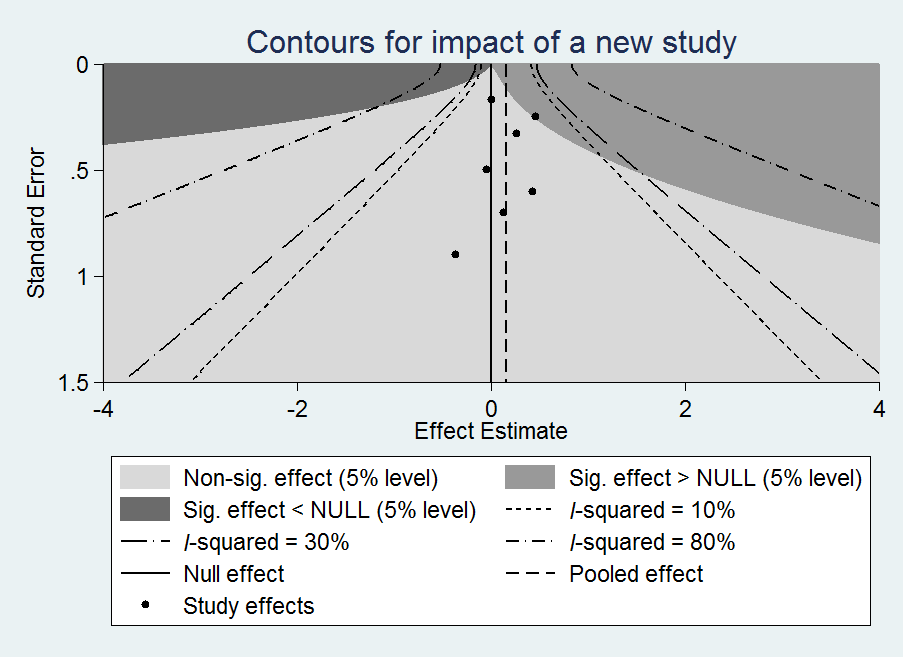 | | |  |
| The significance contour-enhanced funnel plot was proposed to assess whether small-study effects (i.e., asymmetry) in a funnel plot might be driven by conventional limits of statistical significance (Peters, Sutton, Jones, Abrams, & Rushton, 2008). Publication bias is only one possible reason for funnel plot asymmetry. Alternative explanations are true between-study heterogeneity or chance alone. Showing regions where studies reach (or do not reach) formal statistical significance helps to exclude such alternative explanations. If indeed publication bias leads to studies with insignificant results being less likely published, then this becomes visible with an implausible number of studies just reaching significance, while insignificant results – especially from small studies – seem to be missing. Conventionally, significance contours for two-sided (Wald) tests (at least) at the 10%, 5% (dark blue in the graph above), and 1% (white in the graph above) level are shown. Significance contours at the 5% level are identical to the 95% confidence regions in the classic funnel plot [2.1], but centered at the null effect, instead of the summary effect. | | Generally, additional evidence funnel plots were proposed to visualize the effect of one hypothetical additional study with a certain effect size and standard error on a meta-analytic estimate [2.1.6 – 2.1.11]. One of the first variants illustrates the effect on the statistical significance of the summary effect (Langan, Higgins, Gregory, & Sutton, 2012). Shaded regions are shown in which a new study has to fall, such that the meta-analytic summary effect either becomes statistically significant (in one direction or the other) or statistically insignificant (conventionally for a two-sided test at the 5% level). These plots might be especially informative, if the robustness of the summary effect is low. Usually, this is the case for rather small meta-analyses or rather ambiguous summery effects. For larger meta-analyses with not completely ambiguous effects, the shaded regions indicating change of the current result often do not contain plausible effect size and standard error values. This, in turn, would illustrate the robustness of the statistical significance of the meta-analytic summary effect. | | | | This variant shows the effect of a hypothetical additional study with a certain effect size and standard error on the estimated between-study variance (heterogeneity) in the meta-analysis (Langan, Higgins, Gregory, & Sutton, 2012). For this purpose, showing the effect on either *I*^2^ or on τ^2^ has been proposed. The contours for specified values of either τ^2^ or *I*^2^ form regions where new studies would have to fall, such that these heterogeneity levels are exceeded. This visualization allows assessing the robustness or sensitivity of the estimated between-study heterogeneity in a meta-analysis. The above plot was created within the Stata software, using the package extfunnel (Crowther, Langan, & Sutton, 2012). | | |  |
| Additional evidence contours funnel plot: Summary CI width [2.1.8] | | Additional evidence contours funnel plot: Summary CI lower/upper bound [2.1.9] | | | | Additional evidence contours funnel plot: Limits of equivalence [2.1.10] | | |  |
| 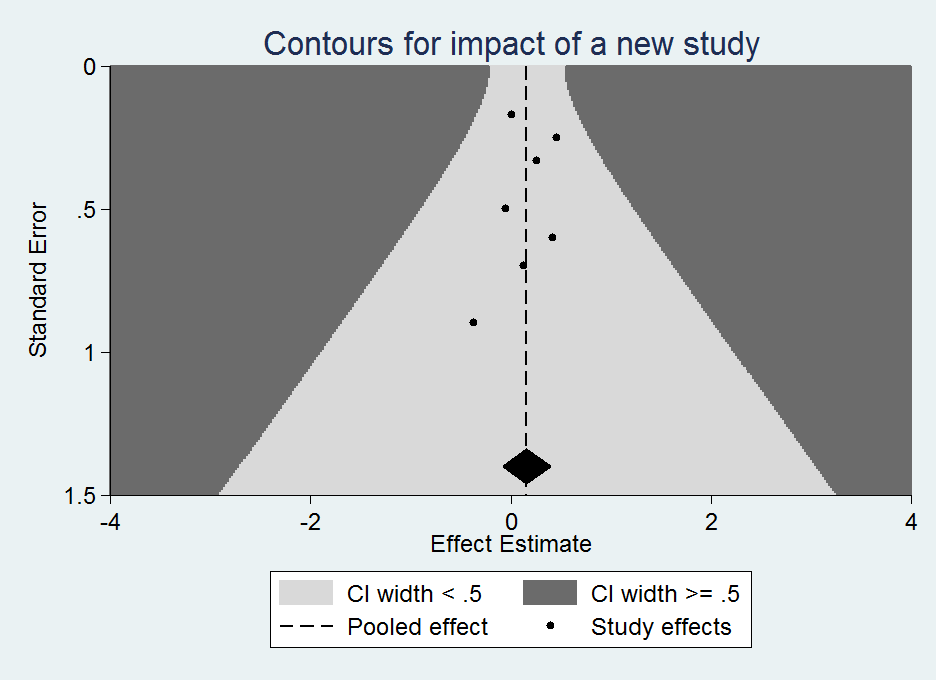 | | 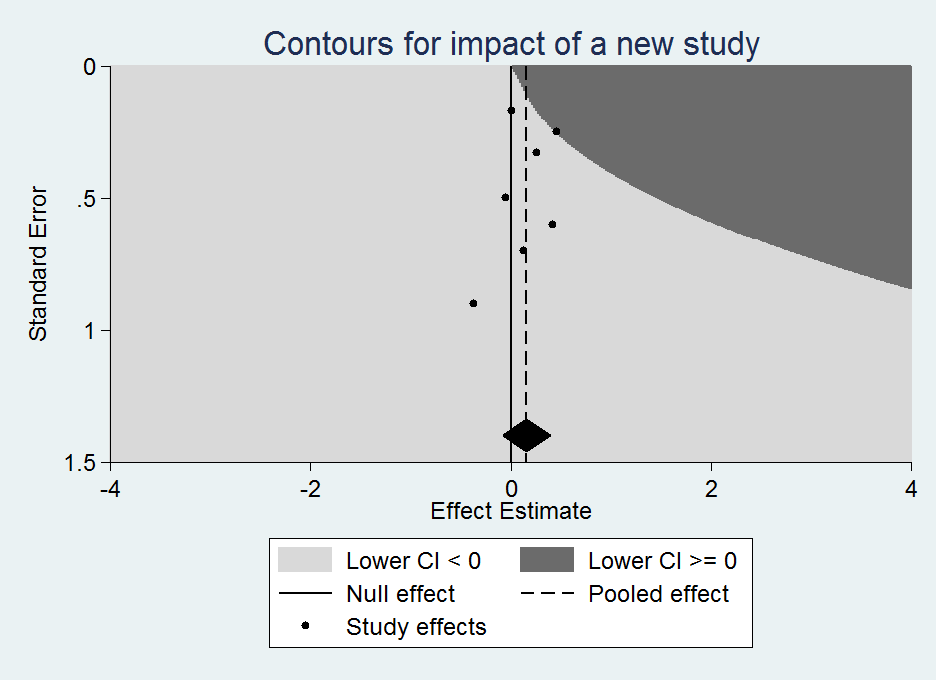 | | | | 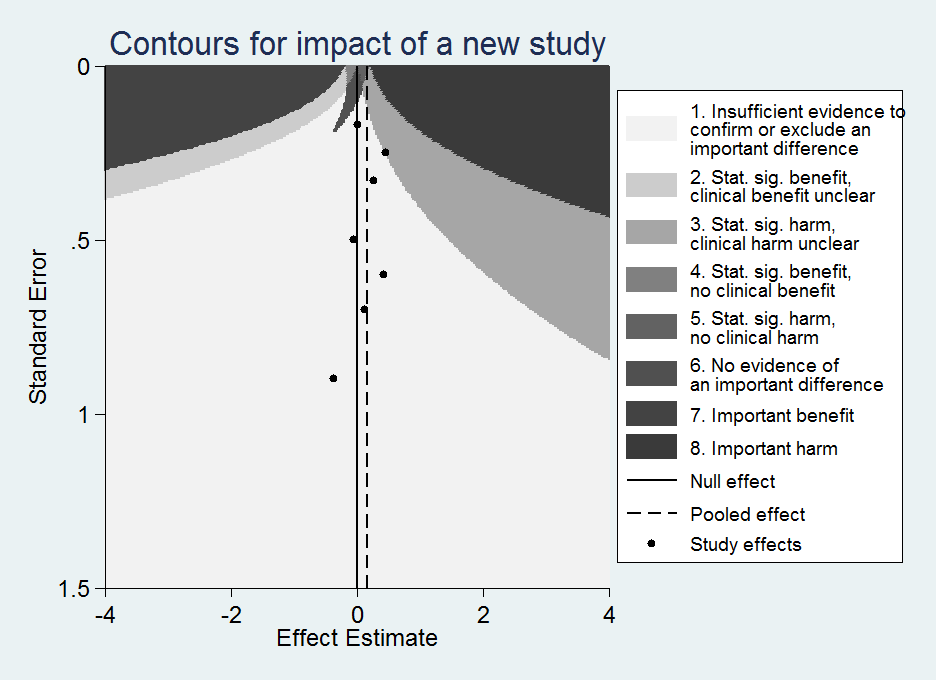 | | |  |
| The effect of a new study on the width of the confidence interval of the summary effect is shown. Shaded regions indicate areas where a new study with a certain effect size and standard error has to fall, such that the confidence interval width of the summary effect exceeds a certain user-specified level. This can be useful, if the robustness of the summary effect and its estimate of uncertainty is of interest. In contrast to [2.1.6], the focus here is on the robustness of the meta-analytic estimates directly, rather than on statistical significance alone. The above plot was created within the Stata software, using the package extfunnel (Crowther, Langan, & Sutton, 2012). | | Another variant of the additional evidence funnel plot visualizes the effect of a hypothetical new study on the upper or lower bound of the confidence interval of the summary effect. Shaded regions indicate where a new study has to fall, such that the lower or upper confidence interval limit of the summary effect exceeds a specified value. By comparing the lower (or upper) limit of the confidence interval to zero, the regions closely correspond to the significance regions in the summary effect significance contours funnel plot [2.1.6], but arbitrary values are possible. For instance, values of practical importance can be chosen, and it can be assessed whether the meta-analytic summary effect robustly and significantly exceeds this threshold. The above plot was created within the Stata software, using the package extfunnel (Crowther, Langan, & Sutton, 2012). | | | | The effect of a new hypothetical study on the summary effect is shown with respect to statistical significance and practical relevance using shaded regions. Limits of equivalence (LOE) are user-specified thresholds often used in the context of clinical research, illustrating effect magnitudes for which the treatment in question is regarded as beneficial (above the superiority LOE), or harmful (below the inferiority LOE), or equivalent compared to a standard therapy (see also LOE forest plot [1.2.5]). The summary effect can either be significantly different from zero or not. Moreover, it can be beneficial (sig. above the superiority LOE), harmful (significantly below the inferiority LOE), equivalent (sig. below the superiority LOE and sig. above the inferiority LOE), or ambiguous (not being sig. different from one or both LOEs). The robustness of the eight possible combinations are visualized for the meta-analytic summary effect (Sutton, Cooper, Jones, Lambert, Thompson, & Abrams, 2007). The above plot was created within the Stata software, using the package extfunnel (Crowther, Langan, & Sutton, 2012). | | |  |
| Additional evidence contours funnel plot: Summary effect [2.1.11] | | Funnel plot with imputed non-statistically-significant unreported effects [2.1.12] | | | Meta-analyser funnel plot [2.1.13] | | | |  |
| 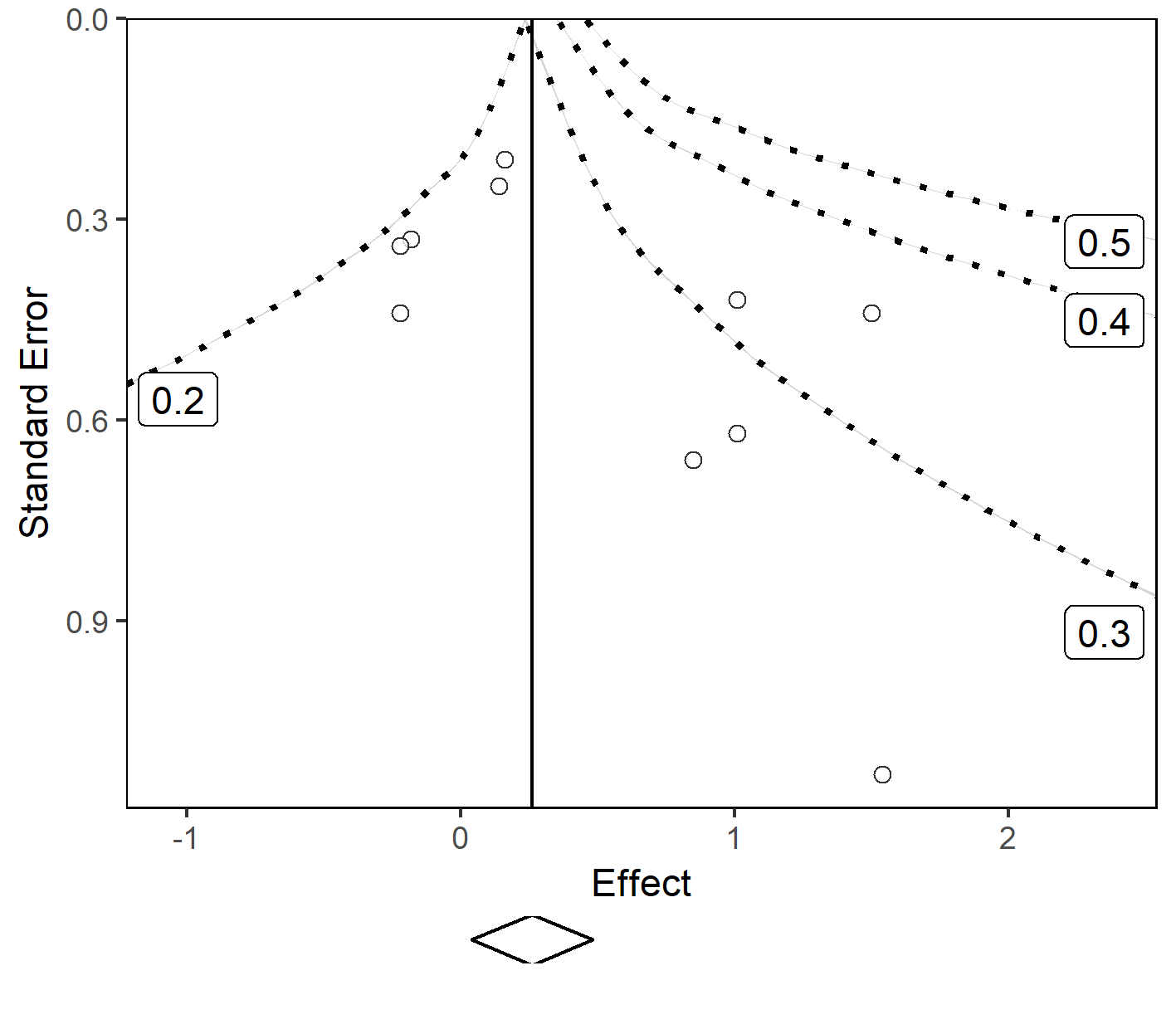 | | 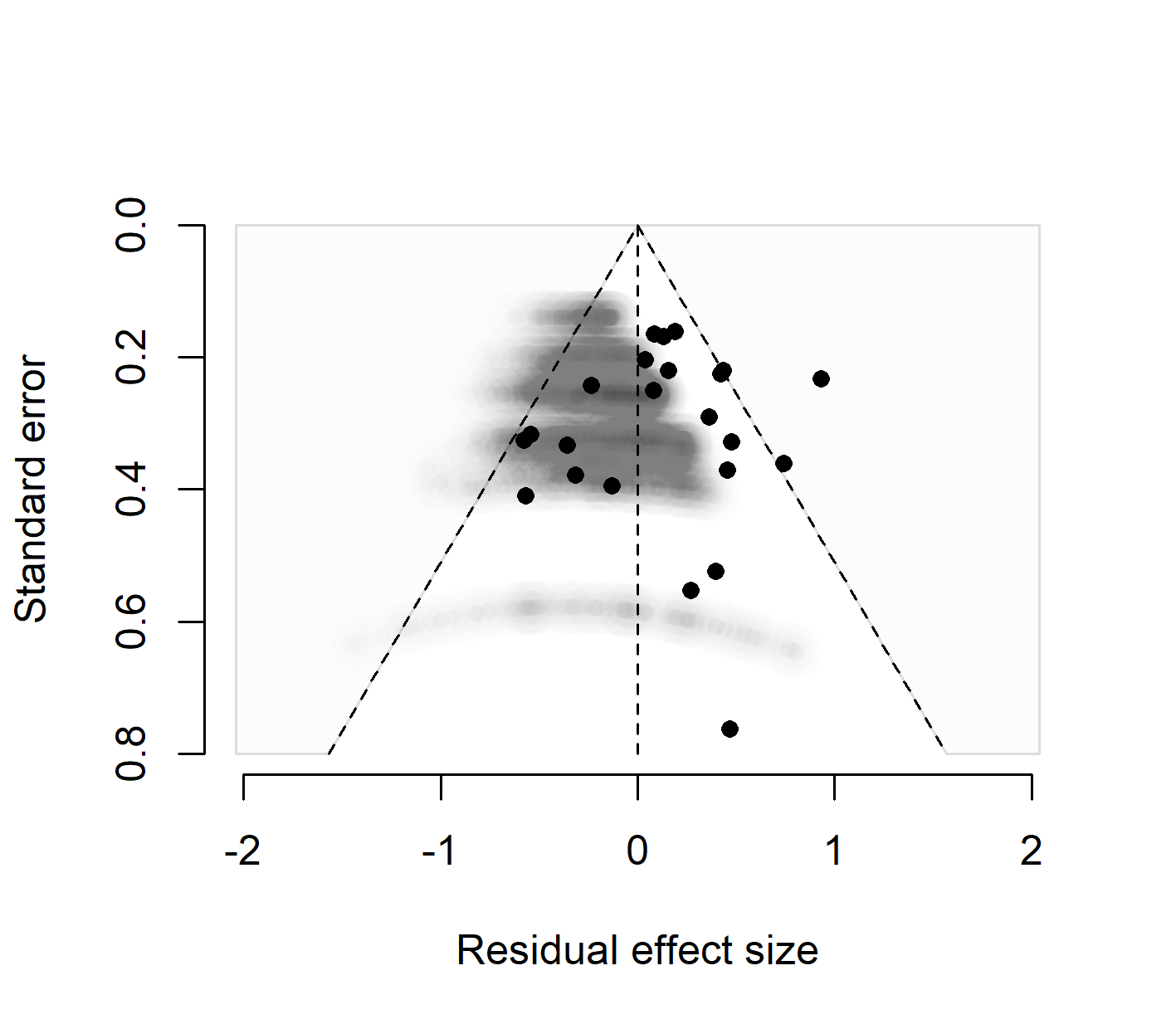 | | | 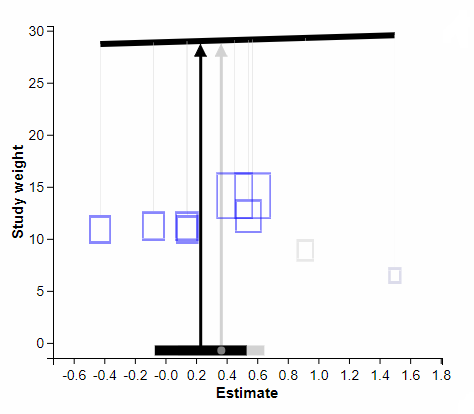 | | | |  |
| Which combination of effect size and standard error of a new study leads to a certain updated meta-analytic summary effect? To answer this question, a variant of additional evidence contour funnel plots has been proposed (Chevance, Schuster, Steele, Ternès, & Platt, 2015). For user-specified values of the meta-analytic summary statistic, the paths showing the respective combinations of effect size and standard error leading to these values are drawn. Proposed summary statistics are the summary effect, the *p* value of the summary effect, or the between-study heterogeneity parameter τ^2^. Like the other variants of additional evidence funnel plots, this plot can be useful for assessing the robustness or sensitivity of important meta-analytic summary results to a new study. | | This funnel plot variant is based on a novel method to impute non-statistically-significant unreported effects proposed and described by Radua et al. (2015). The funnel plot variant shows standard errors on the y axis, as well as a 95% confidence triangle - like the traditional funnel plot [2.1] - and effect sizes centered by the meta-analytic summary effect at the x axis. Shaded ellipses are plotted and approximately contain 95% of all non-statistically-significant unreported effects. Like the trim-and-fill funnel plot [2.1.4], the funnel plot with imputed non-statistically-significant unreported effects allows to examine which studies potentially are missing in the meta-analysis due to publication bias. Imputed non-statistically-significant unreported effects have also been visualized using forest plots (for further details, see Radua et al., 2015). The above example plot was created using the R package metansue (Radua, 2018). | | | The meta-analyser funnel plot is an interactive web application to visualize fixed-effect and random-effects meta-analysis as a physical system (Bowden & Jackson, 2016). This funnel plot-like display shows the effect sizes on the x axis and the meta-analytic weights (either absolute or relative) on the y axis.  The studies are visualized as literal weights, hanging down a scale. The meta-analytic summary effect is then the point for which the scale is in balance. Studies can be interactively removed and the new calibration of the scale (corresponding to an updated summary effect) observed. The plot allows an intuitive interpretation of meta-analytic weighting. It is therefore predestined for teaching the rationale behind classic meta-analytic models. Furthermore, it allows to interactively illustrate the effect of studies, study groups, and outliers in particular, on the summary effect. The above plot was created using R code provided with the source reference of the application (Bowden & Jackson, 2016). | | | |  |
| Funnel plot with summary diamond [2.1.14] | | Funnel plot with bias-corrected effect sizes [2.1.15] | | | |  |  |  |  |
| 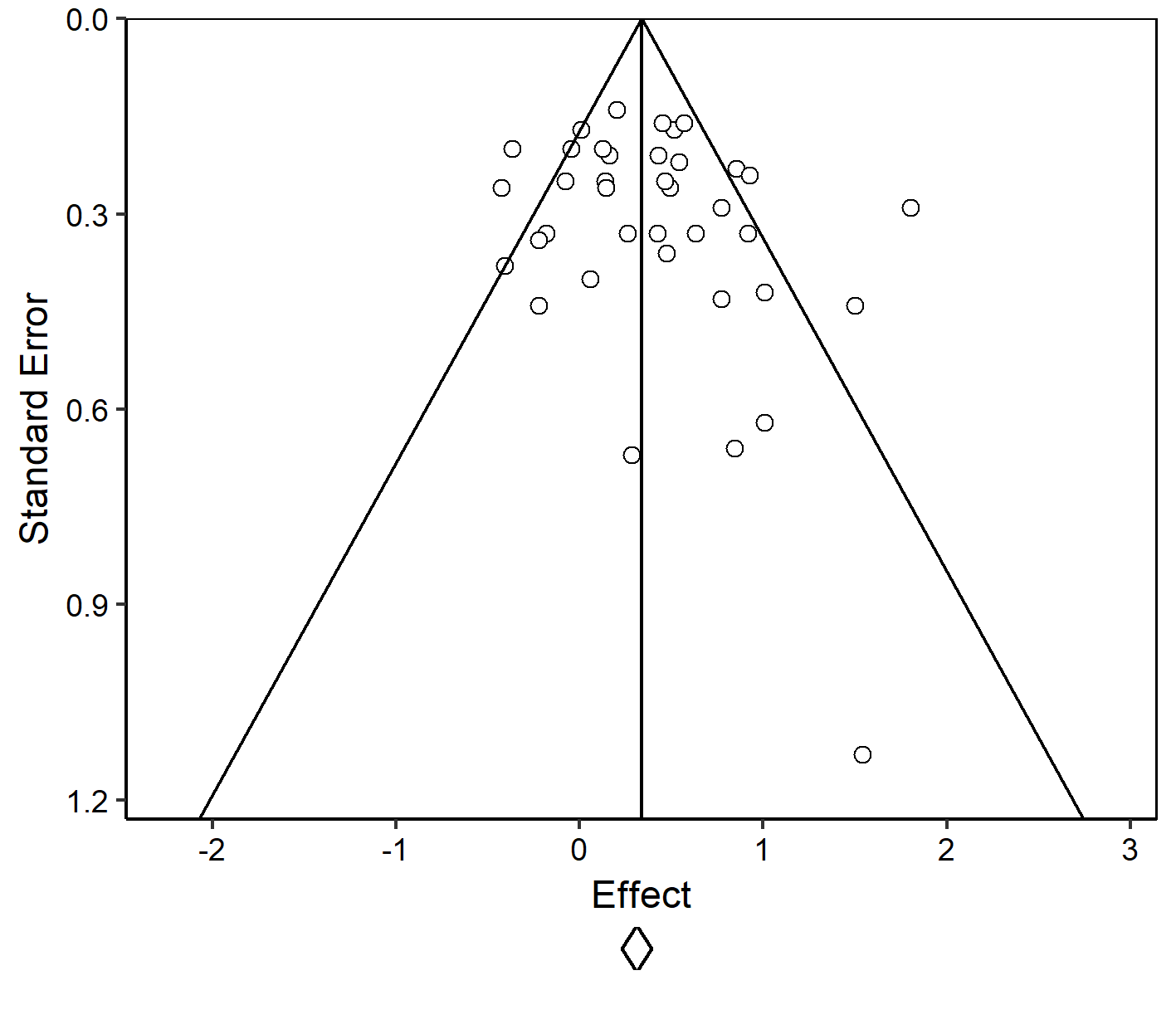 | | 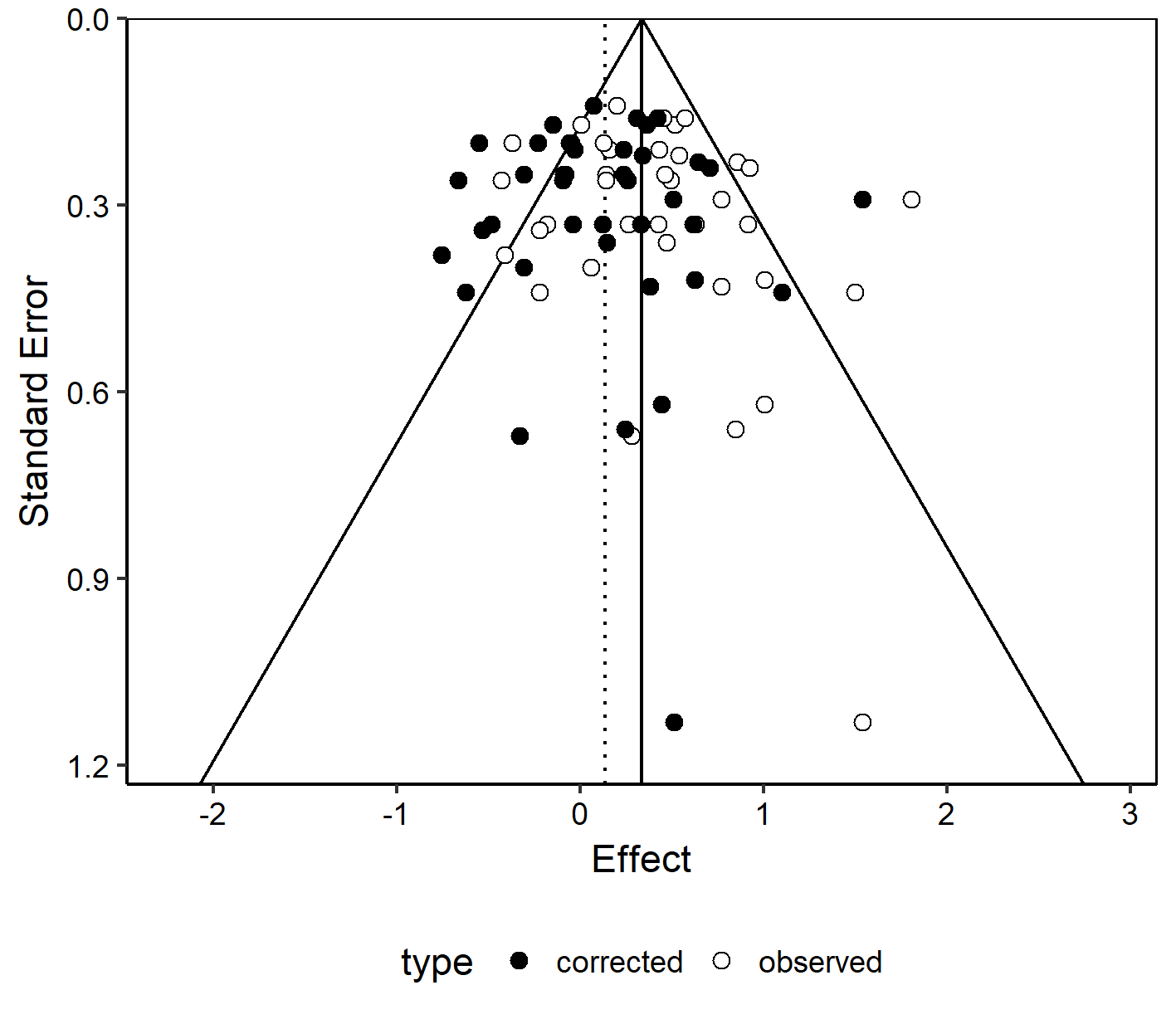 | | | |  |  |  |  |
| The funnel plot [2.1] traditionally does not show a confidence interval – or any other measure of statistical uncertainty – for the meta-analytic summary effect. The funnel plot with summary diamond has often been used to additionally show this information. The summary diamond showing the summary effect (mid-point) and confidence interval (width) has either been positioned below the funnel plot or, alternatively, within the funnel plot like an additional study, with the summary effect and the standard error of the summary effect as position in the funnel plot. For an example application, see Rothstein, Sutton, and Borenstein (2005, p. 79). | | Regression-test methods have been used to show bias-corrected effect sizes (see also [2.1.2]). For each study effect size, the estimated intercept from Egger’s regression test, times the standard error, is subtracted. These bias-corrected estimates are then shown in the funnel plot and allow to further examine the potential role of publication bias. In addition, the observed and corrected summary effects are indicated by a solid and dashed line, respectively. For further details and an example, see Roberts and Stanley (2005, pp. 26-32). | | | |  |  |  |  |
| Galbraith plot (a.k.a. radial plot) [2.2] | | | | Subgroup Galbraith plot [2.2.1] | | | | |  |
| 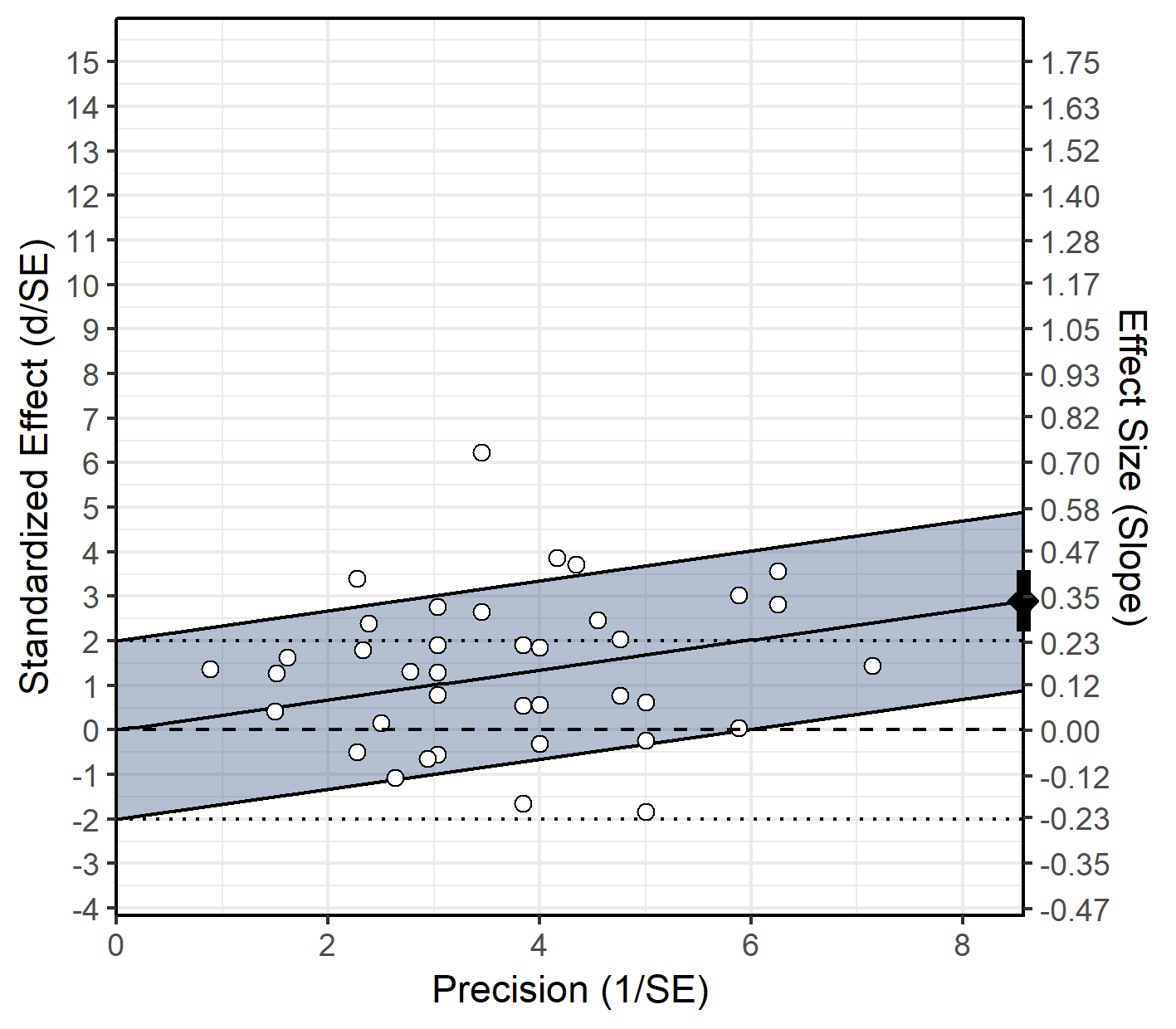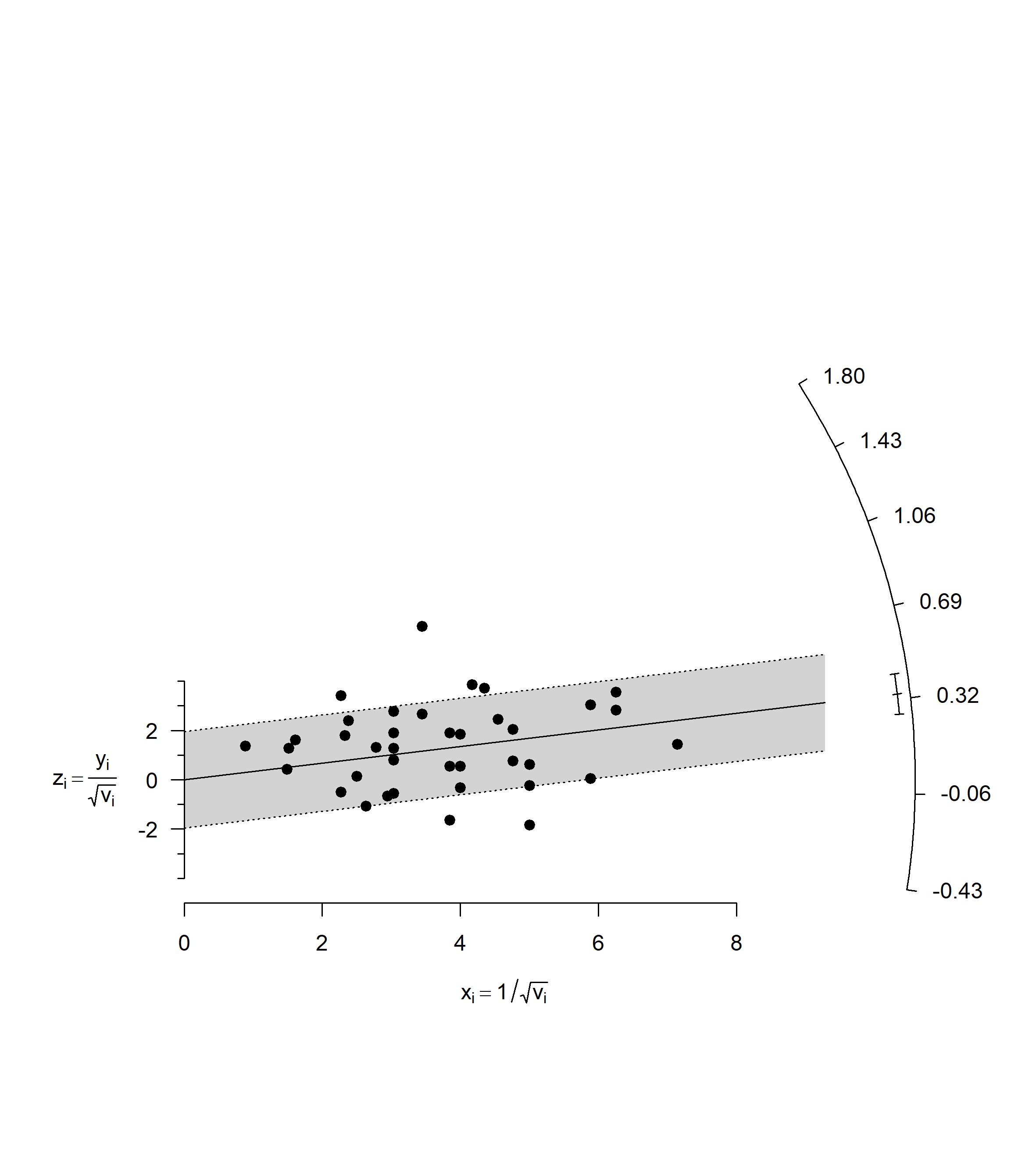 | | | | 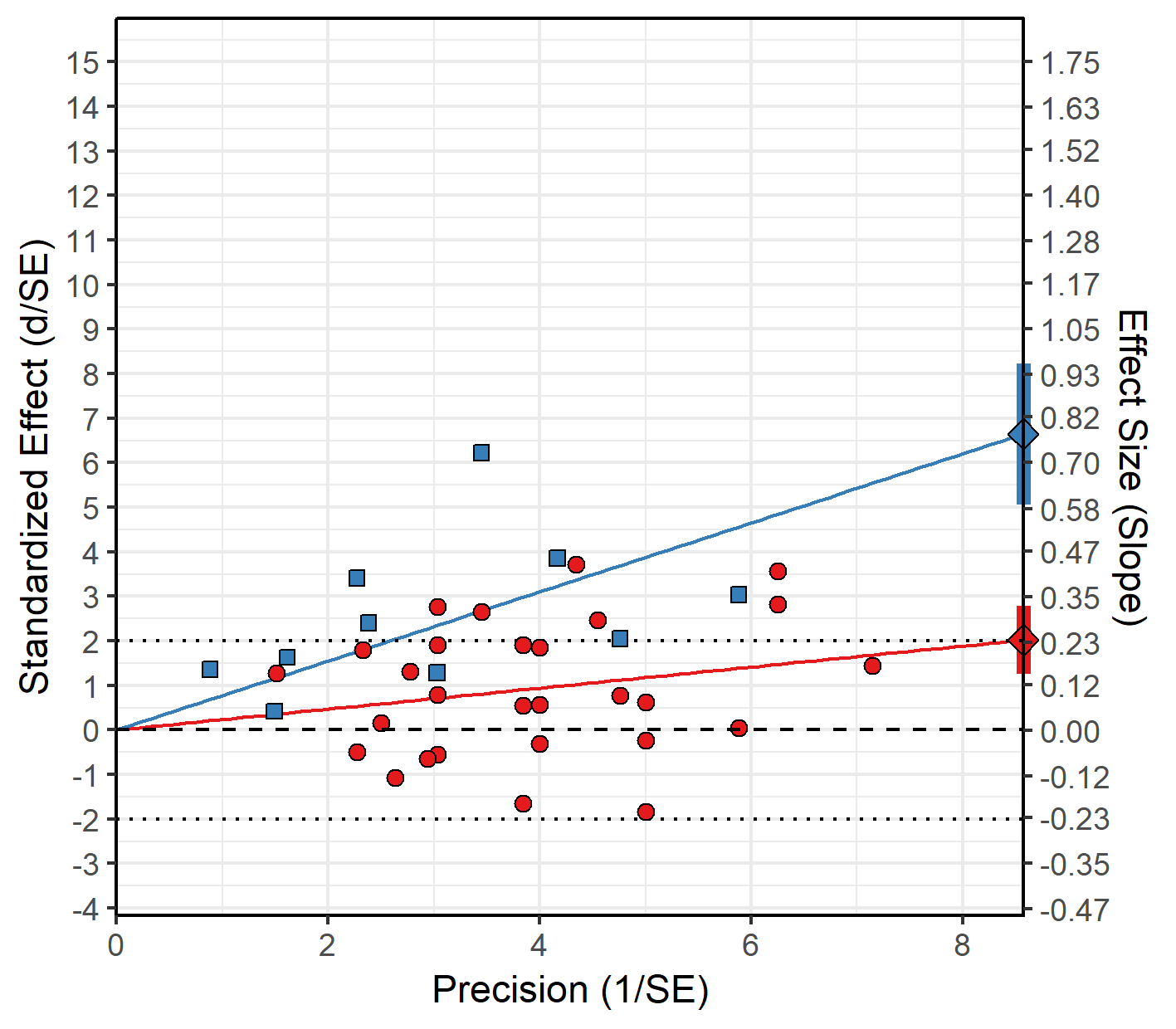 | | | | |  |
| The Galbraith plot or radial plot has been developed as an alternative to confidence interval plots [1.1] to present meta-analytic data (Galbraith, 1988). The Galbraith plot is closely related to the funnel plot [2.1] and essentially conveys the same information. Studies are shown in a scatterplot, with their precision on the x axis and the standardized effect sizes on the y axis. The main difference to the funnel plot [2.1] is therefore that effect sizes are divided by their standard error and are shown on the y axis, instead of on the x axis. The regression line in the Galbraith plot forced through the origin has a slope equal to the meta-analytic summary effect (in the fixed-effect model). Also, lines through the origin and any study point have a slope equal to the study effect sizes. A second axis can be shown on the right-hand side, allowing to read of these slopes of the summary effect line or any study-specific effect lines. This second axis typically, but not necessarily, is plotted as radial axis (as in the right example plot above) and includes the confidence interval of the meta-analytic summary effect. As in the funnel plot, confidence contours and significance contours (see also [2.1.5]) can be shown as parallel lines (plus and minus 1.96) around the summary effect line and the null effect line, respectively. Confidence contours show homogeneity regions, where roughly 95% of all studies are expected to fall (under the fixed-effect model). An unexpectedly large number of studies lying outside these contours is suggestive for heterogeneity. Single studies lying far outside the confidence contours might be regarded as outliers. Studies lying outside the significance contours observed statistically significant effects (using a two-sided Wald test with a significance level of 5%). The example radial plot above on the right-hand side was created using the R package metafor (Viechtbauer, 2010). | | | | Study subgroups of interest can be readily depicted in a Galbraith plot by using different plotting symbols or colors (Galbraith, 1988). This might help to explain between-study heterogeneity by categorical study features. The estimated summary effect for each group can be depicted by (the slope of) dedicated lines of fit. If the depicted study feature explains between-study heterogeneity, the summary lines should differ in their slopes and studies within a subgroup should scatter symmetrically around the subgroup’s corresponding summary line. By utilizing horizontal lines of statistical significance thresholds, systematic differences in statistical significance between studies of different subgroups can be examined. | | | | |  |
| Galbraith plot with Egger regression [2.2.2] | 3D Galbraith plot [2.2.3] | | | Gravity plot [2.3] | | | | |  |
| 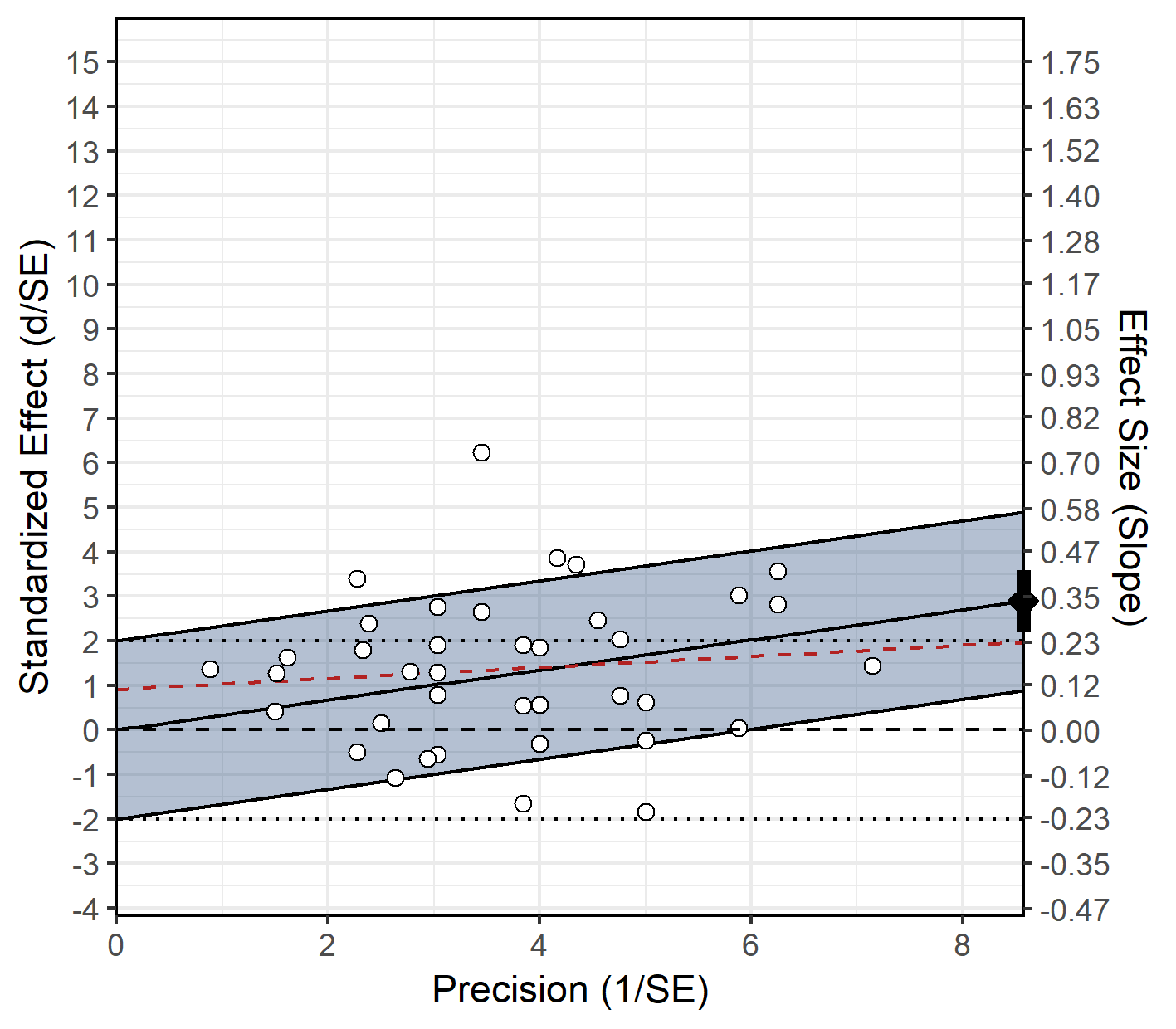 | 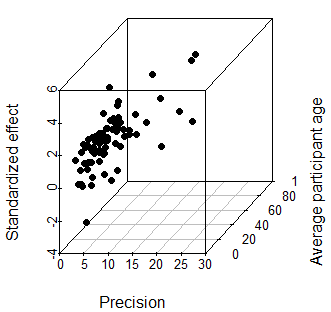 | | | 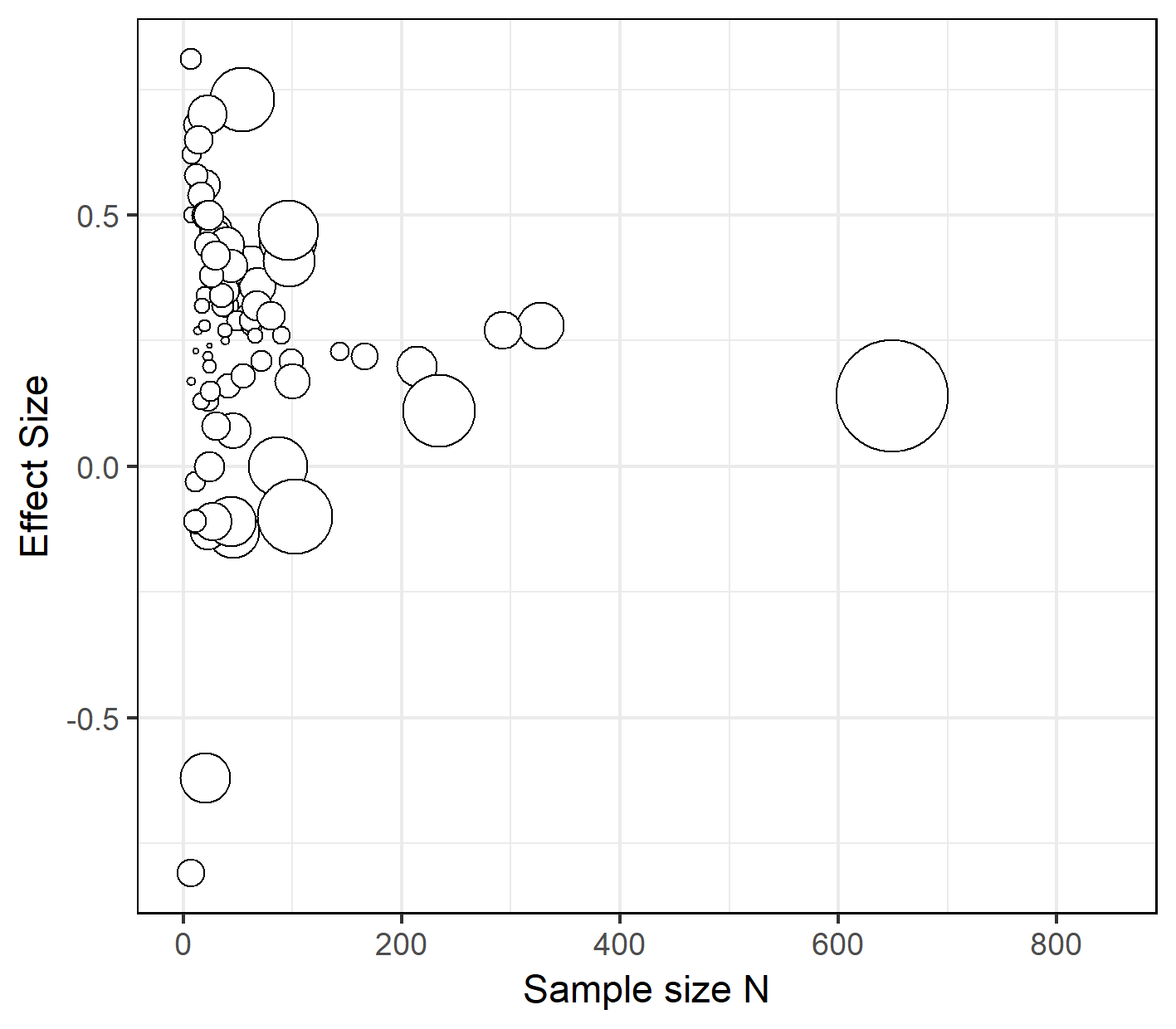 | | | | |  |
| Egger’s regression (Egger, Smith, Schneider, & Minder, 1997) is the ordinary least-squares regression of the standardized effect size (effect size divided by the standard error) on precision (1 divided by the standard error). The line of fit of this regression can be readily depicted within the Galbraith plot. Egger’s regression test is based on the intercept of this regression line. Small-study effects (i.e., funnel plot asymmetry) lead to an intercept of this line unequal zero. If studies with large standard errors tend to report larger positive effect sizes, this leads to an intercept larger than zero, whereas if studies with larger standard errors tend to report larger negative effect sizes, this leads to an intercept smaller than zero. The slope of the Egger’s line of fit has been suggested as a possible publication bias corrected meta-analytic summary estimate (see also [2.1.2]). | Extending Galbraith plots [2.2] to a third dimension has been proposed to incorporate information on a continuous study covariate (Laroche, 2015). While the subgroup Galbraith plot [2.2.1] allows incorporating a discrete study covariate in a two-dimensional display, a continuous effect moderator can be shown on a third axis in three dimensions. An example for the use of a 3D version of the Galbraith plot can be found in Laroche (2015, p. 147). | | | The influence of a single study on the magnitude of the estimated summary effect depends simultaneously on (1) the sample size of the study, because larger studies (with smaller standard errors) have more meta-analytic weight; and (2) on the unusualness of the observed effect size. Gee (2005) termed the combined influence of a study’s effect and a study’s size on the estimated summary effect as gravity. The gravity of a specific study is quantified with leave-one-out methods by computing the change of the summary effect, if this particular study is not considered in the estimation of the summary effect. The gravity plot (Gee, 2005) is a scatterplot with the sample size on the x axis, the effect size on the y axis, and plotting symbols with size proportional to each studies gravity. Therefore, it visualizes the combined effect of effect size and sample size on the estimated summary effect. Typically, studies near the summary effect possess low gravity, unless they have very large weights. However, for unusually large or small effect sizes, even small studies can have a surprisingly large gravity. | | | | |  |
| Doi plot [2.4] | | | Begg and Mazmudar test display [2.5] | | | | Begg and Mazmudar test display with subgroups [2.5.1] | | |
|  | | | 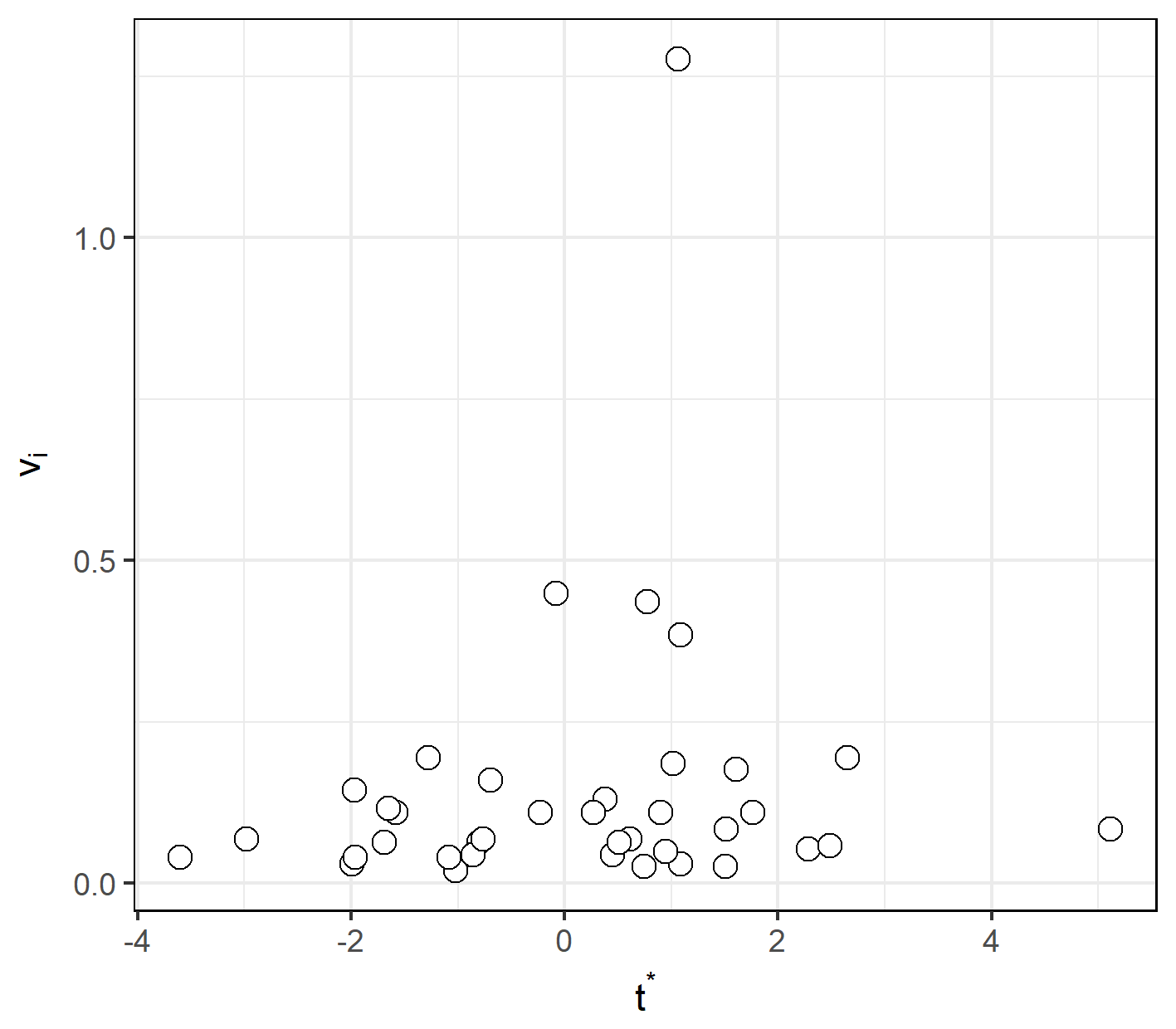 | | | | 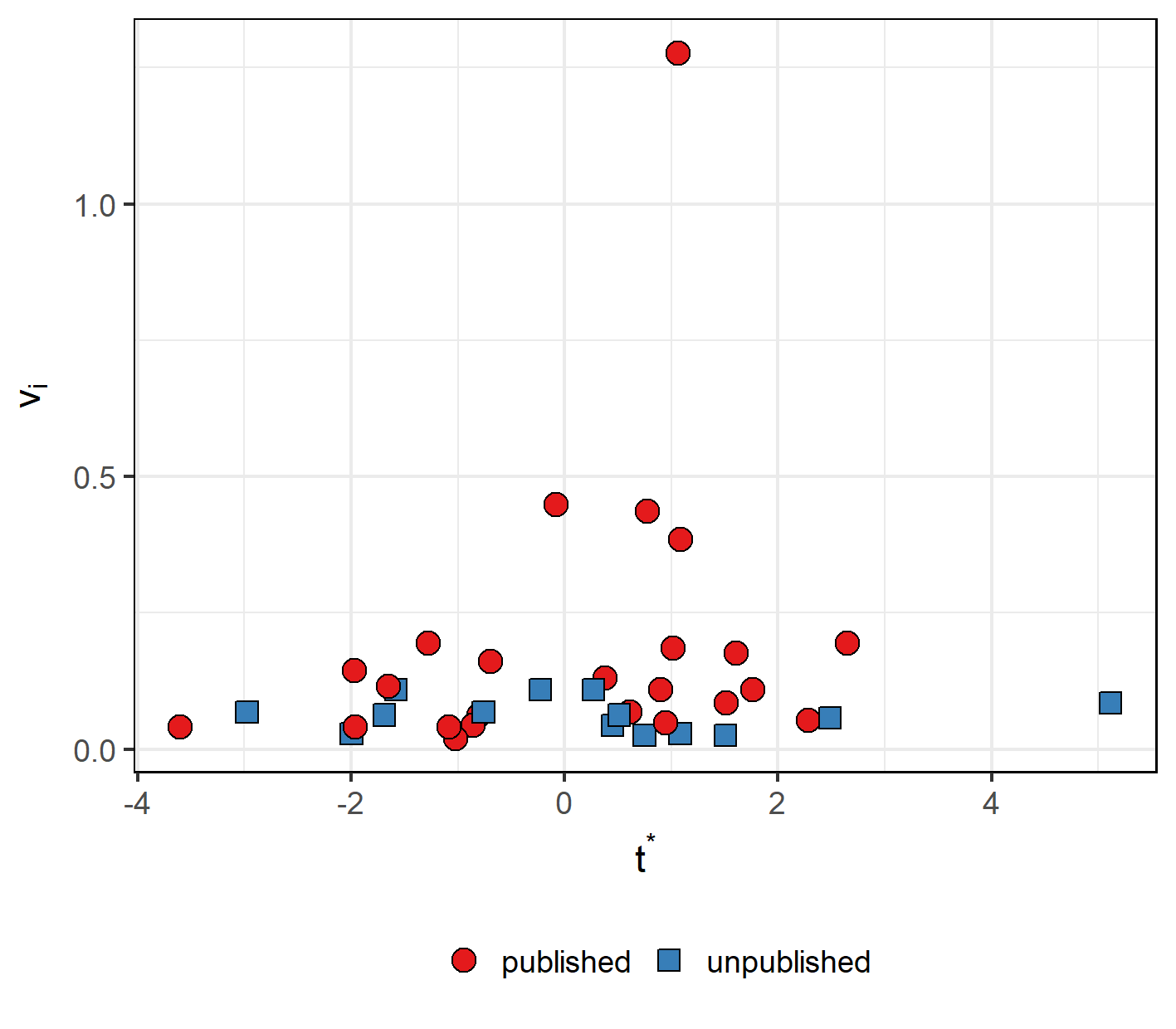 | | |
| The doi plot (Barendregt & Doi, 2016) was proposed as an alternative to the funnel plot [2.1] to examine small-study effects, i.e., an association of standard error with effect size. The doi plot displays the effect size on the x axis and a Z-score on the y axis. The Z-score is based on ranks of the sum of (a function of) the standard errors of all studies with effect sizes up to a certain value (for a detailed elaboration, see Barendregt & Doi, 2016). For a set of symmetric studies, the most precise studies should be at top of the doi plot, with a Z-score close to zero. For studies with less precision, the Z-score should symmetrically decreases on either side of the peak. Therefore, a similar number of studies on each side of the peak and symmetric, equally spread arms on both sides are expected. Violations of this expected symmetric mountain shape indicates asymmetry (i.e., small-study effects). An index has been developed and applied to objectively quantify the extent of asymmetry in the doi plot (Furuya-Kanamori & Doi, 2016). The plot above was created with the addon MetaXL for Excel (Barendregt & Doi, 2016). | | | The Begg and Mazumdar test for publication bias (Begg & Mazumdar, 1994) is a non-parametric test for funnel plot asymmetry. It is based on the rank correlation (Kendall’s tau) between the standardized centered effect sizes and the corresponding variances of the effect sizes. The Begg and Mazmudar test display shows these two quantities in a scatterplot and therefore depicts the tested association visually. The plot might help to examine and understand the reasons for specific test results. | | | | In the Begg and Mazmudar test display, different study subgroups can be readily depicted by different plotting symbols or colors. This can aid in the assessment of whether test results of the Begg and Mazumdar test for publication bias (Begg & Mazumdar, 1994) might be driven by certain study subgroups. Subgroups of interest in this context can be, for instance, significant vs. insignificant results, or published vs. unpublished studies (see also subgroup funnel plot [2.1.1]). | | |

| Scatterplot of effect size with continuous moderator [3.1] | Binned scatterplot [3.1.1] | Meta-regression plot [3.2] |
| --- | --- | --- |
| 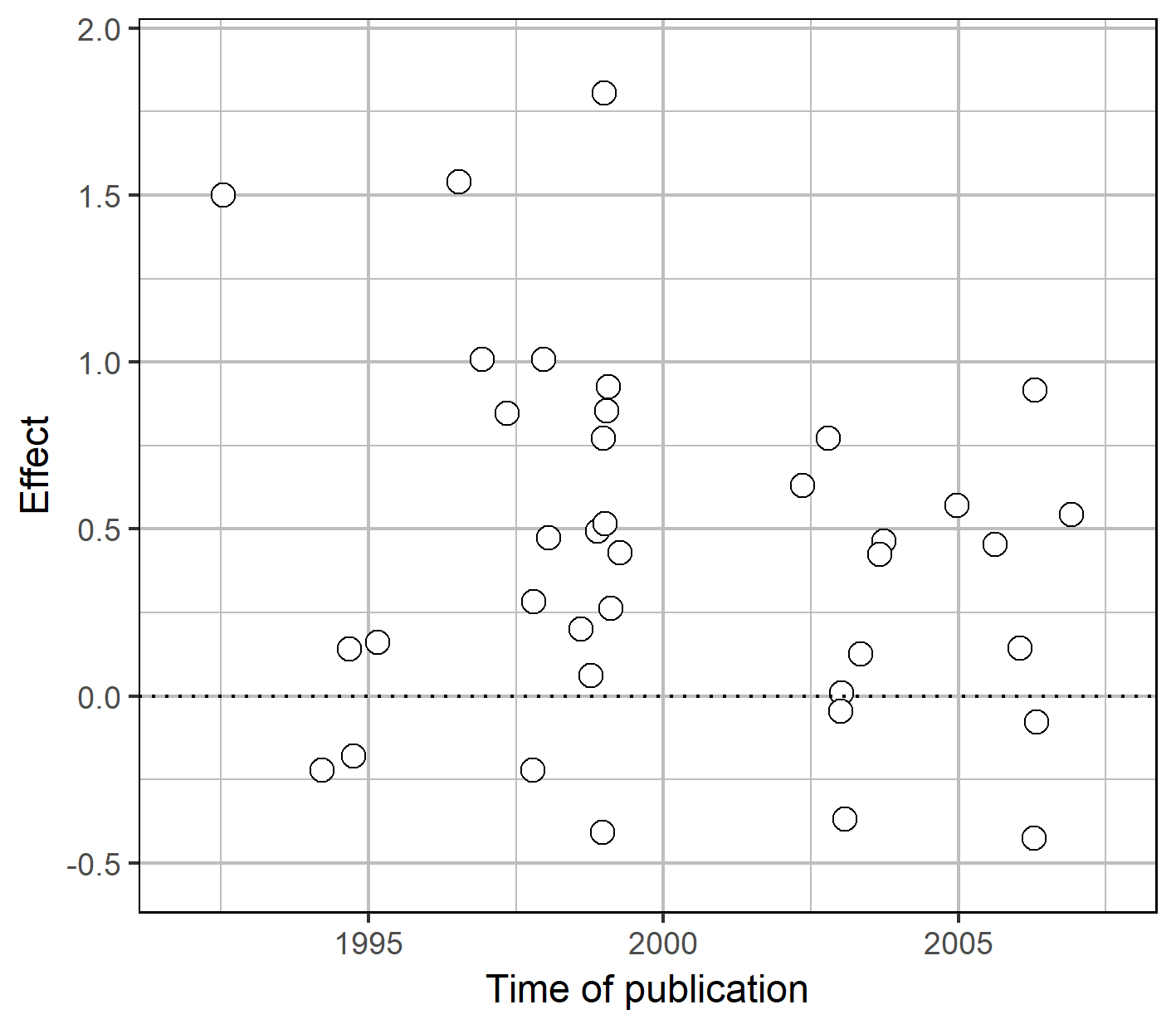 | 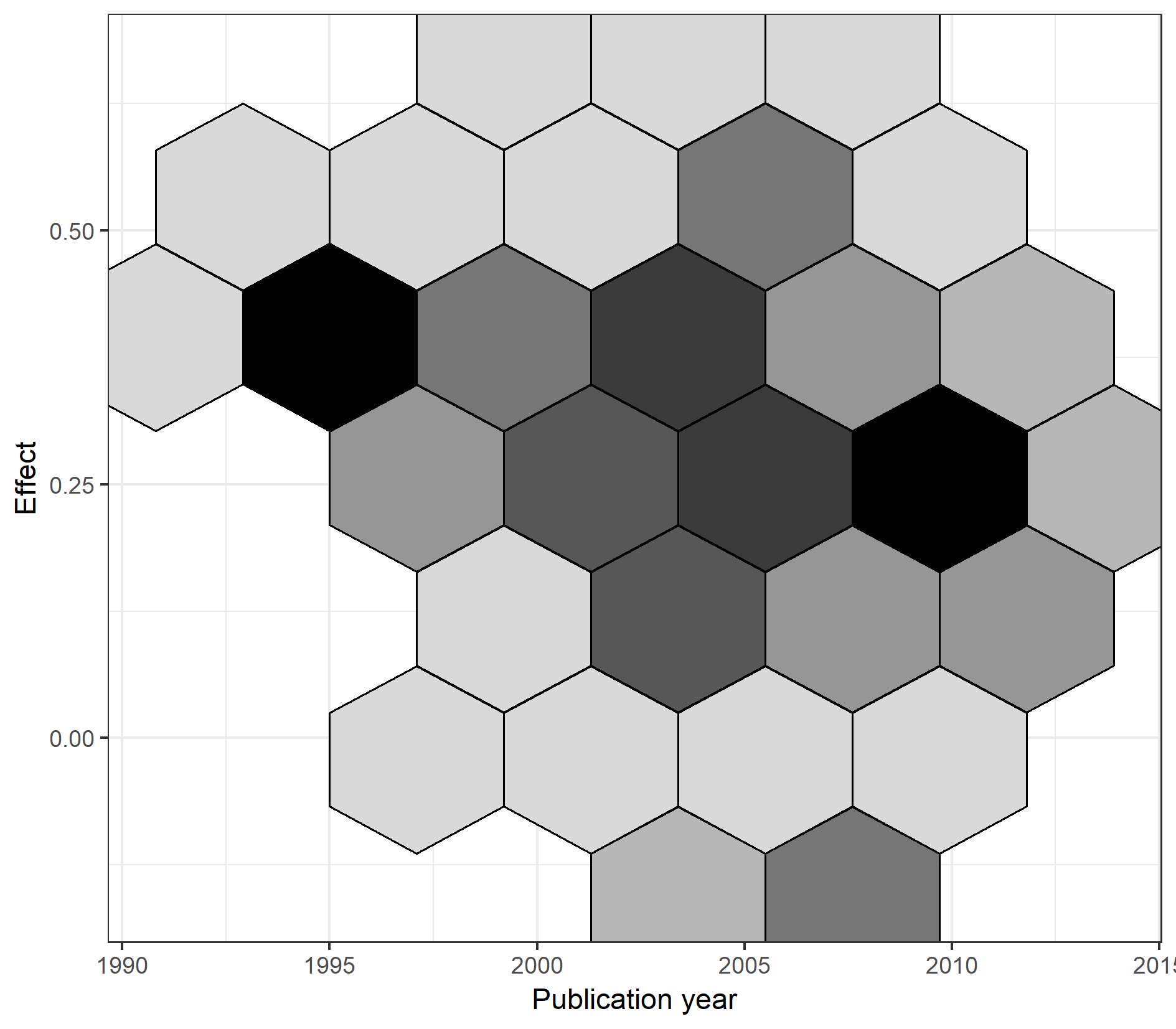 | 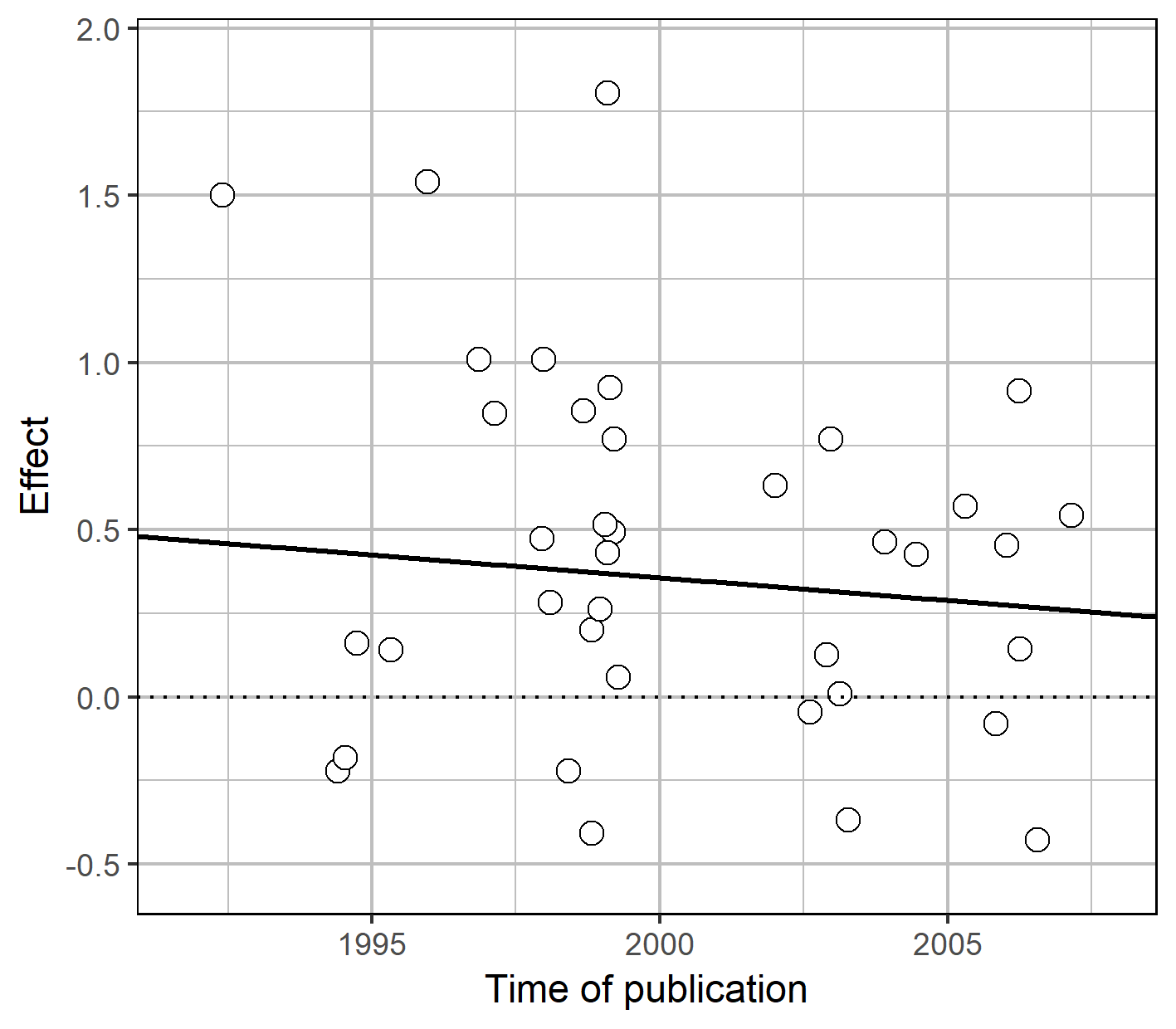 |
| Scatterplots to visualize any associations of study effect sizes with study covariates were one of the first statistical displays used for meta-analysis (Glass, 1977). Such scatterplots are regularly used to explore between-study heterogeneity explainable by a continuous effect moderator (e.g., publication year, drug dose, or mean age of study participants). Many genuine plots developed for meta-analysis are specialized versions of scatterplots of effect sizes and other continuous statistics on the study-level (e.g., the funnel plot [2.1], Galbraith plot [2.2], or L’Abbé plot [8.1]). | Binned scatterplots have been proposed in the context of meta-analysis of many studies. Binned scatterplots do not show each study, as in conventional scatterplots [3.1], but rather divide the scatterplot in equally sized regions (i.e., two‑dimensional bins). Shading can be used to show the number of studies falling within each bin. Alternatively, sunflower plots have been proposed, using different flower-like symbols with a varying number of petals, instead of shading, to indicate the number of studies within a bin. For an example, see Schulze, Holling, and Böhning (2003, pp. 213-215). | The estimated effect-size mean, conditional on the covariate of interest, has been visualized with meta-regression lines (Glass, 1977). The regression line additionally aids in the assessment of the association of effect sizes with the covariate. The linear or non-linear meta-regression line of fit can be estimated by any appropriate parametric or non-parametric meta-analytic model. |
| Meta-regression weight bubble plot [3.2.1] | Meta-regression plot with subgroups [3.2.2] | Meta-regression plot with confidence or prediction bands [3.2.3] |
| 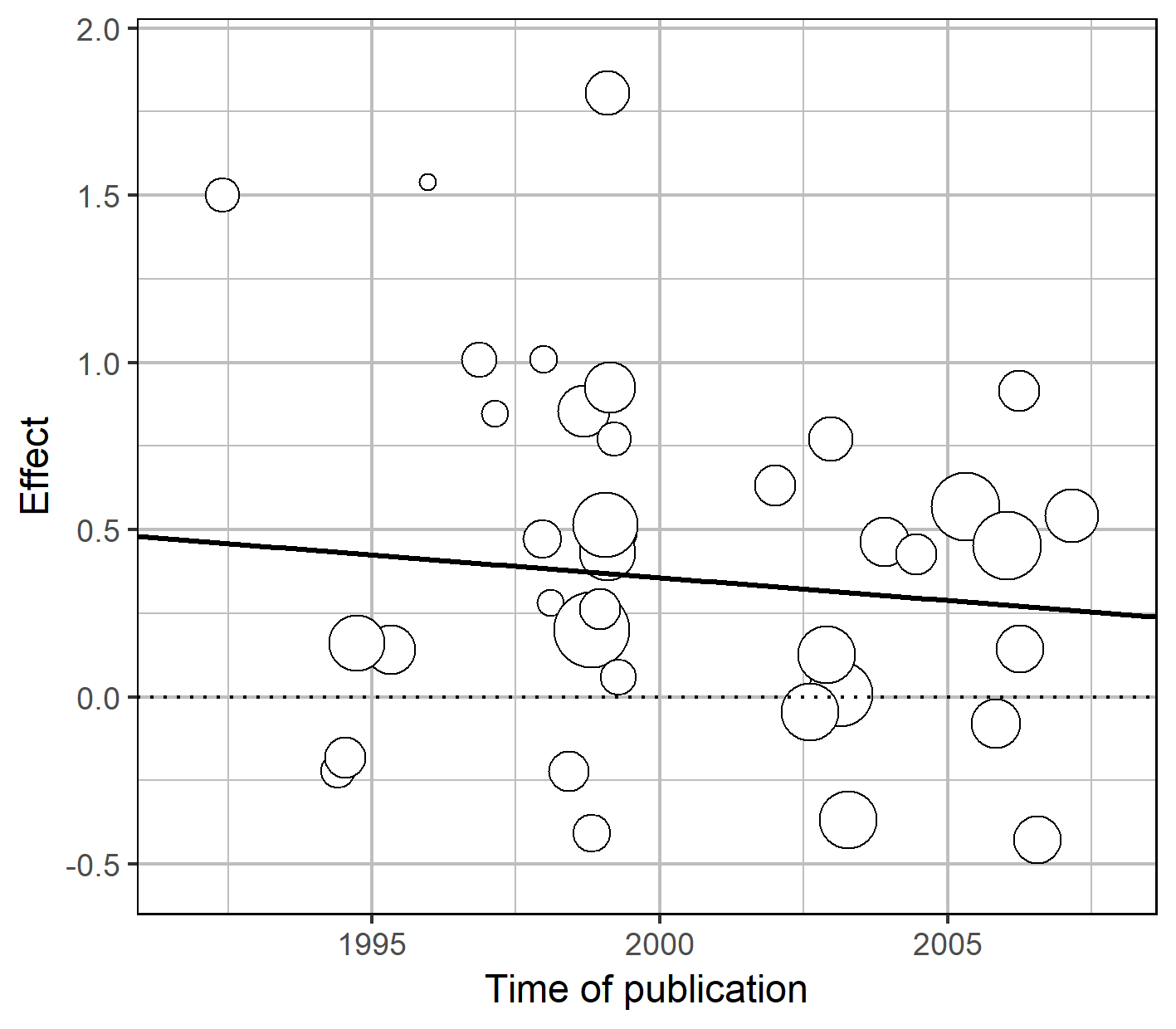 | 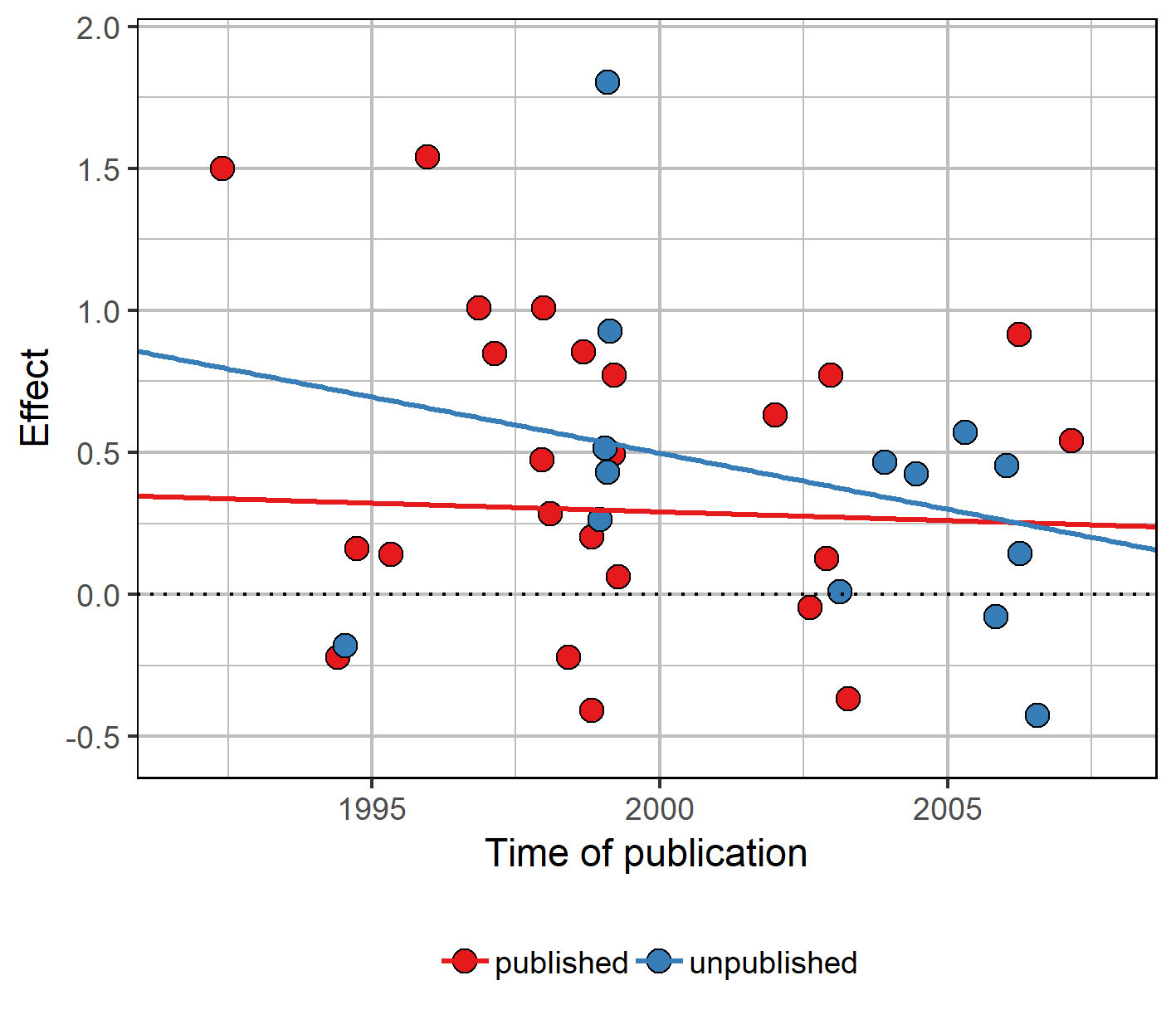 | 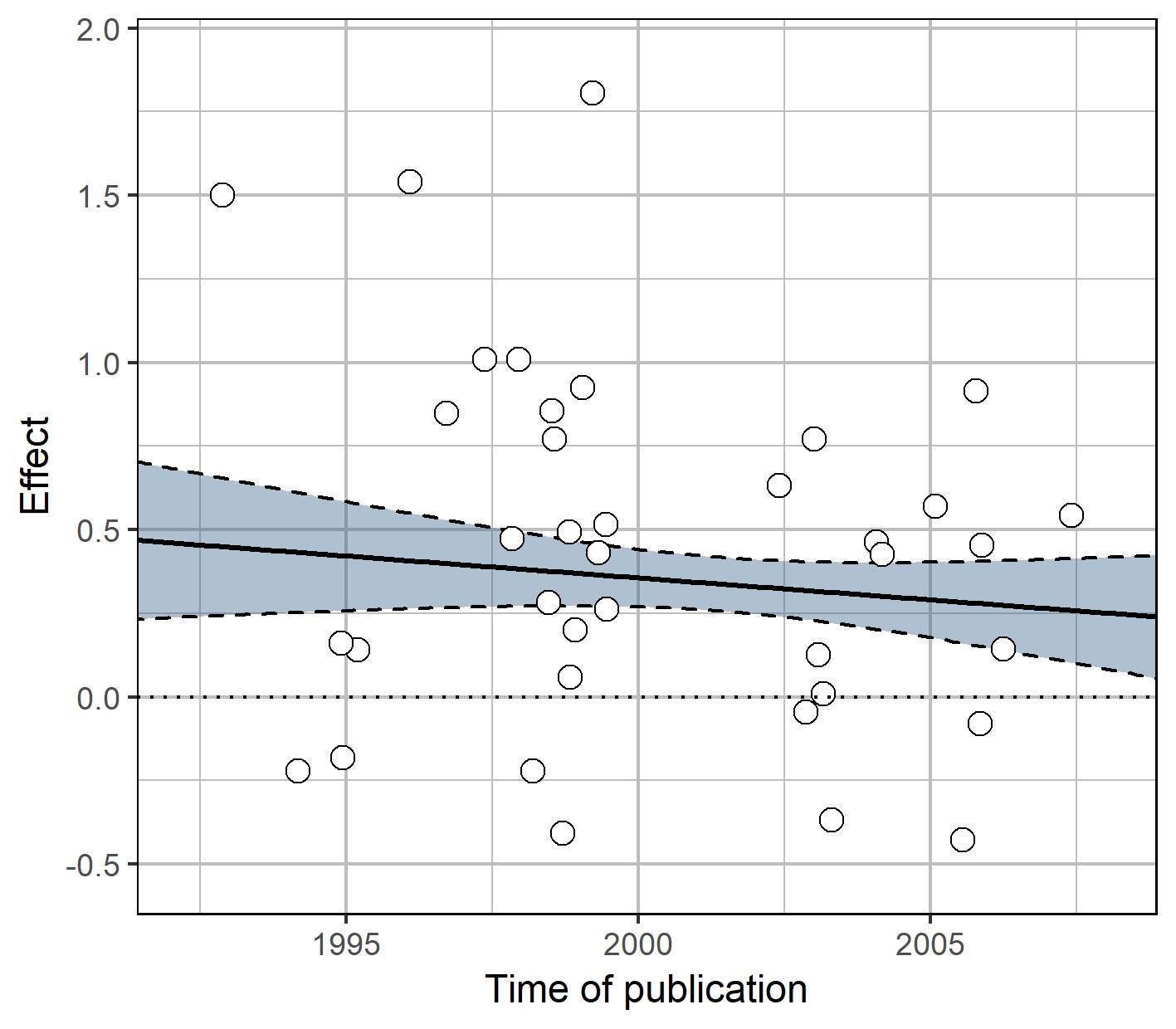 |
| The meta-regression weight bubble plot has been used to show information on the meta-analytic weight per study in the scatterplot. Each study is shown with a bubble proportional in size to its meta-analytic weight (inversely related to the studies variance). Therefore, more precise effect estimates are shown with larger bubbles than imprecise estimates. Classic meta-regression models incorporate the study weights into the estimation of the line of fit (weighted least-squares regression), such that more precise studies are also more influential than less precise studies. Thus, the weight bubble plot has been especially used to visualize such meta-regression models, when the number of studies is not very large. For an example, see Sutton, Abrams, Jones, Sheldon, and Song (2000, pp. 97, 104). | Study subgroup information can be readily incorporated in meta-regression plots ([3.2], [3.2.1]). For this purpose, subgroup specific meta-regression lines are estimated and shown, using different colors or line types. For an example, see Koricheva, Gurevitch, and Mengersen (2013, p. 346). | Within meta-regression plots [3.2], the uncertainty in the estimated meta-regression line has been visualized. Confidence bands (pointwise) show confidence (or credibility) intervals for the meta-analytic summary effect, given a specific covariate value. Alternatively, prediction bands can be shown, to visualize a probable effect size region for a new study, given a covariate value. For an example, see Sterne (2009, p. 86). |

| Meta-regression plot including effect-size confidence intervals [3.2.4] | Surface plot [3.2.5] | Trim-and-fill meta-regression plot [3.2.6] |
| --- | --- | --- |
| 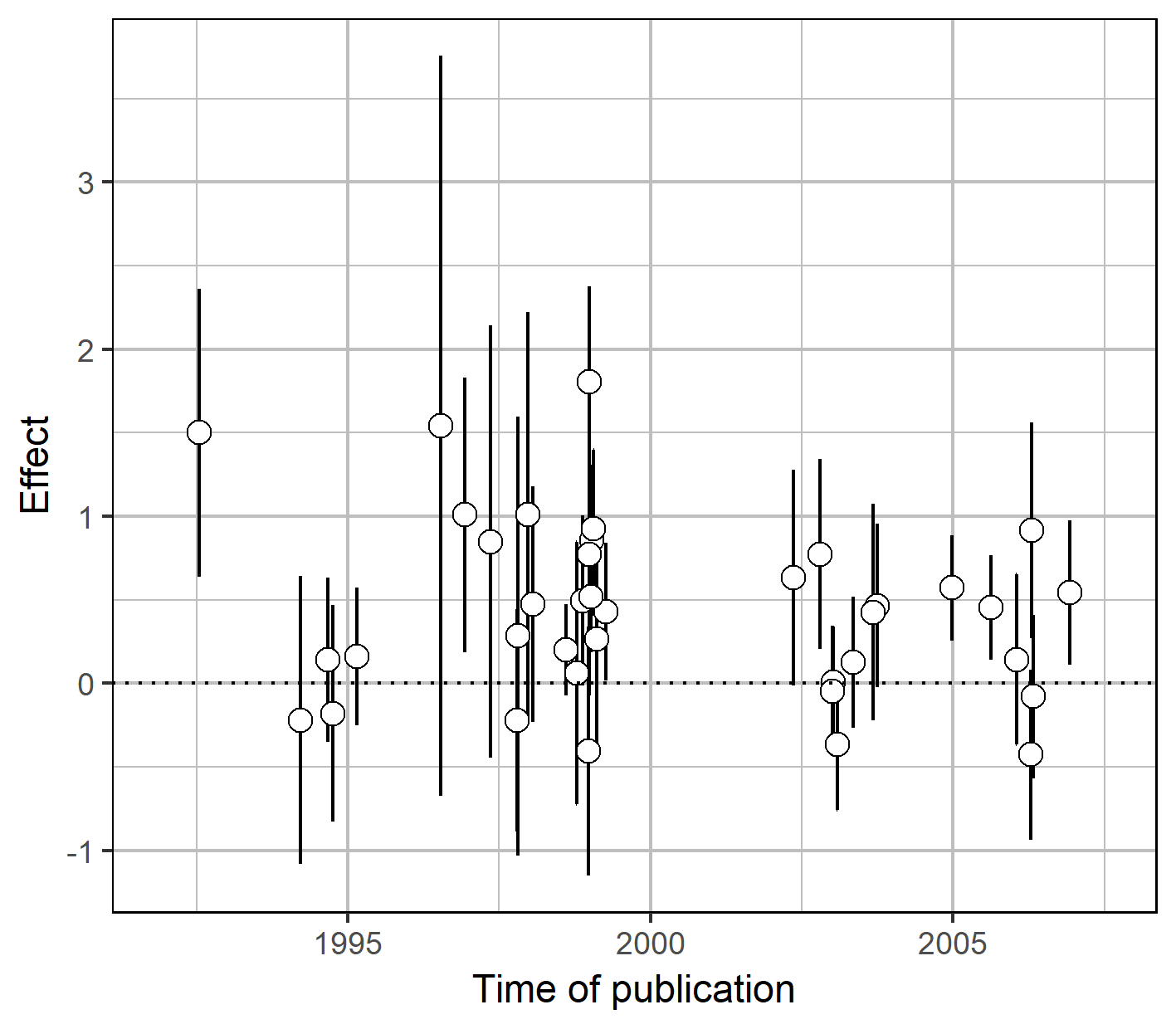 | 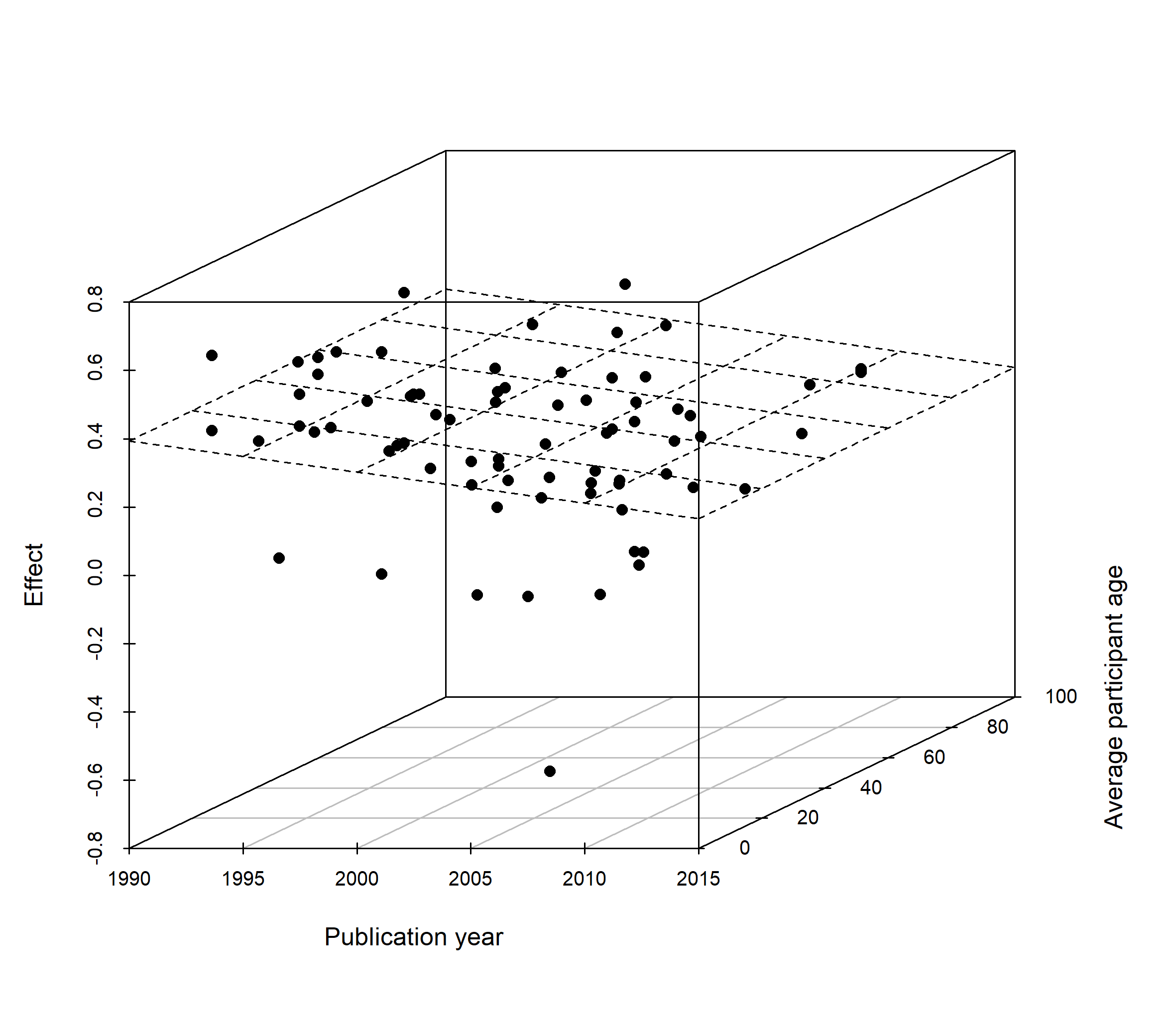 | 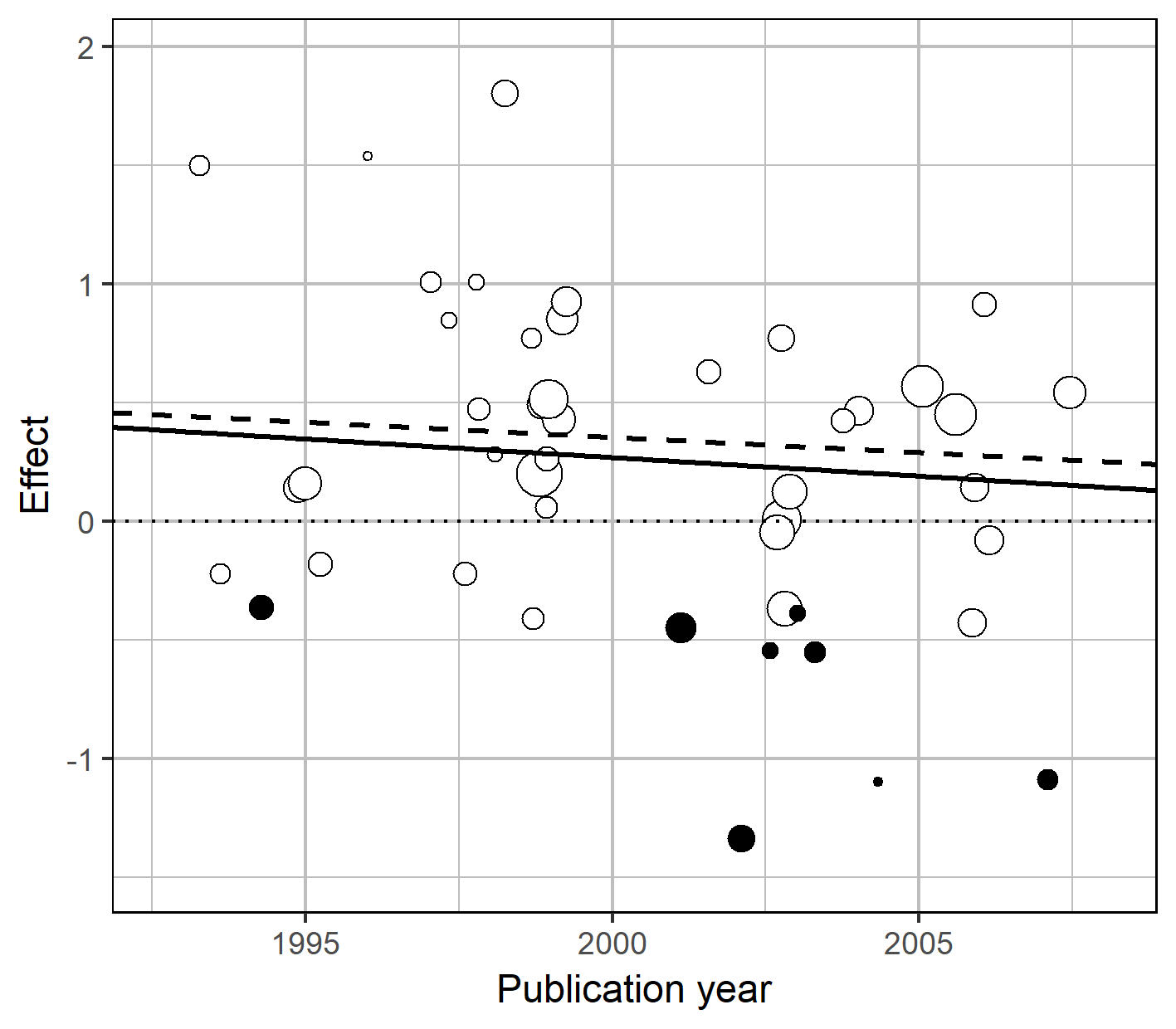 |
| Scatterplots have been used to also show study confidence intervals (Light & Pillemer, 1984, p. 73). They allow incorporating information on study precision by considering the length of study confidence intervals. In addition, not only the association of effect size with a study covariate, but also with study precision, can be examined. For large-sized meta-analyses with many included studies, this plot can get crowded quickly, similar to the related weight bubble plot [3.2.1]. An alternative is a forest plot [1.2], with studies ordered with respect to the covariate of interest. | A three-dimensional surface display has been proposed to examine the association of study effect sizes with two study covariates simultaneously (Lau, Ioannidis, & Schmid, 1998). Points within the three-dimensional space show each observed study effect size and covariate values. An overlain surface shows the estimates fitted values by a meta-regression model, given the two covariates. | An extension of the trim-and-fill method (Duval & Tweedie, 2000; see also [2.1.4]) to meta-regression has been proposed (Weinhandl & Duval, 2012). The trim-and-fill meta-regression plot shows the estimated missing studies due to publication bias and the adjusted meta-regression line of fit. The plot allows assessing the role of potential publication bias in the context of meta-regression. |
| Meta-analytic regression/classification tree [3.2.7] | Meta-analytic partial dependence plot [3.2.8] | Time-to-event data: Meta-STEPP [3.3] |
| 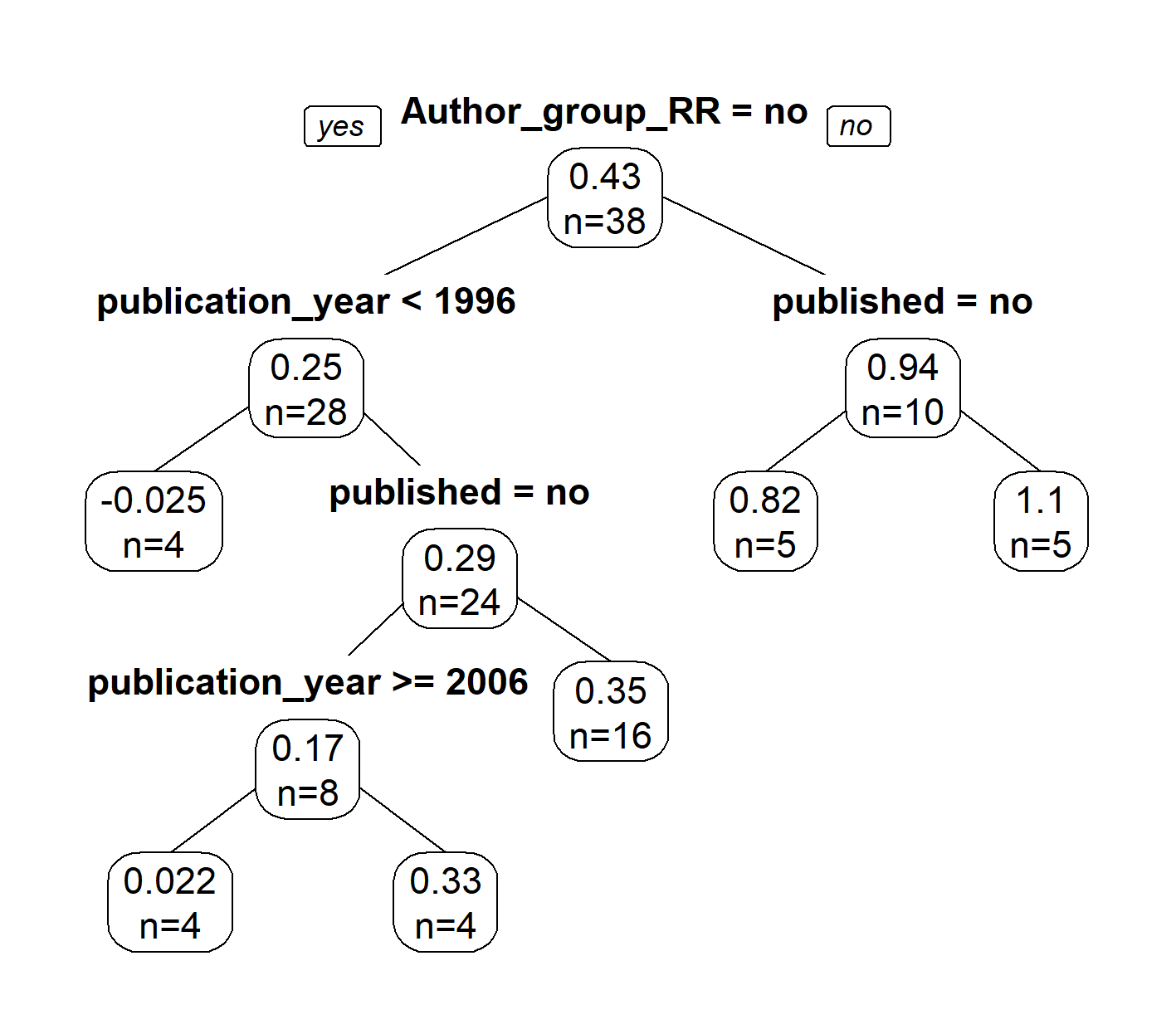 | 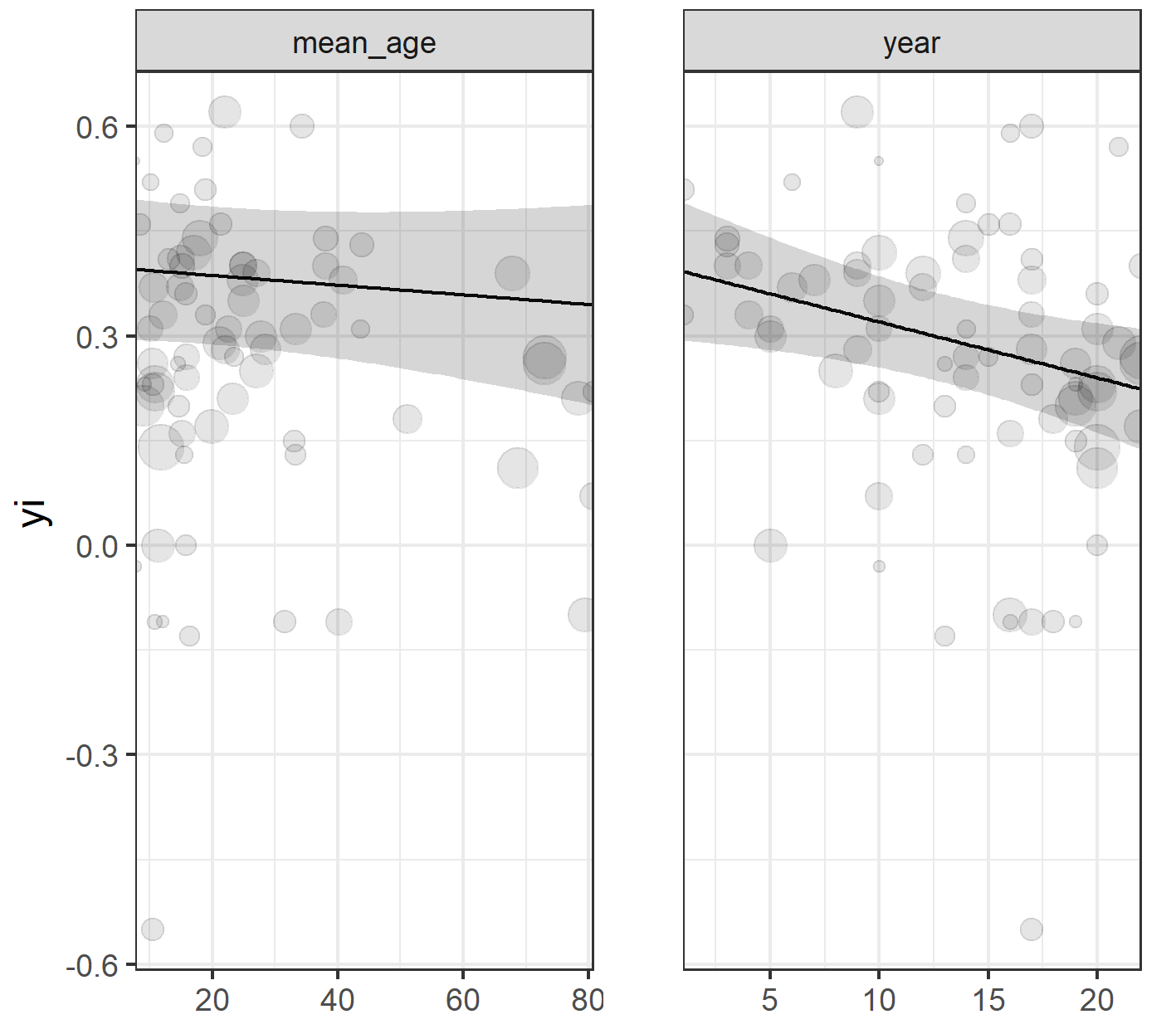 | 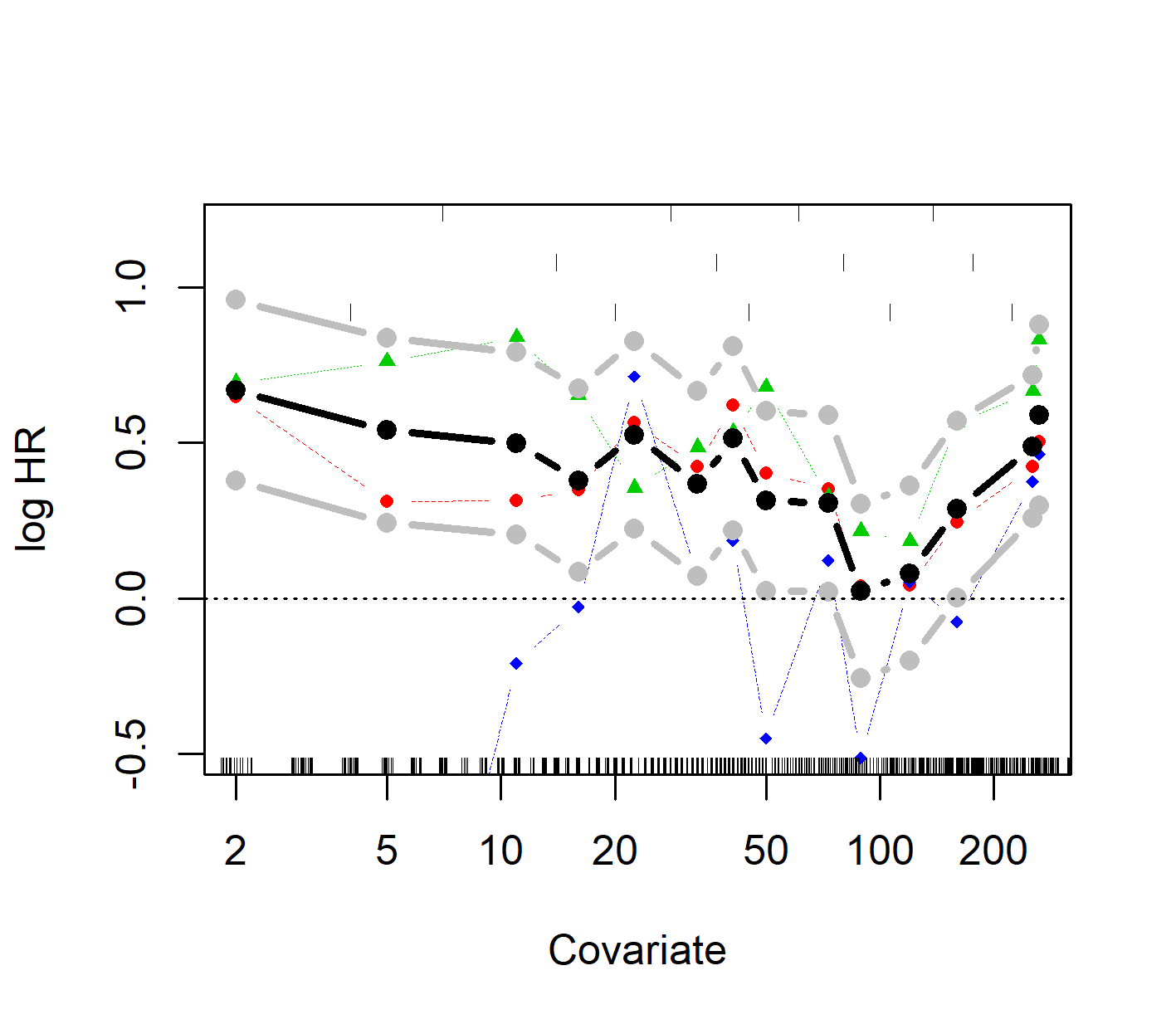 |
| Regression and classification tress have been recently introduced in the context of meta-analysis (Dusseldorp, van Genugten, van Buuren, Verheijden, & van Empelen, 2014). Regression tress successively partition the data into groups of studies with similar effect size. The used covariates and cutoff values forming the tree and the corresponding decision rules can be easily visualized by a tree displays. Starting from the top of the tree, a series of binary decisions regarding study covariates leads to an effect size estimate for a given study. Regression trees can help to explore and visualize drivers of between study heterogeneity. The above example plot was created with the R package rpart (Therneau & Atkinson, 2018). | As an alternative to regression trees, random forests have been proposed to explore between-study heterogeneity, using several covariates in the context of meta-analysis (van Lissa, 2017). Meta-analytic partial dependence plots show the estimated (marginal) association of a specific covariate with study effect sizes, when all other covariates in the random forest are averaged out. This partial dependence line can inform about the estimated nature of the association with the covariate of interest (e.g., linear or non-linear; monotone or not). The above example plot was created using the R package metaforest (van Lissa, 2017). | The Meta-STEPP (subpopulation treatment effect pattern plot) accompanies a recently proposed algorithm to detect and test associations of the treatment effect with a covariate in time-to-event data (Wang, Cole, Bonetti, & Gelber, 2016). In essence, using individual patient data, the summary log hazard ratio against a reference treatment is estimated for intervals of increasing covariate values. The resulting Meta-STEPP result and confidence interval is shown (in the example above with black and grey thick lines), while the corresponding study-level results are shown as well (in the above example: colored thin lines). Observed events in all studies and corresponding covariate values are shown with tick marks at the bottom of the graph. For further details, see Wang, Cole, Bonetti, and Gelber (2016). The above plot was created using the R package metaStepp and example data therein (Wang, Cole, Bonetti, & Gelber, 2016). |

| Tau square sensitivity plot [4.1] | Tau square sensitivity plot with posterior distribution [4.1.1] | Leave-one-out sensitivity lineplot [4.2] |
| --- | --- | --- |
| 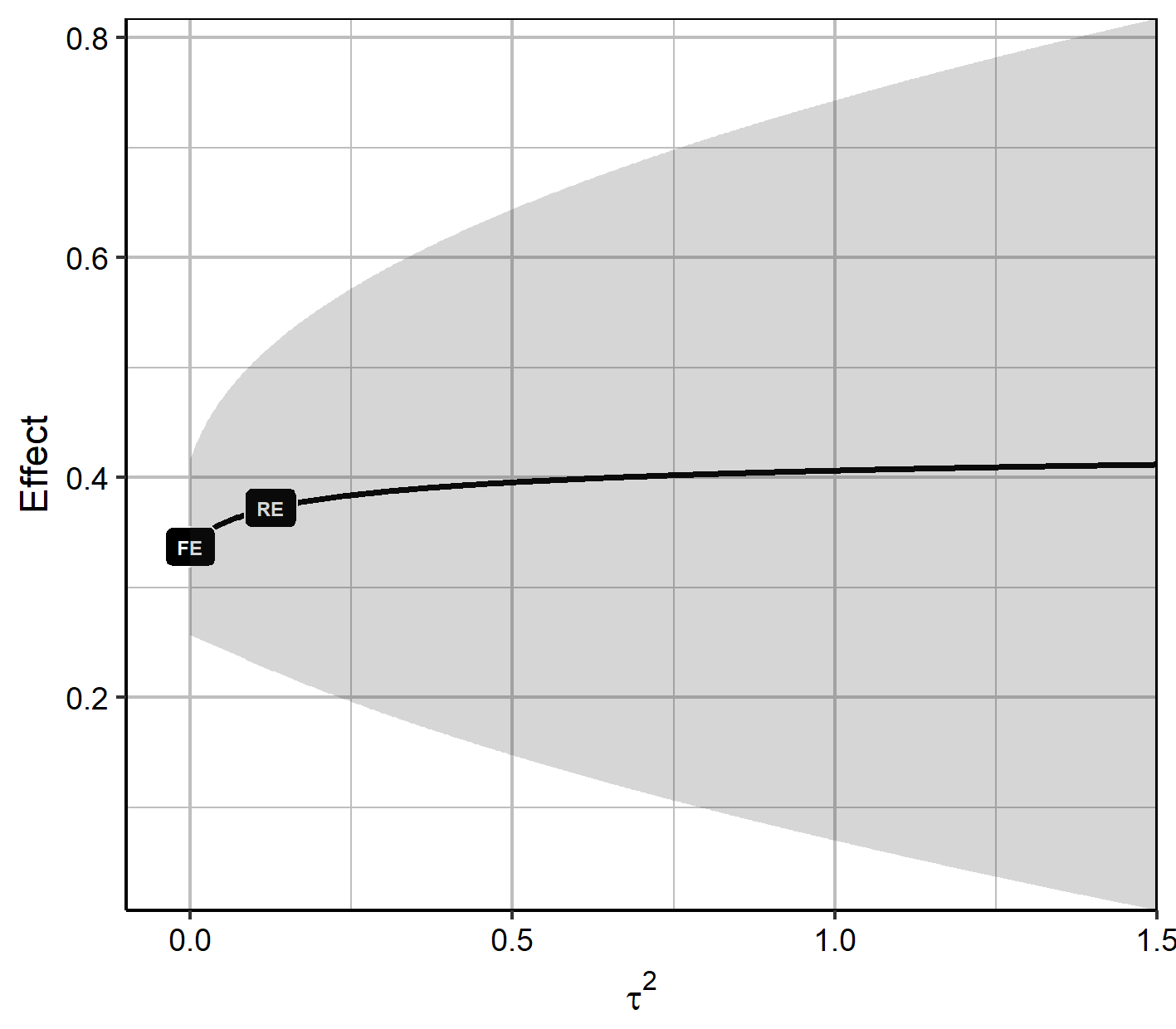 | 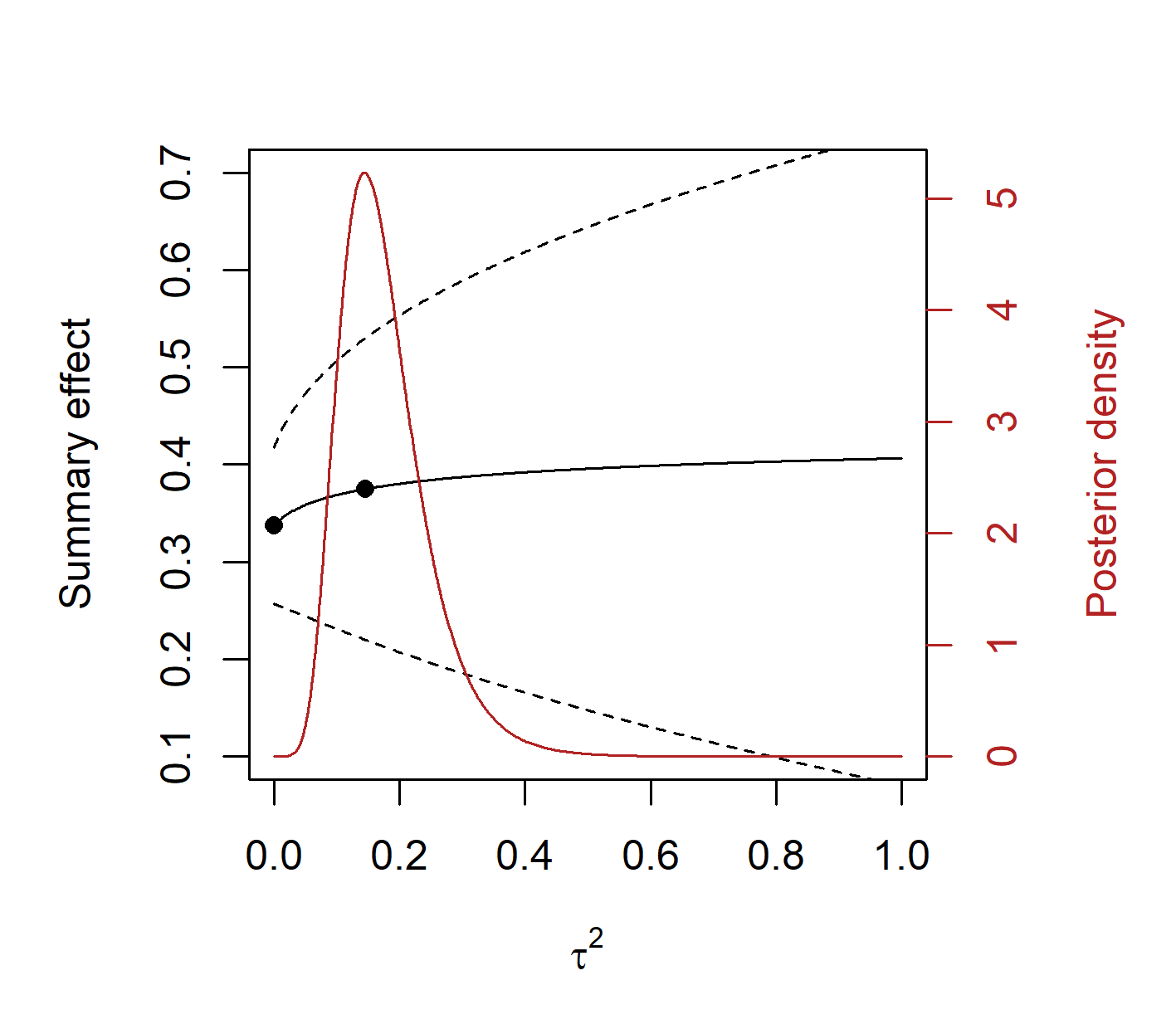 | 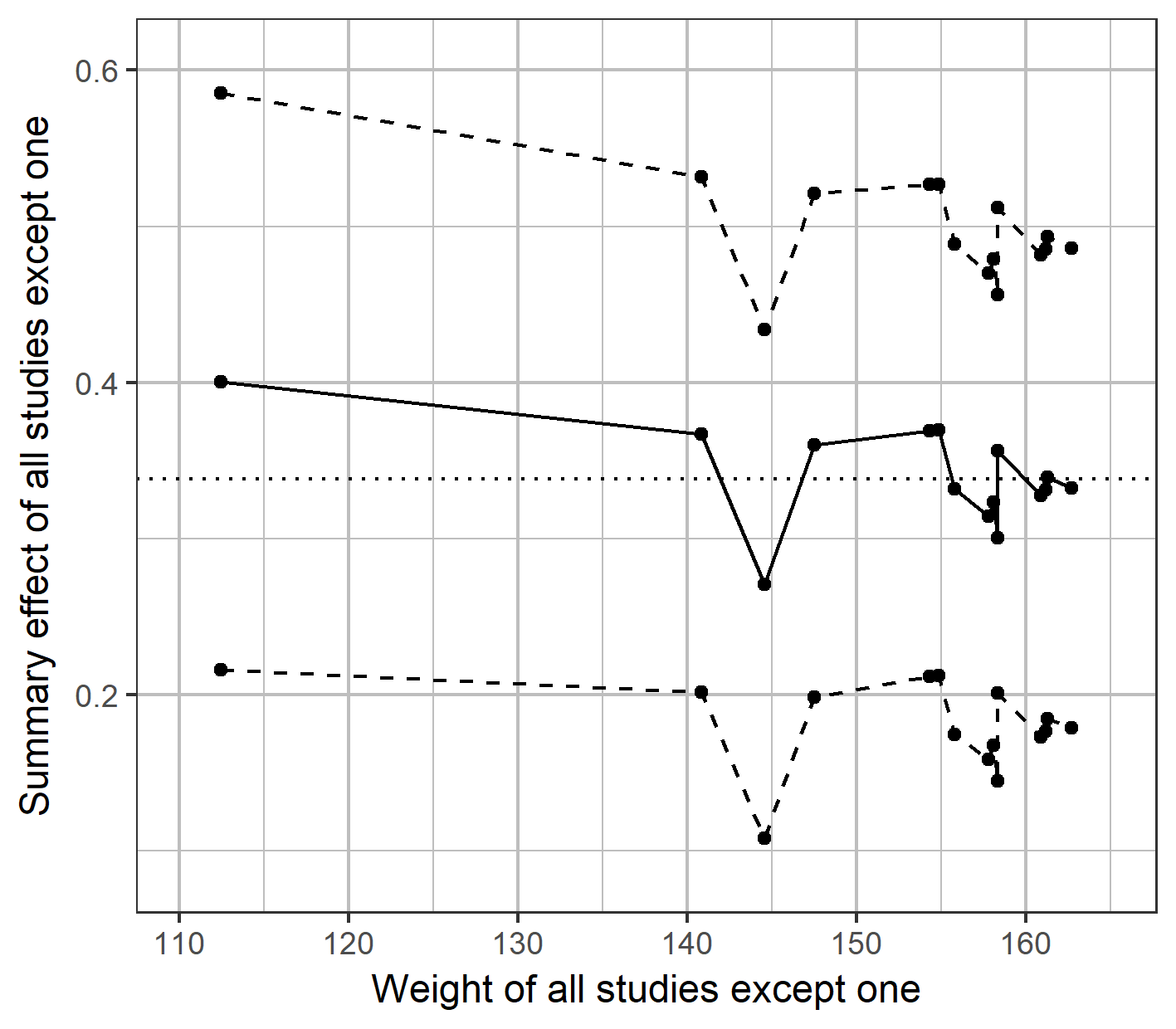 |
| The magnitude of the between-study variance parameter τ^2^ in a meta-analytic random effects model influences the estimated meta-analytic summary effect. For very large values of τ^2^, the summary effect estimator converges to an unweighted mean of the effect sizes, whereas for a τ^2^ of zero, it coincides with the fixed-effect estimator. The tau square sensitivity plot shows the trace of summary effect values and corresponding confidence intervals as a function of τ^2^ (Thompson, 1993). In addition, the actually estimated τ^2^ value is highlighted. Similar trace plots have also been used in the context of Bayesian meta-analysis to visualize the sensitivity of the posterior effect distribution of the summary and study effects to the size of the between-study variance component (e.g., Rubin, 1981). | A variant of the tau square sensitivity plot [4.1] in the context of Bayesian meta-analysis additionally shows the estimated posterior density of the between-study variance parameter τ^2^. Information on the posterior probability/density of τ^2^ values can therefore be incorporated in the assessment of the sensitivity of the meta-analytic summary effect to different τ^2^ values (for an example application, see Stangl & Berry, 2000, pp. 150-151). | The leave-one-out sensitivity lineplot has been proposed to explore the sensitivity of the meta-analytic summary effect to the exclusion of single studies from the meta-analysis (Elvik, 1998). Each study in the meta-analysis is left out once, and a meta-analysis with the remaining studies is computed. The resulting estimated summary effects and 95% confidence intervals are shown on the y axis, and the sum of all meta-analytic weights of the remaining studies is shown on the x axis. The meta-analytic summary effect including all studies is shown by a horizontal line. Leave-one-out results are shown with dots connected by lines. Deviations of the horizontal line indicate sensitivity of the results to the exclusion of a specific study. |
| Leave-one-out sensitivity forest plot [4.2.1] | Baujat plot [4.3] | Number of additional participants required to obtain significance plot [4.4] |
| 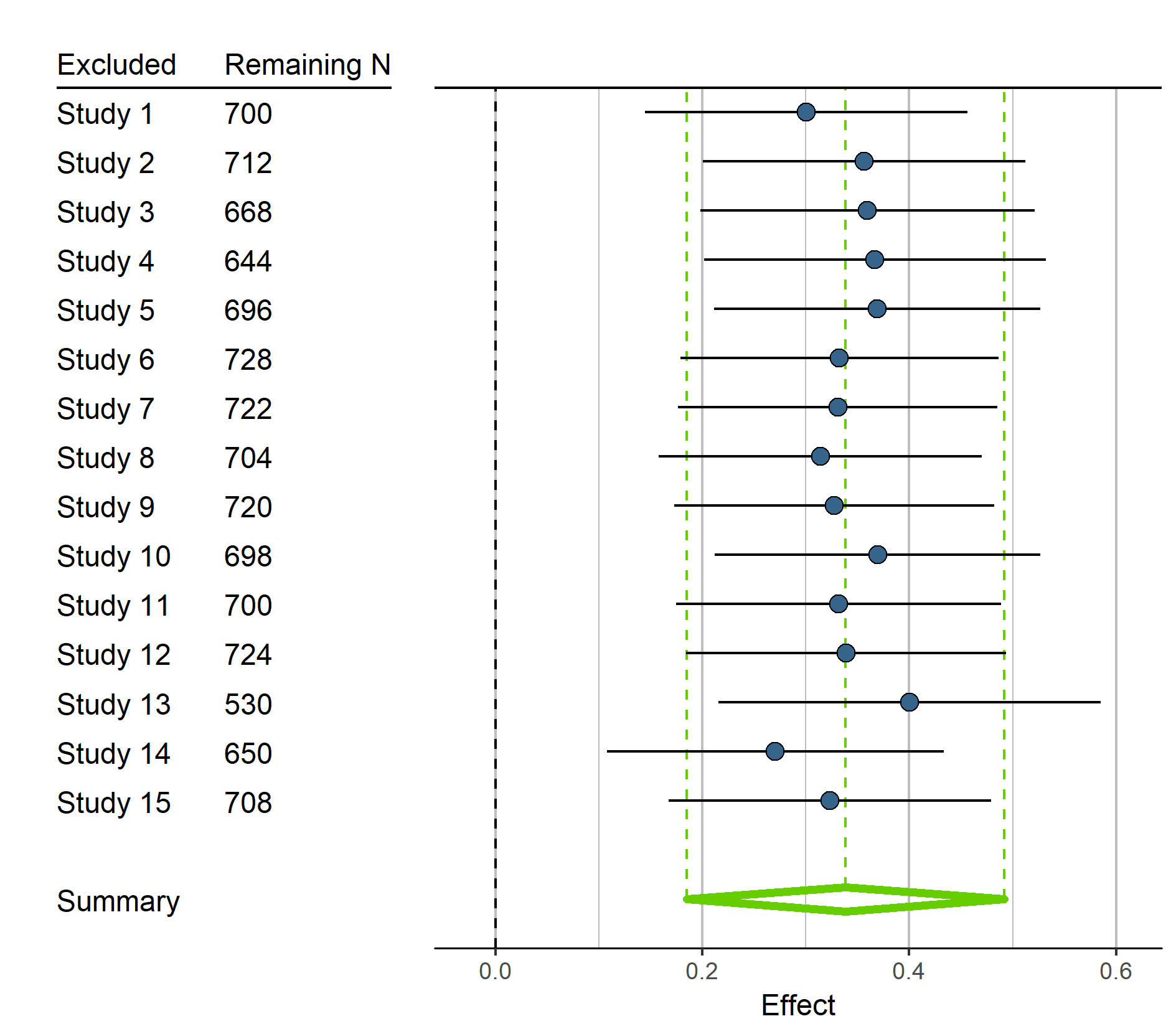 | 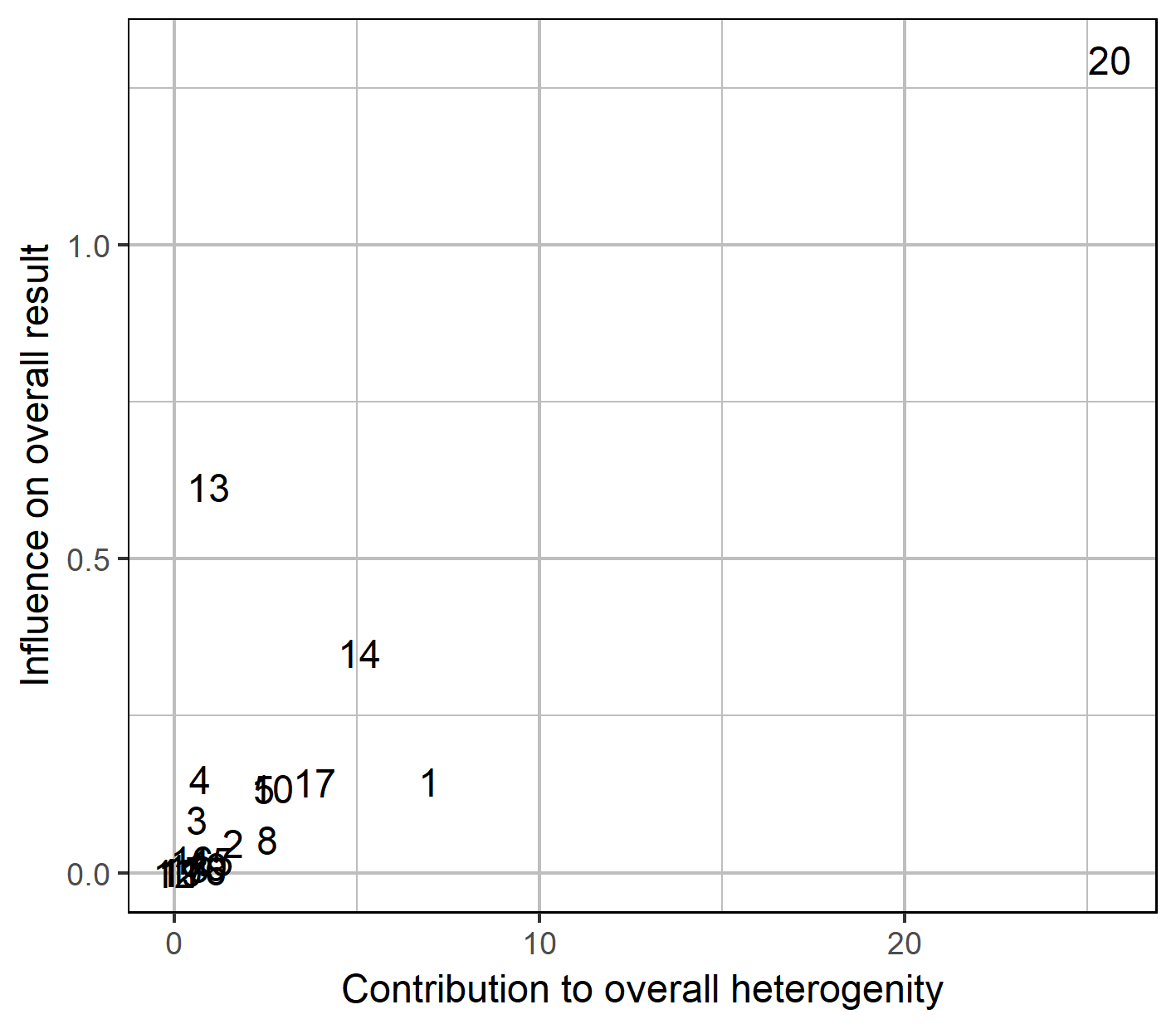 | 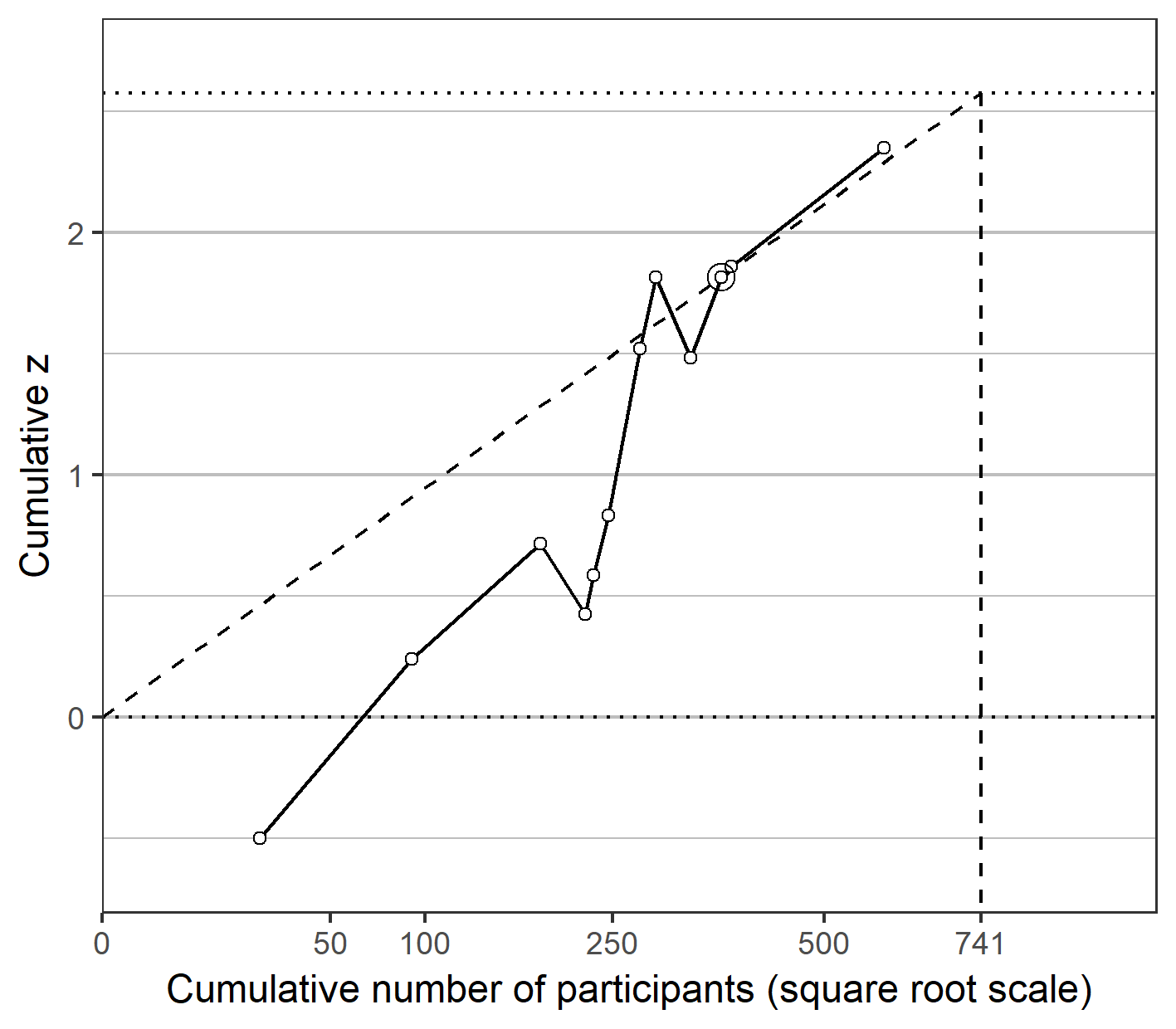 |
| Forest plots have been proposed and regularly used to visualize leave-one-out sensitivity analysis for checking the robustness of the meta-analytic results to the exclusion of single studies from the meta-analysis. For each study excluded, the meta-analytic summary effect of the remaining studies and the corresponding confidence interval is displayed. At the bottom, the overall meta-analytic summary effect using all studies is shown for comparison. Reference lines for the null effect, overall summary effect, and confidence limits of the overall summary effect support the evaluation of the leave-one-out results. As in the regular forest plot [1.2], the sequence of excluded studies in the leave-one-out sensitivity forest plot is arbitrary and can be defined by the user. An example application can be found in Sutton, Abrams, Jones, Sheldon, and Song (2000, p. 149). | The Baujat plot has been proposed to identify studies that are main sources of between-study heterogeneity in a meta-analysis (Baujat, Mahé, Pignon, & Hill, 2002). For each study, the influence on the summary effect and the contribution to the between-study heterogeneity is shown in a scatterplot. The influence on the summary effect for a specific study is defined as the squared (standardized) difference of the meta-analytic summary effect, as estimated with and without the study in question. The contribution of the study to the between-study heterogeneity (*Q* statistic) is the weighted and squared difference of the observed effect sizes from the estimated summary effect. All studies are marked with study identifier numbers. The Bajuat plot allows to identify studies that are unusual (i.e, sources of heterogeneity), influential, or both. Studies that are highly influential can appear to be no source of heterogeneity, but potentially nevertheless drive heterogeneity by pulling the summary effect away from all other (potentially homogenous) studies. Such studies can be found in the upper left corner of the Baujat plot. | The number of additional participants has been proposed and used to graphically show the amount of new evidence necessary, such that the meta-analytic summary value is expected to reach significance (for further details, see Barrowman, Fang, Sampson, & Moher, 2003). The solid line shows the *z* value for all evidence up to a given accumulated sample size over all studies (shown on the x axis on the square root scale). A diagonal dashed line passes through the origin and a meta-analysis of the first *k* studies (in the example above the first 10 studies). The value on the x axis corresponding to the point where the diagonal dashed line crosses the significance threshold of interest (e.g., for α = .01, two-sided, as in the above example) shows the number of cumulative participants necessary. The plot can be used as a robustness check of the (non-)significance of a meta-analytic summary effect and can help to find meta-analyses worth updating with new evidence (see also [5.2.2]). |
| Influence plot [4.5] | Study influence and outlier diagnostic lineplots [4.6] | Metaplot [4.7] |
| 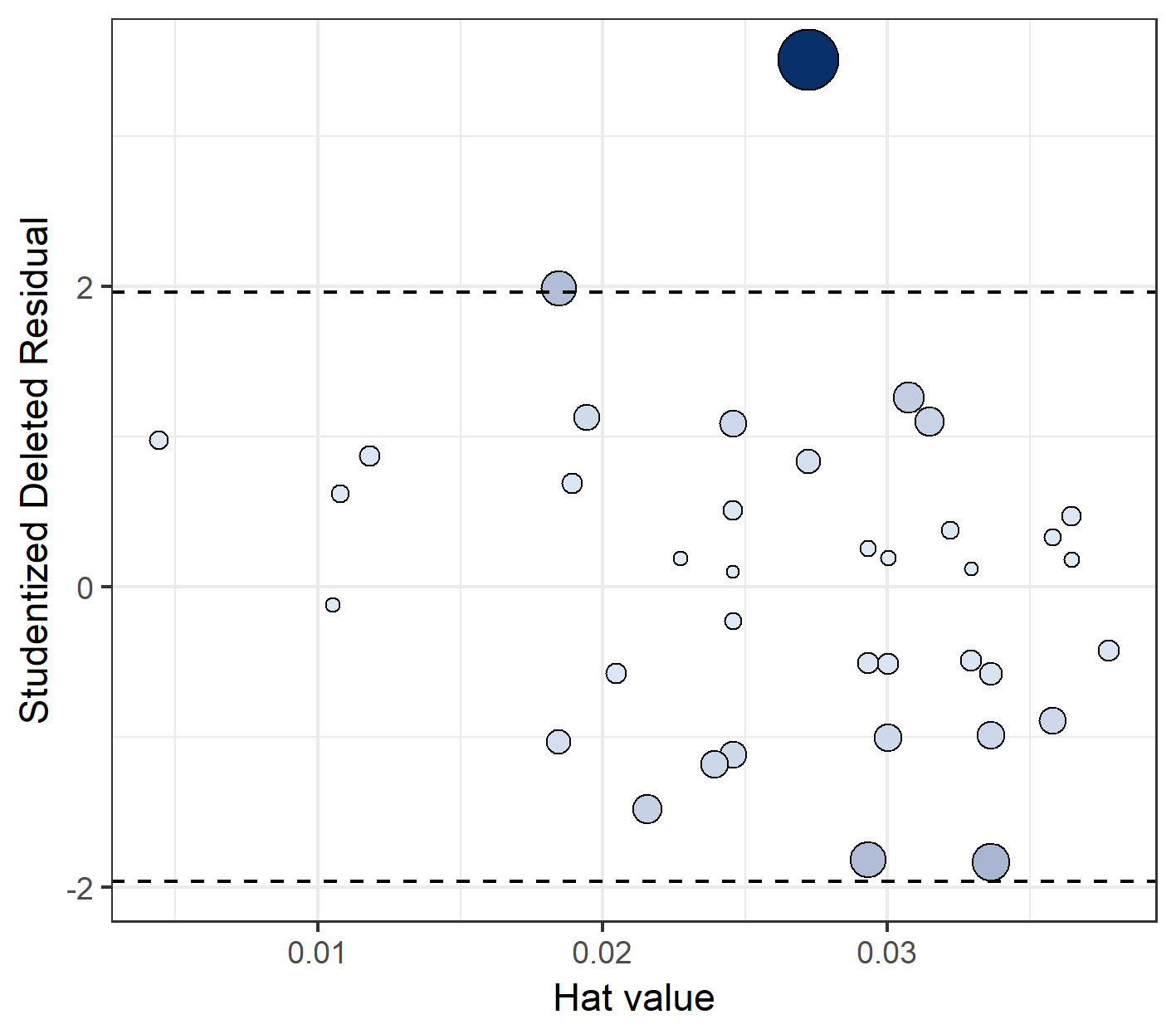 | 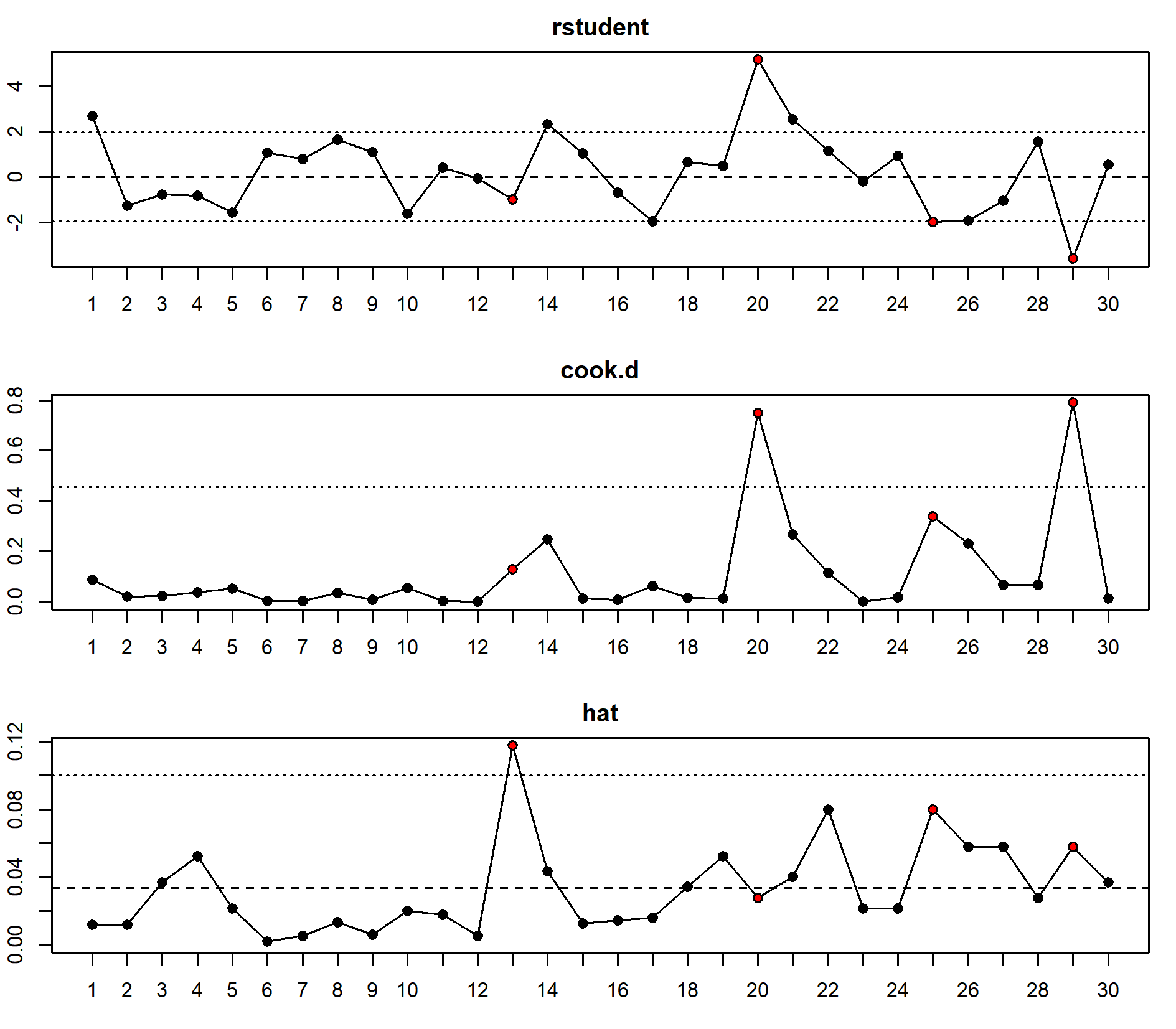 | 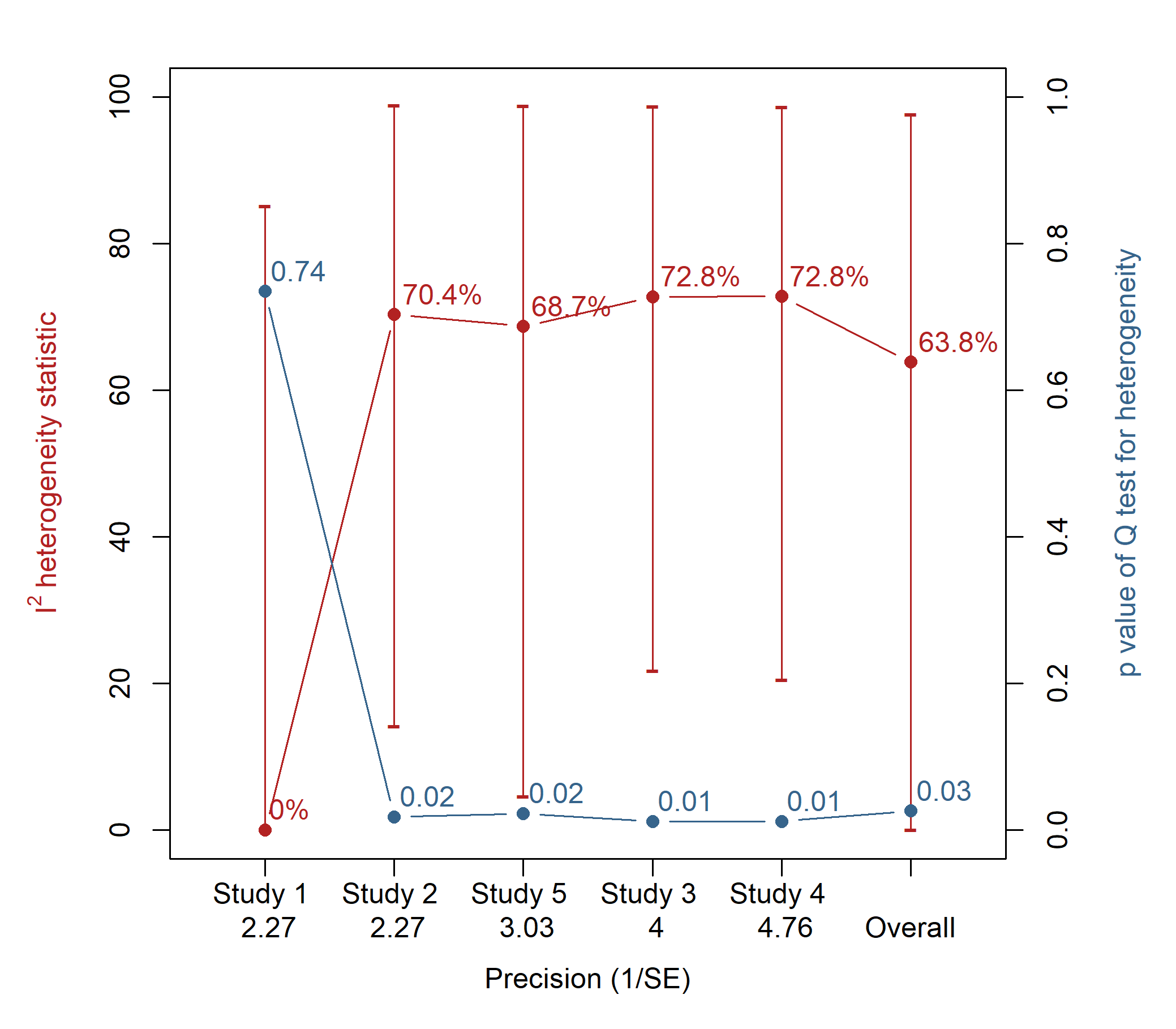 |
| General outlier, leverage, and influence statistics from linear regression analysis and corresponding visualizations have been introduced in the context of meta-analysis and meta-regression in particular (Viechtbauer & Cheung, 2010). The influence plot shows three of these statistics. First, studentized deleted (leave-one-out) residuals are shown on the y axis as measure for the outlyingness of observed study effects. Second, hat values are shown on the x axis as indicators for the unusualness of covariate value combinations for each study (i.e., leverage). Third, points representing each study are proportional in size to their Cook’s distance as a measure of influence on the meta-analytic summary estimate. For further details, see Viechtbauer and Cheung (2010). | An alternative to the influence plot [4.5] is a line plot, showing a specific outlier, leverage, or influence statistic for each study in the meta-analysis (Viechtbauer & Cheung, 2010). Study values are arranged by an identifier number in arbitrary order and connected by lines. Statistics proposed for this purpose are studentized deleted residuals (outlyingness), Cook’s distance (influence), hat values (leverage), and the meta-analytic weight of each study. In addition, the COVRATIO, DFITTS, and DFBETAS statistic have been used, indicating the effect that deleting one study has on the variance covariance matrix of the parameter estimates, the meta-analytic summary estimate, and the meta-regression parameters, respectively. In addition, the effect of the exclusion of single studies on the estimated heterogeneity can be of interest and visualized. For further details, see Viechtbauer and Cheung (2010). | The metaplot has been proposed as statistical display to visualize between study-heterogeneity in a meta-analysis (Poorolajal, Mahmoodi, Majdzadeh, & Fotouhi, 2010). Heterogeneity statistics are shown for the meta-analysis (overall), as well as for each study left out once and for meta-analyses computed with all remaining studies (leave-one-out meta-analyses). The display uses dual y axes to simultaneously display two heterogeneity statistics: *I*^2^ and the *p* value of the *Q* test statistic of cross-study effect heterogeneity. Changes in the (leave-one-out) heterogeneity statistics might indicate single drivers of between-study heterogeneity. |
| GOSH plot [4.8] | Outlier probability plot [4.9] | Forward plot [4.10] |
| 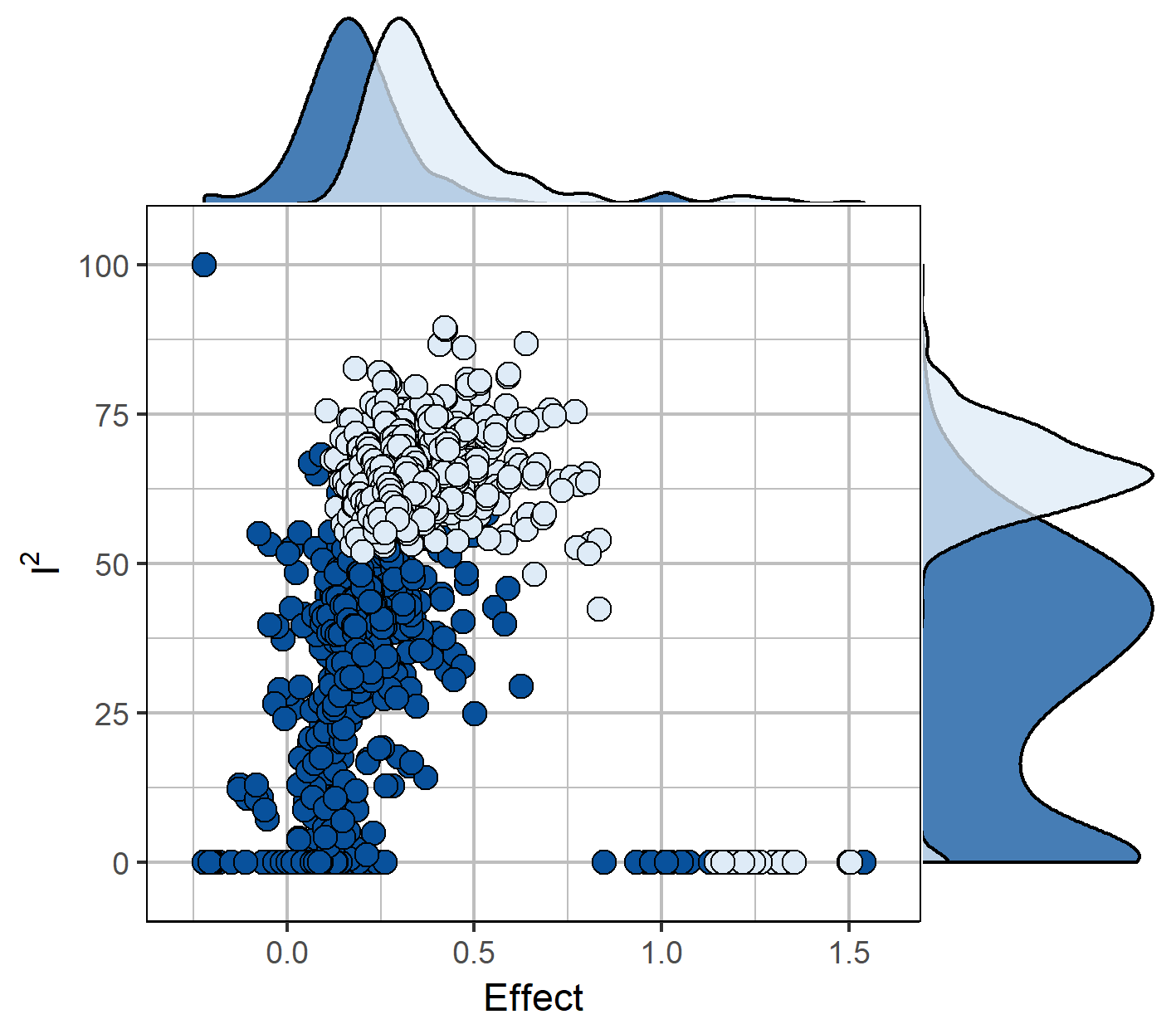 | 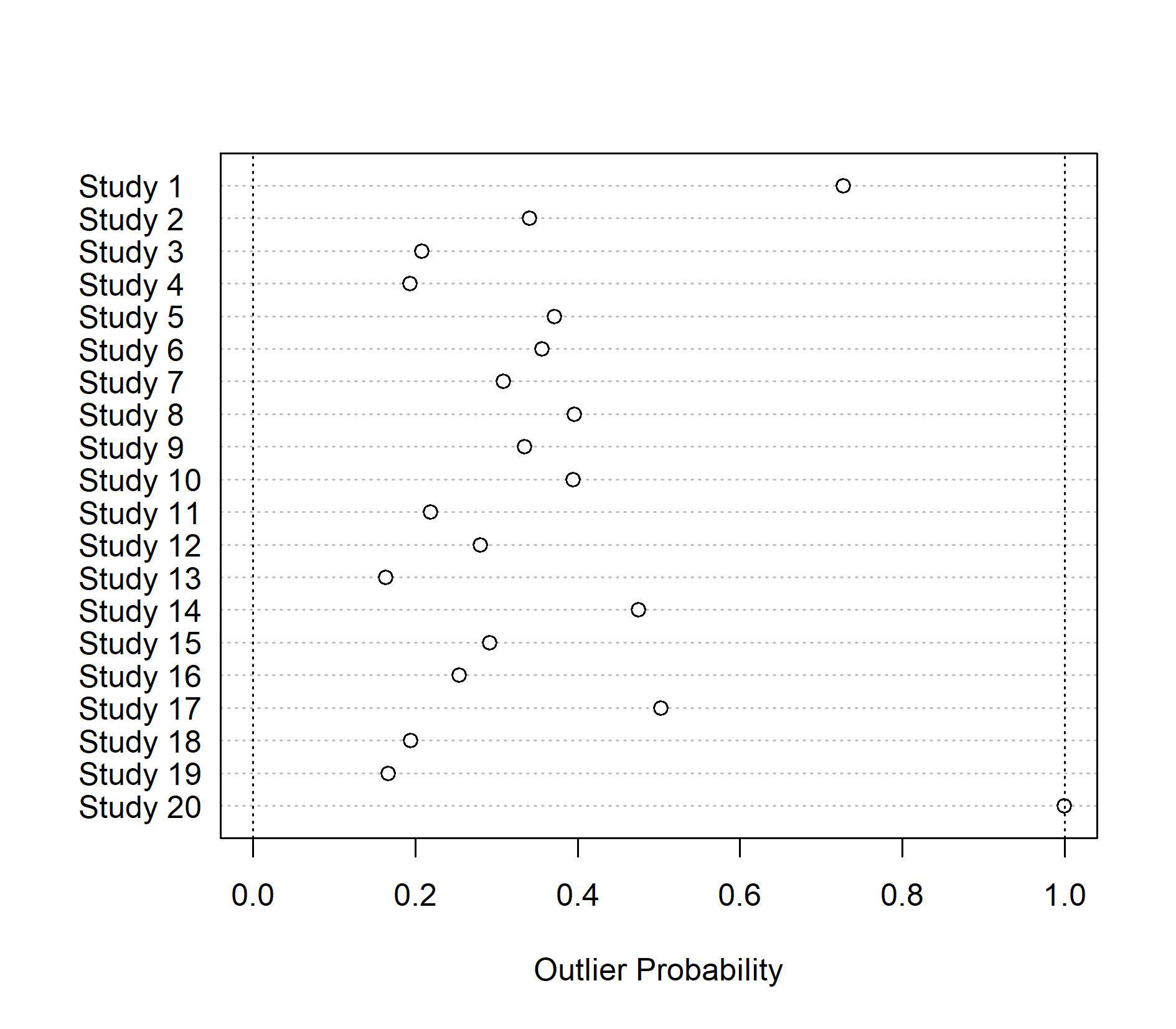 | 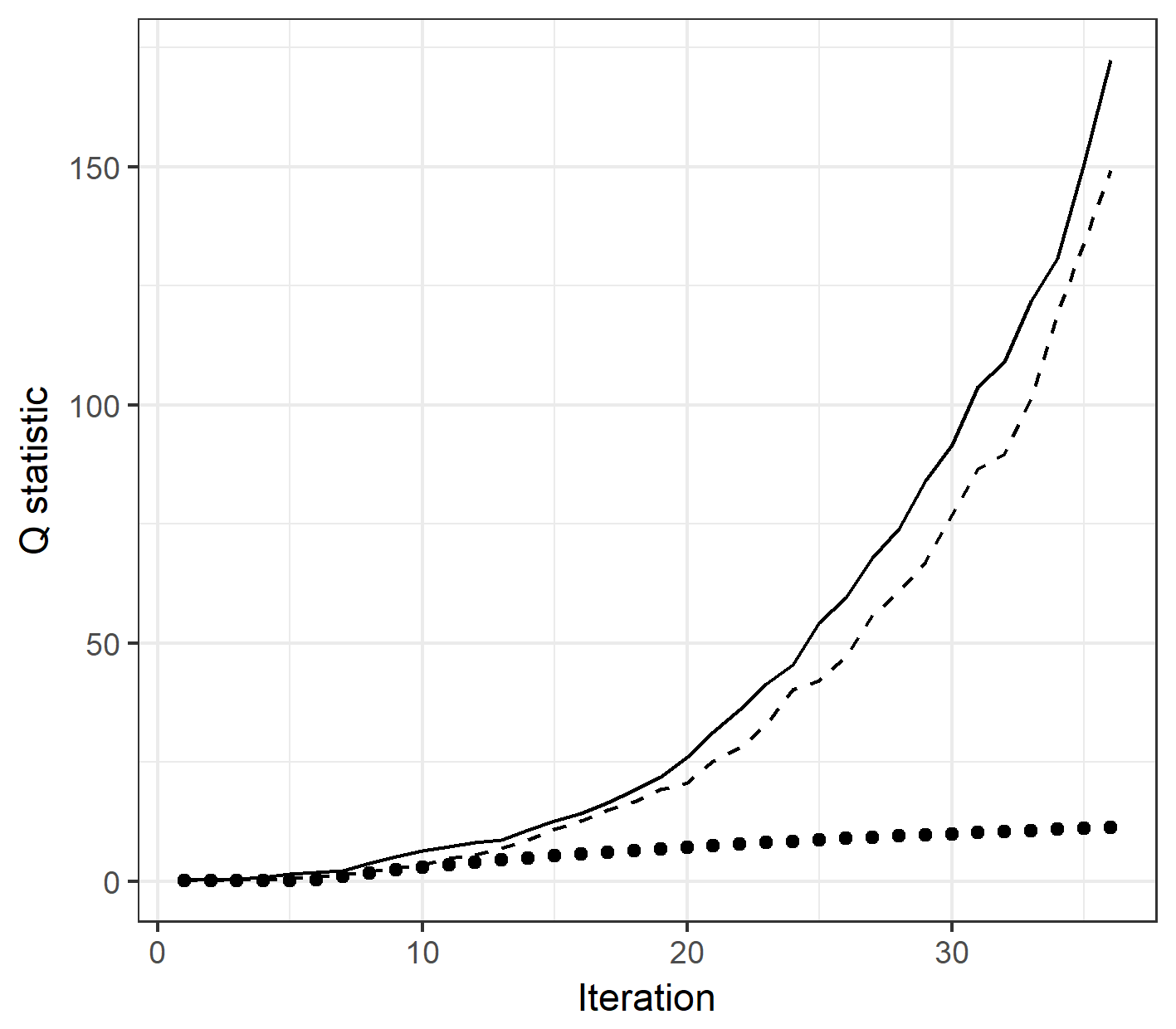 |
| The graphical display of study heterogeneity (GOSH) has been proposed to present results of all-subset meta-analysis (i.e., combinatorial meta-analysis; Olkin, Dahabreh, & Trikalinos, 2012). For *k* studies in a meta-analysis, there are 2*^k^* – 1 study subsets for which a meta-analysis can be computed. The GOSH plot visualizes the results of all these subset meta-analyses in a scatterplot. One axis shows the summary effect and the other axis the between-study heterogeneity *I*^2^ statistic. Density estimates for the empirical distribution of these two statistics are shown in the scatterplot margins (see also [6.5]). Subsets including a specific study can be highlighted to check for the sensitivity of the meta-analysis summary statistics to the inclusion of this study. For further details, see Olkin, Dahabreh, and Trikalinos (2012); for an application example, see Voracek, Kossmeier, and Tran (2019). | The outlier probability plot accompanies a newly proposed meta-analytic modeling framework, wherein studies are seen as a finite mixture of outlying and non-outlying studies (Beath, 2014). In this framework, outlying studies are still included in the meta-analysis, but weighted down. The outlier probability plot shows for each study the posterior probability that this study belongs to the class of outlying studies in a dot chart. The plot above and underlying computations were obtained using the R package metaplus (Beath, 2016). | The forward plot accompanies an outlier detection algorithm (Mavridis, Moustaki, Wall, & Salanti, 2016). In essence, the algorithm estimates a meta-analysis with a subset of studies that likely is free from outliers and then sequentially adds new studies that are most similar to the current set. The change in statistics of interest (e.g., summary, effect, heterogeneity *Q* value, estimated τ^2^ parameter, or Cook’s distance) is monitored and shown by dots in the forward plot for each iteration (i.e., successive inclusion of a study). Sudden changes in the forward plot by including a study indicate a potential outlier. Monitoring boundaries obtained via parametric bootstrap are shown with solid and dashed lines. Exceeding these monitoring bounds indicate potentially outlying studies that are unlikely to occur by chance alone. For further details, see Mavridis, Moustaki, Wall, and Salanti (2016). |
| Impact of unmeasured confounding sensitivity plot [4.11] | Tau square estimator sensitivity plot [4.12] | Cross-validated residual plot [4.13] |
| 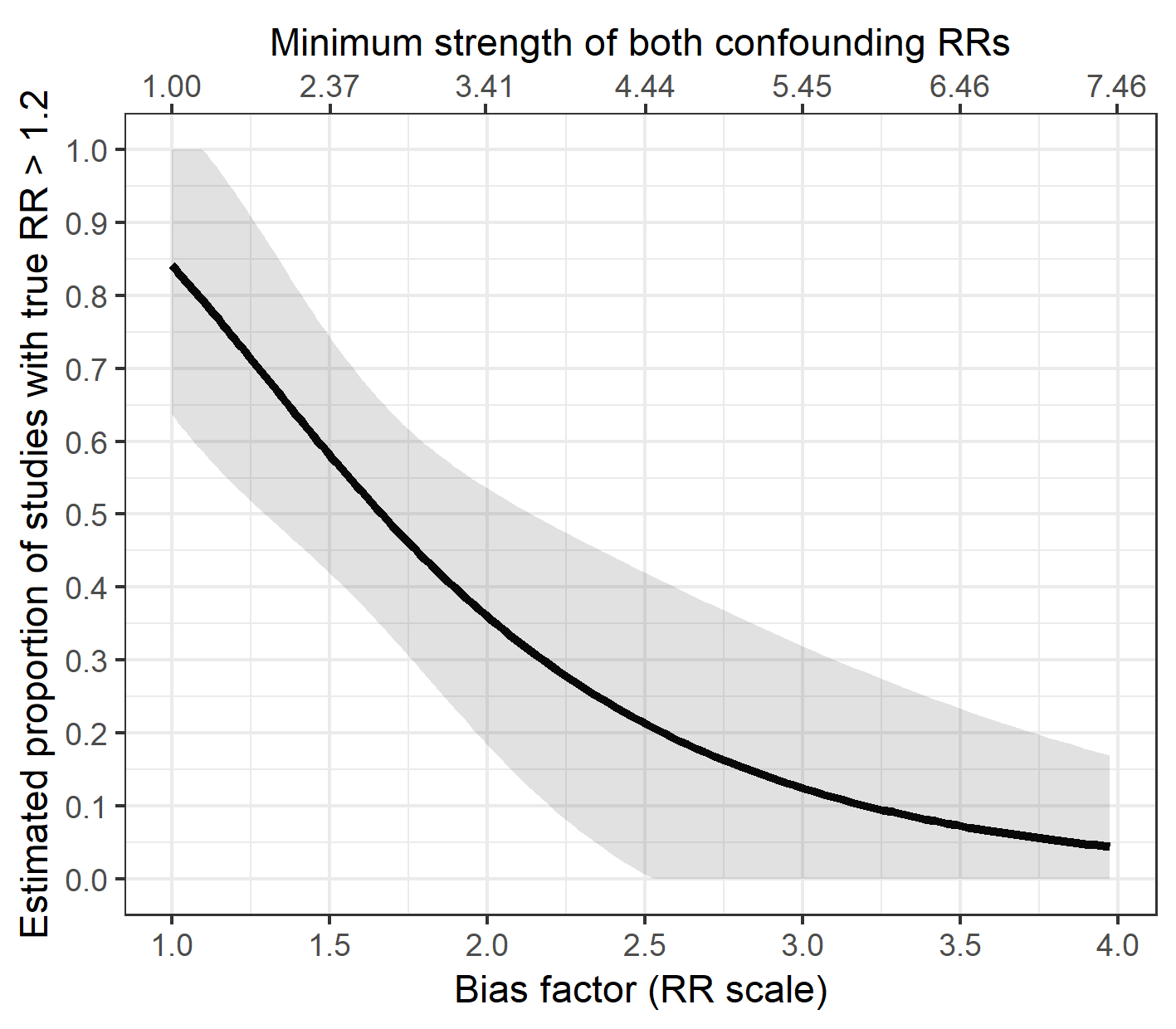 | 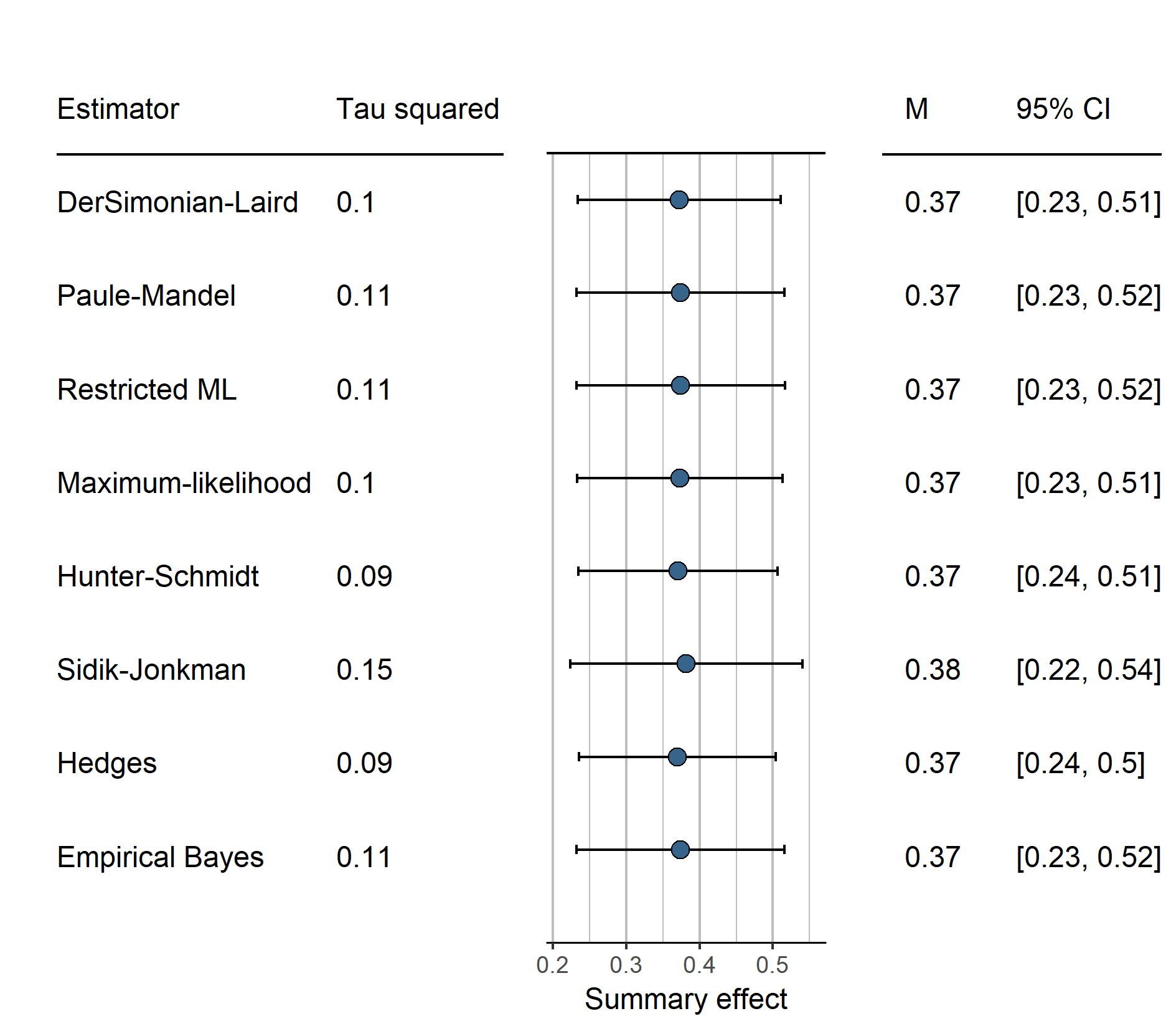 | c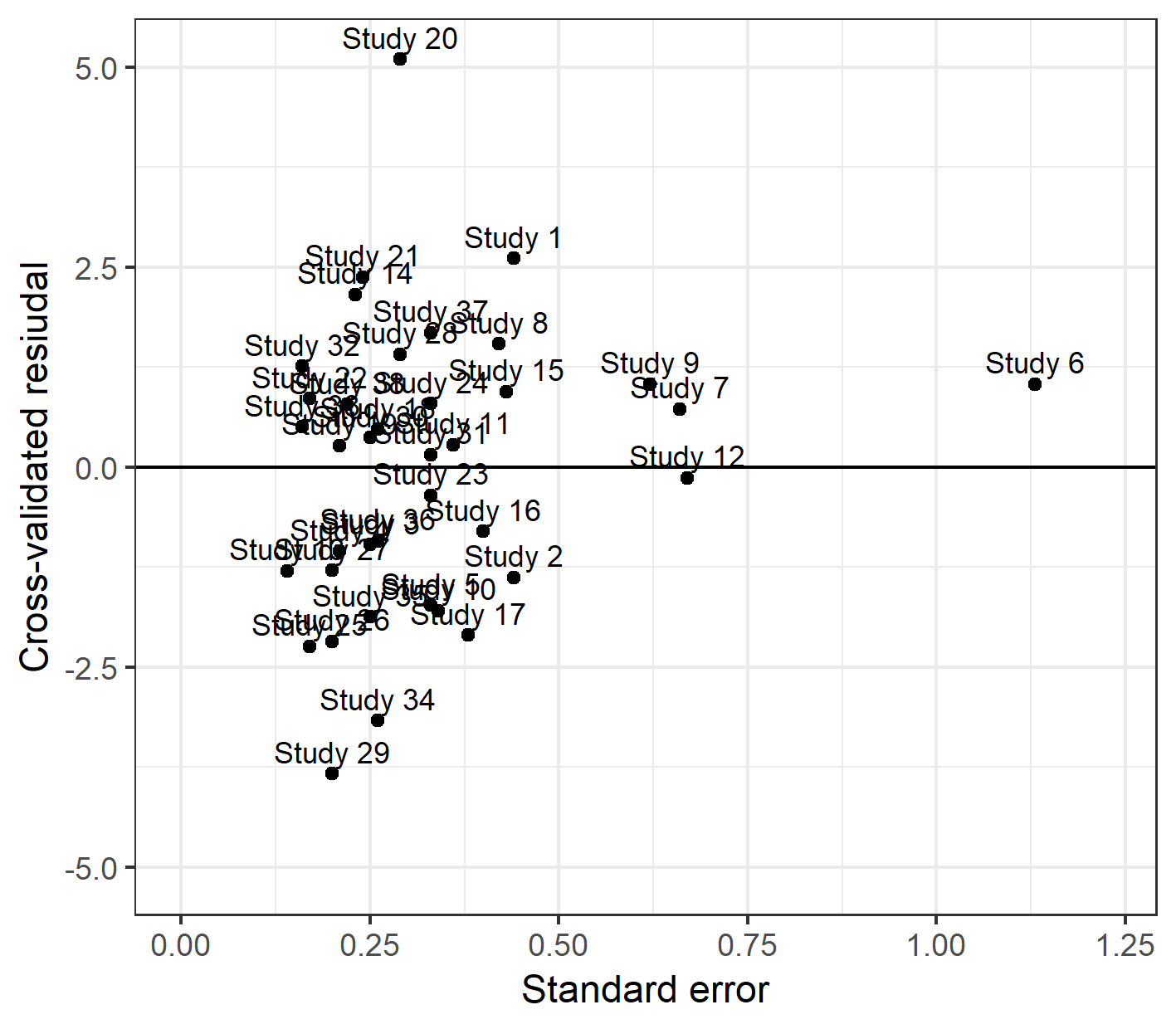 |
| The impact of unmeasured confounding sensitivity plot visualizes results of a proposed method to examine the robustness of random effects meta-analysis to unmeasured confounding in observational primary studies (Mathur & VanderWeele, 2017). The plot shows the estimated proportion and confidence interval of studies in the meta-analysis, with true underlying expected effects smaller (or higher) than an effect of interest as a function of the extent of unmeasured confounding (for further details, see Mathur & VanderWeele, 2017). The above plot and related results were created with the R package ConfoundedMeta (Mathur, Tyler, & VanderWeele, 2017). | Numerous estimators for the between-study variance parameter τ^2^ in the random-effects model exist (for a review, see Veroniki et al., 2016). The tau square estimator sensitivity plot shows the corresponding meta-analytic summary effect estimate for using a selection of these different estimators. Comparing the different results informs about the robustness of the meta-analytic summary effect to using different τ^2^ estimators. For an example application and further details, see Schwarzer, Carpenter, and Rücker (2015, pp. 34-37). | The cross-validated residual plot shows the cross-validated standardized residual for each study. Residuals can be obtained by using the meta-analytic summary estimate computed with leaving the study out (thus the term ‘cross-validated’ residuals; see also influence plot [4.5]). The cross-validated residuals of each study are plotted against their standard error, resulting in a plot similar to the funnel display [2.1]. If the meta-analytic model is correct, studies should scatter randomly and symmetrically around the horizontal zero line. Potential outliers are identifiable by large cross-validated residuals. Positive trends in the cross-validated residual plot indicate small-study effects (i.e., small studies with large standard errors tend to report larger effects). For further details and an example application, see Stangl and Berry (2000, pp. 154-155). |

| Effect-size time-series plot [5.1] | Quality control chart: X bar chart [5.1.1] | Quality control chart: CUMSUM chart [5.1.2] |
| --- | --- | --- |
| 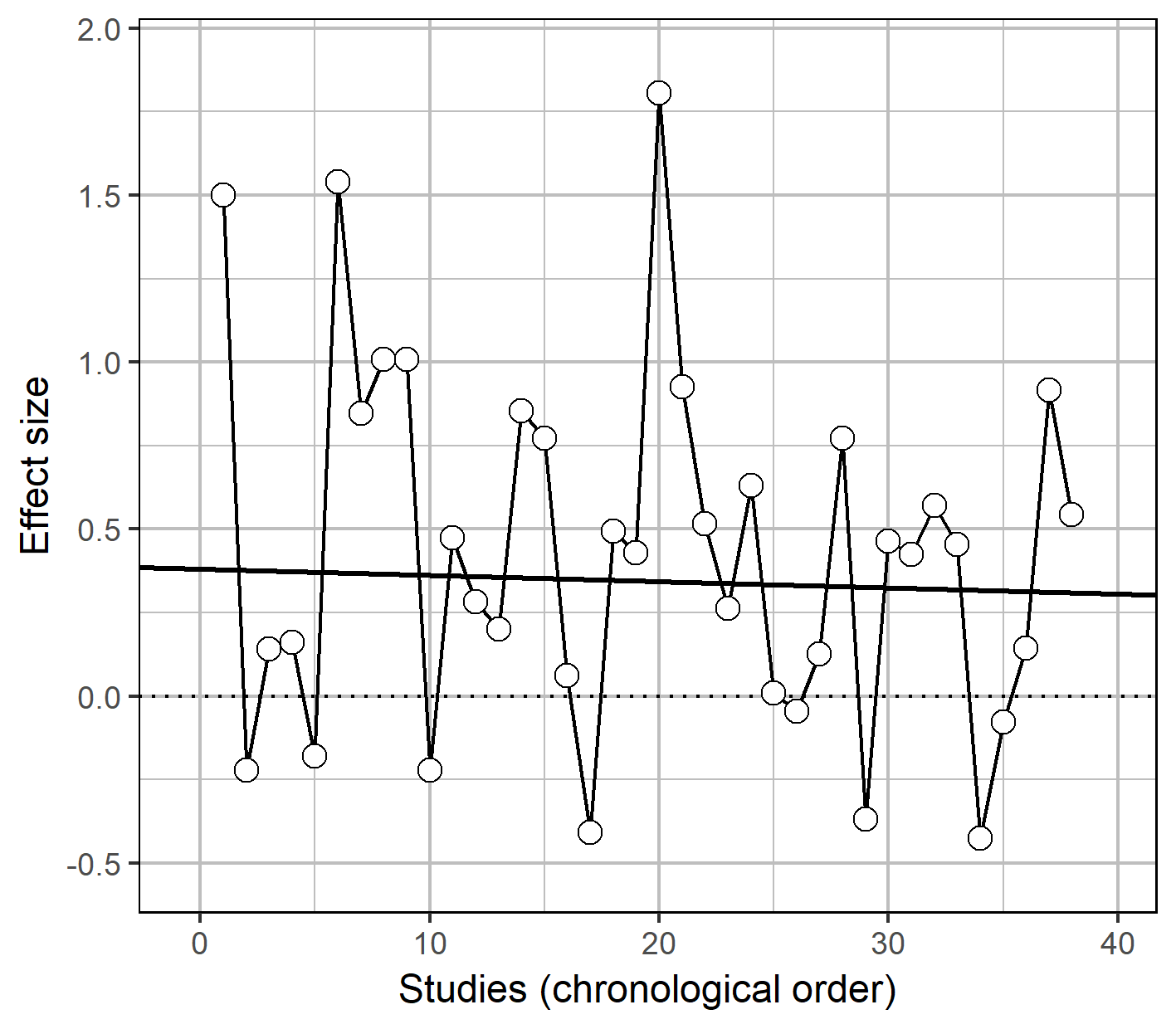 | 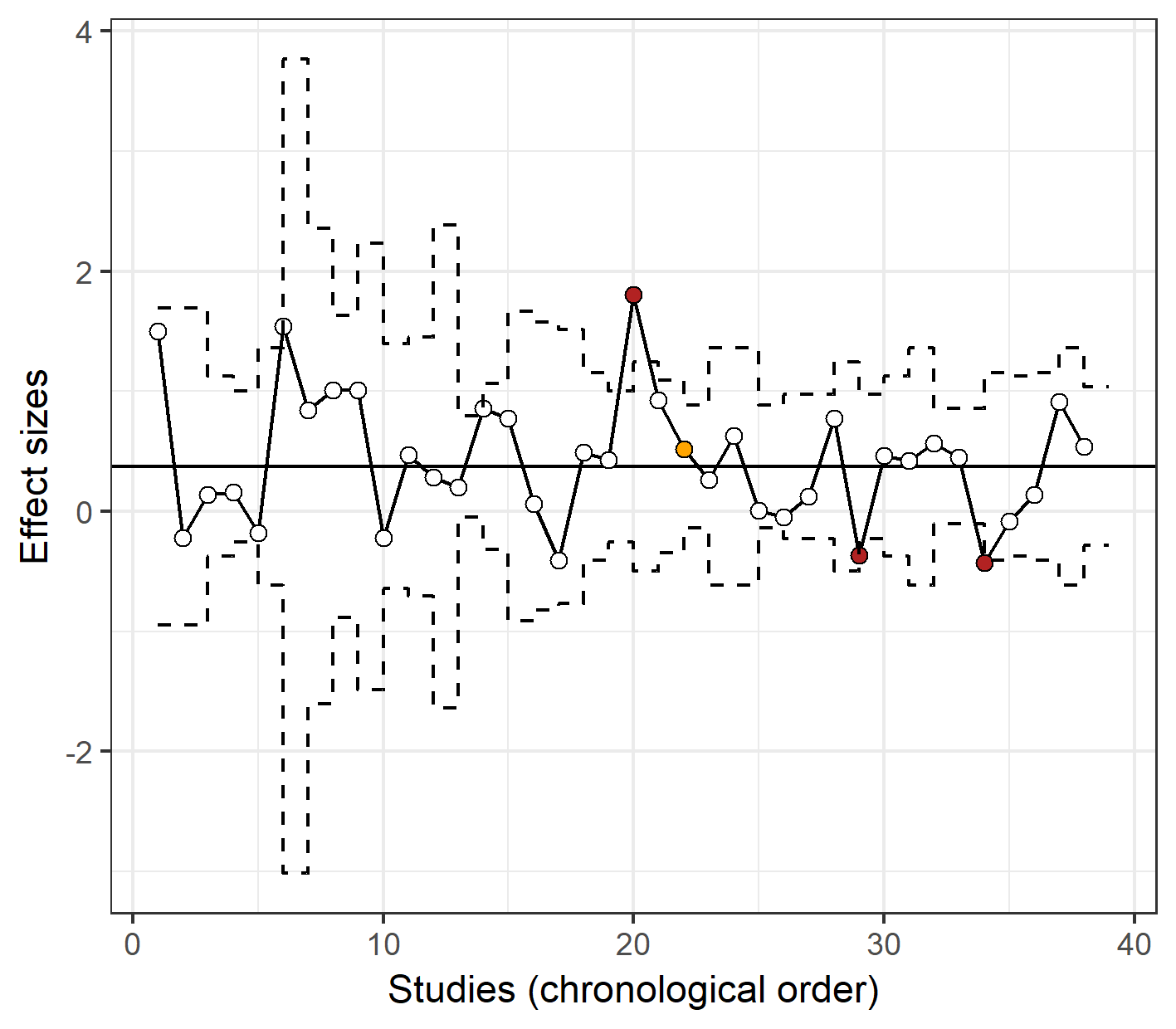 | 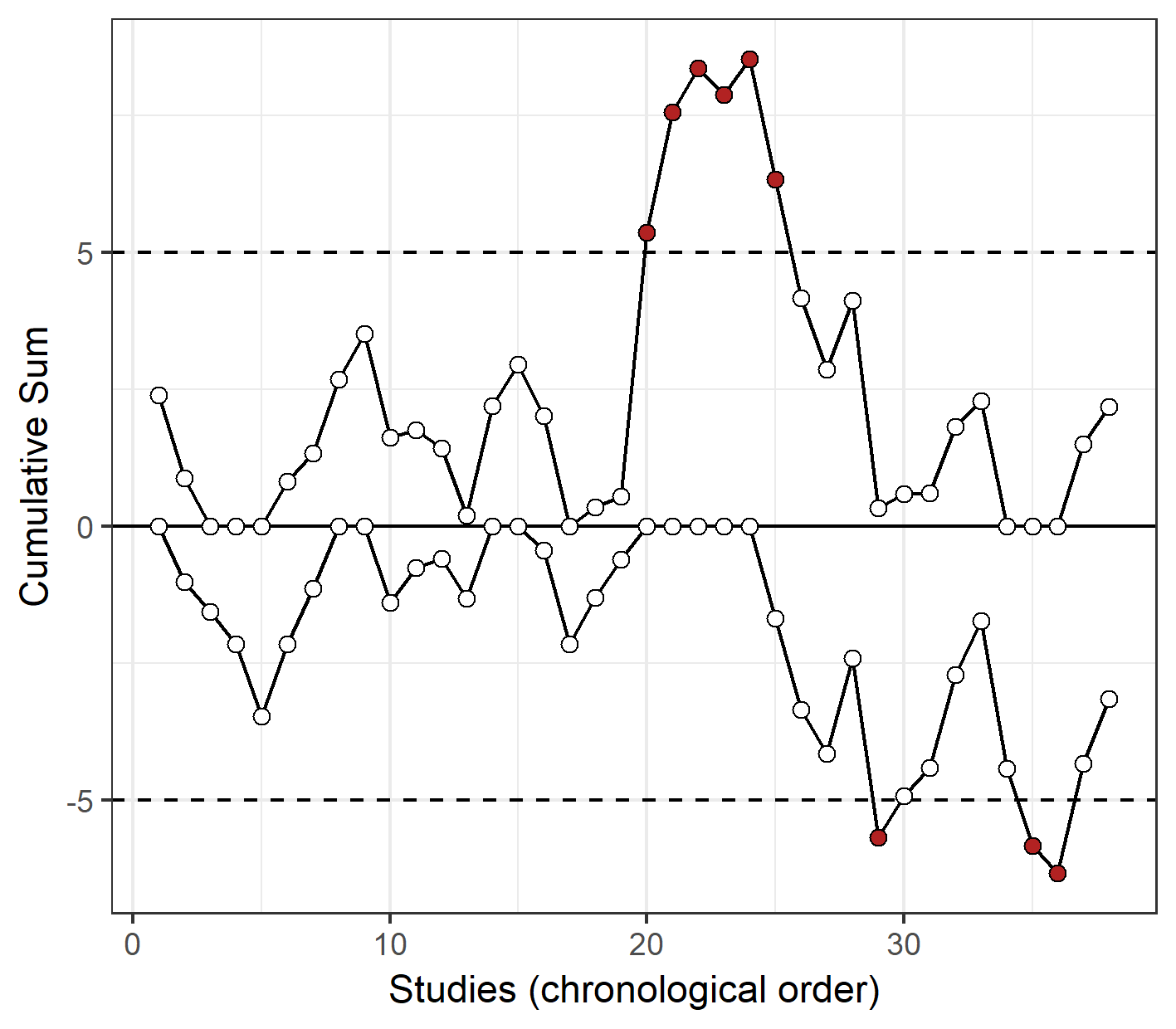 |
| The accumulation of evidence over time oftentimes is informative in meta-analyses as a robustness check. Time-series plots showing the observed effect sizes as a function of time have been used early on in the context of meta-analysis (Light & Pillemer, 1984, pp. 70-71). Effect sizes are shown in chronological order (or by publication date) as a special case of [3.1] and optionally connected by a line. In addition, a regression line can be overlain, to further indicate any positive or negative time trends. | The X bar chart as a standard quality control chart has been proposed to show outliers and trends over time in the context of meta-analysis. Quality control charts assume a fixed-effect model, with all study effects as realizations of a normal distribution with the same expected population effect size. The accumulating evidence is plotted as an effect-size time-series plot (as in [5.1]). In addition, the estimated meta-analytic summary effect is indicated by a horizontal line. Control limits are drawn for each study symmetrically around this summary effect (typically 3 or 5 times the study’s standard error). Studies exceeding the control limits are highlighted in red as unusually high or low. In addition, run tests are conducted, indicating whether there is an implausible number of consecutive studies with effect sizes all larger (or smaller) than the observed summary effect. The first study for which a run test gets significant, is highlighted in orange. For further details, see Kulinskaya and Koricheva (2010). | An alternative to the X bar chart [5.1.1] is the CUMSUM chart, which likewise is a standard quality control chart. The interpretation of CUMSUM chart might be less straightforward than the X bar chart, but the power to detect time trends is increased. In the CUMSUM chart, the cumulative standardized positive (upper line) and negative deviations (lower line) of the observed effect sizes from the expected effect size (e.g., the meta-analytic summary effect) are shown. In addition, the upper line is constrained to be never lower than zero, whereas the lower line cannot exceed zero. Control limits are visualized by horizontal lines (usually at positions -5 and 5). Exceeding the control limits (either in positive or negative direction) indicates time trends in the accumulating evidence (i.e., the effect-size process is then said to be out of control). For further details, see Kulinskaya and Koricheva (2010). |
| Cumulative meta-analysis plot [5.2] | Subgroup cumulative meta-analysis [5.2.1] | Cumulative meta-analysis with monitoring boundaries [5.2.2] |
| 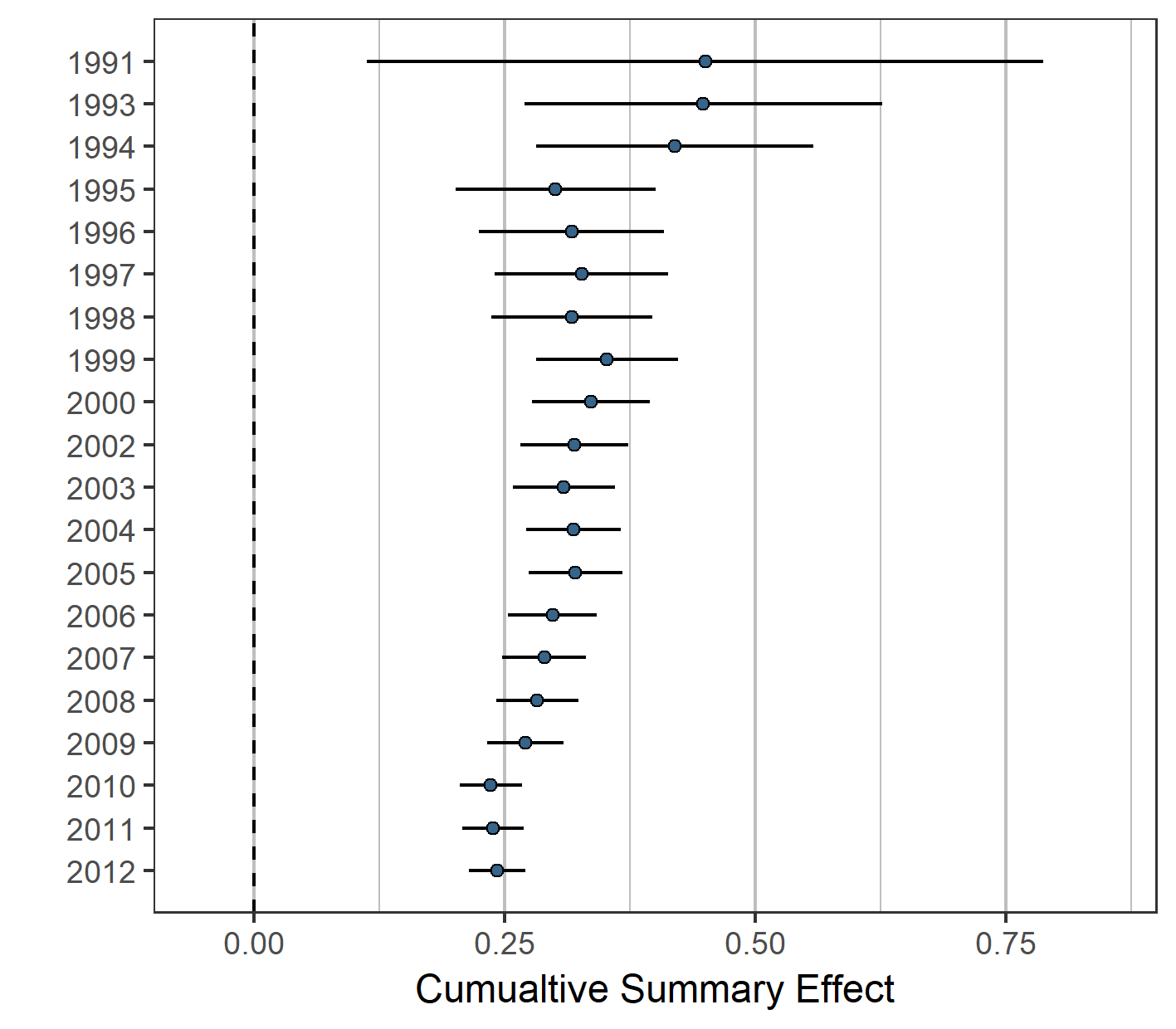 | 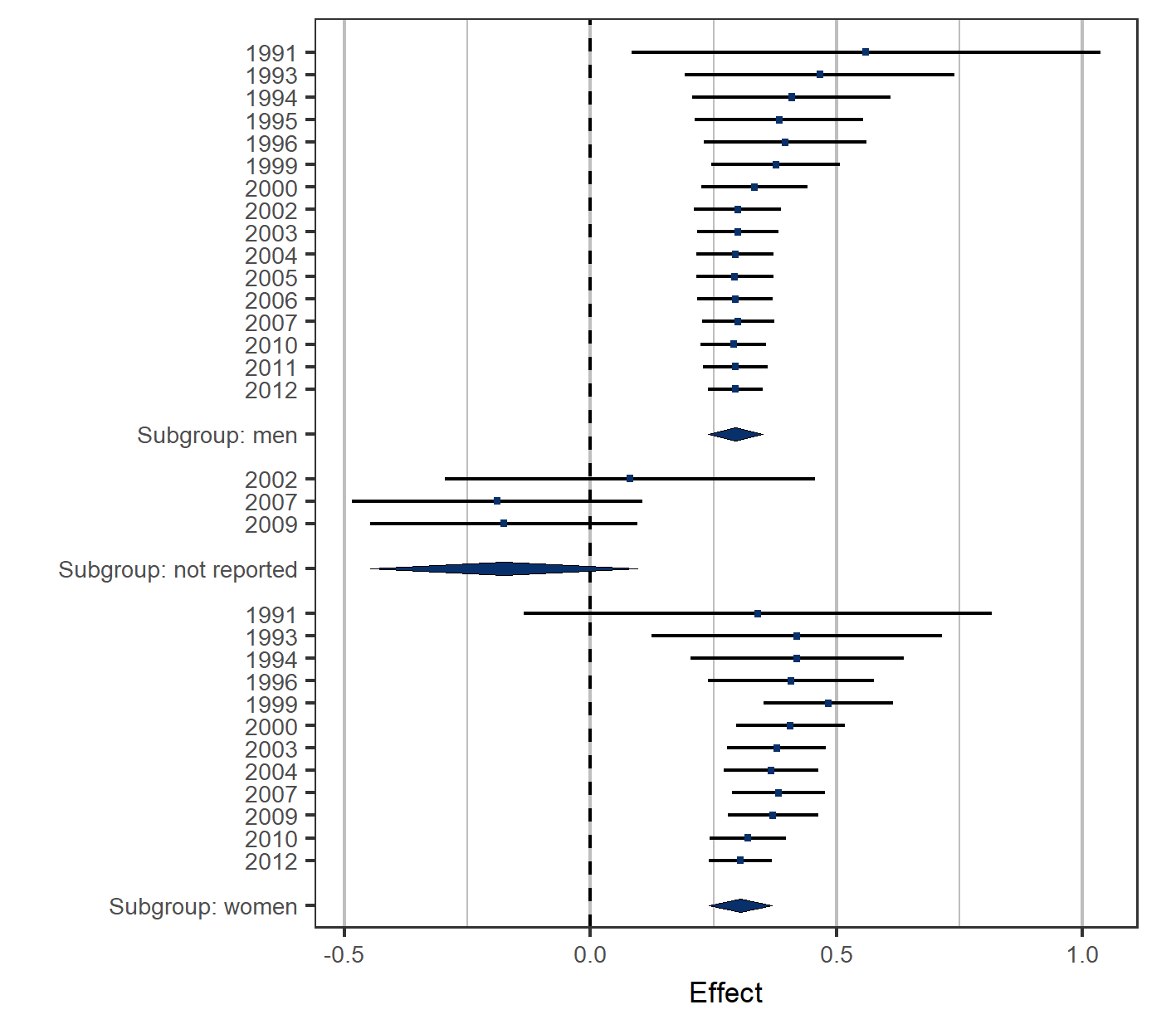 | 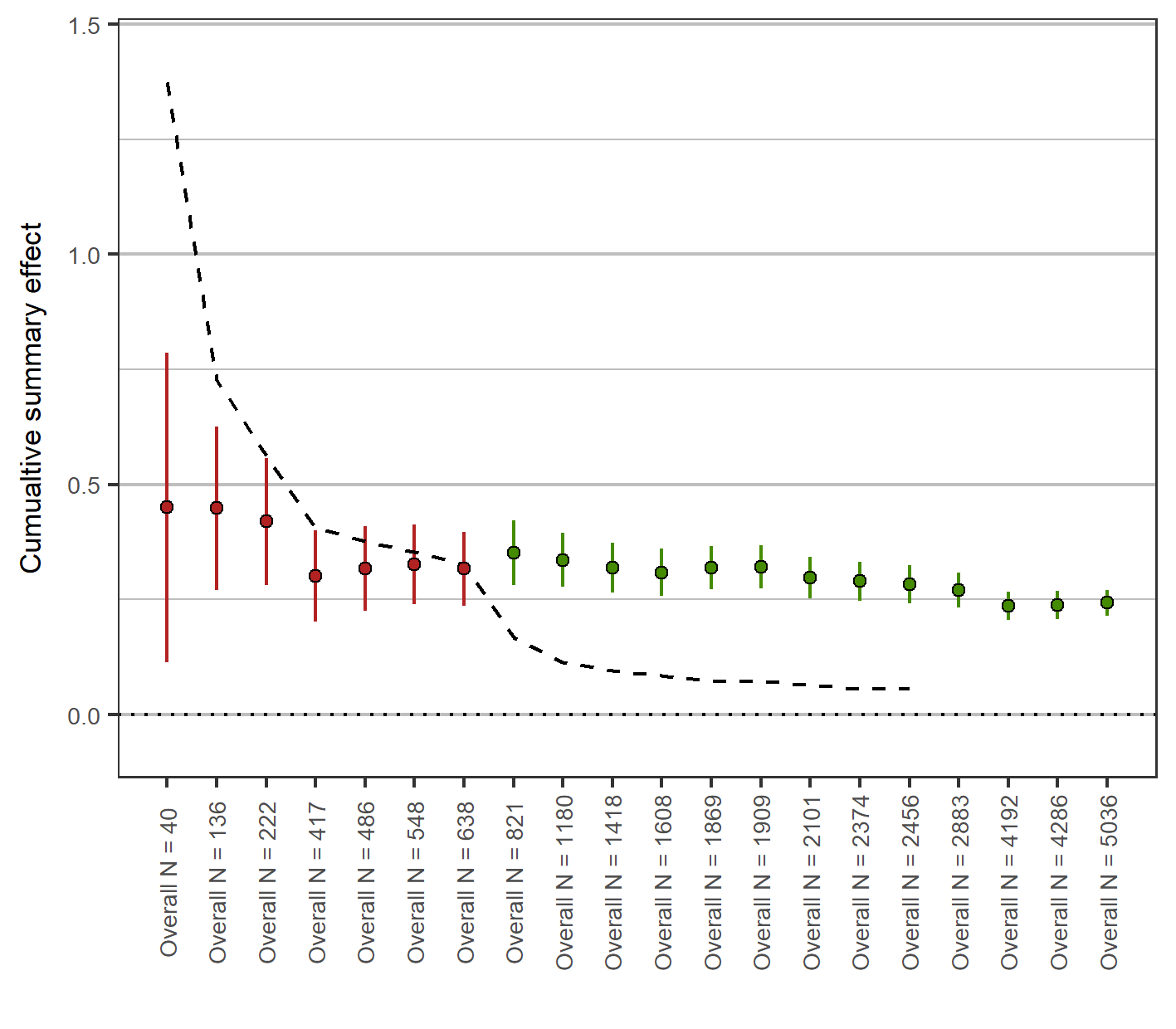 |
| The cumulative meta-analysis plot shows the evolution of the meta-analytic summary plot over time in a summary forest plot (see also [1.2.2]). For each time point, all information available up to that time point is used for the computation of the meta-analytic estimates. The meta-analytic summary effect and its confidence interval is shown separately for each year (Lau, Antman, Jimenez-Silva, Kupelnick, Mosteller, & Chalmers, 1992) or for each information step (i.e., newly available effect estimate; Ioannidis, Contopoulos-Ioannidis, & Lau, 1999). The plot allows examining time trends in the accumulated evidence (e.g., a decline in effect sizes) and the extent of increased precision over time. | The accumulating evidence over time can be visualized differentiated by subgroups. For each study subgroup, a cumulative meta-analysis [5.2] is shown separately in the same display. This plot is a special case of the subgroup forest plot [1.2.1]. For an example application, see Rothstein, Sutton, and Borenstein (2005, pp. 251-252). | Monitoring boundaries represent sequential inferential tests which assess whether all the accumulated evidence available up to a specific time point is sufficient to reject the null hypothesis in question. Such sequential monitoring boundaries have been proposed and visualized in the context of (cumulative) meta-analysis, in order to check whether and when the cumulative evidence is significant and convincing, or still inconclusive (for further details, see Pogue & Yusuf, 1997). |
| Cumulative summary effect ratio plot [5.2.3] | Cumulative heterogeneity plot [5.2.4] | Cumulative Bayes factor plot [5.2.5] |
| 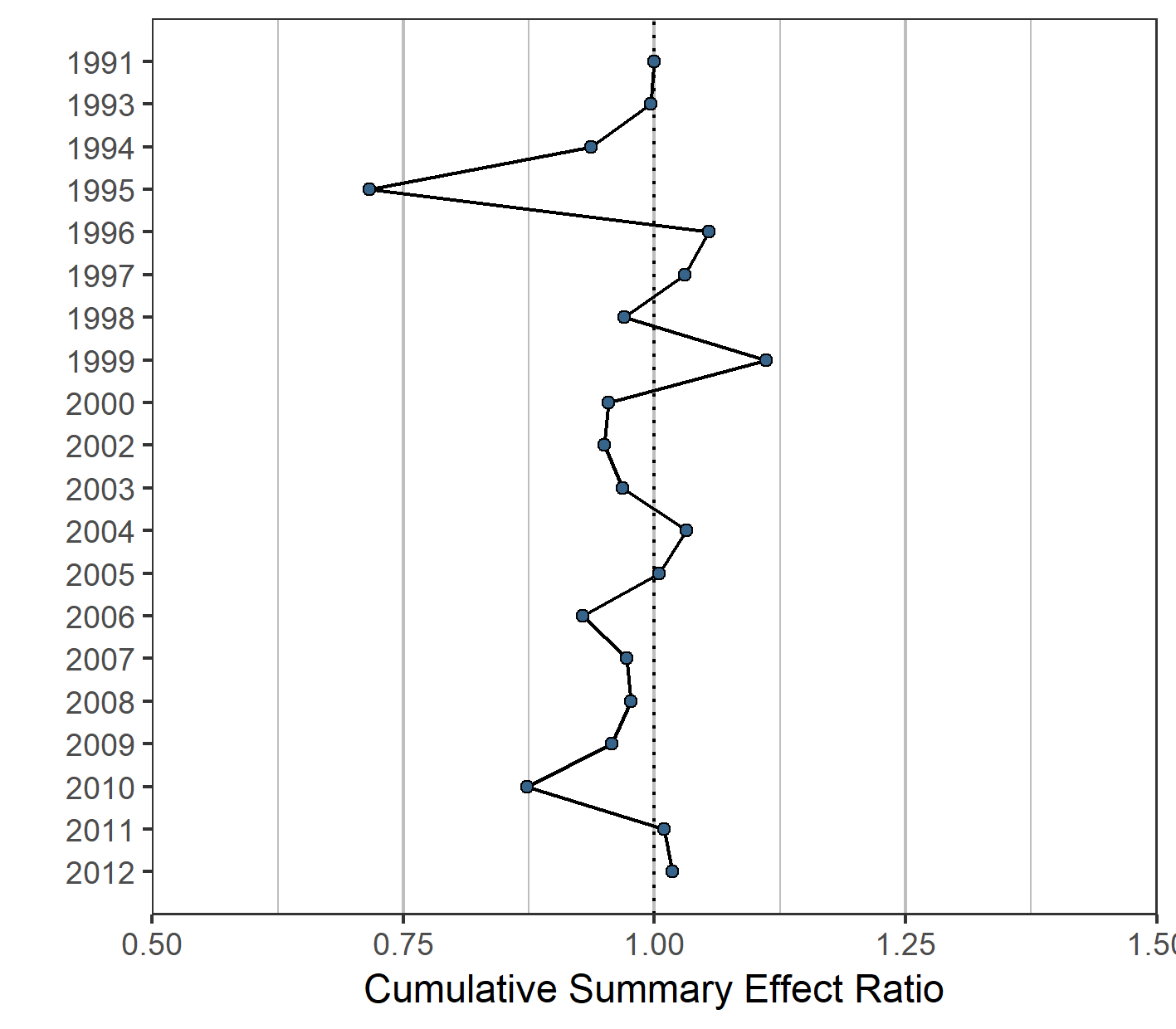 | 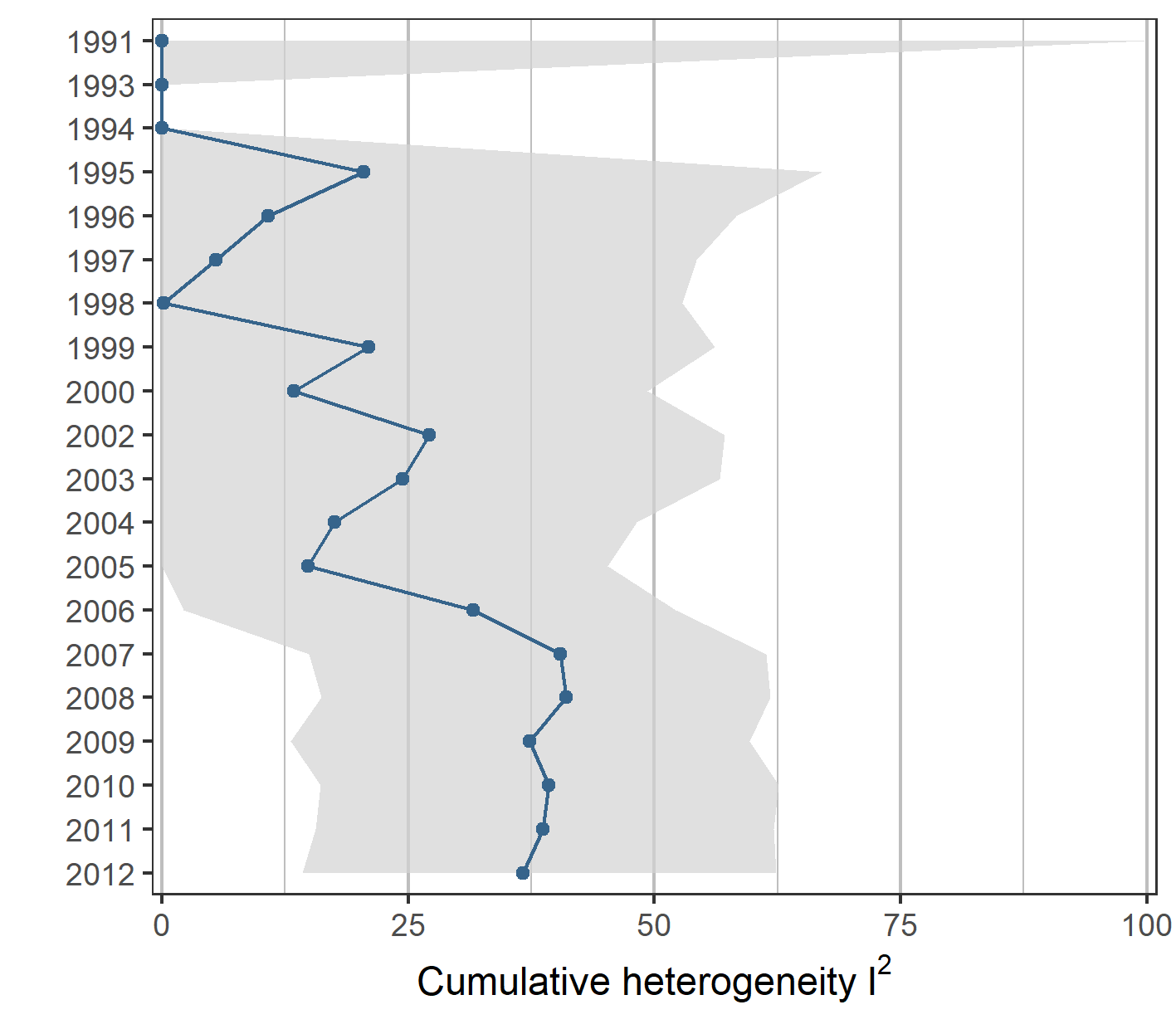 | 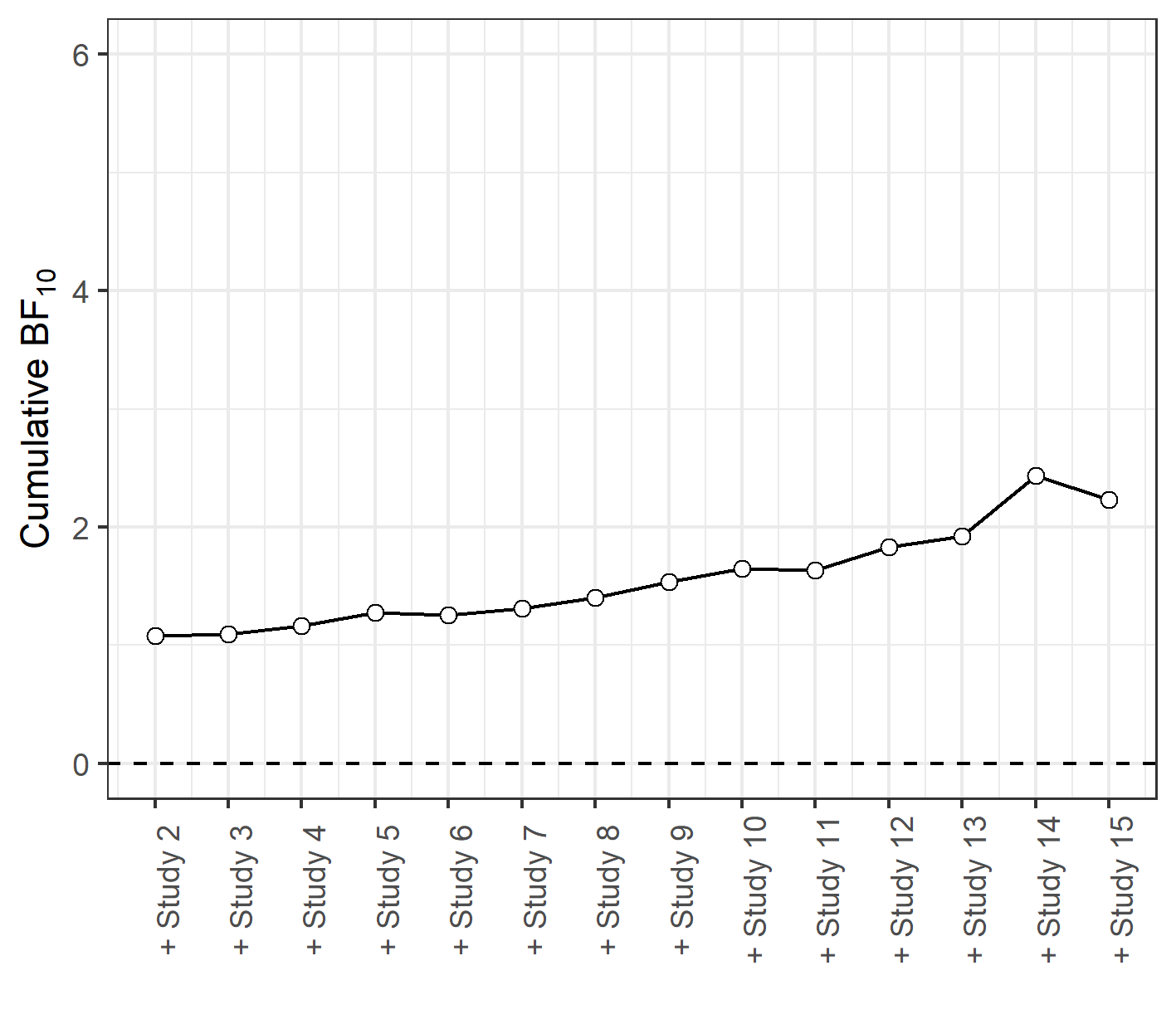 |
| A variant of the cumulative meta-analysis plot [5.2] has been proposed that does not show the absolute cumulative meta-analytic effect at each time-point, but rather the relative change of the meta-analytic effect (Ioannidis, Contopoulos-Ioannidis, & Lau, 1999). For each year or information step (i.e., newly available evidence), the ratio of the meta-analytic summary effect at this time point and the summary effect at the previous time-point are computed. The trajectory of relative changes is plotted from top to bottom in a line plot. | In addition to cumulative meta-analysis plots [5.2], showing the development of the meta-analytic summary effect over time, cumulative heterogeneity plots have been proposed (Villanueva & Zavarsek, 2004). Cumulative heterogeneity plots show the trajectory of meta-analytic between-study heterogeneity parameter estimates (e.g., the *Q* or *I*^2^ statistics) over time. | The accumulation of evidence in the context of Bayesian meta-analysis has been shown using Bayes factors (Scheibehenne, Jamil, & Wagenmakers, 2016). For each additional study sequentially included in the meta-analysis, the corresponding Bayes factor is computed. The trajectory of these (cumulative) Bayes factors over time is visualized in a time-series plot. Conventional categories of strength of evidence (e.g., weak, moderate, and strong evidence) corresponding to specific Bayes factor values have been shown on a second axis or by horizontal reference lines. |

| Predicted Bayes factor for an additional study plot [5.2.6] | Plot of cumulative results [5.3] | Comparison of meta-analyses: Initial vs. subsequent evidence scatterplot [5.4] |
| --- | --- | --- |
| 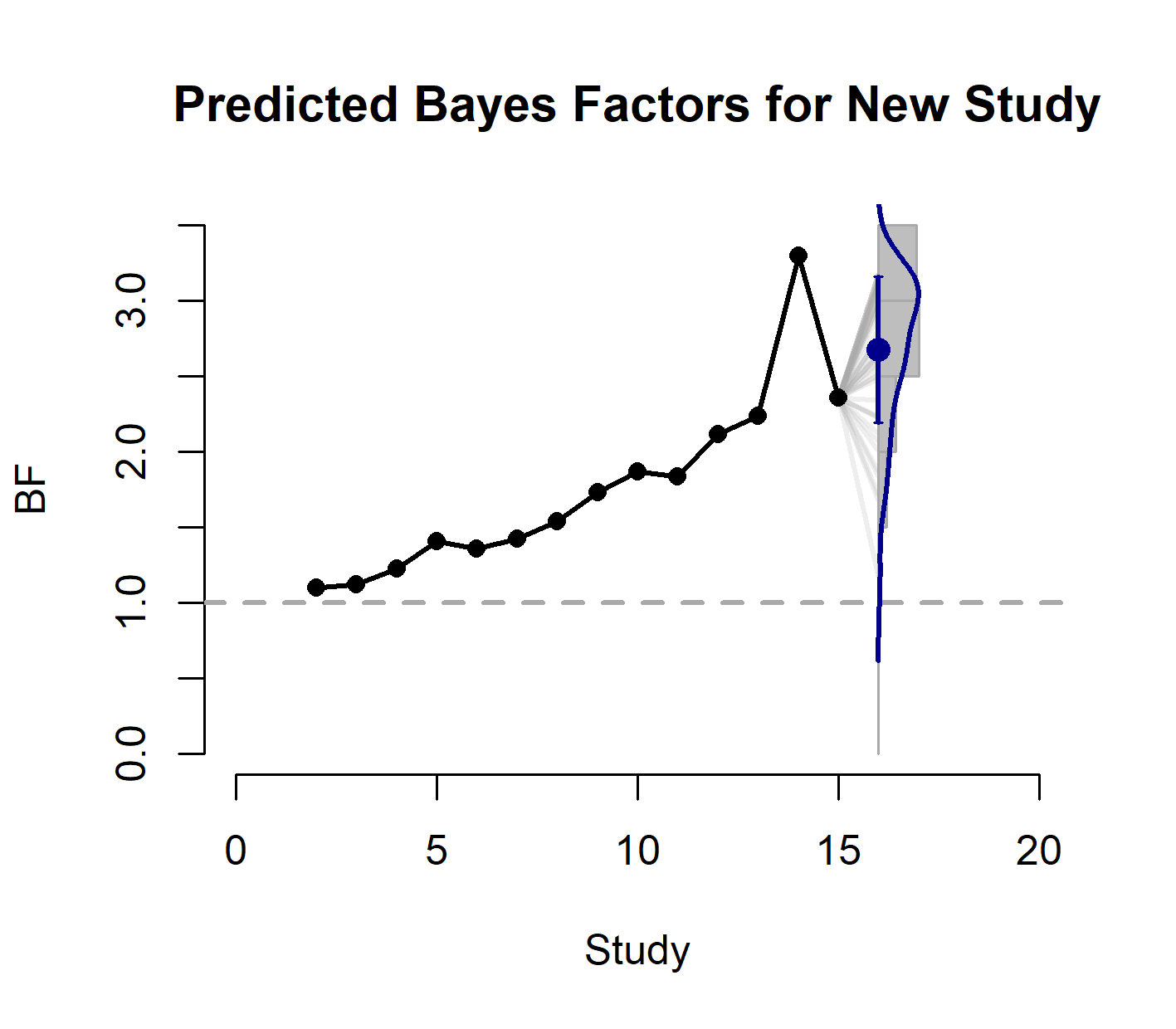 | 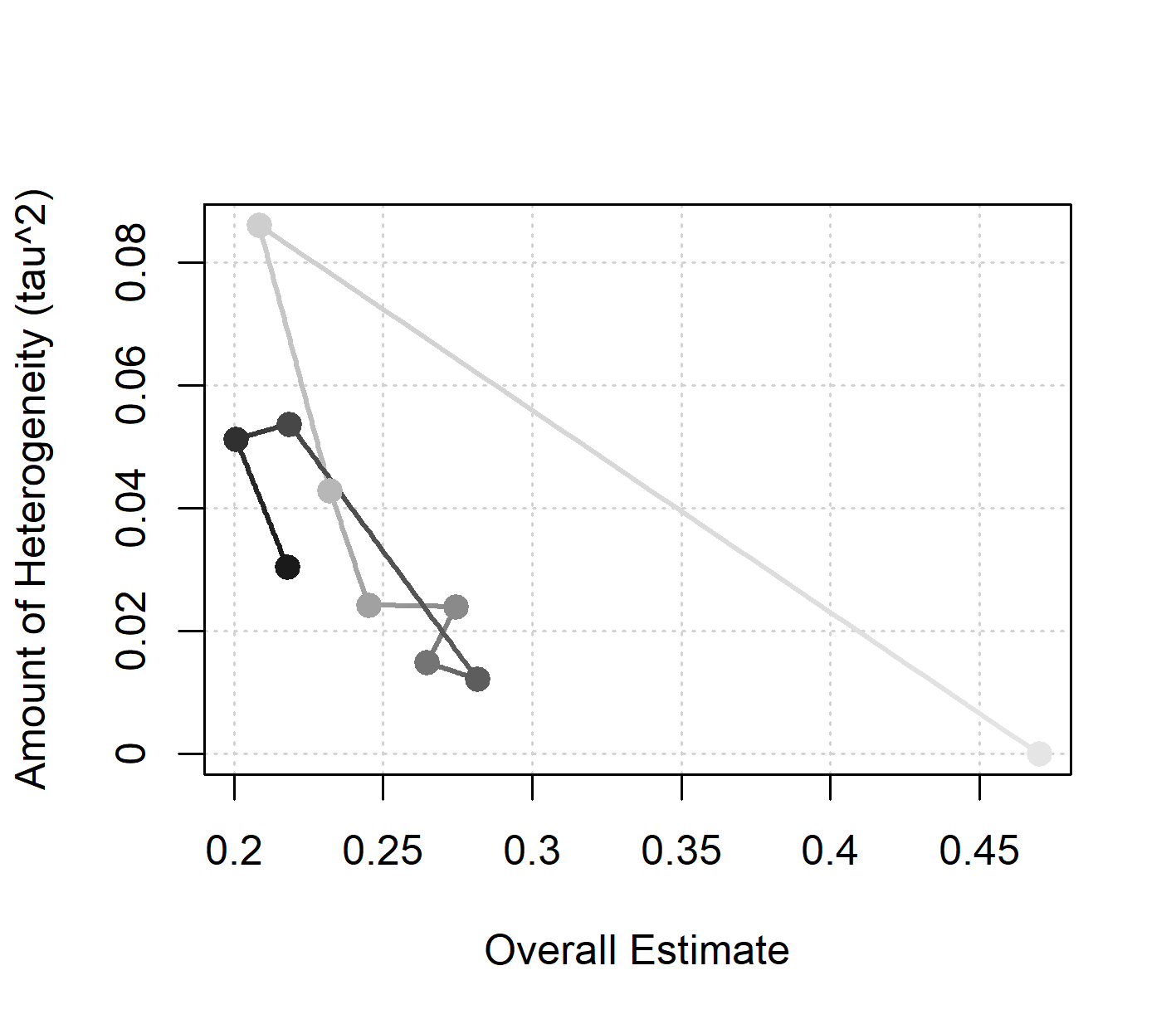 | 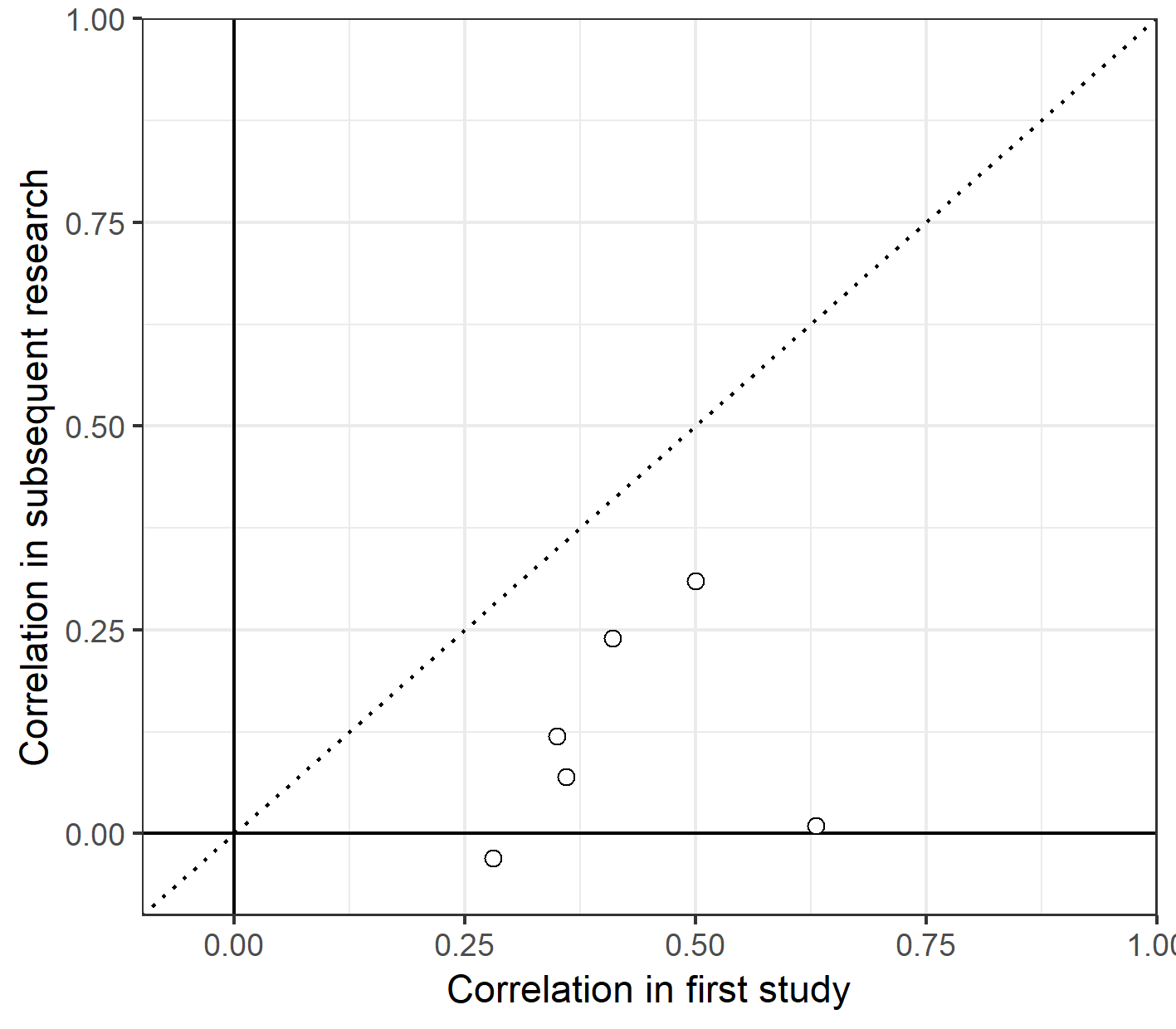 |
| The expected effect of a hypothetical new study on the (cumulative) Bayes factor has been shown in a variant of the cumulative Bayes factor plot [5.2.5]. The plot allows assessing the robustness of the current meta-analytic results to conducting and incorporating a new study. For further details, see Heck, Gronau, and Wagenmakers (2017), who introduced this plot in the context of Bayesian model averaging of fixed-effect and random-effects meta-analytic models (Gronau, van Erp, Heck, Cesario, Jonas, & Wagenmakers, 2017). The above plot and related computations were obtained using the R package metaBMA (Heck, Gronau, & Wagenmakers, 2017). | The plot of cumulative results was added to the R package metafor (Viechtbauer, 2010) in 2015. The plot shows the trajectory of two meta-analytic estimates over time simultaneously in a scatterplot. For each time point, the overall summary effect and the estimated between-study heterogeneity statistic τ^2^ are plotted, considering only evidence available up to this time-point. Results for each time point are connected by lines as a trajectory of results over time. The sequence of results is visualized by shading; early results are shaded in a light color, whereas later results become more and more dark colored. | Time trends of effect sizes in meta-analyses often suggest a decline effect, such that the observed effect size in the first study tends to be larger than effects observed in consecutive replicating studies. A scatterplot has been used to visualize this phenomenon, by showing the effect size observed in the first study in several meta-analyses (x axis), as compared to their meta-analytic summary effect, using all other studies (y axis). A decline in effect sizes indicated visible through meta-analytic summary effects lying under the 45-degree line. For further details and an example application, see Trikalinos and Iaonnidis (2005, pp. 244-247). |

| Histogram [6.1] | Histogram, weighted [6.1.1] | Histogram, subgroups [6.1.2] |
| --- | --- | --- |
| 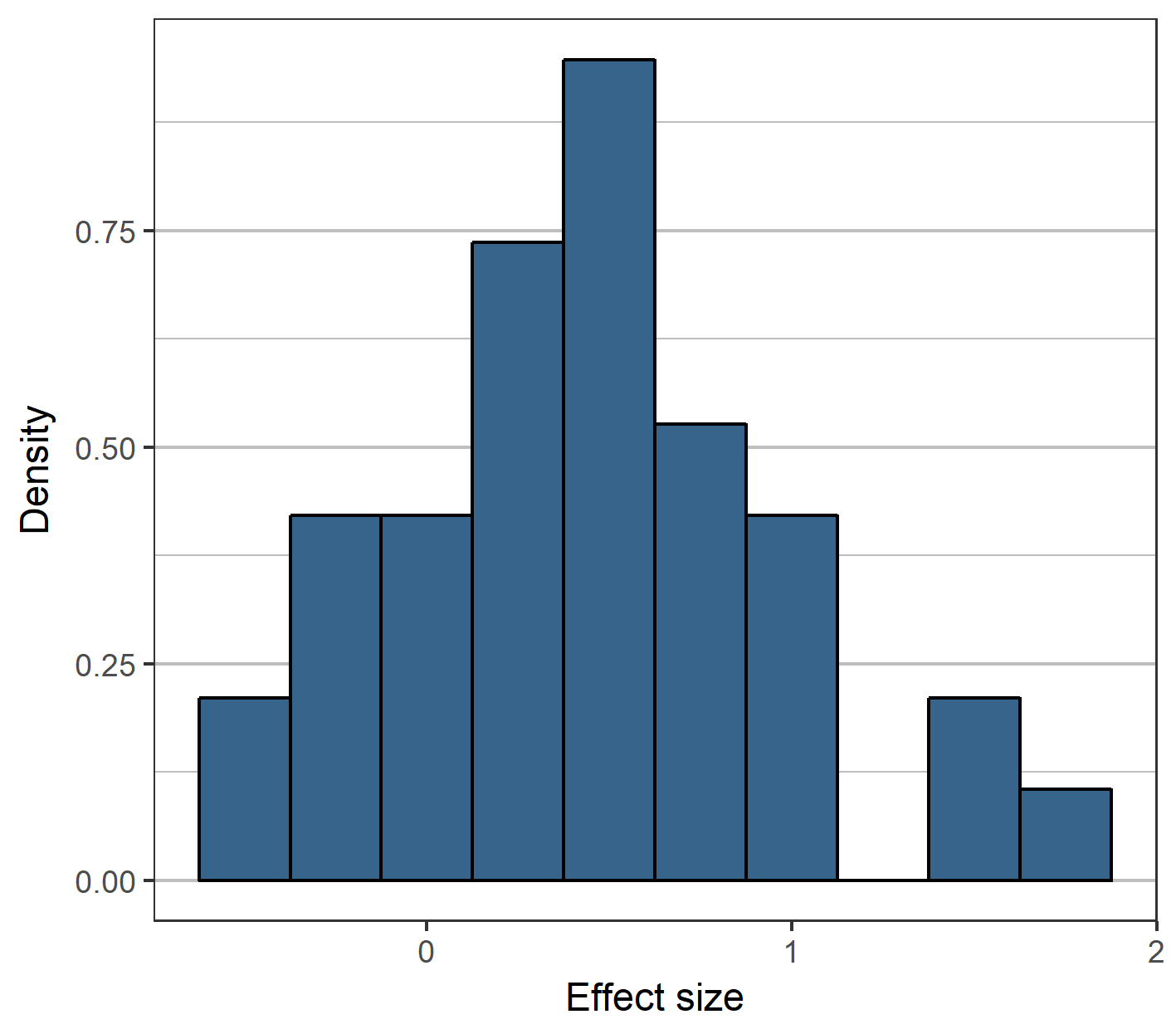 | 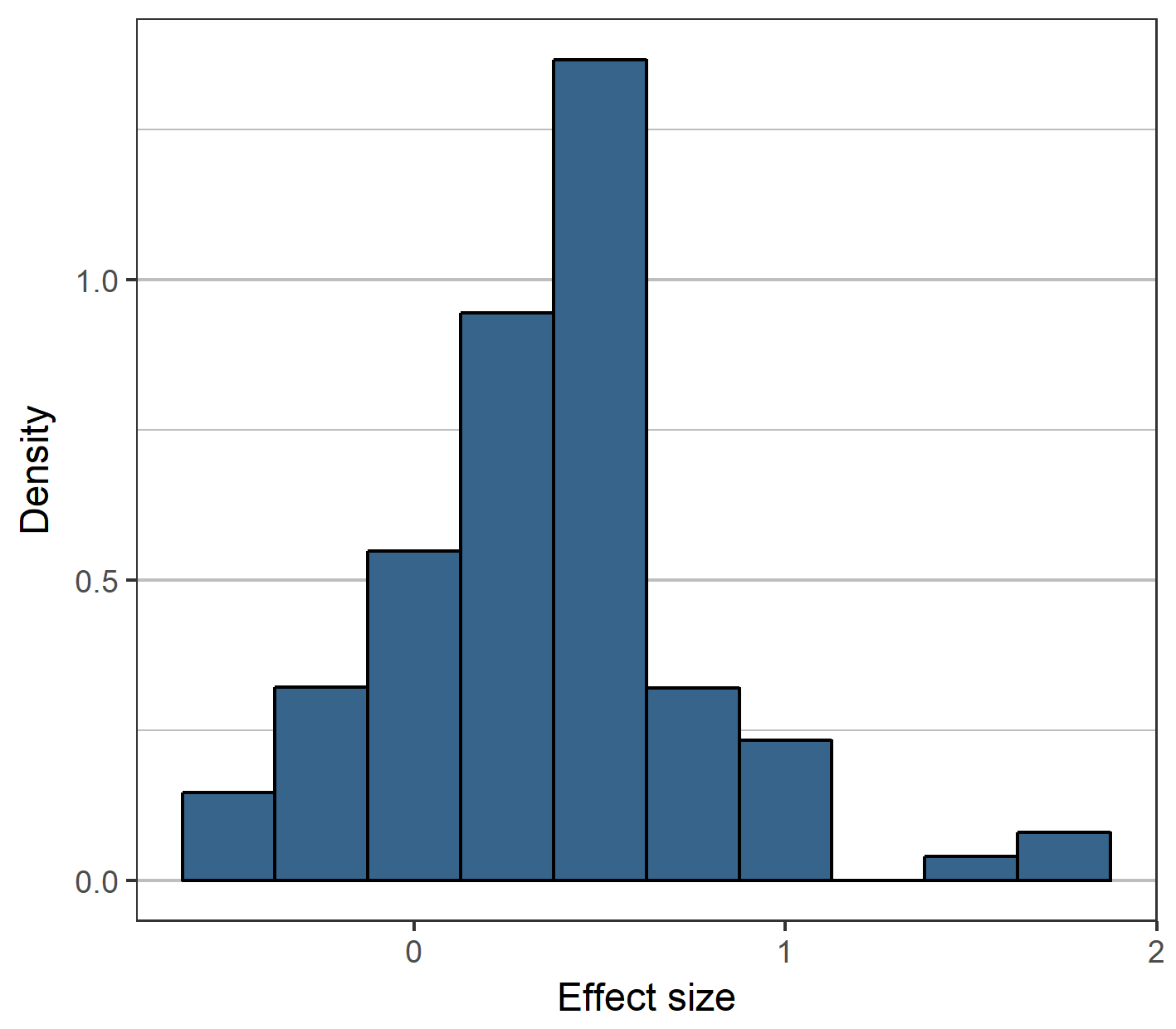 | 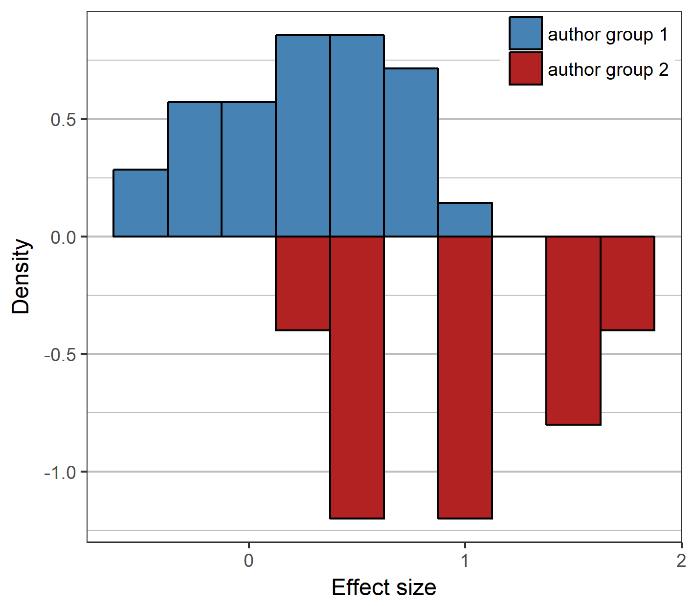 |
| As one of the most prevalent general displays for the visualization of univariate empirical distributions, histograms were also used early on in the context of meta-analysis to visualize effect-size distributions. For effect-size intervals, the empirical distribution is visualized by bars with areas proportional to the observed proportion of observed effect sizes. The number and width of bins can alter the appearance of the plot considerably and has to be carefully chosen by the user. The use of histograms for effect sizes in meta-analysis has been discouraged in the past, because no information on the standard error or meta-analytic weight is provided (e.g., Anzures-Cabrera & Higgins, 2010). However, weighted histograms [6.1.1] or histograms using standardized effect sizes are alternatives which incorporate this information. An early published example in the context of meta-analysis can be found in Light and Pillemer (1984, p. 97). | The weighted histogram is a way to visualize the empirical effect-size distribution, but at the same time incorporate information on the meta-analytic weight (commonly as a function of the variance of effect sizes). For the unweighted histogram, the areas of the bars for an effect-size interval are proportional to the observed proportion of effect sizes. In the weighted histograms, these areas are proportional to the proportion of the total weight of all effects in the meta-analysis. Therefore, precise estimates visually contribute more than imprecise estimates, and typical effect size values are visually more pronounced. Meta-analytic software to create weighted histograms was available not later than in 2000 (Rosenberg, Adams, & Gurevitch, 2000). | The distribution of effect sizes within subgroups (e.g., effect sizes from different author groups) have been visualized with histograms. Histograms for each subgroup can be stacked or arranged back-to-back for this purpose (as in the above example). Meta-analytic software to create subgroup histograms was available not later than in 2000 (Rosenberg, Adams, & Gurevitch, 2000). |
| Boxplot [6.2] | Boxplot, weighted [6.2.1] | Stem-and-leaf display [6.3] |
| 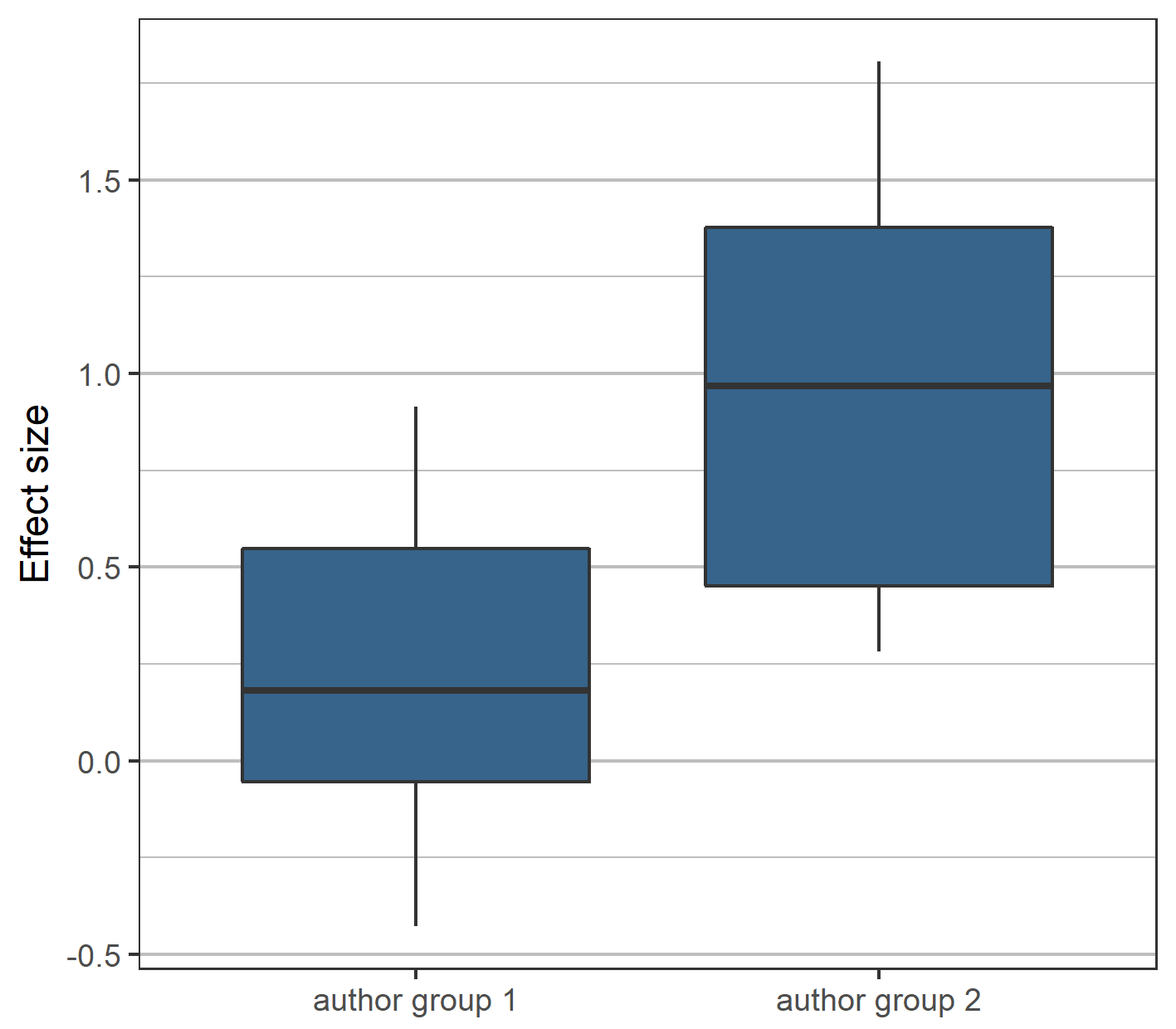 | 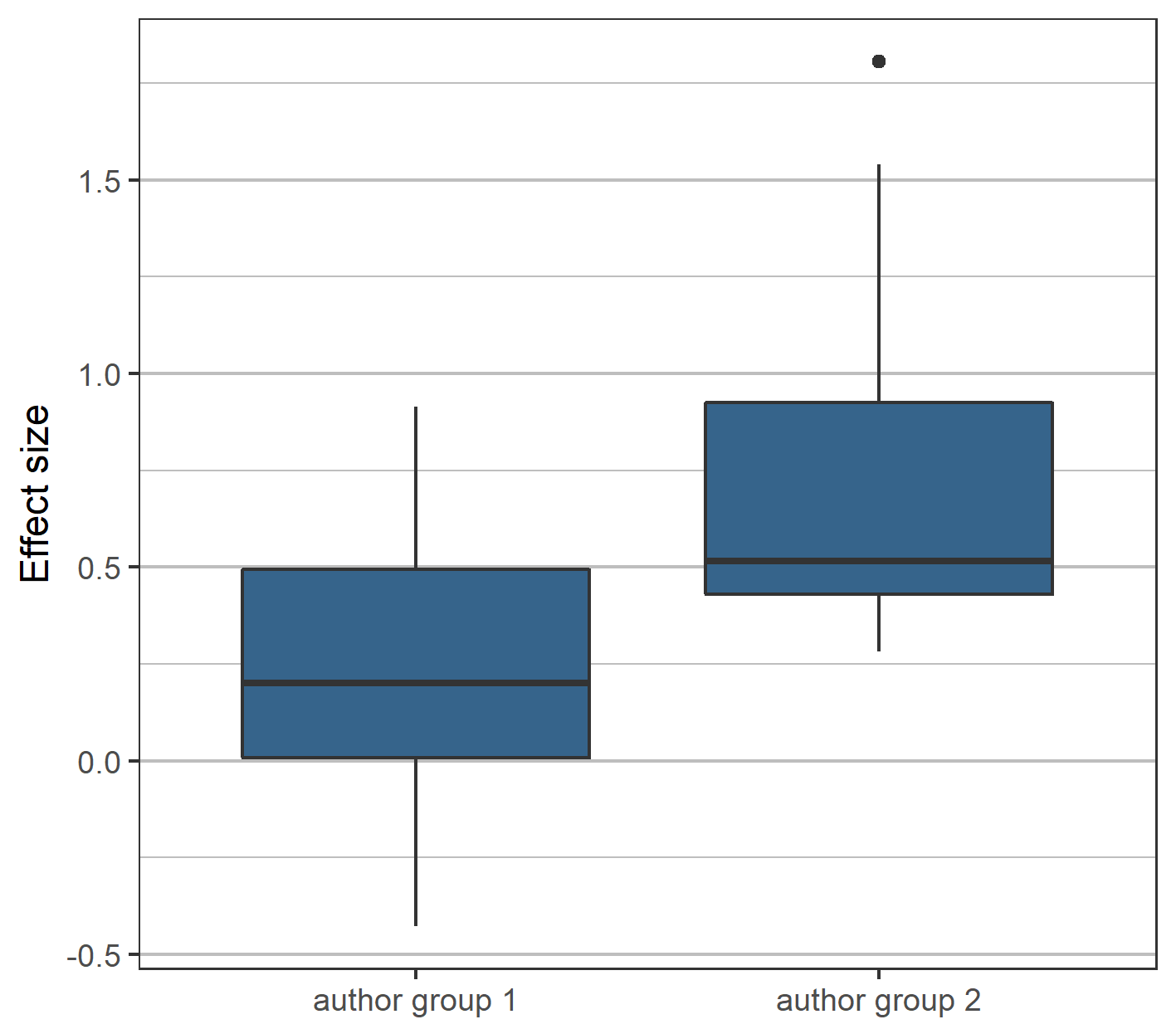 |  |
| Boxplots are a further general display for statistical data (Tukey, 1977), which have been used early on in the context of meta-analyses to visualize effect-size distributions (e.g., Glass, McGraw & Smith, 1981, p. 156). Boxplots summarize the distribution of empirical data with five quantile statistics (0%, 25%, 50%, 75%, and 100%) and two measures of the spread of the data (interquartile range; full range of the data). Boxplots have been used to compare effect-size distributions of more than one study subgroup. Like histograms [6.1], the use of boxplots for effect sizes in meta-analysis has been questioned in the past, because no information on the standard error or meta-analytic weight is provided (e.g., Anzures-Cabrera & Higgins, 2010). | Weighted boxplots are an alternative to unweighted boxplots [6.2]. They allow incorporating meta-analytic weights (e.g., inverse variances) in the visualization of the observed effect-size distribution. In weighted boxplots, quantiles are shown with respect to the meta-analytic weights, instead of effect sizes. For instance, the box shows in which range the intermediate effect sizes with 50% of the total meta-analytic weight lie. Software to construct weighted boxplots in the context of meta-analysis has been available not later than in mid-2000s (Bax, Yu, Ikeda, Tsuruta, & Moons, 2006). | Like the histogram [6.1] and the boxplot [6.2] the stem-and-leaf display is another general statistical display used in meta-analysis. The steam-and-leaf display shows for each study-effect size the first one or two digits on the left-hand side (the stem), and the following digit on the right hand-side (the leaf). It therefore bins the data in stems, similar to the histogram [6.1], with the number of leaves showing the frequency of study-effect sizes per bin. One advantage of the stem-and-leaf display, when compared with the histogram, boxplot, or density plots is that the actual raw effect sizes are shown and extractable. A potential disadvantage is that stem-and-leaf-display might be less suited for very large meta-analyses with hundreds of effect sizes (then binning is necessary), but this should seldom be the case in the context of meta-analysis. A further potential disadvantage is that meta-analytic weights are not displayed. For an early example of its application in meta-analysis, see Rosenthal (1984, p. 111). |

| Stem-and-leaf display, subgroups [6.3.1] | Dot plot [6.4] | Density plot [6.5] |
| --- | --- | --- |
|  | 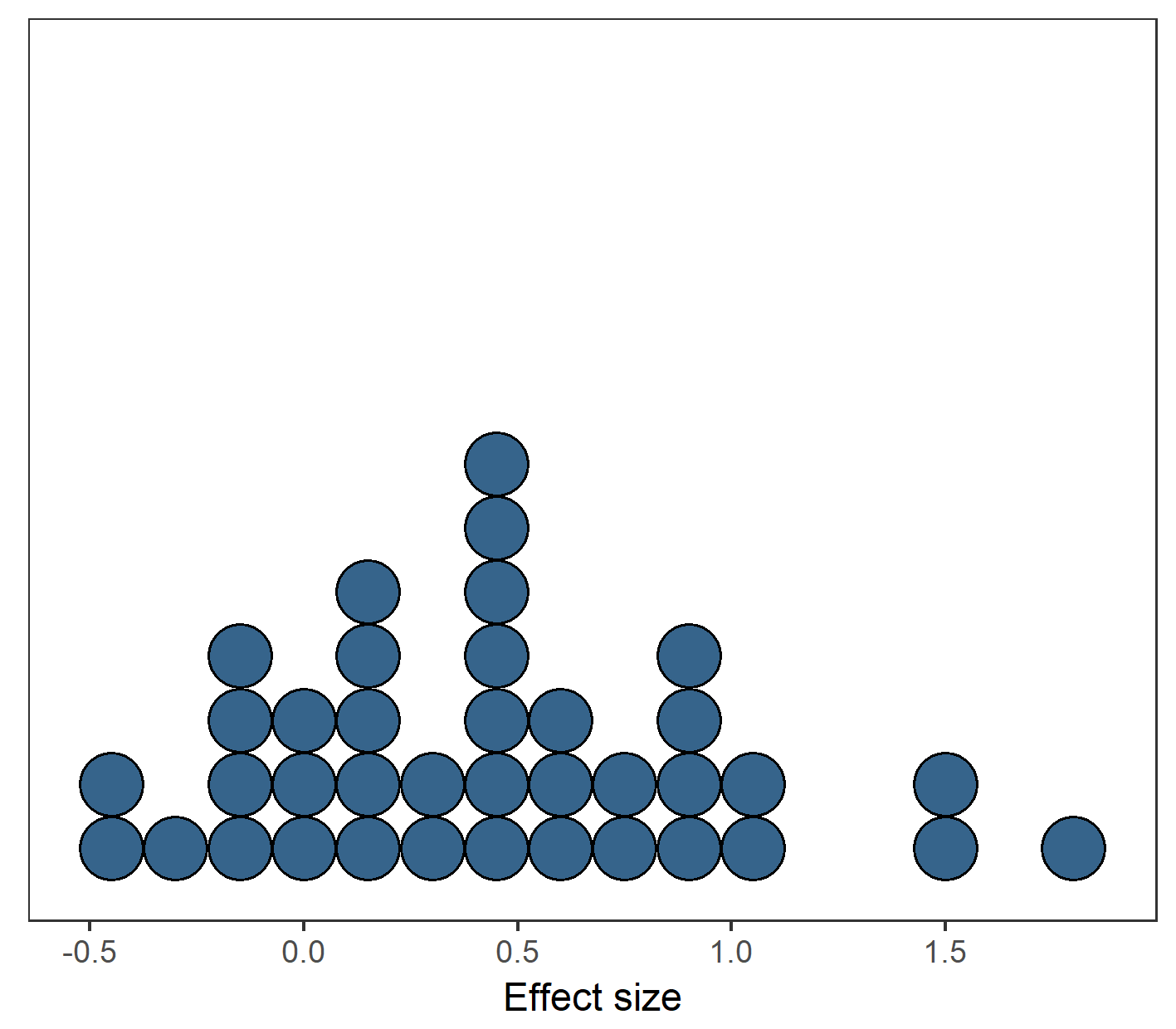 | 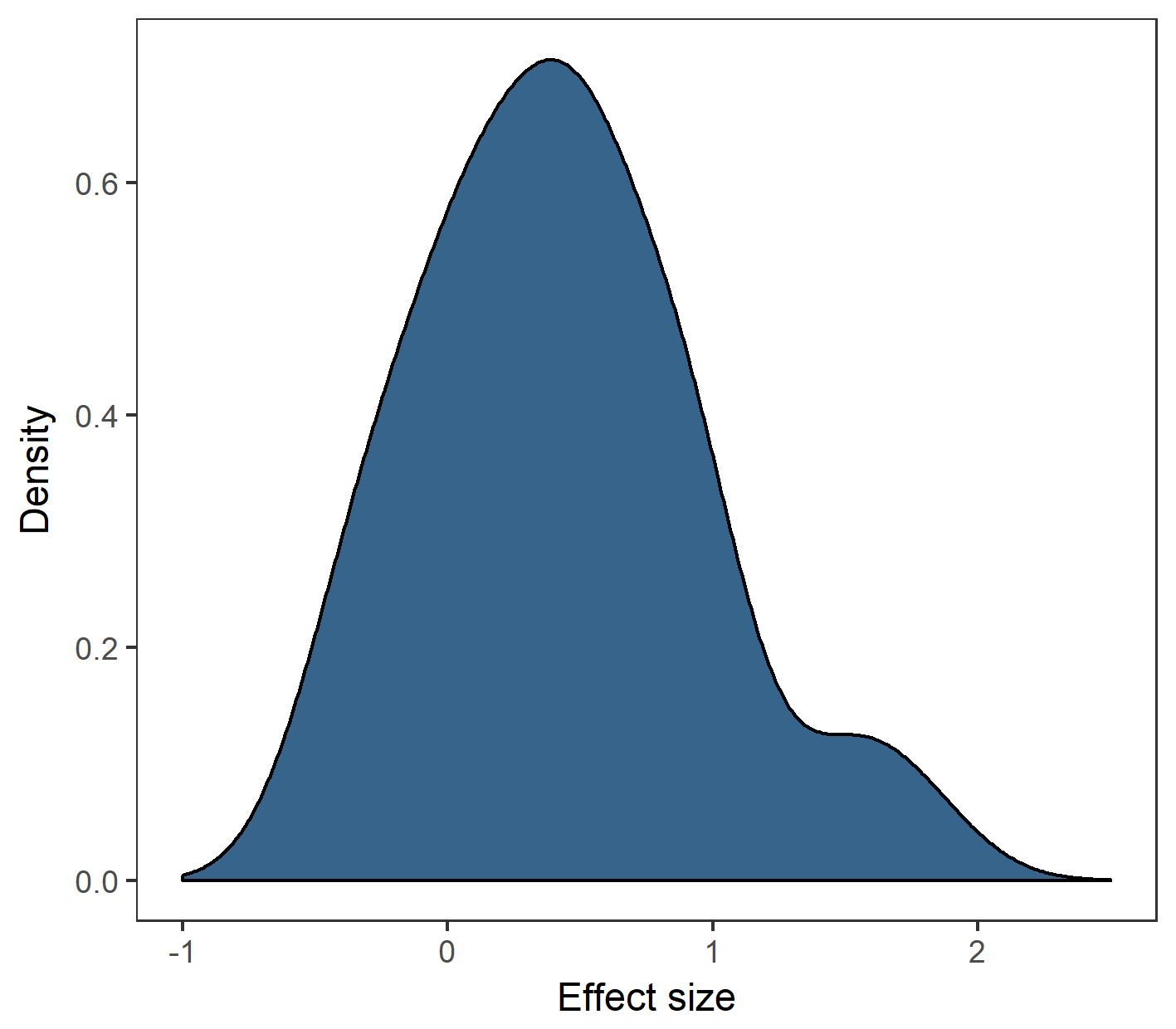 |
| A variant of the steam-and-leaf display allows comparing effect-size distributions of two subgroups of interest. Two stem-and-leaf displays – one for each subgroup – are arranged back-to-back and share the same stem. An example for the use of this variant in the context of meta-analysis can be found in Cooper and Hedges (1994, p. 447). | The dot plot is a simple, easy to understand, display which has been used early on to display meta-analytic data (Light & Pillemer, 1984, p. 59). For effect-size intervals, dots are stacked up for each observed study-effect size. Compared to its alternatives (e.g., Histograms [6.1] or density plots [6.5]), the dot plot might be less suited for very large meta-analyses (with hundreds of studies), and meta-analytic weights cannot be displayed. | Density plots have been used to visualize the empirical distribution of effect sizes. Density plots estimate the underlying (probability) density distribution in a smooth way, using kernel functions (e.g., the normal distribution density). The smoothness of the density estimate can be varied by a user-specified parameter. For further general details on density plots, see Chambers, Cleveland, Kleiner, and Tukey (1983). An early example of its use in the context of meta-analysis can be found in Galbraith (1988). |

| Density plot, weighted [6.5.1] | Density plot, subgroups [6.5.2] | Collection of study-effect likelihoods [6.6] |
| --- | --- | --- |
| 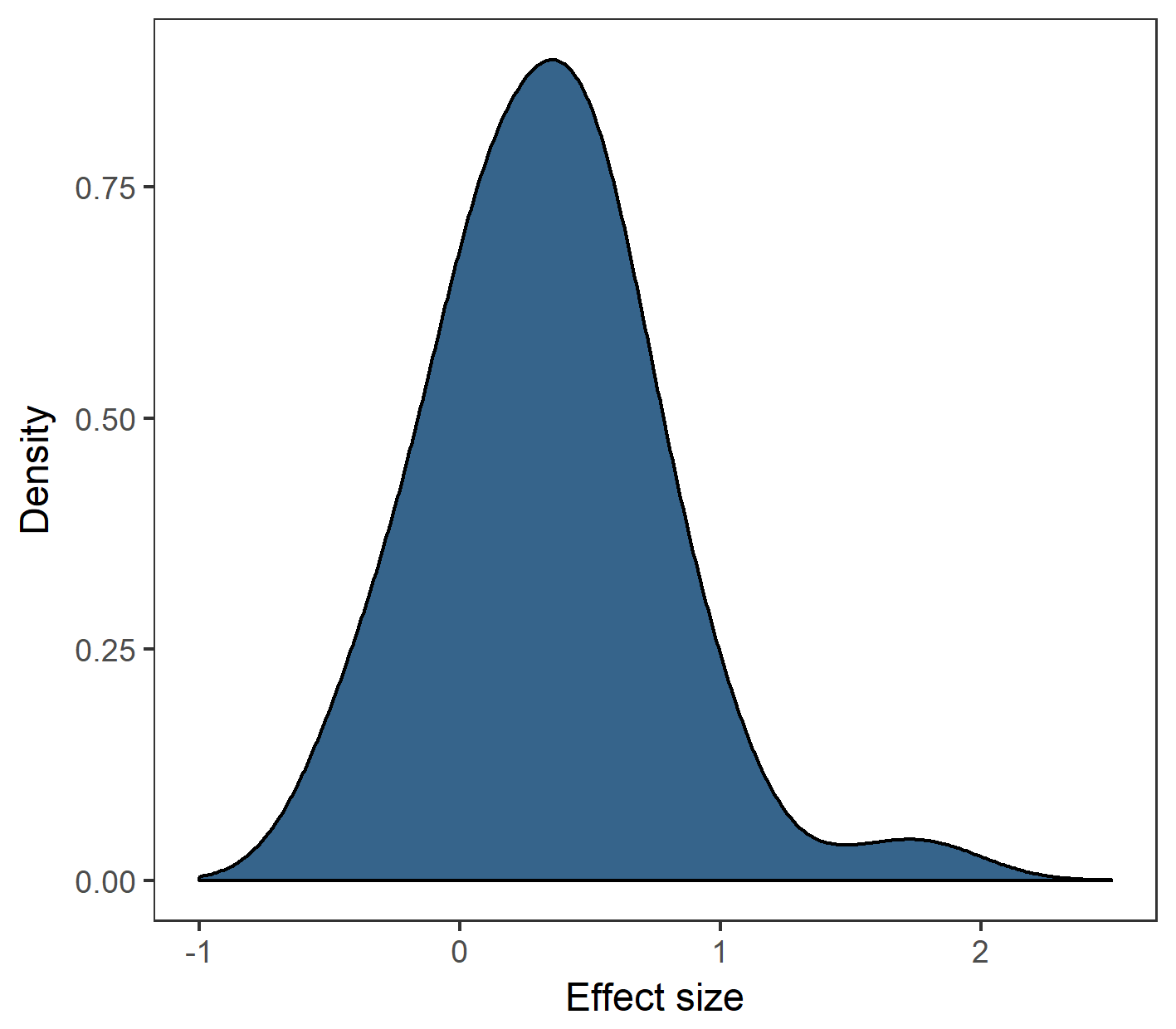 | 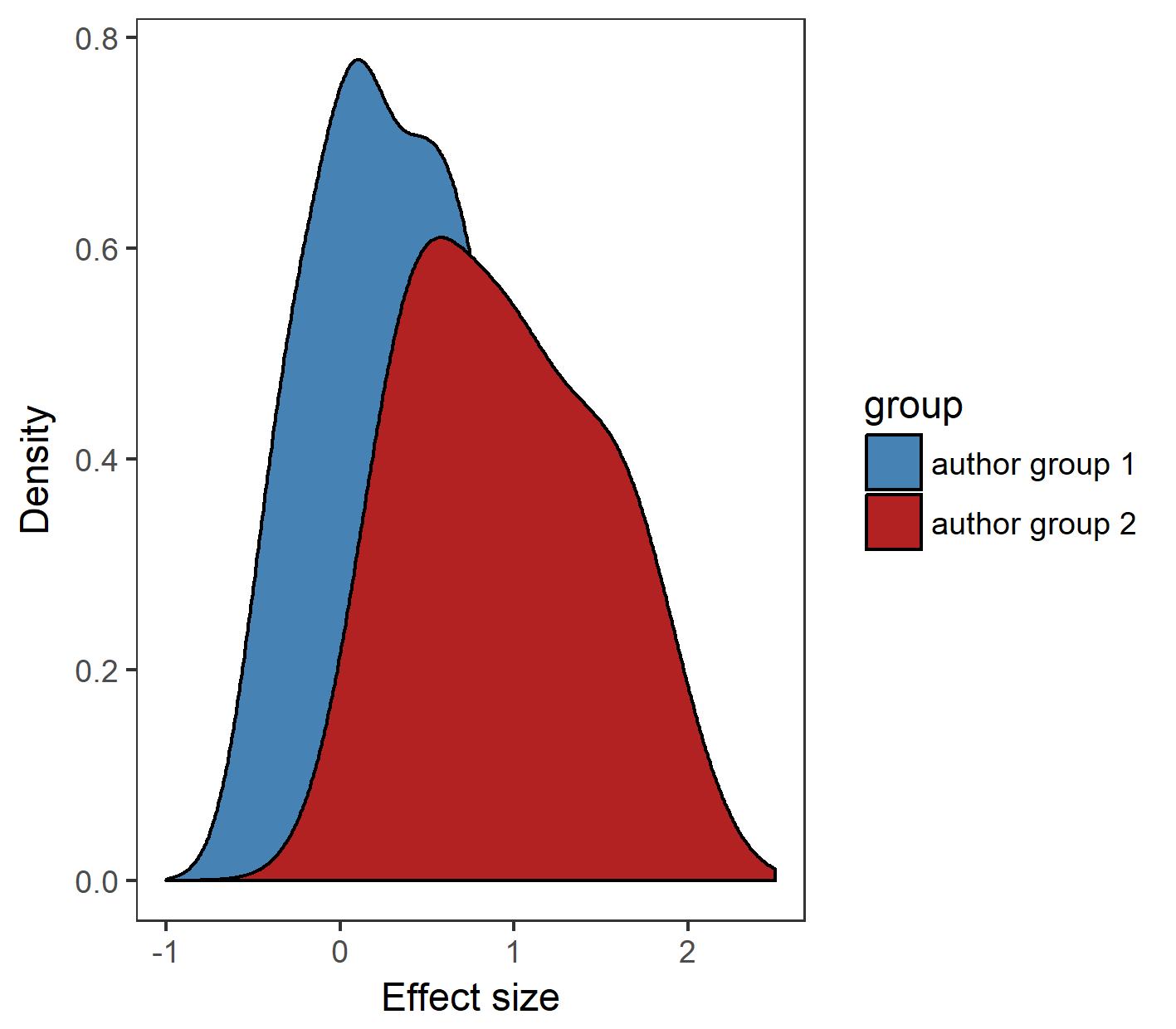 | 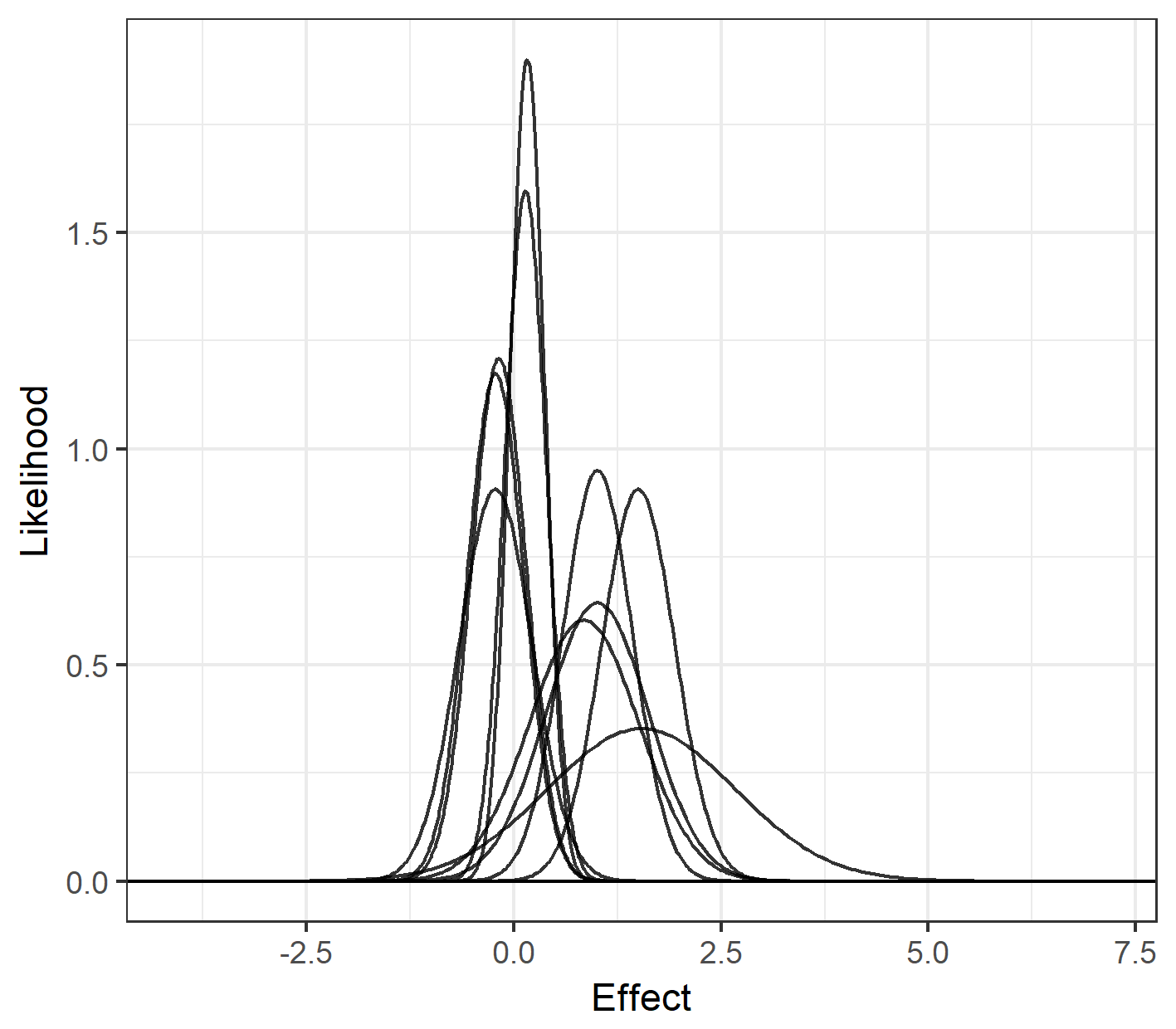 |
| Similar to the histogram [6.1] and the boxplot [6.2], a weighted variant of the density plot exists and has been used to incorporate meta-analytic weights in the visualization of the empirical effect-size distribution. Weights are incorporated by choosing the width of kernel functions proportional to the meta-analytic weight. More concentrated kernel functions are used for effect sizes with larger weight (e.g., normal distribution with smaller variance), and wider kernel functions for effect sizes with smaller weight (e.g., normal distributions with larger variance). For an early example of its use in the context of meta-analysis, see Galbraith (1988). | The estimated density distributions of two or more distinct study subgroups have been shown with subgroup density plots. For each subgroup, separate densities are estimated and either overlain (as in the above example) or shown back-to-back. For an example of the application of this variant, see Roberts and Stanley (2005, p. 222). | Effect sizes have been visualized using study-specific likelihoods (van Houwelingen, Zwinderman, & Stijnen, 1993). For each observed effect size and its variance, as well as an assumption about its distribution (e.g., normal), the likelihood function dependent on the true population effect parameter is drawn. The resulting plot shows likelihoods for each study. Effect-size regions where many study-specific likelihoods are simultaneously high, can implicitly be taken as regions of plausible true effect values, given the data (see also raindrop plot [1.2.4] and confidence distribution plot [1.2.6]). |

| Normal quantile-quantile (Q-Q) plot of effect sizes [6.7] |
| --- |
| 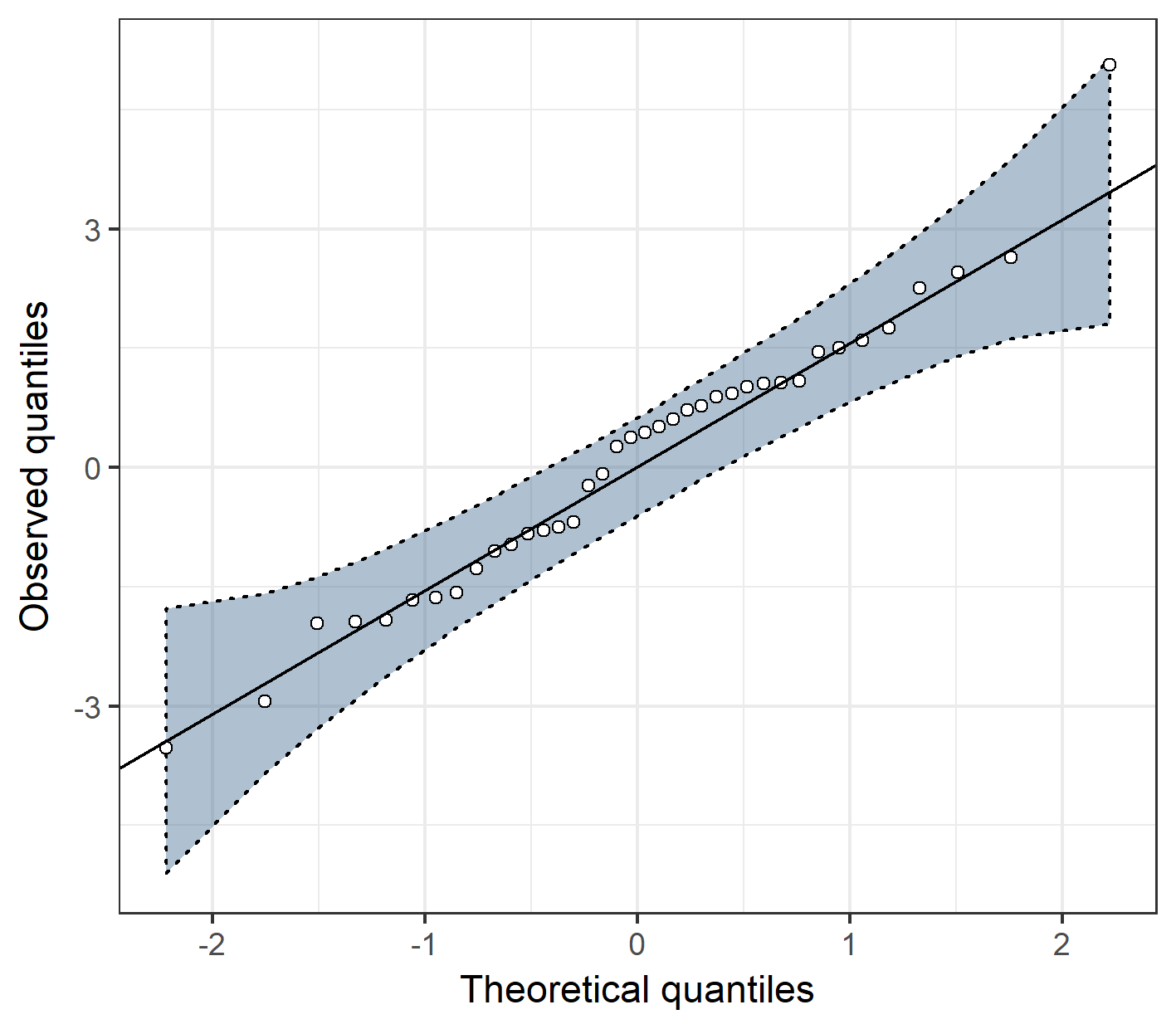 |
| Normal quantile-quantile (Q-Q) plots have been recommended as general diagnostic display for meta-analysis (Wang & Bushman, 1998). Observed effect-size quantiles are plotted against expected quantiles under normality. Large deviations from the plotted line (with confidence bands helping this evaluation) indicate violations of the normality assumption. In the context of meta-analysis, the Q-Q plot was introduced as alternative to the funnel plot [2.1] to assess normality, heterogeneity, as well as publication bias (Wang & Bushman, 1998). Heterogeneity is indicated by bends in the curve of effect sizes. A long tail in the expected direction might indicate publication bias. For further general details regarding Q‑Q plots, see Chambers, Cleveland, Kleiner, and Tukey (1983). |

| Bar chart [7.1] | Pie chart [7.2] | Line/dot chart with continuous covariate [7.3] |
| --- | --- | --- |
| 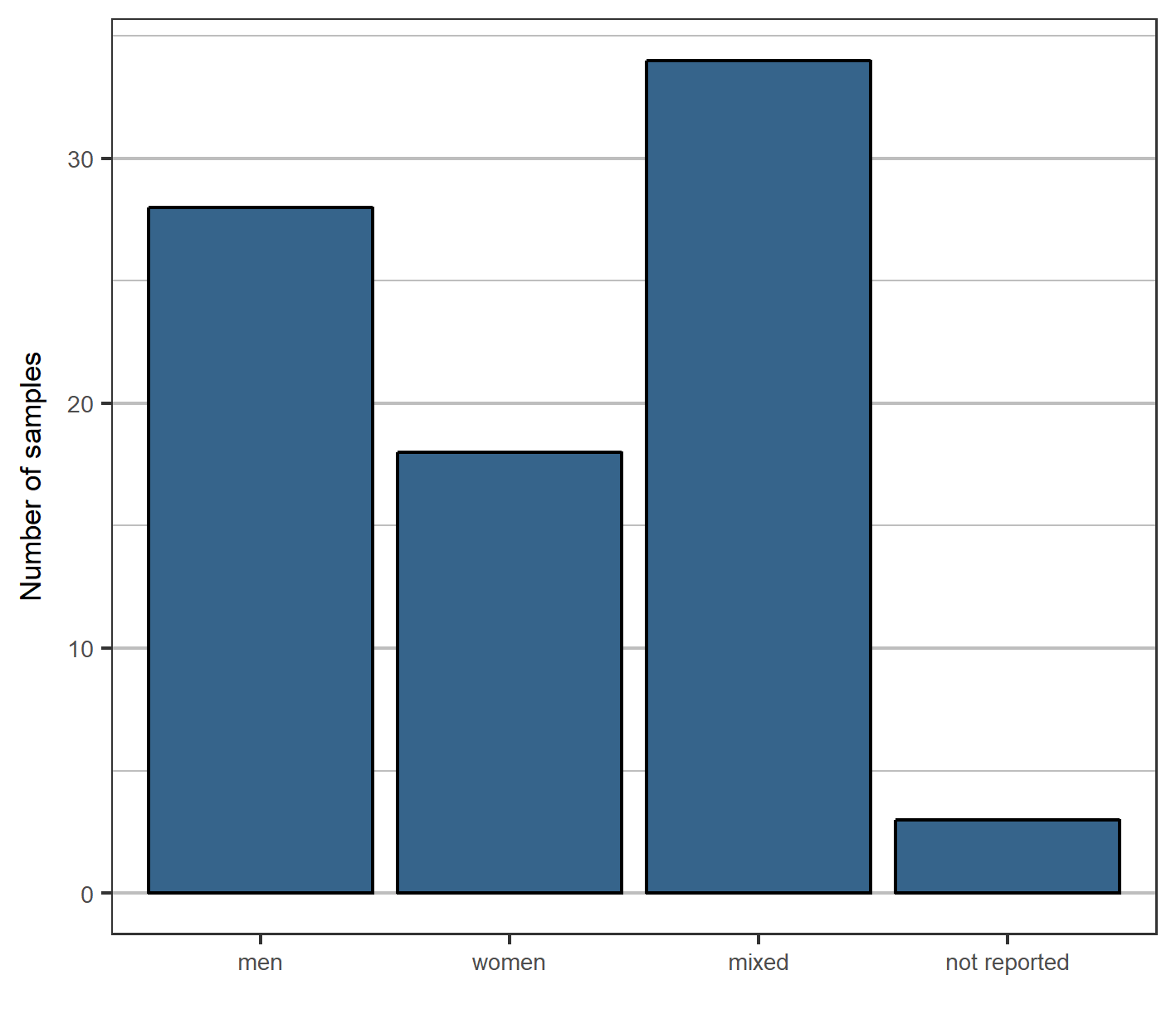 | 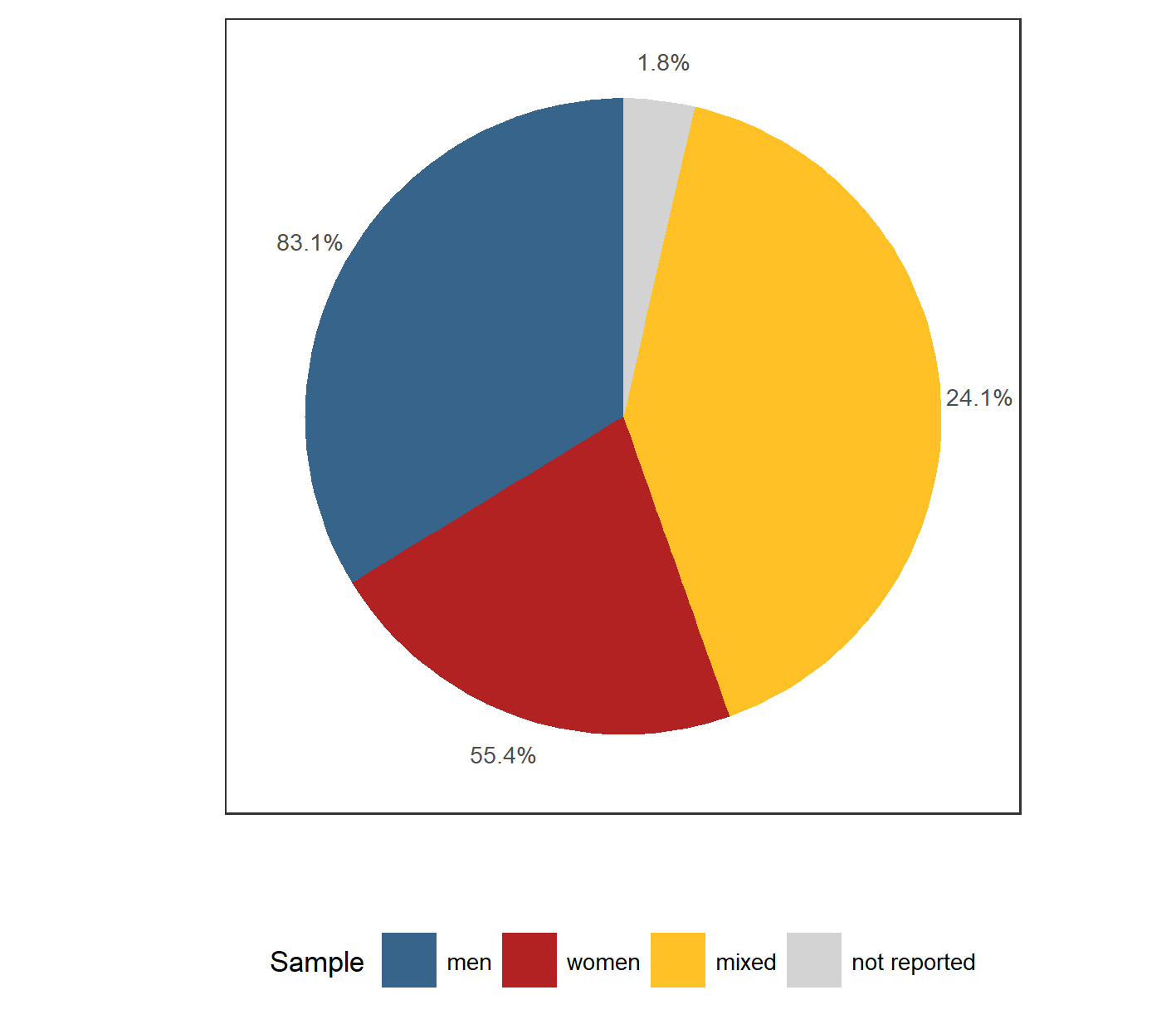 | 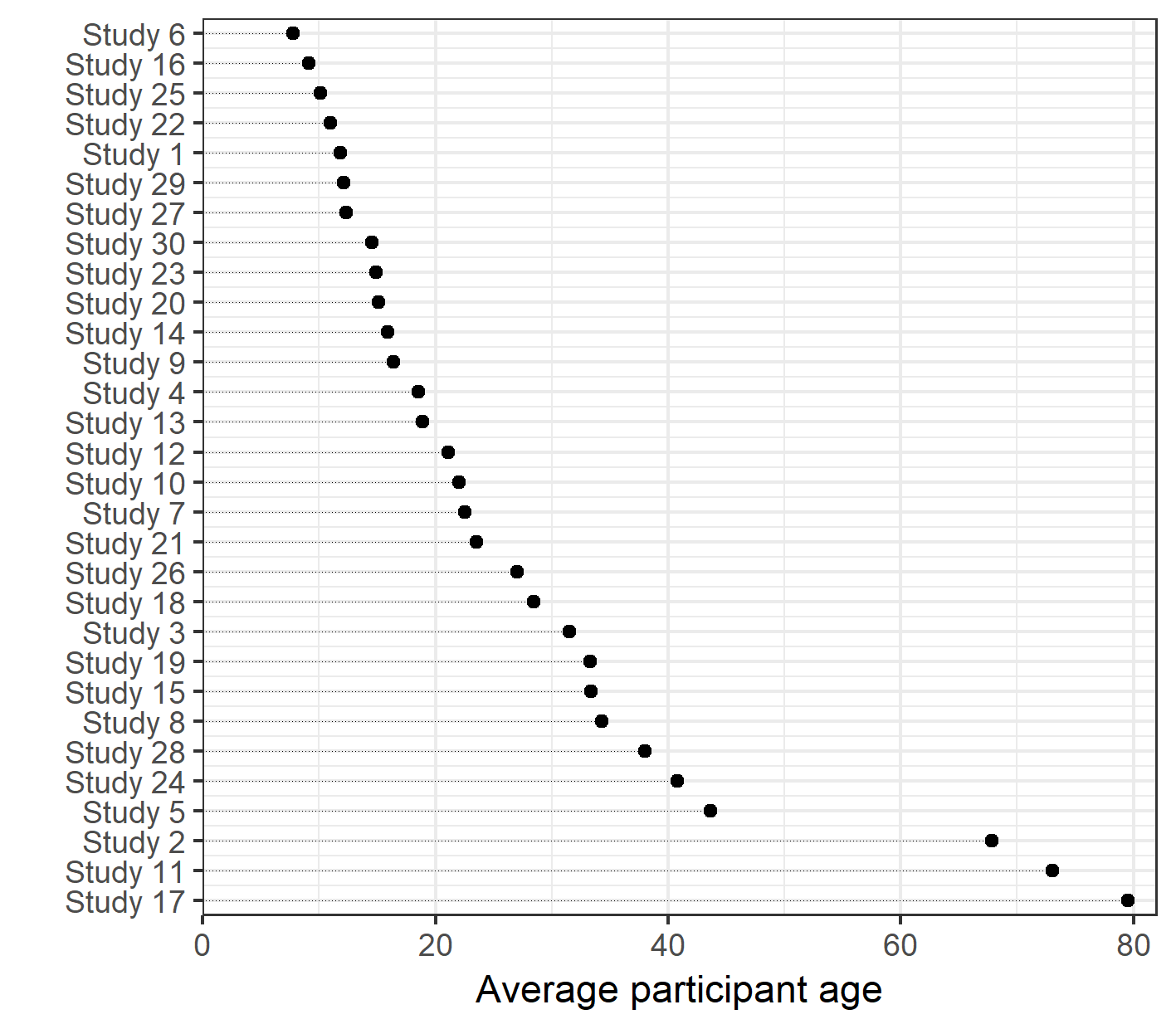 |
| Bar charts have been used in the context of meta-analysis to visualize the distribution of categorical study features. The height of the bars either shows the absolute or relative frequency of different categories. Side-by-side or stacked bar charts can be used to show the distribution of a categorical study feature for two or more study subgroups. | The pie chart is a classic display of categorical statistical data. Pie charts are widely and generally seen inferior to bar charts [7.1], because the angles of pie segments in the pie chart are harder to discern than the height of the bars in bar charts (see, e.g., Cleveland & McGill, 1984). The pie chart has been used in the context of meta-analysis to visualize the distribution of discrete study features (e.g., Bettany-Saltikov, 2012, p. 120). | Dot charts have been used in the context of meta-analysis to visualize the distributions of continuous study covariate values. For each study, the covariate values are shown by dots and (optionally) lines. Sorting studies by the covariate value in an increasing or decreasing order helps to assess the variance and skewness of the covariate distribution. |

| Risk of bias plot [7.4] | Risk of bias summary plot [7.4.1] | Harvest plot [7.5] |
| --- | --- | --- |
| 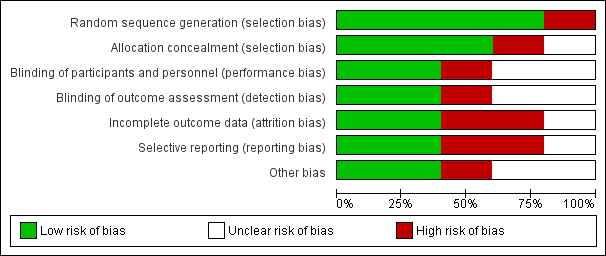 | 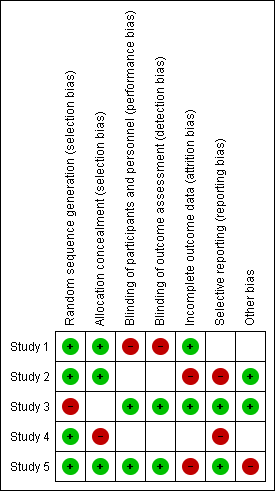 |  |
| The risk of bias plot summarizes the results of risk of bias assessments for a set of studies (Higgins & Green, 2008, pp. 202-206). For each form of bias assessed, the proportion of studies judged to have low, unclear, and high risk of bias are shown within bar charts. The above plot was created using the software RevMan (version 5.3; Cochrane Collaboration, 2014). | The risk of bias summary plot shows the risks of bias assessments on the study level (Higgins & Green, 2008, pp. 202-206). For each form of bias assessed and each study the judged risk for this bias (low, unclear, or high) is shown in a matrix display. The distribution of risks over all studies (i.e., rows in the matrix display) is depicted in the risk of bias plot [7.4]. The above plot was created using the software RevMan (version 5.3; Cochrane Collaboration, 2014). | The harvest plot has been developed to summarize a potentially diverse set of studies and evidence for which standard forest plots are not suitable (Ogilvie et al., 2008). The harvest plot is a matrix display that orders and shows studies according to different treatments examined, study characteristics, and observed effect direction (negative, no effect, positive effect). In the example of Ogilvie et al. (2008), available studies in each cell are represented by bars with their height proportional to the suitability of the study design and the shading indicating the hardness of behavioral outcome measures used. Additionally, for each study the number of quality criteria that are met is annotated. A modified version of the harvest plot, including indicators for study quality – by additional shading, positions according to statistical significance, and additional annotations for the type of study design, sample size, and study identifiers – has been proposed and used as well (Crowther, Avenell, MacLennan, & Mowatt, 2011). |

| PRISMA flow chart [7.6] | Comparison of meta-analyses: Veritas plot [7.7] | Error matrix display [7.8] |
| --- | --- | --- |
| 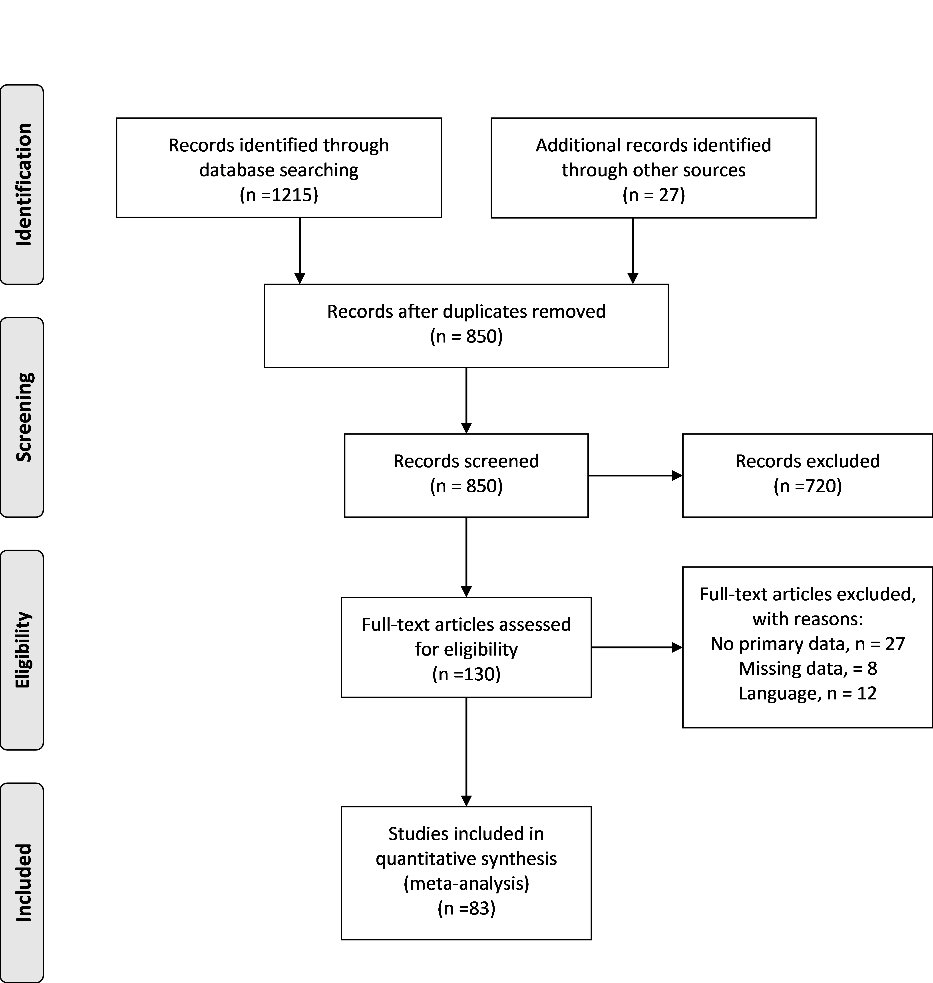 | 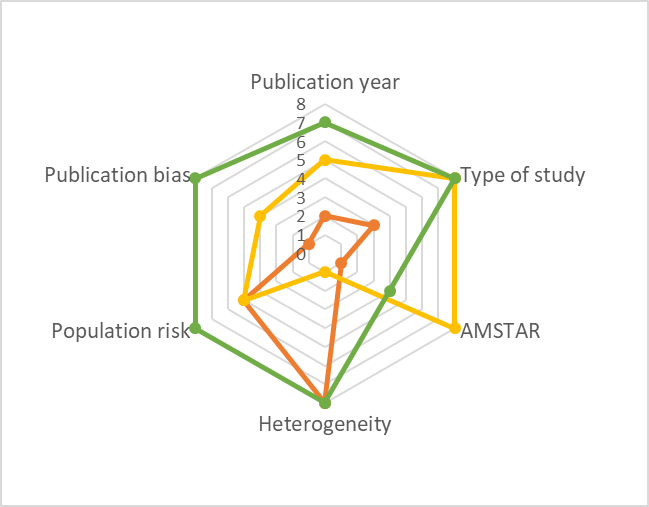 | 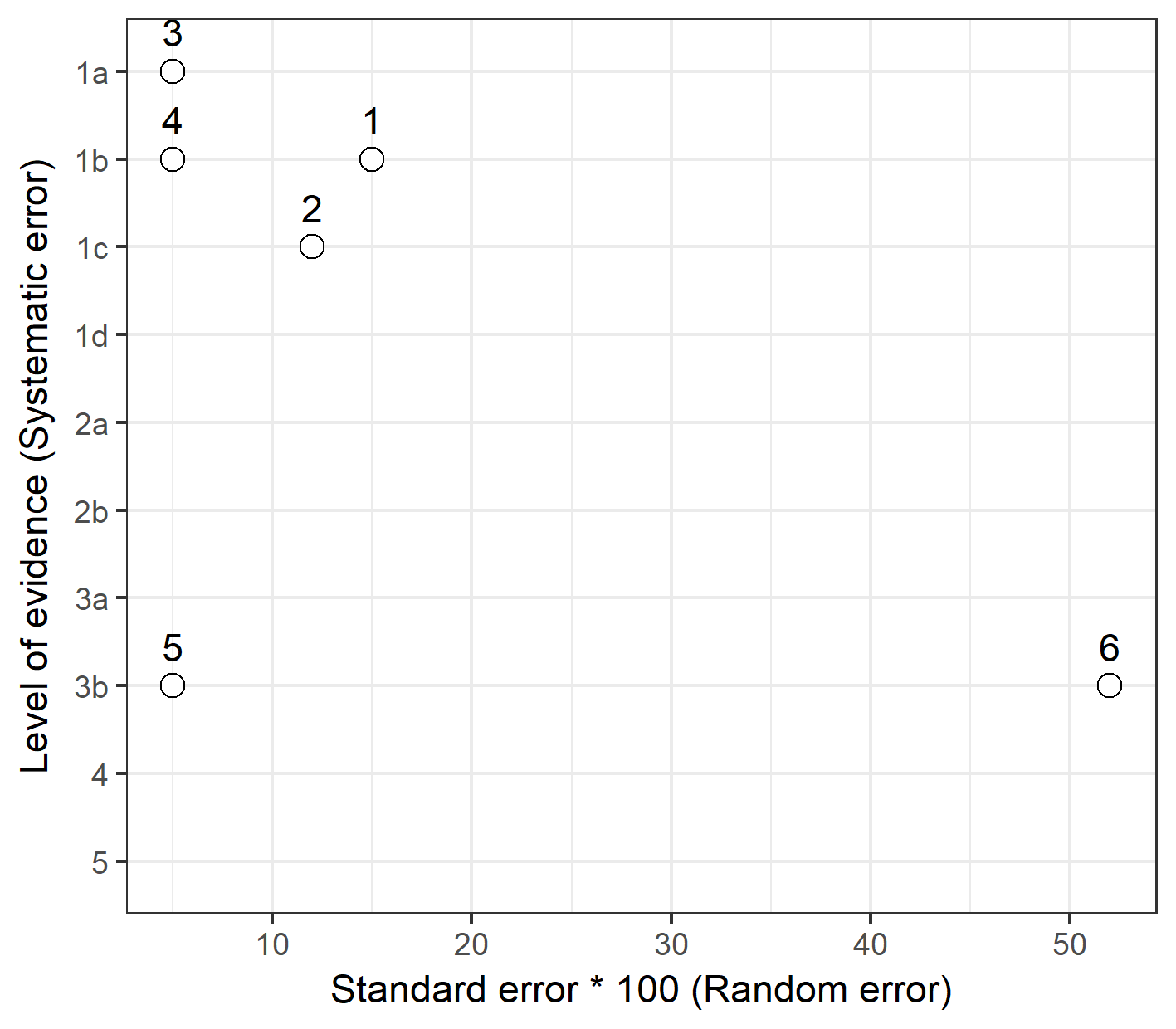 |
| The PRISMA flow chart is a standardized device to visualize the literature search and study selection for systematic reviews and meta-analyses (Moher, Liberati, Tetzlaff, & Altman, 2009). Starting with all potentially relevant publications retrieved, a sequential flow diagram from top to bottom shows transparently for which reasons studies have been excluded and how the final number of studies for the systematic review or meta-analysis was arrived at. | The veritas plot allows to compare different meta-analyses with respect to characteristics and quality criteria of interest to identify the most trustworthy and robust available evidence (Panesar, Rao, Vecht, Mirza, & Darzi, 2009). Different features of interest are shown in a radar plot (e.g., quality assessment, heterogeneity measures, assessed risk of bias). Values in these features for all meta-analyses are shown and compared by differently colored lines. A further possible application of this display is to compare different study subgroups within a meta-analysis. | The error matrix display has been introduced as one possibility to provide an overview of the available evidence for a research question of interest (Keus, Wetterslev, Gluud, & van Laarhoven, 2010). The error matrix display is a scatterplot, with the standard error of each study (random error) on one axis and the level of evidence of each study according to a hierarchy of evidence on the other axis (systematic error). Each study is represented by a point and identifiable by a numerical study identifier. Studies in the upper left corner (low random error, low systematic error) represent strong evidence. For further details, see Keus, Wetterslev, Gluud, and van Laarhoven (2010). |
| 3D error matrix plot [7.8.1] | Effect-direction plot [7.9] | Evidence-map bubble plot [7.10] |
| 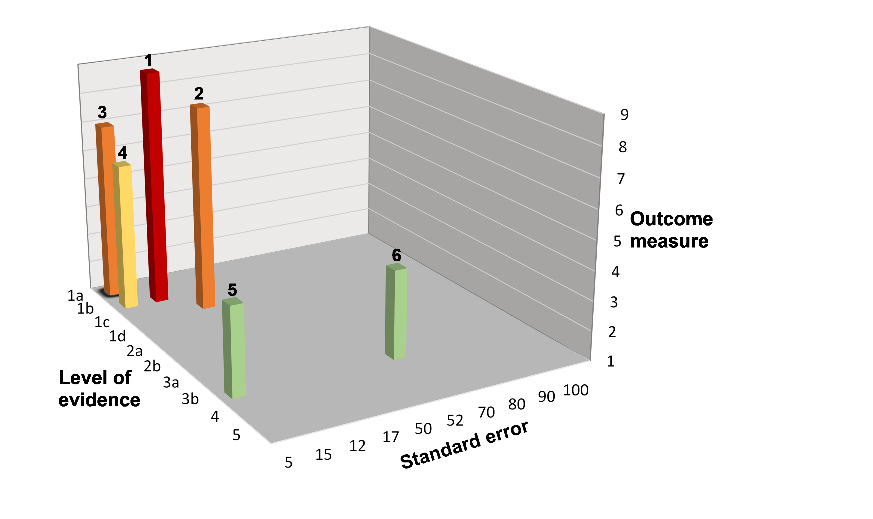 |  |  |
| The three-dimensional error matrix plot extends the error matrix plot [7.8] to a third dimension (Keus, Wetterslev, Gluud, & van Laarhoven, 2010). This third dimension shows the assessed importance of the outcome measure examined in each study (design error), in addition to systematic and random error. Studies contributing the strongest evidence are represented by high bars in the upper left corner of the display (in the above example, these are the studies with identifier numbers 1, 2, 3, and 4). | The effect-direction plot has been introduced to display multiple, not necessarily standardized, outcome measures and study features for numerous studies in a systematic review (Thomson & Thomas, 2013). The display is a table plot, designed to summarize the observed effect direction and statistical significance for each study (rows) and for different outcomes (columns) with arrows (up = positive, down = negative, sideways = no or inconsistent effect; dark shading = statistically significant, light shading = statistically not significant). In addition, study characteristics can be shown for each study in separate columns (e.g., study design or study quality score). | An evidence-map bubble plot has been used to descriptively visualize the amount of evidence available for different combinations of study features (Wang, Shams-White, Bright, Parrott, & 2016). A matrix display is constructed, for which each cell represents a combination of two types of study features (in the above example plot, type of sample in the rows and outcome used in the columns). For each cell, all available studies for this combination of study features are shown with bubbles sized proportional to their sample size. Therefore, the number and size of bubbles within cells informs about the evidence available, whereas the position and scatter of bubbles within cells is uninformative. |
| Dendrogram of meta-cluster analysis [7.11] |  |  |
| 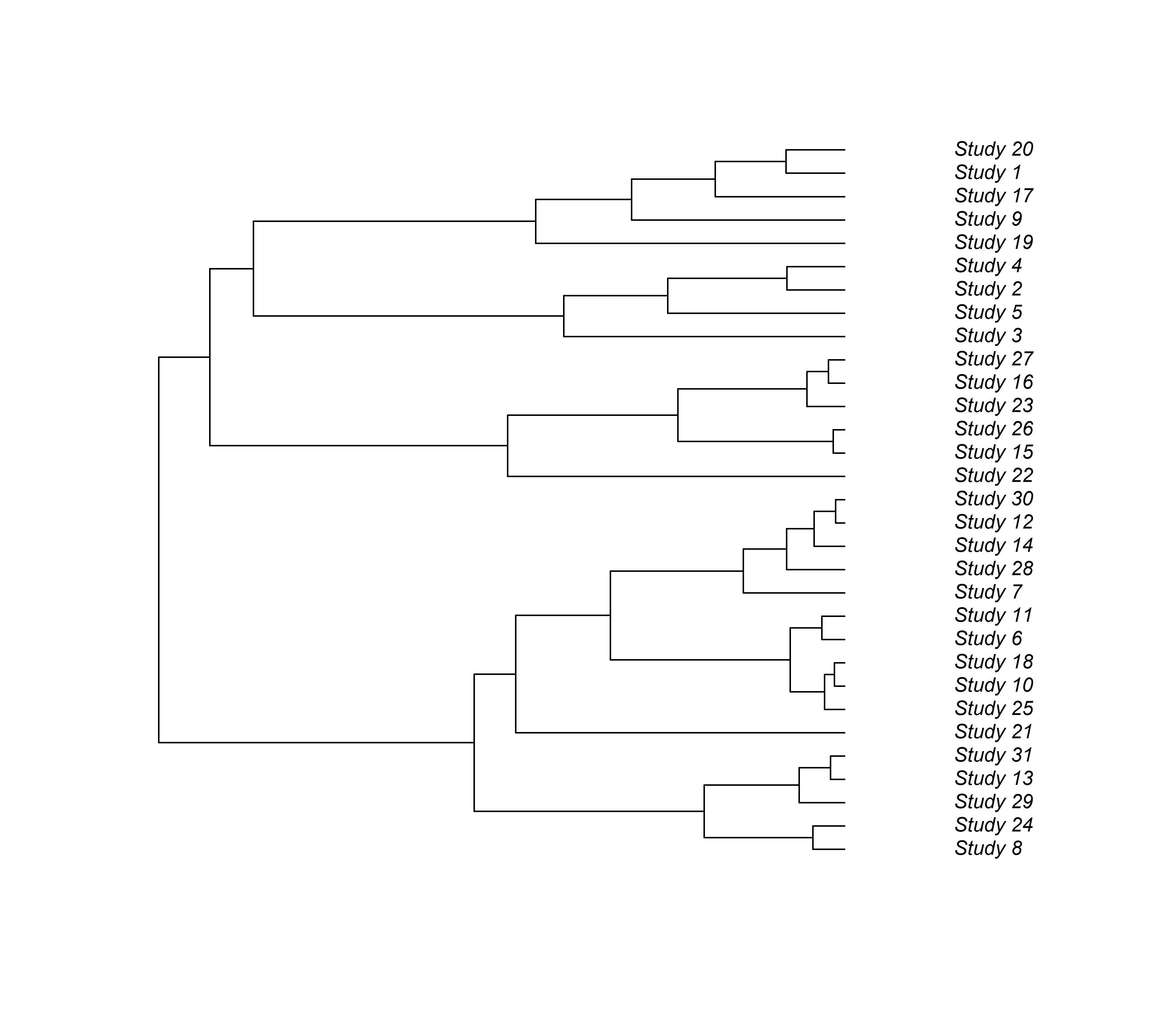 |  |  |
| Meta-cluster analysis has been proposed to explain heterogeneity and to find clusters of similar studies, according to study features and study outcomes. Hierarchical meta-cluster analysis has been visualized, using a dendrogram which displays the observed hierarchy of study clusters (for further details, see Hanji, 2017, p.251-252). |  |  |

| Dichotomous outcomes: L’Abbé plot [8.1] | | Dichotomous outcomes: Subgroup L’Abbé plot [8.1.1] |
| --- | --- | --- |
| 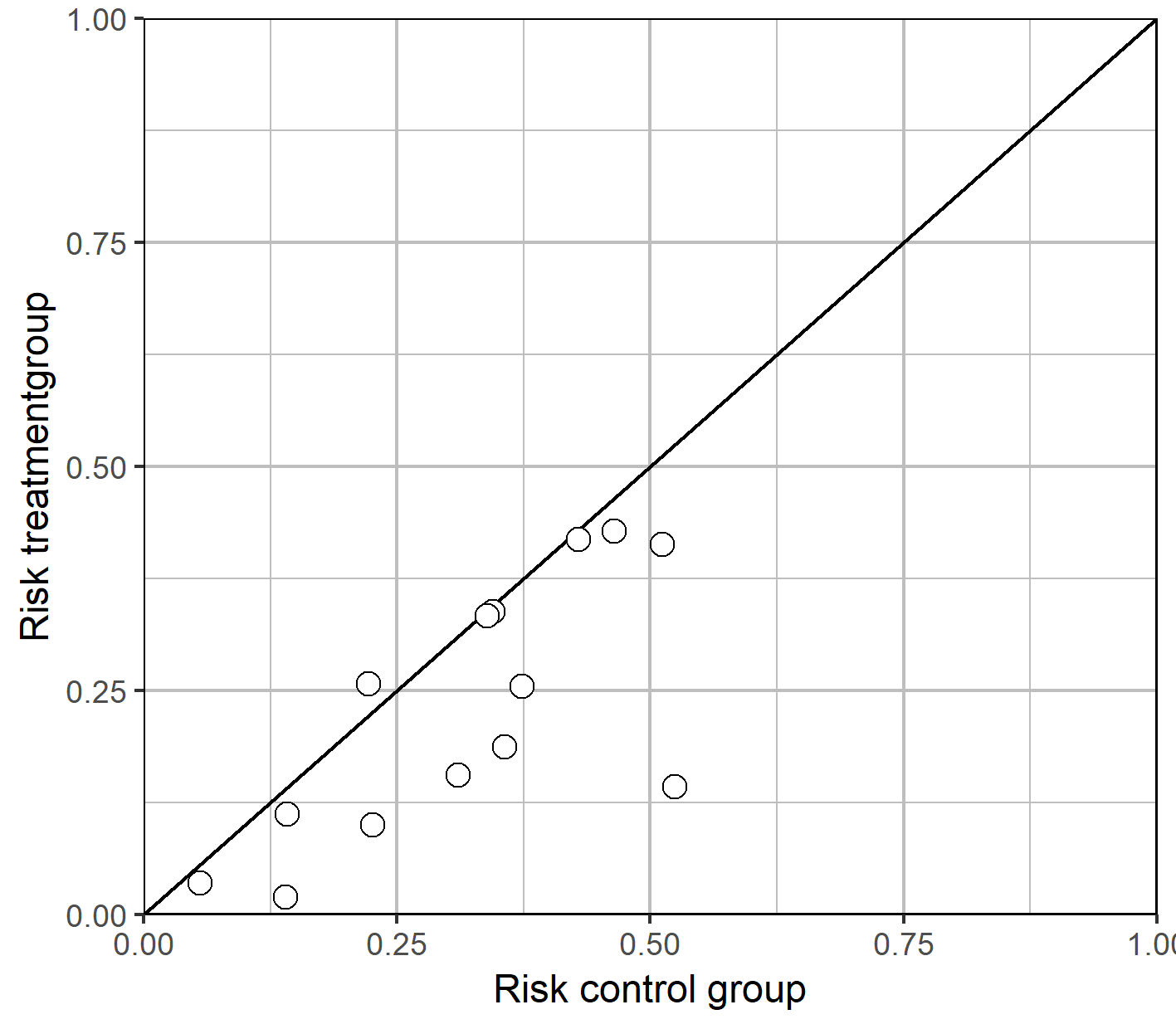 | |  |
| The L’Abbé plot has been introduced to display results of studies with dichotomous outcomes and to assess effect heterogeneity (L’Abbé, Detsky, & O'Rourke, 1987). The estimated risk of an event in one group (e.g., the treatment group) and in a second group (e.g., the control group) within each study is shown in a scatterplot. If the risk for an event is equal in both groups, studies should randomly scatter around the 45-degree reference line going through the origin. Homogeneity of effects in the L’Abbé plot can be checked for risk differences, risk ratios, and odds ratios. No heterogeneity of risk differences within studies should lead to study results randomly scattering around a 45-degree line, with the intercept corresponding to the underlying risk difference. No heterogeneity in risk ratios should lead to studies roughly lying on a line through the origin, with the slope corresponding to the underlying risk ratio. No heterogeneity in odds ratios should lead to studies roughly lying on a smooth curve (see also [8.1.2]), going through the points (0,0) and (1,1), with the curvature corresponding to the underlying odds ratio. Deviations of these systematic patterns convey information about the amount of effect heterogeneity between studies. Single studies not following the general pattern indicate potential outliers. Related scatterplots, sometimes referred to as Brinley plots (Peiffer, Maldjian, & Laurienti, 2008), have also been used to compare means of continuous outcomes in two groups for each study (see also [8.3]). | | A variant of the L’Abbé plot [8.1], showing subgroup effects, has been introduced and used as well (L’Abbé, Detsky, & O'Rourke, 1987). For this purpose, studies are color-coded with respect to study subgroups. Effect heterogeneity can be explained by the subgroup of interest, if (a) studies within different subgroups show a different pattern in the L’Abbé plot, and (b) studies within the same subgroup roughly lie on the same line. |
| Dichotomous outcomes: L’Abbé plot with summary effect contours [8.1.2] | Dichotomous outcomes: L’Abbé weight bubble plot [8.1.3] | Dichotomous outcomes: Baseline graph [8.1.4] |
|  |  |  |
| Contours have been proposed for the L’Abbé plot [8.1], to either show the meta-analytic summary effect or user-specified effect contours (Jimenez, Guallar, & Martín-Moreno, 1997). User-specified contours of the underlying effect can help to assess heterogeneity of effects between studies. For a homogenous set of studies, points should roughly scatter around the same effect line. These underlying effects can either be shown as risk differences (lines with slope of 1, and the intercept corresponding in size to the risk difference), risk ratios (lines with intercept of 0, with the slopes corresponding in size to the risk ratio), or odds ratio (curves through (0,0) and (1,1), with the curvature corresponding to the size of the odds ratio, as in the above example). | The precision of estimates within each study (i.e., the number of participants and events observed) has been shown within a L’Abbé using weight bubbles (see also [3.2.1]). Each study is represented by a point proportional to this study’s meta-analytic weight or sample size. | The baseline graph can be seen as a variant of the L’Abbé plot [8.1], showing the risk difference for the event in the two groups and corresponding confidence intervals on the y axis. By using the risk difference on the y axis, the reference line for no effect now is horizontal, instead of diagonal. If there is no heterogeneity between studies, they should lie on a straight horizontal line (homogeneity in risk differences) or along a sloped line going through the origin (homogeneity in risk ratios). An association of the risk in the reference group on the x axis with the observed risk difference can be seen by a linear trend in the plot. For a published example, see Stangl and Berry (2000, p. 136). |
| Time-to-event data: Study survival curves [8.2] | Bivariate meta-analysis plot [8.3] | Bivariate meta-analysis plot with confidence ellipse [8.3.1] |
|  |  |  |
| Study survival curves have been used to display study-level results in meta-analyses of survival data (Voest, van Houwelingen, & Neijt, 1989). The survival function shows the estimated probability that the actual survival time is at least as long as a given time point (see also [9.2]). Study-specific survival curves – either estimated non-parametrically (e.g., Kaplan-Meier curves) or parametrically – are simultaneously shown and differentiated by different colors or line types. | Multivariate meta-analysis simultaneously summarizes more than one outcome per study, taking into account correlations between outcomes (van Houwelingen, Zwinderman, & Stijnen, 1993). For two outcomes per study (or more generally cluster level), the bivariate-meta-analysis plot shows both outcomes for each study in a scatterplot and the summary effect obtained in a multivariate meta-analysis as a diamond. Summary diamonds from univariate meta-analysis, only considering one outcome per study, are shown at the bottom and left-hand side of the plot. The above plot was created using the R package metaSEM (Cheung, 2015). | Confidence ellipsoids can be shown in a bivariate meta-analysis plot (van Houwelingen, Zwinderman, & Stijnen, 1993). The 95% confidence ellipsoids for the meta-analytic summary effect (red in the example above) has a 95% ad-hoc probability to contain the two-dimensional true effect. Also shown are confidence intervals and ellipsoids for study-specific random effects (i.e., the expected effect for a new study). The above plot was created using the R package metaSEM (Cheung, 2015). |
| Cross-hairs scatterplot [8.3.2] | Cross-hairs scatterplot with subgroups [8.3.3] | Dichotomous outcomes: ROC plot [8.4] |
|  |  |  |
| The cross-hairs scatterplot shows two outcomes per study, including study-specific confidence intervals (Brannick & Gültaş, 2016). For each study, the observed effects in both outcomes are shown, and associations between both outcomes become visible. In addition, confidence intervals for each effect are shown (resembling a cross-hair). Horizontal and vertical reference lines of no effect are additionally shown. At the bottom and left-hand side of the plot, boxplots [6.2] visualize the univariate distribution of effects in all studies. The above plot was created using the R package metaplotr (Gültaş & Brannick, 2016). | Subgroup information has been incorporated into the cross-hairs scatterplot [8.3.2] by different color coding (Brannick & Gültaş, 2016). The plot thus allows to visualize different associations between outcomes for studies in different subgroups. The above plot was created using the R package metaplotr (Gültaş & Brannick, 2016). | ROC (receiver operating characteristic) plots have been proposed and used in the context of meta-analysis to display results of diagnostic test accuracy studies (Moses, Shapiro, & Littenberg, 1993). As a special case of the bivariate meta-analysis plot [8.3], for each study the estimated sensitivity (probability that the test is positive, conditional on the event of interest having occurred) and one minus specificity (probability that the test is negative, conditional on the event of interest not having occurred) are shown in a scatterplot. The diagonal 45-degree reference line shows what can be expected, if the test completely randomly gets positive with a specified prevalence. This therefore is the lower bound for the performance of any test. If a meta-analytic summary effect was computed, a summary diamond (potentially with a confidence ellipse) can be shown as well (see also [8.3.1]). For a general overview of meta-analysis with test accuracy data, see Sutton, Abrams, Jones, Sheldon, and Song (2000, p.209-215). |
| Dichotomous outcomes: ROC plot with summary ROC curve [8.4.1] | Dichotomous outcomes: Cross-hairs plot [8.4.2] | Dichotomous outcomes: ROC plot with subgroups [8.4.3] |
|  |  |  |
| Meta-analytic methods to estimate a summary receiver operating characteristic (ROC) curve from results of primary studies allow summarizing the relationship and tradeoff between sensitivity and specificity (Moses, Shapiro, & Littenberg, 1993). This summary ROC curve has been shown – either additionally or exclusively – in meta-analytic ROC plots [8.4]. For a general overview of meta-analysis with test accuracy data, see Sutton, Abrams, Jones, Sheldon, and Song, (2000, pp. 209-215). | The cross-hairs plot allows displaying study results in a meta-analysis of diagnostic test accuracy studies (Phillips, Stewart, & Sutton, 2010). As a special case of the later proposed cross-hairs scatterplot [8.3.2], sensitivity and one minus specificity estimates of each study are shown in a ROC (receiver operating characteristic) plot [8.4]. In addition, confidence intervals for each study are displayed for both estimates, resulting in symbols resembling cross-hairs. Contrary to forest plots [1.2] for sensitivity and specificity separately, the cross-hairs plot conveys information on the natural association of sensitivity and specificity. | Information on study subgroups has been displayed in ROC (receiver operating characteristic) plots [8.4] as well. Summary estimates and summary ROC curves are shown for studies in a certain study subgroup separately, using different colors, line types, or symbols. For an example and further details, see Whiting et al. (2008). |
| Dichotomous outcomes: Treatment benefit vs. control plot per 100 patients [8.5] | Dichotomous outcomes: Olliaro display [8.6] | Dichotomous outcomes: Subgroup Olliaro display [8.6.1] |
|  |  |  |
| The treatment benefit vs. control plot per 100 patients plot has been developed in the context of health-care research as an articulate display to present the results of studies with dichotomous outcomes (Deeks & Altman, 2001, pp. 313-335). Similar to the baseline graph [8.1.4], the risk for an event (e.g., death) in the control group is shown on the x axis, and the difference of risks in the treatment and control group is shown on the y axis. Risks are multiplied by 100 and therefore show the expected number of events per 100 patients in the control group (x axis), and the expected number of additional events in the treatment group (y axis). Confidence intervals for the estimated number of additional events in the treatment group are displayed for each study. The meta-analytic summary effect is shown, using three different outcome metrics (see also L’Abbé plot [8.1]): risk difference [*RD*; horizontal line], risk ratio [*RR*; line with corresponding slope] and odds ratio [*OR*; curved line with corresponding curvature]). Estimated summary risk differences (corresponding to summary *RD*, *RR*, or *OR* lines) for risks in the control group, not observed in any primary study, are dashed, in order to indicate extrapolation. | The Olliaro display has been proposed to inform about (a) the relative effect of a test treatment compared to a reference treatment, (b) the absolute effect of the test treatment, and (c) the between-study heterogeneity of reported effects (Olliaro & Vaillant, 2010). The risk difference of the test treatment compared to a reference treatment is plotted on the x axis (relative effect), and the failure rate of the test treatment on the y axis (absolute effect). A vertical null effect reference line divides the display in segments where the test treatment (left) or reference treatment (right) showed better results. A horizontal reference line shows the maximum tolerated failure rate for a treatment, in order to be regarded as effective. Confidence intervals for the failure rates are shown as well, and each study is plotted proportional in size to the study’s sample size. The area of the polygon connecting all studies is used as an indicator of heterogeneity. | The Olliaro display [8.6] has been used to compare the absolute and relative effect of different study subgroups (e.g., subgroups comparing different test treatments with the same control treatment; Olliaro & Vaillant, 2010). The area of subgroup-specific polygons conveys information about the between-study heterogeneity within subgroups and heterogeneity differences between subgroups. |
| Dichotomous outcomes: Threshold plot [8.7] | Meta-analytic Bland-Altman plot [8.8] |  |
|  |  |  |
| The threshold plot accompanies newly proposed methods for the meta-analysis of diagnostic accuracy studies which report sensitivity and specificity estimates for more than one diagnostic threshold value (Steinhauser, Schumacher, & Rücker, 2016). The threshold plot visualizes the estimated meta-analytic summary and study-level sensitivity and specificity for different diagnostic thresholds. The probability that the test is negative is plotted as a function of the diagnostic threshold, separately for actually negative cases (corresponding to specificity) and positive cases (corresponding to one minus sensitivity). The estimated optimal threshold is indicated by a vertical line (for further details, see Steinhauser, Schumacher, & Rücker, 2016). The above plot was created by using and adapting code and example data provided within supplemental material of Steinhauser, Schumacher, and Rücker (2016). | The Bland-Altman plot (Bland & Altman, 1986) has been used in the context of meta-analysis to compare two correlated outcome measures per study. For each study, the difference of the two measures is plotted on the y axis, and the mean of the two measures is plotted on the x axis. Horizontal reference lines indicate the mean difference between the two measures and 95% confidence limits for this difference. The meta-analytic Bland-Altman plot allows examining (a) whether one measure within each study is generally higher than the other, (b) how large the variation in the differences between measures is, and (c) whether the size of the difference between measures is dependent on the magnitude of measures. A source reference in the context of meta-analysis, covering the Bland-Altman plot, is Koricheva, Gurevitch, and Mengersen (2013, pp. 346-347). |  |

| Glass distributional overlap display [9.1] | Time-to-event data: Summary survival curve [9.2] | Time-to-event data: Subgroup survival curves [9.2.1] |
| --- | --- | --- |
|  |  |  |
| As early as 1976, Glass used two overlapping normal distributions to present and illustrate the meta-analytic summary effect as standardized mean difference between two groups (commonly referred to as the Cohen *d* effect size). The magnitude of the summary Cohen *d* value is visualized by the amount of standard deviations the means of the two distributions differ from each other (Glass, 1976). | Methods for meta-analyses of survival data have been proposed in the late 1980s (Voest, van Houwelingen, & Neijt, 1989). To present the results of a meta-analysis of several survival analyses, standard survival curve plots have been used, showing the summary survival curve. The summary survival curve synthesizes the survival curves from all primary studies and shows the estimated probability to survive at least up to this time point, incorporating all available evidence. | Summary survival curves [9.2] have also been used to visualize meta-analytic summary results of survival analyses for different subgroups of studies (e.g., studies differing in drug dosage used). For this purpose, separate survival curves are computed and shown, using different colors or line types (Voest, van Houwelingen, & Neijt, 1989). |

| Summary path diagram (SEM) [9.3] | Genetic data: Summary *q* value plot [9.4] | Genetic data: Summary Q-Q plot [9.5] |
| --- | --- | --- |
|  |  |  |
| For the presentation of meta-analysis using the structural equation modeling (SEM) framework, path diagrams have been used (for an early example, see Shadish & Sweeney, 1991). SEM in the context of meta-analysis has two main areas of application (for details, see Cheung & Chan, 2005). First, standard meta-analysis, potentially with several moderators, is computed within the SEM framework. Second, results from SEMs reported in primary studies are meta-analyzed. In both cases, path diagrams are suitable to present the underlying model and the estimated summary effects. | For the meta-analysis of micro-array data, summary *q* value plots have been used (Rhodes, Barrette, Rubin, Ghosh, & Chinnaiyan, 2002). The summary *q* value statistic based on all primary studies is shown in increasing order for all genes of interest. The *q* values on the y axis are adjusted *p* values, used when numerous significance tests are performed, in order to control the false discovery rate (i.e., the expected proportion of false positives among all significant test results). Either overall summary *q* values are shown (as in the above example), or separate curves for study subgroups, or study-specific *q* value curves are visualized (for examples, see Rhodes, Barrette, Rubin, Ghosh, & Chinnaiyan, 2002). | In meta-analysis of genetic data, the results of many statistical tests are summarized (e.g., differential expressions of many genes of interest). Summary Q-Q plots are the summary version of Q-Q plots, often used in primary genetic studies (see also [6.7]). The observed quantiles of meta-analytic summary statistics for each gene are plotted against expected quantiles of these statistics. Examples for summary statistics used are the observed heterogeneity *Q* statistics obtained for each gene, compared against the χ^2^ distribution expected under homogeneity, or the distribution of observed summary *p* values for each gene, compared with a uniform distribution, expected if all null hypotheses were true (Choi, Yu, Kim, & Yoo, 2003). The above plot was created using the R package qqman (Turner, 2017). |
| Hattie barometer display [9.6] | Comparison of meta-analyses: FEM vs. REM summary estimates [9.7] | Comparison of meta-analyses: Heterogeneity [9.8] |
|  |  |  |
| An easy to understand infographic in the form of a barometer display has been developed and used to present meta-analytic summary effects (Hattie, 2008). The barometer display is divided into broad effect-size magnitude regions. These regions of effect-size magnitudes are labeled specific to the research area in questions. In the case of Hattie (2008), effects have been labeled according to practically relevant effects in educational research. An arrow points to the observed effect size within the effect-size barometer. For an example, see Hattie (2008, p. 19). | A scatterplot has been used to compare the meta-analytic summary effect resulting from the fixed-effect and from the random-effects meta-analytic model (Bowden, Tierney, Copas, & Burdett, 2011). For a number of meta-analyses, the summary estimates from the two models and corresponding confidence intervals are shown by cross-hairs (see also [8.3.2]). Deviations from the 45-degree line indicate sensitivity to the model choice. The observation of between-study heterogeneity (*I*^2^) for each meta-analysis is represented by filled points, and color coding is used to indicate meta-analyses where the two models differ in the statistical significance of the meta-analytic summary effect. | For several meta-analyses, the amount of estimated between-study heterogeneity has been visualized by a confidence interval plot (Bowden, Tierney, Copas, & Burdett, 2011). For each meta-analysis, the estimated *I*^2^ heterogeneity statistic and 95% confidence interval is shown. In addition, the τ^2^ estimate is annotated at the bottom. Color coding is used to differentiate meta-analyses with no, insubstantial, or substantial estimated heterogeneity (using conventional heterogeneity limits). |

| Comparison of meta-analyses: Tau square estimates [9.9] | Genetic data: Meta-analytic circos plot [9.10] | | | Fishbone diagram [9.11] | | |
| --- | --- | --- | --- | --- | --- | --- |
|  |  | | |  | | |
| For several meta-analyses, the difference of methods for the estimation of the between-study variance parameter τ^2^ has been visualized in a scatterplot (Bowden, Tierney, Copas, & Burdett, 2011). For each meta-analysis, τ^2^ estimated by two different methods is shown. Deviations from the 45-degree line indicate sensitivity to method choice. As in [9.8], color coding is used to differentiate between meta-analysis with no, insubstantial, or substantial observed heterogeneity. | Circos plots are a visualization framework for genetic studies (Krzywinski et al., 2009). They are especially suited for comparing different genomes, displaying relationships between genome positions, as well as relationships with different phenotypes (e.g., cancer types). For an example in the context of a meta-analysis of micro-array data, see Feichtinger, McFarlane, and Larcombe (2012). The above example plot was created using the R package circlize (Gu, 2014). | | | Fishbone diagrams have been introduced to present combined effects and evidence summaries for multiple outcomes in a clear and structured way (Gartlehner et al., 2017). For a health-related research question, stated in the head of the fishbone, available summaries from systematic reviews and meta-analyses for different outcome measures are depicted at separate fishbones. Proposed summary information for each outcome measure can be the meta-analytic summary effect, the number of studies and total sample size examining this outcome measure, and the strength of available evidence, using evidence rating systems (e.g., GRADE). For further details, see Gartlehner et al. (2017). | | |
| Evidence flowers [9.12] | | Likelihood, prior, or posterior distribution plot [9.13] | | | Bootstrap chart [9.13.1] | |
|  | |  | | |  | |
| Evidence flowers are a recently proposed visualization tool to communicate multiple related summary results of meta-analyses and systematic reviews to audiences without statistical-technical background in the context of health research (Babatunde et al., 2018). For a health-related condition of interest, available empirical evidence for different treatments are summarized and presented in the petals of the evidence flower. Within each petal, treatment-specific summary outcomes can be provided. In addition, each petal is colored according to an evidence-rating system (e.g., GRADE), indicating the strength of empirical evidence. For further details, see Babatunde et al. (2018). | | Results of Bayesian meta-analysis have been regularly visualized by plots overlaying the likelihood, the prior, and the estimated posterior distribution (Stangl & Berry, 2000). These triplots allow to visually examine the effect of prior knowledge on the observed likelihood to arrive at the estimated posterior distribution of the true parameter. Separate posterior distributions can be shown for different subgroups (e.g., treatment comparisons). Plots of likelihoods or profile likelihoods of key parameters have been used (not necessarily exclusively) for Bayesian meta-analysis as well (Eddy, Hasselblad, & Schachter, 1992). The above example plot was created with the R package metaBMA (Heck, Gronau, & Wagenmakers, 2017). | | | The sampling distribution of meta-analytic parameter estimates can be approximated with the (empirical) bootstrap technique (Rudner, Glass, Evartt, & Emery, 2002). From all studies in the meta-analysis, a sample of studies of the same size as the original meta-analysis is drawn with replacement, and the parameter of interest (usually the summary effect or the between-study heterogeneity τ^2^) is estimated. This is repeated many (say, 1000) times, and all obtained parameter estimates are then plotted in a density chart [6.5] or histogram [6.1]. In addition, a corresponding bootstrap confidence interval for the parameter can be highlighted within the plot (as in the above example). | |
| Likelihood, confidence, or posterior region plot for two parameters [9.13.2] | Predictive distribution or interval plot [9.13.3] | | Dichotomous outcomes: Fagan nomogram [9.14] | | |  |
|  |  | |  | | |  |
| Region plots show probable or likely values for two meta-analytic parameters simultaneously. In the context of meta-analysis, these parameters are predominately the summary effect and the between-study heterogeneity parameter τ^2^. Region plots show the joint posterior distribution in the context of Bayesian meta-analysis, either using contours (as in the example above) or three-dimensional surfaces (Stangl & Berry, 2000, p.372). Moreover, likelihood-based confidence regions (Rukhin, 2015) have been used. Region plots allow examining which pairs of parameter values are plausible at the same time. The above example plot was created using the R package bayesmeta (Roever, 2017). | Prediction intervals and (posterior) predictive distribution have been used in the context of random-effects meta-analysis and Bayesian meta-analysis. In essence, prediction intervals and (posterior) predictive distributions show where the effect size of a new hypothetical observation is expected to fall. Oftentimes, the prediction interval is shown in addition to the summary effect’s confidence interval in a forest plot [1.2]. In random-effects meta-analysis, prediction intervals (for study-specific true effects) show in which effect-size region the expected effect size of a new study is likely to fall. Prediction intervals are wider than the confidence interval of the summary effect; they often are shown as an additional diamond (see bottom of the above example display). An example in the context of meta-analysis can be found in Stangl and Berry (2000, pp. 73, 79). | | The Fagan nomogram (also known as likelihood-ratio nomogram) can be used to show the summary result of a meta-analysis of diagnostic test accuracy studies. For one or more diagnostic tests, the Fagan nomogram shows the estimated probability for a condition prior to the test (baseline) and after the diagnostic test, with either a negative or positive test result. For an example application in the context of meta-analysis, see Shen et al. (2014), and for further details, Whiting et al. (2008). | | |  |
| Dichotomous outcomes: Probability-modifying plot [9.14.1] | Genetic data: Meta-analytic Manhattan plot [9.15] | | Genetic data: Meta-analytic Miami plot [9.15.1] | | |  |
|  |  | |  | | |  |
| The probability-modifying plot displays the estimated post-test probability to have a condition as a function of the pre-test probability for the condition. Probabilities are shown separately for a positive and negative diagnostic test. Like the Fagan nomogram, the probability-modifying plot can be used to report summary results of meta-analysis of test accuracy studies. Summary results for more than one test of interest can be compared. For further details, see Whiting et al. (2008). | Manhattan plots are widely used to present results of genome-wide association studies. The Manhattan plot shows the negative logarithm (base 10) of the *p* value of the association of a specific single-nucleotide polymorphism with a phenotype of interest. Meta-analytic Manhattan plots show *p* values obtained by summarizing results of several primary study; that is, each point in the meta-analytic Manhattan plot is a summary *p* value. Horizontal reference lines can be used to show *p* value thresholds (in the above plot, 10^-5^ and 10^-7^). For a published example application of this display in the context of meta-analysis, see Fogh et al. (2013). The above example plot was created using the R package qqman (Turner, 2017). | | The meta-analytic Miami plot has been used to display and compare two meta-analytic Manhattan plots [9.15]. For each single-nucleotide polymorphism, summary *p* values are shown for two study subgroups or phenotypes of interest separately. For a published example and further details, see Winkler, Kutalik, Gorski, Lottaz, Kronenberg, and Heid (2014). The above example plot was created using the R package qqman (Turner, 2017). | | |  |
| Genetic data: Meta-analytic regional association plot [9.15.2] | Genetic data: Meta-analytic volcano plot [9.16] | | Genetic data: Position-summary line plot [9.17] | | |  |
|  |  | |  | | |  |
| Regional association plots are a variant of Manhattan plots [9.15], but for a more limited region of the chromosome (i.e., zoomed-in Manhattan plots). For each single-nucleotide polymorphism, the association summary *p* value is shown. In addition, estimated recombination rates are shown on a second axis. For an example application in the context of meta-analysis, see Ng et al. (2014). The above example plot was created using the R package gap and example data therein (Zhao, 2007). | In meta-analyses of genetic studies with many statistical tests, often not only the *p* value of each test, but also an effect-size measure is of interest. The meta-analytic volcano plot shows for each statistical test the summary *p* value on the y axis (usually transformed to minus the logarithm of base 10) and an effect-size measure (usually transformed with the logarithm of base 2) on the x axis. In the context of meta-analysis of genome-wide association studies, for each single-nucleotide polymorphism (SNP), the summary *p* value of the association with the phenotype of interest is shown against the odds ratio of that phenotype and SNP. Therefore, SNPs with strong associations, that are at the same time statistically significant, appear at the top right-hand or left-hand side of the plot. For an example application, see van Uitert et al. (2015). | | The position-summary line plot allows presenting the results of a meta-analysis of quantitative trait-locus mapping studies. Quantitative trait loci (QTL) are areas of the genome associated with a quantitative trait in a phenotype of a population. For a region on the genome, an estimated summary measure of association (e.g., summary LOD [logarithmic odds ratio] score) with the quantitative trait of interest is shown with line plots. For further published examples, see Guerra and Goldstein (2010, pp. 59 -63). | | |  |
| Genetic data: Summary heatmap [9.18] | Meta-analytic neuroimaging plot [9.19] | |  |  |  |  |
|  |  | |  |  |  |  |
| Meta-analyses of gene expression data have been displayed in (summary) heatmaps (Rhodes, Barrette, Rubin, Ghosh, & Chinnaiyan, 2002). For different genes of interest, either summary expression levels for each study, or study subgroup or expression levels for different samples, or sample subgroups (nested within studies/datasets) are indicated by color and saturation. The above example plot was created with the R package MetaIntegrator (Haynes et al., 2018). | Meta-analysis of functional neuroimaging data is used to summarize brain-activity level data from primary studies. The activity level in different areas of the brain while performing mental tasks is summarized using data from all available primary studies. These summarized activity levels are shown with two-dimensional or three-dimensional displays of the brain, in an anatomically defined heatmap-like display. Color-coding is used to indicate areas with high summary activation levels. The above example was created using the software ENIGMA-viewer and example data therein (Zhang et al., 2017). | |  |  |  |  |

| Schweder-Spjøtvoll plot [10.1] | Publication-probability plot (selection model) [10.2] | Sensitivity contour plot (selection model) [10.3] | |  |
| --- | --- | --- | --- | --- |
|  |  |  | |  |
| The Schweder-Spjøtvoll plot displays the cumulative distribution of observed *p* values and was proposed as a general display to evaluate a whole set of *p* values corresponding to a large number of significance tests (Schweder & Spjøtvoll, 1982). The *p* values from tests, for which the null hypothesis is true, are uniformly distributed and should lie on a straight line, while this is not true for *p* values stemming from (appropriately powered) hypothesis tests for which the null hypothesis is not true. The Schweder-Spjøtvoll plot was proposed in the context of meta-analysis for meta-analytic methods to summarize *p* values (Dewey, 2018). | In selection models, the probability of effect sizes to be observed and published can be formulated as a weighted probability distribution function. A common corresponding weight function depends only on the *p* value of the effects (Dear & Begg, 1992; Vevea & Woods 2005). This estimated weight function has been visualized, using step functions (for further details, see Hedges & Vevea, 2005). | Different selection models exist, which model the underlying mechanism of publication bias. One widely known section model is the Copas selection model (Copas & Shi, 2000). In the Copas selection model, the mechanism of publication bias is formulated using two parameters, γ_0_ and γ_1_, determining the severity of publication bias depending on the precision of studies (for further details, see, e.g., Schwarzer, Carpenter, & Rücker, 2015, pp. 128-134). The sensitivity contour plot visualizes combinations of these two parameter values, resulting in a specific adjusted summary effect by contour lines (dashed lines). The solid line of steepest descent shows the sequence of parameter value combinations for which the adjusted summary effect is most sensitive. The above plot was created using the R package metasens (Schwarzer, Carpenter, & Rücker, 2017). | |  |
| Test of excess significance alpha sensitivity plot [10.4] | Caliper test display [10.5] | Plot of truncated normal distribution [10.6] | |  |
|  |  |  | |  |
| The test of excess significance (TES) is a widely used evidentiality test (Ioannidis & Trikalinos, 2007). For a set of studies, the TES examines whether there are more statistically significant studies than expected, given their individual power to detect an effect of interest with a specific magnitude (e.g., the meta-analytic summary effect value). The TES alpha sensitivity plot shows the *p* value of the TES as a function of the statistical significance criteria α for which study effects were initially considered significant. If there is an excess of significant studies in the set due to bias (significance chasing or *p*-hacking), the TES should be in particular significant for conventionally used significance criteria (i.e., *p* < .05). | The caliper test is a test for bias in a set of studies (Gerber & Malhotra, 2008). The principal idea is, when there is no bias, roughly the same number of results slightly below a conventional significance threshold (e.g., .05) and above this threshold are expected. If there is an abundance of just‑significant results, this indicates bias. The caliper test display shows the distribution of test statistics and highlights the number of test statistics in a small interval above and below the significance threshold (dashed line in the above example plot) as basis for the caliper test. | The truncated normal distribution plot has been used to visualize the results of a method introduced to estimate the proportion of studies missing due to publication bias (Formann, 2008). This method is based on the idea that observed effect sizes are normally distributed. However, due to selection bias, the effect sizes actually published follow a truncated normal distribution. The estimated truncated normal distribution due to publication bias is shown alongside the expected untruncated distribution without selection bias. | |  |
| Genetic data: P-M plot [10.7] | *p-*curve [10.8] | *p*-curve sensitivity plot [10.8.1] | |  |
|  |  |  | |  |
| The P-M plot has been proposed for genetic data, showing the *p* value of each study and the *m* value, which is the posterior probability that the effect exists in each study (Han & Eskin, 2012). The *m* value and *p* value for each study for a locus of interest are shown in a scatterplot. For a set of heterogeneous studies, the P-M plot allows separating those studies for which an effect exists from those studies in which no real effect exists. Studies with low *p* values and high *m* values (top right) are predicted to have found a true effect, whereas studies with high *p* values and medium-to-low *m* values are seen as ambiguous, or are predicted not to have found a true effect, respectively. | The *p*-curve visualizes the probability density distribution of significant *p* values in the context of *p*-curve analysis (Simonsohn, Nelson, & Simmons, 2014). Left-skewed *p-*curves with an abundance of just-significant *p* values indicate bias in the set of studies. If the null hypothesis is true, a uniform distribution of *p* values is expected (indicated by a horizontal dashed line). If there is evidential value in the set of findings (i.e., the alternative hypothesis is true), then a right-skewed *p*-curve is expected. The expected (right-skewed) *p*-curve for a set of studies with 33% power is also shown as a green reference curve. Main test results of the *p* curve analyses are often given as well within the plot: Is the observed *p* curve significantly left-skewed or right skewed? Is the observed *p*-curve more right-skewed than what is expected for 33% power? | Visual sensitivity analyses for a *p*-curve analyses have been previously used (Taylor & Munafò, 2016). To check the sensitivity of the *p*-curve analysis, test results are recomputed with the *k* highest or *k* lowest observed *p* values excluded. The *p* value of the *p*-curve tests (right skew, left skew, more right-skewed than 33% power curve) are then shown as a function of the number of excluded observed *p* values. Test results might be less convincing, if they are highly sensitive to the exclusion of only a small number of *p* values. | |  |
| *p*-value plot for selection bias (selection model) [10.9] | Treatment effect plot (selection model) [10.10] | Albatross plot [10.11] | | |
|  |  |  | | |
| In the Copas selection model (Copas & Shi, 2000), the severity of the modeled publication bias can be expressed as the probability that the trial with the largest observed standard error would be published. The *p*-value plot for selection bias shows the *p* value for unexplained selection bias as a function of the publishing probability of the study with the largest standard error. The *p* value at the y axis corresponds to the test whether there still is unexplained selection beyond the selection model used. It is evaluated for distinct summary values lying on the line of steepest descent in the sensitivity contour plot [10.3], as well as for no selection bias. In the above example plot, an insignificant *p* value higher than .10 is obtained for a publishing probability (for the smallest study) of 60%. The corresponding treatment effect of this publishing probability could then be read-off of the treatment effect plot [10.10] (for further details, see Schwarzer, Carpenter, & Rücker, 2015, pp. 128-134). The above plot was created using the R package metasens (Schwarzer, Carpenter, & Rücker, 2017). | The results of the Copas selection model (Copas & Shi, 2000) have been visualized with the treatment effect plot (Schwarzer, Carpenter, & Rücker, 2015, pp. 128-134). In the Copas selection model, the severity of the modeled publication bias can be expressed as the probability that the trial with the largest observed standard error would be published. The treatment effect plot shows the estimated (adjusted) meta-analytic summary effect and 95% confidence intervals by the model as a function of this publication probability of the study with the largest standard error. As in the *p* value plot for selection bias [10.9], estimates are shown for distinct summary values lying on the line of steepest descent in the sensitivity contour plot [10.3], as well as for no selection bias. The above plot was created using the R package metasens (Schwarzer, Carpenter, & Rücker, 2017). | The albatross plot (Harrison, Jones, Martin, Lewis, & Higgins, 2017) allows visualizing meta-analytic data without knowing study effect sizes. It is based on one-sided *p* values and sample sizes or, alternatively, on two-sided *p* values, the direction of effect, and sample sizes. The *p* values are depicted on a (transformed) x axis (with small *p* values on either side, depending on the direction of effect), whereas the sample size is shown on the y axis. For many effect statistics (e.g., standardized mean difference, correlation, odds ratio), contours can be drawn, showing sample size and *p* value combinations corresponding to a specified effect magnitude. For a meta-analysis of homogeneous studies, it is expected that studies scatter around the contour corresponding to the true underlying effect. The contours in the albatross therefore allow an approximative assessment of the summary effect, even if no study effects are available. If studies do not scatter symmetrically around any contour, this is indicative for an excess of between-study heterogeneity. The above plot was created within Stata, using the module ALBATROSS (Harrison, 2017). | | |
| Albatross subgroup plot [10.11.1] | Weighted effect-size density (selection model) [10.12] | | Maximum-bias forest plot [10.13] | |
|  |  | |  | |
| A variant of the albatross plot [10.11] has been proposed to illustrate potential sources of between-study heterogeneity. Study groups are depicted by different symbols. This might aid to assess whether different study groups scatter around different effect contours which indicate different underlying effects for these subgroups. The above plot was created within Stata, using the module ALBATROSS (Harrison, 2017). | Weighted effect-size density plots have been used to visualize the estimated weighted-density distribution of effect sizes when publication bias is incorporated via a (often *p*-value based) weight function (as shown in [10.2]). For further details on selection models using weight functions, see Vevea and Hedges (1995) and Vevea and Woods (2005). In the above example, statistically significant effect sizes in the expected direction have an increased probability to be published, as compared to non-significant effect sizes. Software to visualize the weighted density (after selection) and to conduct related analyses using the Vevea-Hedges weight-function model for publication bias is available in the R package weightr and as a shiny application (Coburn & Vevea, 2017). | | The maximum-bias forest plot visualizes the sensitivity of the meta-analytic summary effect to missing studies due to publication bias, using methods introduced in Copas and Jackson (2004). Summary effects and their 95% confidence intervals are shown for the fixed-effect model as well as for the random-effects model. This allows assessing the effect of missing studies for both of this widely used meta-analytic models. The above plot was created using the R package metasens (Schwarzer, Carpenter, & Rücker, 2017). | |

| Network graph [11.1] | Flow-of-evidence graph [11.1.1] | 3D network graph [11.1.2] |
| --- | --- | --- |
|  |  |  |
| Network graphs (Lumley, 2002) visualize the structure of the treatment networks; that is, which treatments have been directly compared in trials. A network graph consists of vertices for each treatment in the network and connecting edges for direct treatment comparisons. The size of the vertices and thickness of edges can be used to depict treatment-specific and comparison-specific information (e.g., the number of trials examining a specific treatment, or the sum of all patients in trials comparing a pair of treatments). The above example plot was created using the R package pcnetmeta (Lin, Zhang, Hodges, & Chu, 2017). | A variant of the network graph [11.1] is the directed, acyclic flow-of-evidence graph which allows to explore the role of direct and indirect comparisons for a specific network estimate (König, Krahn, & Binder, 2013). For a specific treatment comparison (in the above example treatment A vs. C), the flow-of-evidence graph visualizes the flow of evidence or contribution to the network estimate, based on direct and indirect evidence in the network. The width of the edges is proportional to the meta-analytic weight of a specific (direct) comparison, whereas a measure for the strength of the contribution is annotated at each contributing path. For further details, see König, Krahn, and Binder (2013). | A three-dimensional network graph has been proposed to visualize average covariate values for each study contributing direct evidence to a specific treatment comparison (Batson, Score, & Sutton, 2017). The 3D network graph therefore allows exploring whether there are covariate differences between treatment comparisons, which might be a reason for inconsistencies in the network. An online tool to create 3D network graphs has been provided at <http://3dnma.com/> (Batson, Score, & Sutton, 2017). |

| Matrix display of treatment comparisons [11.1.3] | Contrast forest plot [11.2] | Summary forest plot matrix [11.2.1] |
| --- | --- | --- |
|  |  |  |
| Another possibility to visualize the structure of a treatment network is a matrix display showing for which treatment comparisons direct evidence exists and which treatments have not yet been directly compared in primary studies. A published example for the application of this display can be found in Palmer and Sterne (2015, p. 338). | The contrast forest plot is a forest plot [1.2] used in the context of network meta-analysis. For a specified reference treatment (e.g., placebo), the estimated relative effects (contrasts) are shown with their confidence intervals (Song, Harvey, & Lilford, 2008). In addition, prediction intervals (see also [9.13.3]) for each treatment, compared to its reference, have been used and are shown within these plots (for an example, see Chaimani, Higgins, Mavridis, Spyridonos, & Salanti, 2013). | The summary forest plot matrix summarizes a multitude of information from a network meta-analysis (Tan, Cooper, Bujkiewicz, Welton, Caldwell, & Sutton, 2014). For each treatment in the network, on the diagonal the estimated ranking probabilities are shown in a rankogram [11.7], including the median rank. In the lower triangle of the matrix, for each corresponding treatment comparison the network estimate of the effect size, as well as the direct estimate from pairwise meta-analysis, are shown in a forest plot with 95% credibility intervals. The corresponding numerical values are shown at the upper triangle matrix. The above plot was created using example code and data provided by Tan et al. (2014). |

| Summary forest plot table [11.2.2] | Network indirect path decomposition forest plot [11.2.3] | Invariant interval forest plot [11.2.4] |
| --- | --- | --- |
|  |  |  |
| The summary forest plot table was proposed alongside the summary forest plot matrix [11.2.1] (Tan, Cooper, Bujkiewicz, Welton, Caldwell, & Sutton, 2014). In essence, the display conveys the same information as the summary forest plot matrix, but the display is arranged differently and lacks rankograms [11.7] for each treatment. In addition, the number of studies comparing treatments directly (head-to-head) is provided. The above plot was created by using example code and data provided by Tan et al. (2014). | For a comparison of two treatments, the estimated relative effects are shown and contrasted for the direct effect (pairwise meta-analysis) and effect estimates obtained indirectly via other treatment comparisons in the network. Differences in directly and indirectly obtained estimates indicate inconsistencies in the network. This plot has been presented alongside a corresponding network graph [11.1] (for further details, see Krahn, Binder, & König, 2014). | The invariant interval forest plot is a contrast forest plot [11.2], additionally showing invariant intervals (Phillippo, Dias, Ades, Didelez, Welton, 2017). Invariant intervals show the necessary change (bias adjustment) in the estimated treatment contrast, such that the optimal treatment decision changes. The display therefore allows assessing the sensitivity of the estimated treatment rankings. In the above example, the optimal treatment is treatment number 3. Contrasts, for which the necessary bias adjustment is within the 95% credibility interval, are highlighted in red. The above plot was created with the R package nmathresh (Phillippo, 2018) and example data therein. |

| Checkerboard unit plot [11.3] | Treatment dissimilarity table plot [11.4] | MDS inconsistency plot [11.4.1] |
| --- | --- | --- |
|  |  |  |
| Checkerboard scores have been used as a measure for the tendency of two treatments not to be compared against each other in primary studies (Salanti, Kavvoura, & Ioannidis, 2008). The checkerboard unit plot shows the checkerboard score for each possible treatment comparison in descending order from left to right. Treatment comparisons with the highest checkerboard scores have been directly compared the least. This can point to gaps in the literature at hand. The curvature of treatment comparisons from left to right can indicate whether selective treatment comparisons in the network are driven by just a few heavily under-researched comparisons or, alternatively, by numerous slightly underused comparisons. For further details, see Salanti, Kavvoura, and Ioannidis (2008). | The treatment dissimilarity table plot shows for each pair of treatments, for which direct evidence exists, a measure of treatment dissimilarity, such as the estimated treatment effect contrast from a pairwise meta-analysis (Chung, & Lumley, 2008). Bubbles proportional in size to the dissimilarity measure are shown as well, highlighting the most dissimilar treatments (i.e., those with the largest relative treatment differences). | The MDS inconsistency plot shows all treatments in a scatterplot, with positions found via multidimensional scaling, using the treatment dissimilarities (as visualized in [11.4]) as the distance metric. Treatments are therefore mapped onto a two-dimensional space, such that their pairwise distances are preserved. Directly compared treatments are connected with lines. For a perfectly consistent treatment network, all treatments should lie on a straight line; that is, only one dimension is necessary to preserve all pairwise treatment differences. For further details, see Chung and Lumley (2008). |

| Ranking table plot [11.5] | Barplot of ranking probabilities [11.5.1] | Median rank chart [11.5.2] |
| --- | --- | --- |
|  |  |  |
| As an alternative to the rankogram [11.7], the ranking table plot also shows for each treatment (rows) the estimated probability for each rank (columns). In addition, for each cell in the table plot, a bubble proportional in size to the corresponding probability is shown. Probabilities within a specific row or column sum up to unity. For further details, see Hawkins, Scott, and Woods (2009). | An alternative to the ranking table plot [11.5] is to show the probabilities for each treatment to have a certain rank in stacked barplots. For further details, see Salanti, Ades, and Ioannidis (2011). | The median rank chart is an easy way to present the obtained rankings of treatments in a network meta-analysis. Each treatment is shown at its (median) rank from the estimated treatment-specific ranking probabilities in a shaded barchart (Tan, Cooper, Bujkiewicz, Welton, Caldwell, & Sutton, 2014). The above plot was created using example code and data provided by Tan et al. (2014). |
| Ranking and ranking probability for different outcome preferences [11.5.3] | Ranking scatterplot for single outcome [11.5.4] | Inconsistency plot [11.6] |
|  |  |  |
| A visualization tool has been proposed to interactively rank treatments by more than one outcome of interest (Naci, 2016). The plot consists of rankograms [11.7] for each treatment, differentiated by several outcomes. The overall ranking of treatments is determined by user-defined preferences for these different outcomes. The above plot was created with the free online statin ranking tool available at http://lse.live.kiln.digital/statins/. For further details, see Naci (2016). | The surface under the cumulative ranking curve (SUCRA; the area under the cumulative rankogram [11.7]) is regularly used to rank treatments in the context of Bayesian network meta-analysis. Treatments are plotted in a scatterplot, with the SUCRA value on the x axis and the corresponding rankings on the y axis. This display therefore combines the discrete ranking information with the underlying continuous decision rule. Alternatively, instead of SUCRA values, the unique dimension from the multi-dimensional scaling approach has also been used as the ranking criterion on the x axis in this plot. For further details, see Chaimani, Higgins, Mavridis, Spyridonos, and Salanti (2013). | The inconsistency plot shows the estimated inconsistency parameter for each closed loop (i.e., the difference between directly and indirectly estimated treatment effects) with 95% confidence intervals. Significant deviations from zero are signs for inconsistencies in the network and for a possible violation of the transitivity assumption in certain loops within the network (Salanti, Marinho, & Higgins, 2009). |

| Rankogram (cumulative or absolute) [11.7] | Probability to be within a range of the best treatment plot [11.7.1] | Network meta-regression plot [11.8] |
| --- | --- | --- |
|  |  |  |
| Rankograms show the estimated probability distribution of ranks for each treatment, similar to [11.5]. For each treatment, the probability that this treatment has a specific rank (according to a criterion of interest) is shown. Either the discrete probability distribution of the ranks is visualized with barplots, or the cumulative distribution function is shown. The area under the cumulative distribution function – also known as the surface under the cumulative ranking curve (SUCRA) – is a widely used summary statistic to assign single ranks to each treatment. For further details, see Cipriani et al. (2009). | Shown for each treatment is the estimated probability that it is not worse by a certain threshold than a reference treatment, according to a criterion of interest. The display therefore allows assessing the sensitivity of the rankings and the likely relative comparisons between treatments and a reference treatment. For further details, see Salanti, Ades, and Ioannidis (2011). | Meta regression can be used to explain sources of heterogeneity and/or inconsistency in network meta-analyses. Network meta-regression plots have been used to visualize the association of effect moderators with the estimated effectiveness of treatments, as compared to a reference treatment (e.g., placebo) in the network (for an early published example, see Cooper, Sutton, Morris, Ades, & Welton, 2009). |
| Contribution network meta-regression plot [11.8.1] | Heat network meta-regression plot [11.8.2] | Diagnostic network plot (leverage, deviance, residuals) [11.9] |
|  |  |  |
| For a treatment comparison of interest, the contribution network meta-regression plot (Donegan, Dias, Tudur-Smith, Marinho, & Welton, 2018) shows the estimated network meta-regression for the relative treatment effect with 95% credibility interval (bottom panel). Effect estimates and covariate values for studies contributing direct evidence to the network meta-regression estimates are depicted by separate bubbles, with their size proportional to their contribution to estimating the slope (blue) and intercept (red). Covariate values and the contribution of studies contributing indirect evidence to the network meta-regression estimates are depicted in a separate dot plot (top panel). The above plot was created using published example code and data (Donegan, Dias, Tudur‐Smith, Marinho, & Welton, 2018). | The heat network meta-regression plot shows the estimated network meta-regression line and 95% credibility intervals for a specific treatment comparison (Donegan, Dias, Tudur-Smith, Marinho, & Welton, 2018). Contrary to [11.8.1], no study-level estimates are shown. Instead, for intervals of covariate values, the contribution of all trials with these covariate values to the meta-regression estimates are shown by colored areas. The color above the regression line indicates the contribution to the intercept, and the color below the regression line indicates the contribution to the estimated slope parameter. The above plot was created using published example code and data (Donegan, Dias, Tudur‐Smith, Marinho, & Welton, 2018). | A diagnostic network plot has been proposed to assess the fit of a network meta-regression model and to identify influential data points (Cooper, Sutton, Morris, Ades, & Welton, 2009). For each treatment effect, the corresponding leverage and the square root of the residual deviance values are shown. Contours show the contribution to the DIC statistic for each data point. Data points lying on a specific contour contribute a value equal to the contour value at residual deviance of zero (in the above example, contours are at values of 1, 2, 3, or 4). The DIC statistic is a measure of model fit, accounting for model complexity. Data points with high leverage and high residual deviance (and therefore large contributions to the DIC statistic) indicate influential data points which the model does not fit well. The above plot was created with the R package gemtc (van Valkenhoef & Kuiper, 2016) and example data therein. |
| Comparison-adjusted funnel plot [11.10] | Contribution plot [11.11] | Netheat plot [11.12] |
|  |  |  |
| The comparison-adjusted funnel plot allows assessing small-study effects in the context of network meta-analysis (Chaimani, Higgins, Mavridis, Spyridonos, & Salanti, 2013). Different treatment comparisons correspond to different meta-analytic summary effect estimates. Therefore, for each study, the difference of the treatment effect and treatment-specific (pairwise) meta-analytic summary effect is used on the x axis. For each study, either the treatment effect, using a common comparator (e.g., placebo), can be used, with studies reporting the same comparisons being indicated by the same color or shape in the funnel plot. Alternatively, treatment comparisons with a characteristic of interest presumably driving a small study-effect can be used for each study (e.g., the newer treatment is compared to the older treatment throughout). For further details, see Chaimani, Higgins, Mavridis, Spyridonos, and Salanti (2013). | The contribution plot is a table plot showing the contribution of direct (pairwise) treatment comparisons (columns) to each network treatment comparison estimate, using direct (if available) and indirect evidence (rows). The contribution of each direct comparison depends on the variance of the estimate, as well as on the structure and complexity of the network. For further details, see Krahn, Binder, and König (2013). | The netheat plot was proposed to identify drivers of inconsistency in a meta-analytic network (Krahn, Binder, & König, 2013). For each mixed treatment comparison, using direct and indirect evidence (rows), the contribution of direct comparisons (columns) to the network estimate is visualized with proportionally sized gray boxes, identical to the contribution plot [11.11]. In addition, heat colors are used to indicate direct comparisons potentially responsible for inconsistencies in the network. The above plot was created using the R package netmeta (Rücker, Schwarzer, Krahn, & König, 2018). |
| Shade plot for contrast weights of treatment comparison [11.13] | Shade plot for *p* values of treatment comparison [11.14] | Minimal parallelism vs. mean path length scatterplot [11.15] |
|  |  |  |
| Whereas the contribution plot [11.11] shows the contribution of different comparisons to network estimates, the shade plot for contrast weights of treatment comparison depicts the respective contribution on the study level. The weight of each study for the estimation of the network estimate of each treatment, as compared to a specified reference treatment (e.g., placebo), is shown. For further details, see Senn, Gavini, Magrez, and Scheen (2013). | The shade plot for *p* values of treatment comparisons depicts the *p* value for each estimated treatment comparison in a heatmap-like display. Whether a *p* value for the comparison of one treatment (column) with another treatment (row) is above or below conventional significance thresholds, is indicated by categorical shading. For further details, see Senn, Gavini, Magrez, and Scheen (2013). | The minimal parallelism and mean path length are descriptive statistics for the role of indirect evidence in a meta-analytic treatment network (König, Krahn, & Binder, 2013). In essence, the minimal parallelism statistic shows how many indirect paths in the network contribute to estimating a certain treatment comparison. The mean path length is a measure for indirectness; that is, whether a network estimate is primarily driven by indirect evidence over several paths. High values of parallelism are desirable for consistent networks and necessary to detect inconsistencies in the subnetwork contributing to a specific treatment comparison. Showing both statistics in a scatterplot allows assessing whether estimates are based on reliable indirect evidence. Comparisons based on a single chain of indirect comparisons (high mean path length, low minimal parallelism) should be interpreted cautiously. |
| Hsu mean-mean plot [11.16] | Clustered ranking plot for two outcomes [11.17] | Network risk of bias chart [11.18] |
|  |  |  |
| The Hsu mean-mean plot is a way to visualize a very large number of different treatment comparisons in a network meta-analysis. For each treatment, the treatment effect relative to a reference treatment is shown with horizontal lines, as well as with vertical lines. The corresponding confidence interval lines are drawn diagonally at each intersection of these effect lines. Any comparison between treatments is significant, if the corresponding confidence interval does not overlap the 45-degree reference line. This is also indicated by dashed lines for non-significant treatment differences. For further details, see Senn, Gavini, Magrez, and Scheen (2013). | The clustered ranking plot shows SUCRA (surface under the cumulative ranking curve) values (or other summary ranking statistics) for two outcomes simultaneously for each treatment in a scatterplot. In addition, treatment clusters are found via cluster analysis of the outcome values of interest, and treatments forming the same clusters are indicated by the same color. For further details, see Chaimani, Higgins, Mavridis, Spyridonos, and Salanti (2013). | The network risk of bias chart visualizes for each estimated mixed treatment comparison the contribution of direct comparisons (in %) to the network estimates in a barchart (left-hand side; also see contribution plot [11.11]). The contribution of direct comparisons is color-coded according to the assessed risk of bias (using the GRADE approach). The risk of bias grading for each comparison, when only considering direct evidence (if available), is additionally shown on the right-hand side. For further details, see Salanti, Del Giovane, Chaimani, Caldwell, and Higgins (2014). |

| Network risk of bias direct evidence contribution chart [11.18.1] | Bland-Altman heterogeneity plot [11.19] | Rank heat plot [11.20] |
| --- | --- | --- |
|  |  |  |
| For comparisons for which direct evidence exists, the overall contribution of this direct evidence to all network estimates is shown in a pie chart. Each direct comparison is color-coded by its risk of bias assessment (as also depicted in [11.18] on the right-hand side). For further details, see Salanti, Del Giovane, Chaimani, Caldwell, and Higgins (2014). | The Bland-Altman heterogeneity plot was specifically proposed to explore heterogeneity in the context of network meta-analysis. As a special case of the meta-analytic Bland-Altman plot [8.8], it shows for each trial and pairwise treatment comparison the mean of the obtained estimated treatment effects within a fixed-effect and a random-effects network model on the x axis and the respective difference of these estimates on the y axis. Studies with treatment comparisons which strongly deviate from the horizontal line at zero might be drivers of heterogeneity in the network. For further details, see Schwarzer, Carpenter, and Rücker (2015, pp. 212-213). | The rank heat plot was proposed to compare treatments on several different outcome measures simultaneously (Veroniki, Straus, Fyraridis, & Tricco, 2016). Each outcome measure of interest is depicted by a circle, with one segment for each treatment. For more than one outcome of interest, the corresponding circles are arranged within each other in decreasing size. The size of the circles and the segments carry no information. For each treatment and outcome of interest, the segment is color-coded according to the probability to be the best-ranked treatment. Treatments for which no data are available for a specific outcome are displayed in white. The above example plot was created using and adapting R code from Veroniki, Straus, Fyraridis, and Tricco (2016). |
| Hasse diagram [11.21] | Partial orderings for two outcomes plot [11.22] | Biplot of partial orderings for more than two outcomes [11.22.1] |
|  |  |  |
| The Hasse diagram was proposed for visualizing the ranking of treatments according to more than one outcome preference (e.g., acceptability, efficacy, cost) in the context of frequentist network meta-analysis and is based on the concept of partial ordering (Rücker & Schwarzer, 2017). The arrangement of treatments reflects their partial ordering, with treatments at the top estimated to be superior to treatments farther down below. Arrows additionally point from superior treatments down to inferior treatments. Arrows already implied by others are omitted for clarity (e.g., in the plot above E is superior to F and F superior to C; therefore, no arrow from E to C is drawn). The above plot was created using the R package netmeta (Rücker, Schwarzer, Krahn, & König, 2018). | A scatterplot has been proposed to show superiority or inferiority of treatments to one another on two outcome measures simultaneously (as in [11.17]) and incorporates partial ordering information (Rücker & Schwarzer, 2017). For each treatment, summary ranking measures (e.g., SUCRA values) for two outcomes are shown. In addition, treatments are connected using the same logic than in drawing the arrows on the Hasse diagram [11.21], additionally showing the partial ordering of treatments. In contrast to the Hasse diagram, the scatterplot also contains information on the outcome-specific, continuous performance of each treatment. The above plot was created using the R package netmeta (Rücker, Schwarzer, Krahn, & König, 2018). | Partial orderings and treatment comparisons, as in [11.22], can also be displayed for more than two outcomes. For this purpose, outcome measures (e.g., SUCRA values) are first mapped onto the two-dimensional space using principal component analysis (Rücker & Schwarzer, 2017). The above plot was created using the R package netmeta (Rücker, Schwarzer, Krahn, & König, 2018). |

| Invariant region plot [11.23] | Bivariate network meta-analysis crosshair plot [11.24] | Covariate distribution plot [11.25] |
| --- | --- | --- |
|  |  |  |
| Proposed alongside the invariant interval forest plot [11.2.4], the invariant region plot allows assessing the sensitivity of the estimated optimal treatment by considering hypothetical bias adjustment for two treatment comparisons simultaneously (Phillippo, Dias, Ades, Didelez, & Welton, 2017). The invariant region shows for which adjustments in two contrasts the optimal treatment decision (in the example above treatment number 3) stays the same. Adjustments outside the region would lead to a new optimal treatment decision. The above plot was created with the R package nmathresh (Phillippo, 2018) and example data therein. | The bivariate network meta-analysis crosshair plot is a meta-analytic cross-hair plot [8.3.2] used in network meta-analysis to visualize estimated treatment effects on two outcomes of interest simultaneously. For a common reference treatment (e.g., placebo; treatment A in the above plot), the relative effects for all other treatment are shown on two dimensions as cross-hairs with 95% credibility intervals. For a published application of this display in the context of network meta-analysis, see Cipriani et al. (2018). | The covariate distribution plot was proposed to visualize the distribution of a covariate of interest in a network (Donegan, Dias, Tudur-Smith, Marinho, & Welton, 2018). Similar to other network graphs [11.1], it shows which treatments are directly compared, but for each (direct) treatment comparison, the distribution of the covariate of interest is visualized using a histogram. The covariate distribution plot can be seen as a two-dimensional alternative to the 3D network graph [11.1.2]. The above plot was created using published example data (Donegan, Dias, Tudur‐Smith, Marinho, & Welton, 2018). |

| Covariate contribution scatterplot [11.26] | Covariate contribution heat plot [11.27] | Network meta-analysis survival plot [11.28] |
| --- | --- | --- |
|  |  |  |
| The covariate contribution scatterplot (Donegan, Dias, Tudur‐Smith, Marinho, & Welton, 2018) visualizes for each treatment comparison the network meta-regression estimates (intercept and slope). The contribution to these network estimates and the covariate value of interest (e.g., average age) for all contributing studies are shown in separate scatterplots for each comparison. Therefore, it allows to assess the distribution of covariate values and to examine which covariate values are predominately present for studies with high contributions to specific network meta-regression estimates. The above plot was created using published example data (Donegan, Dias, Tudur‐Smith, Marinho, & Welton, 2018). | The covariate contribution heat plot (Donegan, Dias, Tudur-Smith, Marinho, & Welton, 2018) visualizes the contribution of trials with certain covariate values to network meta-regression estimates in a heatmap display. The display allows to depict which typical covariate values were present in studies driving the network estimates (i.e., had the highest contribution). Treatment effect estimates for covariate values not present, or hardly contributing to the network estimates, should be interpreted cautiously (because these constitute extrapolations). The above plot was created using published example code and data (Donegan, Dias, Tudur‐Smith, Marinho, & Welton, 2018). | Estimated treatment-specific survival curves over time, as well as corresponding hazard ratios of treatments against a common reference treatment (e.g., placebo), have been used to visualize network meta-analyses of time-to-event data. For a published example, see Dias, Ades, Welton, Jansen, and Sutton (2018, p.299). |

Anzures-Cabrera, J., & Higgins, J. P. (2010). Graphical displays for meta-analysis: An overview with suggestions for practice. *Research Synthesis Methods*, *1*, 66-80.

Babatunde, O. O., Tan, V., Jordan, J. L., Dziedzic, K., ChewGraham, C. A., Jinks, C., ... van der Windt, D. A. (2018). Evidence flowers: An innovative, visual method of presenting “best evidence” summaries to health professional and lay audiences. *Research Synthesis Methods*, *9*, 273-284.

Barendregt, J. J., & Doi, S. (2016) *MetaXL User Guide*. Sunrise Beach, Australia: EpiGear International.

Barrowman, N. J., Fang, M., Sampson, M., & Moher, D. (2003). Identifying null meta-analyses that are ripe for updating. *BMC Medical Research Methodology, 3*, 13.

Barrowman, N. J., & Myers, R. A. (2003). Raindrop plots: A new way to display collections of likelihoods and distributions. *American Statistician*, *57*, 268-274.

Batson, S., Score, R., & Sutton, A. J. (2017). Three-dimensional evidence network plot system: Covariate imbalances and effects in network meta-analysis explored using a new software tool. *Journal of Clinical Epidemiology*, *86*, 182-195.

Baujat, B., Mahé, C., Pignon, J. P., & Hill, C. (2002). A graphical method for exploring heterogeneity in metaanalyses: Application to a metaanalysis of 65 trials. *Statistics in Medicine*, *21*, 2641-2652.

Bax, L., Yu, L. M., Ikeda, N., Tsuruta, H., & Moons, K. G. (2006). Development and validation of MIX: Comprehensive free software for meta-analysis of causal research data. *BMC Medical Research Methodology*, *6*, 50.

Beath, K. J. (2014). A finite mixture method for outlier detection and robustness in meta-analysis. *Research Synthesis Methods*, *5*, 285-293.

Beath, K. J. (2016). metaplus: An R package for the analysis of robust meta-analysis and meta-regression, *R Journal*, *8*, 5-16.

Begg, C. B., & Mazumdar, M. (1994). Operating characteristics of a rank correlation test for publication bias. *Biometrics*, *50*, 1088-1101.

Bettany-Saltikov, J. (2012). *How to do a systematic literature review in nursing: A step-by-step guide*. Maidenhead, UK: Open University Press.

Bland, J. M., & Altman, D. (1986). Statistical methods for assessing agreement between two methods of clinical measurement. *Lancet*, *327*, 307-310.

Bowden, J., & Jackson, C. (2016). Weighing evidence “steampunk” style via the Meta-Analyser. *American Statistician*, *70*, 385-394.

Bowden, J., Tierney, J. F., Copas, A. J., & Burdett, S. (2011). Quantifying, displaying and accounting for heterogeneity in the meta-analysis of RCTs using standard and generalised Q statistics. *BMC Medical Research Methodology*, *11*, 41.

Brannick, M. T., & Gültaş, M. (2017). Cross-hairs: A scatterplot for metaanalysis in R. *Research Synthesis Methods*, *8*, 53-63.

Chaimani, A., Higgins, J. P., Mavridis, D., Spyridonos, P., & Salanti, G. (2013). Graphical tools for network meta-analysis in STATA. *PLOS ONE*, *8*, e76654.

Chambers, J. M., Cleveland, W. S., Kleiner, B., & Tukey, P. A. (1983). *Graphical methods for data analysis*. Pacific Grove, CA: Wadsworth & Brooks/Cole.

Cheung, M. W. L. (2015). metaSEM: An R package for meta-analysis using structural equation modeling. *Frontiers in Psychology*, *5*, 1521.

Cheung, M. W. L., & Chan, W. (2005). Meta-analytic structural equation modeling: A two-stage approach. *Psychological Methods*, *10*, 40-64.

Chevance, A., Schuster, T., Steele, R., Ternès, N., & Platt, R. W. (2015). Contour plot assessment of existing meta-analyses confirms robust association of statin use and acute kidney injury risk. *Journal of Clinical Epidemiology*, *68*, 1138-1143.

Choi, J. K., Yu, U., Kim, S., & Yoo, O. J. (2003). Combining multiple microarray studies and modeling interstudy variation. *Bioinformatics*, *19*, i84-i90.

Chung, H., & Lumley, T. (2008). Graphical exploration of network meta-analysis data: The use of multidimensional scaling. *Clinical Trials*, *5*, 301-307.

Cipriani, A., Furukawa, T. A., Salanti, G., Chaimani, A., Atkinson, L. Z., Ogawa, Y., ... Egger, M. (2018). Comparative efficacy and acceptability of 21 antidepressant drugs for the acute treatment of adults with major depressive disorder: a systematic review and network meta-analysis. *Lancet*, *391*, 1357-1366.

Cipriani, A., Furukawa, T. A., Salanti, G., Geddes, J. R., Higgins, J. P., Churchill, R., ... Tansella, M. (2009). Comparative efficacy and acceptability of 12 new-generation antidepressants: A multiple-treatments meta-analysis. *Lancet*, *373*, 746-758.

Cleveland, W. S., & McGill, R. (1984). Graphical perception: Theory, experimentation, and application to the development of graphical methods. *Journal of the American Statistical Association*, *79*, 531–554.

Coburn, K. M., & Vevea, J. L. (2017). weightr [R software package]. Retrieved from <https://CRAN.R-project.org/package=weightr>

Cohen, J. (1992). A power primer. *Psychological Bulletin*, *112*, 155-159.

Cooper, H., & Hedges, L. V. (Eds.) (1994). *The handbook of research synthesis*. New York: Russell Sage.

Cooper, N. J., Sutton, A. J., Morris, D., Ades, A. E., & Welton, N. J. (2009). Addressing between-study heterogeneity and inconsistency in mixed treatment comparisons: application to stroke prevention treatments in individuals with non-rheumatic atrial fibrillation. *Statistics in Medicine*, *28*, 1861-1881.

Copas, J., & Jackson, D. (2004). A bound for publication bias based on the fraction of unpublished studies. *Biometrics*, *60*, 146–53.

Copas, J., & Shi, J. Q. (2000). Meta-analysis, funnel plots and sensitivity analysis. *Biostatistics*, *1*, 247-262.

Crowther, M., Avenell, A., MacLennan, G., & Mowatt, G. (2011). A further use for the harvest plot: A novel method for the presentation of data synthesis. *Research Synthesis Methods*, *2*, 79-83.

Crowther, M. J., Langan, D., & Sutton, A. J. (2012). Graphical augmentations to the funnel plot to assess the impact of additional evidence on a meta-analysis. *Stata Journal*, *12*, 605-622.

Dear, K. B., & Begg, C. B. (1992). An approach for assessing publication bias prior to performing a meta-analysis. *Statistical Science*, *7*, 237-245.

Deeks, J. J., & Altman, D. G. (2001) *Effect measures for meta-analysis of trials with binary outcomes*. In Egger, M., Smith, G. D., & Altman, D. G. (Eds.), *Systematic reviews in health care: Meta-analysis in context* (2nd ed., pp. 313-335). London: BMJ Books.

Dewey, M. (2018). metap [R software package]. Retrieved from <https://CRAN.R-project.org/package=metap>

Dias, S., Ades, A. E., Welton, N. J., Jansen, J. P., & Sutton, A. J. (2018). *Network meta-analysis for decision-making*. Chichester, UK: Wiley-Blackwell.

Donegan, S., Dias, S., Tudur-Smith, C., Marinho, V., & Welton, N. J. (2018). Graphs of study contributions and covariate distributions for network meta‐regression. *Research Synthesis Methods*, *9*, 243-260.

Dusseldorp, E., van Genugten, L., van Buuren, S., Verheijden, M. W., & van Empelen, P. (2014). Combinations of techniques that effectively change health behavior: Evidence from meta-CART analysis. *Health Psychology*, 33, 1530-1540.

Duval, S., & Tweedie, R. (2000). Trim and fill: A simple funnel-plot-based method of testing and adjusting for publication bias in meta-analysis. *Biometrics*, *56*, 455-463.

Eddy, D. M., Hasselblad, V., & Shachter, R. (1992). *Meta-analysis by the confidence profile method: The statistical synthesis of evidence*. Boston: Academic Press.

Egger, M., Smith, G. D., Schneider, M., & Minder, C. (1997). Bias in meta-analysis detected by a simple, graphical test. *British Medical Journal*, *315*, 629-634.

Elvik, R. (1998). Evaluating the statistical conclusion validity of weighted mean results in meta-analysis by analysing funnel graph diagrams. *Accident Analysis & Prevention*, *30*, 255-266.

Feichtinger, J., McFarlane, R. J., & Larcombe, L. D. (2012). CancerMA: A web-based tool for automatic meta-analysis of public cancer microarray data. *Database, 2012,* bas055.

Fogh, I., Ratti, A., Gellera, C., Lin, K., Tiloca, C., Moskvina, V., ... Gentilini, D. (2013). A genome-wide association meta-analysis identifies a novel locus at 17q11. 2 associated with sporadic amyotrophic lateral sclerosis. *Human Molecular Genetics*, *23*, 2220-2231.

Formann, A. K. (2008). Estimating the proportion of studies missing for meta-analysis due to publication bias. *Contemporary Clinical Trials*, *29*, 732-739.

Freiman, J. A., Chalmers, T. C., Smith Jr, H., & Kuebler, R. R. (1978). The importance of beta, the type II error and sample size in the design and interpretation of the randomized control trial: Survey of 71 negative trials. *New England Journal of Medicine*, *299*, 690-694.

Furuya-Kanamori, L., & Doi, S. A. (2016). Angry birds, angry children, and angry meta-analysts: A reanalysis. *Perspectives on Psychological Science*, *11*, 408-414.

Galbraith, R. F. (1988). A note on graphical presentation of estimated odds ratios from several clinical trials. *Statistics in Medicine*, *7*, 889-894.

Gartlehner, G., Schultes, M. T., Titscher, V., Morgan, L. C., Bobashev, G. V., Williams, P., & West, S. L. (2017). User testing of an adaptation of fishbone diagrams to depict results of systematic reviews. *BMC Medical Research Methodology*, *17*, 169.

Gee, T. (2005). Capturing study influence: The concept of ‘gravity’ in meta-analysis. *Australian Counselling Research Journal*, *1*, 52-75.

Gerber, A. S., & Malhotra, N. (2008). Publication bias in empirical sociological research: Do arbitrary significance levels distort published results? *Sociological Methods & Research*, *37*, 3-30.

Glass, G. V. (1976). Primary, secondary, and meta-analysis of research. *Educational Researcher*, *5*, 3-8.

Glass, G. V. (1977). Integrating findings: The meta-analysis of research. *Review of Research in Education*, *5*, 351-379.

Glass, G. V., McGraw, B., & Smith, M. L. (1981). *Meta-analysis in social research*. Beverly Hills, CA: Sage.

Gronau, Q. F., van Erp, S., Heck, D. W., Cesario, J., Jonas, K. J., & Wagenmakers, E. J. (2017). A Bayesian model-averaged meta-analysis of the power pose effect with informed and default priors: The case of felt power. *Comprehensive Results in Social Psychology*, *2*, 123-138.

Gu, Z. (2014). circlize [R software package]. Retrieved from <https://CRAN.R-project.org/package=circlize>

Guerra, R., & Goldstein, D. R. (Eds.) (2010). *Meta-analysis and combining information in genetics and genomics*. Boca Raton, FL: CRC Press.

Gültaş, M., & Brannick, M. T. (2016). metaplotr [R software package]. Retrieved from <https://CRAN.R-project.org/package=metaplotr>

Han, B., & Eskin, E. (2012). Interpreting meta-analyses of genome-wide association studies. *PLOS Genetics*, *8*, e1002555.

Hanji, M. B. (2017). *Meta-analysis in psychiatry research: Fundamental and advanced methods*. Toronto: Apple Academic Press.

Harrison, S. (2017). ALBATROSS [Stata software module]. Retrieved from <https://ideas.repec.org/c/boc/bocode/s458296.html>

Harrison, S., Jones, H. E., Martin, R. M., Lewis, S. J., & Higgins, J. P. (2017). The albatross plot: A novel graphical tool for presenting results of diversely reported studies in a systematic review. *Research Synthesis Methods*, 8, 281-289.

Hattie, J. (2008). *Visible learning: A synthesis of over 800 meta-analyses relating to achievement*. London: Routledge.

Hawkins, N., Scott, D. A., Woods, B. S., & Thatcher, N. (2009). No study left behind: A network meta-analysis in non-small-cell lung cancer demonstrating the importance of considering all relevant data. *Value in Health*, *12*, 996-1003.

Haynes, W. A., Vallania, F., Tomczak, A., Sweeney, T. E., Bongen, E., Rao, A. M., & Purvesh, K. (2017). MetaIntegrator [R software package]. Retrieved from <https://CRAN.R-project.org/package=MetaIntegrator>

Heck, D. W., Gronau, Q. F., & Wagenmakers, E.-J. (2017). metaBMA [R software package]. Retrieved from <https://CRAN.R-project.org/package=metaBMA>

Hedges, L. V. & Vevea, J. L. (2005). *Selection method approaches*. In H. R. Rothstein, A. J. Sutton, & M. Borenstein (Eds.), *Publication bias in meta-analysis: Prevention, assessment and adjustments* (pp. 145-174). Chichester, West Sussex: Wiley.

Higgins, J. P. T., & Green, S. (Eds.) (2008). *Cochrane handbook for systematic reviews of interventions*. Chichester, West Sussex: Wiley.

Hyde, J. S., & Linn, M. C. (1986). *The psychology of gender: Advances through meta-analysis*. Baltimore, MD: Johns Hopkins University Press.

Ioannidis, J. P., Contopoulos-Ioannidis, D. G., & Lau, J. (1999). Recursive cumulative meta-analysis: A diagnostic for the evolution of total randomized evidence from group and individual patient data. *Journal of Clinical Epidemiology*, *52*, 281-291.

Ioannidis, J. P., & Trikalinos, T. A. (2007). An exploratory test for an excess of significant findings. *Clinical Trials*, *4*, 245-253.

Jimenez, F. J., Guallar, E., & Martín-Moreno, J. M. (1997). A graphical display useful for meta-analysis. *European Journal of Public Health*, *7*, 101-105.

Keus, F., Wetterslev, J., Gluud, C., & van Laarhoven, C. J. (2010). Evidence at a glance: Error matrix approach for overviewing available evidence. *BMC Medical Research Methodology*, *10*, 1.

König, J., Krahn, U., & Binder, H. (2013). Visualizing the flow of evidence in network meta-analysis and characterizing mixed treatment comparisons. *Statistics in Medicine*, *32*, 5414-5429.

Koricheva, J., Gurevitch, J., & Mengersen, K. (Eds.). (2013). Handbook of meta-analysis in ecology and evolution. Princeton, NJ: Princeton University Press.

Krahn, U., Binder, H., & König, J. (2013). A graphical tool for locating inconsistency in network meta-analyses. *BMC Medical Research Methodology*, *13*, 1.

Krahn, U., Binder, H., & König, J. (2014). Visualizing inconsistency in network meta-analysis by independent path decomposition. *BMC Medical Research Methodology*, *1*, 131.

Krzywinski, M., Schein, J., Birol, I., Connors, J., Gascoyne, R., Horsman, D., ... Marra, M. A. (2009). Circos: An information aesthetic for comparative genomics. *Genome Research*, *19*, 1639-1645.

Kulinskaya, E., & Koricheva, J. (2010). Use of quality control charts for detection of outliers and temporal trends in cumulative meta-analysis. *Research Synthesis Methods*, *1*, 297-307.

L’Abbé, K. A., Detsky, A. S., & O’Rourke, K. (1987). Meta-analysis in clinical research. *Annals of Internal Medicine*, 107, 224-233.

Langan, D., Higgins, J. P., Gregory, W., & Sutton, A. J. (2012). Graphical augmentations to the funnel plot to assess the impact of additional evidence on a meta-analysis. *Journal of Clinical Epidemiology*, *65*, 511-519.

Laroche, P. (2015). *La méta-analyse: Méthodes et applications en sciences sociales*. Louvain-la-Neuve: De Boeck.

Lau, J., Antman, E. M., Jimenez-Silva, J., Kupelnick, B., Mosteller, F., & Chalmers, T. C. (1992). Cumulative meta-analysis of therapeutic trials for myocardial infarction. *New England Journal of Medicine*, *327*, 248-254.

Lau, J., Ioannidis, J. P., & Schmid, C. H. (1998). Summing up evidence: One answer is not always enough. *Lancet*, *351*, 123-127.

Lewis, S., & Clarke, M. (2001). Forest plots: Trying to see the wood and the trees. *British Medical Journal*, *322*, 1479-1480.

Light, R. J., & Pillemer, D. B. (1984). *Summing up: The science of reviewing research*. Cambridge, MA: Harvard University Press.

Lin, L., Zhang, J., Hodges, J. S., Chu, H. (2017). Performing arm-based network meta-analysis in R with the pcnetmeta package. *Journal of Statistical Software*, *80*, 1-25.

Lumley, T. (2002). Network meta-analysis for indirect treatment comparisons. *Statistics in Medicine*, *21*, 2313-2324.

Mathur, M. B., & VanderWeele, T. J. (2017, July 31). Sensitivity analysis for unmeasured confounding in meta-analyses. Retrieved from <https://arxiv.org/pdf/1707.09076.pdf>

Mathur, M. B., Tyler, J., & VanderWeele, T. J. (2017) ConfoundedMeta [R software package]. Retrieved from <https://CRAN.R-project.org/package=ConfoundedMeta>

Mavridis, D., Moustaki, I., Wall, M., & Salanti, G. (2017). Detecting outlying studies in meta-regression models using a forward search algorithm. *Research Synthesis Methods*, *8*, 199-211.

Moher, D., Liberati, A., Tetzlaff, J., & Altman, D. G. (2009). Preferred reporting items for systematic reviews and meta-analyses: The PRISMA statement. *Annals of Internal Medicine*, *151*, 264-269.

Moses, L. E., Shapiro, D., & Littenberg, B. (1993). Combining independent studies of a diagnostic test into a summary ROC curve: Data-analytic approaches and some additional considerations. *Statistics in Medicine*, *12*, 1293-1316.

Naci, H. (2016). Communication of treatment rankings obtained from network meta-analysis using data visualization. *Circulation: Cardiovascular Quality and Outcomes*, *9*, 605-608.

Nakagawa, S., Noble, D. W., Senior, A. M., & Lagisz, M. (2017). Meta-evaluation of meta-analysis: Ten appraisal questions for biologists. *BMC Biology*, *15*, 18.

Ng, M. C., Shriner, D., Chen, B. H., Li, J., Chen, W. M., Guo, X., ... Comeau, M. E. (2014). Meta-analysis of genome-wide association studies in African Americans provides insights into the genetic architecture of type 2 diabetes. *PLS Genetics*, *10*, e1004517.

Normand, S. L. T. (1999). Tutorial in biostatistics meta-analysis: Formulating, evaluating, combining, and reporting. *Statistics in Medicine*, *18*, 321-359.

Ogilvie, D., Fayter, D., Petticrew, M., Sowden, A., Thomas, S., Whitehead, M., & Worthy, G. (2008). The harvest plot: A method for synthesising evidence about the differential effects of interventions. *BMC Medical Research Methodology*, *8*, 1.

Olkin, I., Dahabreh, I. J., & Trikalinos, T. A. (2012). GOSH: A graphical display of study heterogeneity. *Research Synthesis Methods*, *3*, 214-223.

Olliaro, P., & Vaillant, M. T. (2010). Alternative visual displays of metaanalysis of malaria treatment trials to facilitate translation of research into policy. *Diagnostic Microbiology and Infectious Disease*, *68*, 422-431.

Palmer, T. M., & Sterne, J. A. C. (Eds.) (2015). *Meta-analysis in Stata: An updated collection from the Stata journal* (2nd ed.). College Station, TX: Stata Press.

Panesar, S. S., Rao, C., Vecht, J. A., Mirza, S. B., Netuveli, G., Morris, R., ... Athanasiou, T. (2009). Development of the Veritas plot and its application in cardiac surgery: An evidence-synthesis graphic tool for the clinician to assess multiple meta-analyses reporting on a common outcome. *Canadian Journal of Surgery*, *52*, E137-E145.

Peiffer, A. M., Maldjian, J. A., & Laurienti, P. J. (2008). Resurrecting brinley plots for a novel use: Meta-analyses of functional brain imaging data in older adults. *Journal of Biomedical Imaging*, *2008*, 167078.

Peters, J. L., Sutton, A. J., Jones, D. R., Abrams, K. R., & Rushton, L. (2008). Contour-enhanced meta-analysis funnel plots help distinguish publication bias from other causes of asymmetry. *Journal of Clinical Epidemiology*, *61*, 991-996.

Phillippo, D. M. (2018) nmathresh [R software package]. Retrieved from <https://CRAN.R-project.org/package=nmathresh>

Phillippo, D. M., Dias, S., Ades, A. E., Didelez, V., & Welton, N. J. (2018). Sensitivity of treatment recommendations to bias in network meta-analysis. *Journal of the Royal Statistical Society: Series A (Statistics in Society)*, *181*, 843-867.

Phillips, B., Stewart, L. A., & Sutton, A. J. (2010). ‘Cross hairs’ plots for diagnostic meta-nalysis. *Research Synthesis Methods*, *1*, 308-315.

Pogue, J. M., & Yusuf, S. (1997). Cumulating evidence from randomized trials: utilizing sequential monitoring boundaries for cumulative meta-analysis. *Controlled Clinical Trials*, *18*, 580-593.

Poorolajal, J., Mahmoodi, M., Majdzadeh, R., & Fotouhi, A. (2010). Metaplot: A novel stata graph for assessing heterogeneity at a glance. *Iranian Journal of Public Health*, *39*, 102-104.

R Core Team (2018). R: A language and environment for statistical computing. R Foundation for Statistical Computing, Vienna, Austria. URL <http://www.R-project.org/>

Radua, J. (2018). metansue [R package software]. Retrieved from <https://CRAN.R-project.org/package=metansue>

Radua, J., Schmidt, A., Borgwardt, S., Heinz, A., Schlagenhauf, F., McGuire, P., & Fusar-Poli, P. (2015). Ventral striatal activation during reward processing in psychosis: a neurofunctional meta-analysis. *JAMA Psychiatry*, *72*, 1243-1251.

Rhodes, D. R., Barrette, T. R., Rubin, M. A., Ghosh, D., & Chinnaiyan, A. M. (2002). Meta-analysis of microarrays. *Cancer Research*, *62*, 4427-4433.

Roberts, C. J., & Stanley, T. D. (Eds.) (2005). *Meta-regression analysis: Issues of publication bias in economics*. Malden, MA: Blackwell.

Roever, C. (2017). bayesmeta [R software package]. Retrieved from <https://CRAN.R-project.org/package=bayesmeta>

Rosenberg, M. S., Adams, D. C., & Gurevitch, J. (2000). *MetaWin: Statistical software for meta-analysis with resampling tests*. Sunderland, MA: Sinauer.

Rosenthal, R. (1984). *Meta-analytic procedures for social research*. Beverly Hills, CA: Sage.

Rothstein, H. R., Sutton, A. J., & Borenstein, M. (Eds.) (2005). *Publication bias in meta-analysis: Prevention, assessment and adjustments*. Chichester, West Sussex: Wiley.

Rubin, D. B. (1981). Estimation in parallel randomized experiments. *Journal of Educational Statistics*, *6*, 377-401.

Rücker, G., & Schwarzer, G. (2017). Resolve conflicting rankings of outcomes in network meta-analysis: Partial ordering of treatments. *Research Synthesis Methods*, *8*, 526-536.

Rücker G., Schwarzer G., Krahn U., & König J. (2018). netmeta [R software package]. Retrieved from <https://CRAN.R-project.org/package=netmeta>

Rudner, L., Glass, G. V., Evartt, D. L., Emery, P. J. (2002). *A user’s guide to the meta-analysis of research studies*. ERIC Clearinghouse on Assessment and Evaluation, University of Maryland, College Park. [<http://echo.edres.org:8080/meta/metaman.htm>]

Rukhin, A. L. (2015). Confidence regions and intervals for meta-analysis model parameters. *Technometrics*, *57*, 547-558.

Salanti, G., Ades, A. E., & Ioannidis, J. P. (2011). Graphical methods and numerical summaries for presenting results from multiple-treatment meta-analysis: An overview and tutorial. *Journal of Clinical Epidemiology*, *64*, 163-171.

Salanti, G., Del Giovane, C., Chaimani, A., Caldwell, D. M., & Higgins, J. P. (2014). Evaluating the quality of evidence from a network meta-analysis. *PLOS ONE*, *9*, e99682.

Salanti, G., Kavvoura, F. K., & Ioannidis, J. P. (2008). Exploring the geometry of treatment networks. *Annals of Internal Medicine*, *148*, 544-553.

Salanti, G., Marinho, V., & Higgins, J. P. (2009). A case study of multiple-treatments meta-analysis demonstrates that covariates should be considered. *Journal of Clinical Epidemiology*, *62*, 857-864.

Sandelowski, M., & Barroso, J. (2007). *Handbook for synthesizing qualitative research*. New York: Springer.

Scheibehenne, B., Jamil, T., & Wagenmakers, E. J. (2016). Bayesian evidence synthesis can reconcile seemingly inconsistent results: The case of hotel towel reuse. *Psychological Science*, *27*, 1043-1046.

Schild, A. H., & Voracek, M. (2013). Less is less: A systematic review of graph use in meta-analyses. *Research Synthesis Methods*, *4*, 209-219.

Schild, A. H., & Voracek, M. (2015). Finding your way out of the forest without a trail of bread crumbs: Development and evaluation of two novel displays of forest plots. *Research Synthesis Methods*, *6*, 74-86.

Schwarzer, G., Carpenter, J. R., & Rücker, G. (2015). *Meta-analysis with R*. Cham, Switzerland: Springer.

Schwarzer, G., Carpenter, J. R., & Rücker G. (2017). metasens [R software package]. Retrieved from <https://CRAN.R-project.org/package=metasens>

Schweder, T., & Spjøtvoll, E. (1982). Plots of *p*-values to evaluate many tests simultaneously. *Biometrika*, *69*, 493-502.

Senn, S., Gavini, F., Magrez, D., & Scheen, A. (2013). Issues in performing a network meta-analysis. *Statistical Methods in Medical Research*, *22*, 169-189.

Shadish, W. R., & Sweeney, R. B. (1991). Mediators and moderators in meta-analysis: There’s a reason we don’t let dodo birds tell us which psychotherapies should have prizes. *Journal of Consulting and Clinical Psychology*, *59*, 883-893.

Shen, Y., Zhu, H., Wan, C., Chen, L., Wang, T., Yang, T., & Wen, F. (2014). Can cholesterol be used to distinguish pleural exudates from transudates? Evidence from a bivariate meta-analysis. *BMC Pulmonary Medicine*, *14*, 61.

Simonsohn, U., Nelson, L. D., & Simmons, J. P. (2014). *P*-curve: A key to the file-drawer. *Journal of Experimental Psychology: General*, *143*, 534-547.

Song, F., Harvey, I., & Lilford, R. (2008). Adjusted indirect comparison may be less biased than direct comparison for evaluating new pharmaceutical interventions. *Journal of Clinical Epidemiology*, *61*, 455-463.

Stangl, D. K., & Berry, D. A. (2000). *Meta-analysis in medicine and health policy*. New York: Dekker.

Stanley, T. D., & Doucouliagos, H. (2014). Meta-regression approximations to reduce publication selection bias. *Research Synthesis Methods*, *5*, 60-78.

Steinhauser, S., Schumacher, M., & Rücker, G. (2016). Modelling multiple thresholds in meta-analysis of diagnostic test accuracy studies. *BMC Medical Research Methodology*, *16*, 97.

Sterne, J. A., & Egger, M. (2001). Funnel plots for detecting bias in meta-analysis: Guidelines on choice of axis. *Journal of Clinical Epidemiology*, *54*, 1046-1055.

Sterne, J. A. (Ed.) (2009). *Meta-analysis in Stata: An updated collection from the Stata Journal*. College Station, TX: Stata Press.

Sutton, A. J., Abrams, K. R., Jones, D. R., Sheldon, T. A., & Song, F. (2000). *Methods for meta-analysis in medical research*. Chichester, West Sussex: Wiley.

Sutton, A. J., Cooper, N. J., Jones, D. R., Lambert, P. C., Thompson, J. R., & Abrams, K. R. (2007). Evidence-based sample size calculations based upon updated meta-analysis. *Statistics in Medicine*, *26*, 2479-2500.

Tan, S. H., Cooper, N. J., Bujkiewicz, S., Welton, N. J., Caldwell, D. M., & Sutton, A. J. (2014). Novel presentational approaches were developed for reporting network meta-analysis. *Journal of Clinical Epidemiology*, *67*, 672-680.

The Cochrane Collaboration (2014). Review Manager (RevMan) (Version 5.3) [Computer program]. Copenhagen: The Nordic Cochrane Centre.

Taylor, A. E., & Munafò, M. R. (2016). Triangulating meta-analyses: The example of the serotonin transporter gene, stressful life events and major depression. *BMC Psychology*, *4*, 23.

Therneau, T., & Atkinson, B. (2018). rpart [R software package]. Retrieved from <https://CRAN.R-project.org/package=rpart>

Thompson, S. G. (1993). Controversies in meta-analysis: The case of the trials of serum cholesterol reduction. *Statistical Methods in Medical Research*, 2, 173-192.

Thompson, C. G. (2016). Graphing effects as fuzzy numbers in meta-analysis. *Journal of Modern Applied Statistical Methods*, *15*, 49.

Thomson, H. J., & Thomas, S. (2013). The effect direction plot: Visual display of non-standardised effects across multiple outcome domains. *Research Synthesis Methods*, *4*, 95-101.

Trikalinos, T. A., & Iaonnidis, J. P. A. (2005). *Assessing the evolution of effect sizes over time*. In H. R. Rothstein, A. J. Sutton, & M. Borenstein (Eds.), *Publication bias in meta-analysis: Prevention, assessment and adjustments* (pp. 241-260). Chichester, West Sussex: Wiley.

Tukey, J. W. (1977). *Exploratory data analysis*. Reading, MA: Addison-Wesley.

Turner, S. (2017). qqman [R software package]. Retrieved from <https://CRAN.R-project.org/package=qqman>

van Houwelingen, H. C., Zwinderman, K. H., & Stijnen, T. (1993). A bivariate approach to meta-analysis. *Statistics in Medicine*, *12*, 2273-2284.

van Lissa, C. J. (2017). metaforest: Exploring heterogeneity in meta-analysis using random forests. R package version 0.1.0. <https://CRAN.R-project.org/package=metaforest>

Van Uitert, M., Moerland, P. D., Enquobahrie, D. A., Laivuori, H., van der Post, J. A., Ris-Stalpers, C., & Afink, G. B. (2015). Meta-analysis of placental transcriptome data identifies a novel molecular pathway related to preeclampsia. *PLOS ONE*, *10*, e0132468.

van Valkenhoef, G., & Kuiper, J. (2016). gemtc [R software package]. Retrieved from <https://CRAN.R-project.org/package=gemtc>

Veroniki, A. A., Jackson, D., Viechtbauer, W., Bender, R., Bowden, J., Knapp, G., ... Salanti, G. (2016). Methods to estimate the between-study variance and its uncertainty in meta-analysis. *Research Synthesis Methods*, *7*, 55-79.

Veroniki, A. A., Straus, S. E., Fyraridis, A., & Tricco, A. C. (2016). The rank-heat plot is a novel way to present the results from a network meta-analysis including multiple outcomes. *Journal of Clinical Epidemiology*, *76*, 193-199.

Vevea, J. L., & Hedges, L. V. (1995). A general linear model for estimating effect size in the presence of publication bias. *Psychometrika*, *60*, 419-435.

Vevea, J. L., & Woods, C. M. (2005). Publication bias in research synthesis: Sensitivity analysis using a priori weight functions. *Psychological Methods*, *10*, 428-443.

Viechtbauer, W. (2010). Conducting meta-analyses in R with the metafor package. *Journal of Statistical Software*, *36*, 1-48.

Viechtbauer, W., & Cheung, M. W. L. (2010). Outlier and influence diagnostics for meta-analysis. *Research Synthesis Methods*, *1*, 112-125.

Villanueva, E. V., & Zavarsek, S. (2004). Evaluating heterogeneity in cumulative meta-analyses. *BMC Medical Research Methodology*, *4*, 18.

Voest, E. E., van Houwelingen, J. C., & Neijt, J. P. (1989). A meta-analysis of prognostic factors in advanced ovarian cancer with median survival and overall survival (measured with the log (relative risk)) as main objectives. *European Journal of Cancer*, *25*, 711-720.

Voracek, M., Kossmeier, M., & Tran, U. S. (2019). Which data to meta-analyze, and how? A specification-curve and multiverse-analysis approach to meta-analysis. *Zeitschrift für Psychologie, 227,* 64-82.

Walker, A. M., Martin-Moreno, J. M., & Artalejo, F. R. (1988). Odd man out: A graphical approach to meta-analysis. *American Journal of Public Health*, *78*, 961-966.

Wang, M. C., & Bushman, B. J. (1998). Using the normal quantile plot to explore meta-analytic data sets. *Psychological Methods*, *3*, 46-54.

Wang, X. V., Cole, B., Bonetti, M., & Gelber, R. D. (2016). Meta-STEPP: Subpopulation treatment effect pattern plot for individual patient data meta‐analysis. *Statistics in Medicine*, *35*, 3704-3716.

Wang, D. D., Shams-White, M., Bright, O. J. M., Parrott, J. S., & Chung, M. (2016). Creating a literature database of low-calorie sweeteners and health studies: Evidence mapping. *BMC Medical Research Methodology*, *16*, 1.

Webb, C., & Roe, B. H. (Eds.) (2007). *Reviewing research evidence for nursing practice: Systematic reviews*. Oxford: Wiley.

Weinhandl, E. D., & Duval, S. (2012). Generalization of trim and fill for application in meta-regression. *Research Synthesis Methods*, *3*, 51-67.

Whiting, P. F., Sterne, J. A., Westwood, M. E., Bachmann, L. M., Harbord, R., Egger, M., & Deeks, J. J. (2008). Graphical presentation of diagnostic information. *BMC Medical Research Methodology*, *8*, 20.

Winkler, T. W., Kutalik, Z., Gorski, M., Lottaz, C., Kronenberg, F., & Heid, I. M. (2014). EasyStrata: Evaluation and visualization of stratified genome-wide association meta-analysis data. *Bioinformatics*, *31*, 259-261.

Xie, M. G., & Singh, K. (2013). Confidence distribution, the frequentist distribution estimator of a parameter: A review. *International Statistical Review*, *81*, 3-39.

Yang, G., Cheng, J. Q., Xie, M. G., & Qian, W. (2017). gmeta [R software package]. Retrieved from <https://CRAN.R-project.org/package=gmeta>

Zhang, G., Kochunov, P., Hong, E., Kelly, S., Whelan, C., Jahanshad, N., ... Chen, J. (2017). ENIGMA-viewer: Interactive visualization strategies for conveying effect sizes in meta-analysis. *BMC Bioinformatics*, *18*, 253.

Zhao, J. H. (2007). A genetic analysis package with R. *Journal of Statistical Software*, *23*, 1-18.
